# Type 1 Diabetes-Associated Autoantibodies, Sera and T-Cell Receptor Sequences Recognize Myelin Basic Protein: A Novel Peripheral Diabetic Neuropathy–Multiple Sclerosis Connection

**DOI:** 10.3390/cells15151384

**Published:** 2026-07-31

**Authors:** Robert Root-Bernstein

**Affiliations:** Emeritus Professor, Department of Physiology, Michigan State University, East Lansing, MI 48824, USA; rootbern@msu.edu

**Keywords:** cellular immunity, humoral immunity, diabetes, multiple sclerosis, neuropathy, PDN, insulin, insulin-associated antigen, IA-2, PTPRN, GAD1, myelin basic protein, cross-reactivity, autoantibodies, *Clostridium*, coxsackievirus, pathogenesis

## Abstract

(1) Background: Neuropathies are common complications of type 1 diabetes (T1D) and previous research demonstrated concomitant demyelinization involving myelin basic protein (MBP). T1D patients have a significantly increased risk of multiple sclerosis (MS) and vice versa. The causes are unknown. (2) Methods: Proteonomic searches using BLAST were used to identify similarities between T-cell receptor (TCR) sequences from T1D and MS patients. LALIGN was used to explore whether T1D-related proteins mimic MBP. Enzyme-linked immunoadsorption assay (ELISA) was used to test for cross-reactivity between T1D-related antibodies or sera with MBP. Ultraviolet spectroscopy was used to test whether T1D-specific T-cell receptor (TCR) sequences recognized MBP. (3) Results: BLAST revealed that a subset of T1D and MS TCR are nearly identical. Sequence similarities were found by LALIGN between MBP and T1D-associated antigens. Antibodies against insulin, PTPRN(IA-2), and GAD1 recognized MBP. Most TCR sequences derived from T1D patients recognized MBP as an antigen. Most T1D-derived sera recognize MBP, but healthy sera do not. BLAST revealed that coxsackievirus proteins do not mimic MBP, but many *Clostridia* proteins do. (4) Conclusions: Cross-reactivity between TID-related antibodies and TCR with MBP may help to explain the pathogenesis of T1D neuropathies and the increased co-occurrence of these two diseases.

## 1. Introduction

Diabetic peripheral neuropathy (DPN) in type 1 diabetes mellitus (T1D) is characterized by a pattern of distal-to-proximal axonal damage to C and Aδ fibers associated with demyelination of large fibers [1]. The main cause of DPN is thought to be repeated or persistent hyperglycemia [2,3] causing damage to the Schwann cells that support myelinated nerves as a result of activation of polyol, hexosamine, protein-kinase C, advanced glycation end-product (AGE), oxidative stress, and poly (ADP ribose) polymerase pathways [4]. Both T1D and type 2 diabetes are significantly associated with risk for chronic inflammatory demyelinating polyneuropathy (CIDP), with reports of between 4 and 27% of CIPD patients also diagnosed with T1D [5,6,7,8,9]—reviewed in [10]. Demyelinization is also occasionally observed in diabetic retinopathies as well [11]. DPN in type 2 diabetes (T2D), on the other hand, is mainly associated with dyslipidemia, obesity, and hypertension [2,3,12] suggesting that either there are multiple causes of DPN or that an unrecognized factor may underlie the DPN risks in both types of diabetes. In fact, two recent serendipitous observations made in our lab suggest that there is a previously unsuspected immunologic mechanism at work in DPN.

The first serendipitous observation occurred while performing a proteonomic study of T-cell receptor (TCR) sequences derived from T1D patients. It was unexpectedly observed that a large number of these sequences were extremely similar to TCR sequences derived from multiple sclerosis (MS) patients and, more particularly, to TCR known to target myelin basic protein (MBP). Independently, while using MBP as a control protein in some enzyme-linked immunoadsorption assays (ELISA), it was observed that anti-insulin antibodies had significant affinity for MBP as well. The research reported here explores the hypothesis that cross-reactivity between diabetic autoantigens and peripheral nerve autoantigens may contribute to DPN pathogenesis, at least in some diabetic patients, and therefore that treatments for MS may benefit such patients and, conversely, insulin therapy may provide some benefits for MS and other neurodegenerative patients. What follows is a pilot study intended merely to test the plausibility of this hypothesis rather than assert its validity.

The hypothesis just stated is less radical than it may at first sound. Despite exhibiting distinct HLA patterns [13], more than ten percent of MS patients also exhibit T1D [14], a risk of T1D three to five times that of non-MS patients [15,16]. T1D patients correspondingly had more than three times the probability of being diagnosed with MS than healthy controls [17]. Moreover, increased insulin and insulin resistance are associated with myelin depletion in middle-aged people with cognitive impairments [18,19] and in particular with MS [20,21,22,23]. The basis for these increased comorbidities is not understood but may lie in part in shared autoantigens. Insulin and insulin-like growth factor 1 (IGF-1) are not only major targets of autoimmunity in T1D but also serve as neurotrophic factors regulating the expression of myelin proteins by Schwann cells [24,25,26,27]. Thus, interfering with insulin and IGF-1 activity decreases MBP production. Additionally, a striking correlation has been observed between recognition of insulin antibody producing cells and recognition of I-Ek MHC class II molecule of the PL/J haplotype in mice, which play a critical role in the development of experimental autoimmune encephalomyelitis (EAE), an animal model for multiple sclerosis (MS) based on cellular autoimmunity against myelin basic protein [28]. Another T1D autoantigen, glutamic acid decarboxylase (GAD), is also a target of autoimmunity in some demyelinating diseases. Autoantibodies to GAD65 correlated with decreases in brain grey matter volume specifically among the one third of MS patients with comorbid T1D [29] and sera with increased binding to GAD and the diabetes autoantigen Protein Tyrosine Phosphatase Receptor Type N (PTPRN, also known as Insulin-Related Antigen 2 (IA-2 or ICA512) also had increased binding to the unrelated antigen myelin oligodendrocyte glycoprotein [30]. Myelin basic protein was not tested in that study. Notably, PTPRN (IA-2) is expressed at axon initial segments and nodes of Ranvier in the central and peripheral nervous systems [31,32] so that T1D patients expressing anti-PTPRN (IA-2) antibodies might also inhibit peripheral nerve conduction. And finally, proteomic immunoprofiling has revealed a common set of neurological and immunological pathways shared by MS and T1D [33]. These commonalities may help to explain the fact that childhood and adolescent obesity are recognized risk factors not only for both types 1 and 2 diabetes but also for MS [34]. The research that follows explores additional aspects of possible relationships between T1D T-cell and antibody-mediated autoimmunity and cross-reactivity with myelin proteins as they may relate to shared diabetes–MS symptoms and risks. To reiterate, these data are preliminary and intended only to test the plausibility of the hypothesis, not as an attempt to prove it.

## 2. Materials and Methods

### 2.1. Outline

The preliminary tests of the hypothesis consist of four basic types of explorations. One involves an explicit study of whether T1D TCR share an unusual degree of overlap with MS TCR with appropriate controls. A second involves using UV spectrophotometry to test whether some of the T1D TCR actually bind to MBP. The third type of test involves enzyme-linked immunoadsorption assays (ELISA) to test whether antibodies associated with T1D (e.g., against insulin, PTPRN, and GAD1) recognize MBP and whether sera from T1D patients (or healthy controls) bind to MBP. Finally, LALIGN is used to test whether any similarities exist between MBP and T1D autoantigenic proteins that might help to explain any antibody cross-reactivity.

### 2.2. Proteins

Sterile human myelin basic protein, purified to GMP standards by Eli Lilly Laboratories (291-61E-261, 22 June 1983) as part of potential multiple sclerosis treatment project [35,36]. Lyophilized and stored at −80 °C.

Sterile porcine myelin basic protein, purified to GMP standards by Eli Lilly Laboratories (CD3605-7A, December 1978) as part of a human multiple sclerosis trial [35,36]. Lyophilized and stored at −80 °C.

Human recombinant insulin, >98% purity, SAFC (St. Louis, MO, USA) 91077C.

Human recombinant glucagon, >98 percent purity, Sigma-Aldrich (St. Louis, MO, USA) G2044.

Human PTPIA2 (PTPRN or IA2) active human recombinant, expressed in *E. coli*, ≥90% (SDS-PAGE) Sigma-Aldrich (St. Louis, MO, USA) SRP0221.

Human GAD1 Acro Biosystems GA1-H5543 (1 Innovation Way, Newark, DE, USA).

T cell receptor sequences from type 1 diabetic patients, synthesized and purified to at least 95% purity (by mass spectrometry) by RS Synthesis (Louisville, KY, USA). Sequences were derived from three patients. TCR 1, 2, and 4 were from the pancreatic draining lymph nodes of one patient [37]; TCR 8, 9, and 10 from the pancreatic draining lymph nodes of a second patient [37]; and TCR K2.4, K2.12, and K2.16 from the peripheral blood lymphocytes of a third patient [38]. Notably, the draining lymph node and peripheral blood lymphocytes were highly homologous: TCR K2.12 and TCR 2 [39] were highly homologous, so only the TCR 2 sequence was synthesized. TCR K2.16 and TCR 4 were also highly homologous [39,40], so only TCR 4 was synthesized. Three of the TCR shared sequence similarity (4, 8, and 9) so an additional consensus sequence was synthesized. It is also notable that TCR K2.12 and TCR 10 are almost identical to TCR from multiple NOD mice [41] and similarities to TCR 1, 2, 8, 9, K2.4, and K2.16 are also evident with mouse NOD TCR [39,40,42,43,44]. Thus, the interactions between the TCR sequences synthesized here are likely to be characteristic of diabetic and NOD TCR from multiple patients as well as diabetic animals.

### 2.3. SERA

LEE Biosolutions (now Medix Biochemica USA), 10850 Metro Court, Maryland Heights, MO 63043 USA.

Innovative Research, Inc., 46430 Peary Court, Novi, MI 48377 USA.

Zen Bio Inc., 3920 S Alston Ave, Durham, NC 27713.
**Disease State****Supplier****Our ID****Sample ID****Age****Sex****Race****HbA1c %****Serum Treatment**T1DLee BiosolutionsLEE 0109E5731 A1c-50.1139MCaucasian11.5EDTAT1DLee BiosolutionsLEE 0209E5731 A1c-50.1236FCaucasian14.4EDTAT1DLee BiosolutionsLEE 0309E5731 A1c-0329FCaucasian10.3EDTAT1DLee BiosolutionsLEE 0409E5731 A1c-0430MCaucasian10.5EDTAT1DLee BiosolutionsLEE 0509E5731 A1c-0524FCaucasian11.3EDTAT1DInnovative Research#69HMN88906961MCaucasian
Off the clotT1DInnovative Research#70HMN88907036MAsian
Off the clotT1DInnovative Research#71HMN88907156FCaucasian
Off the clotHealthyZen-BioHealthy 1HSER-2ML?FCaucasian
Off the clotHealthyInnovative ResearchHealthy 8639521-08646MAfrican American
Off the clotHealthyInnovative ResearchHealthy 8739521-08736FAfrican American
Off the clotHealthyInnovative ResearchHealthy 8839521-08840MAfrican American
Off the clotHealthyInnovative ResearchHealthy 8939521-08959MAfrican American
Off the clot

### 2.4. Antibodies

Goat anti-human, horse radish peroxidase (HRP) labeled (END Millipore Corp., 290 Concord Rd., Billerica, MA, USA) AP112P.

Goat anti-guinea pig, horse radish peroxidase (HRP) labeled, Invitrogen (Carlsbad, CA 92008, USA) A18769.

Goat anti-rabbit IgG-HRP, Invitrogen (Carlsbad, CA 92008, USA) 65-6120.

Rabbit polyclonal Anti-GAD65 Antibody, Sigma-Aldrich (St. Louis, MO, USA) ABN101.

Rabbit anti-PTPRN, Sigma-Aldrich (St. Louis, MO, USA) HPA007179.

Rabbit anti-Insulin (INS). Sigma-Aldrich (St. Louis, MO, USA) HPA004932.

Guinea pig anti-Insulin, Abcam (Waltham, MA 02453, USA) ab7842.

Guinea pig anti-insulin, Millipore (290 Concord Rd., Billerica, MA, USA) AB3440.

Guinea pig anti-insulin Invitrogen (Carlsbad, CA 92008, USA) PA1-296938.

### 2.5. Chemicals

Tween 20, Sigma-Aldrich Chemical Company (St. Louis, MO, USA), P1379.

Polyvinyl alcohol, MW 13,000-23,000, Sigma-Aldrich Chemical Company (St. Louis, MO, USA) 363170.

ABTS Single Reagent (2-2′-AZINO-bis (3-ethylbenziazoline-6-sulfonic acid) EMD Millipore Corporation (USA) ES004.

pH 7.0 phosphate buffer, ThermoFisher Scientific (Fair Lawn, NJ, USA) 258590025.

pH 4.63 sodium acetate/acetic acid 0.1 M, ThermoFisher Scientific (Fair Lawn, NJ, USA).

### 2.6. Equipment

SPECTRAmax Ultraviolet and visible light microplate spectrophotometer, Molecular Devices (Sunnyvale, CA, USA).

96-well ELISA plates, ThermoFisher Scientific (Fair Lawn, NJ, USA).

### 2.7. Programs

SOFTmax PRO, Version 4.0, Molecular Devices (Sunnyvale, CA, USA).

Microsoft Excel Office 365 2024.

### 2.8. UV Spectroscopy Study of TCR-Antigen Binding

Ultraviolet (UV) spectroscopy was used to determine whether the TCR sequences bound to human MBP. The MBP was dissolved at 0.05 mM in pH 7.0 phosphate buffer, and 50 µM solutions were then made for use in spectroscopy. The TCR peptides were dissolved in the same buffer at 0.5 mM and then serially diluted eight times by thirds so that the range of concentrations was such that at the low end of the concentrations, no binding would be observable, while at the high end of the concentrations, the binding would saturate. The results could then be plotted in a Scatchard plot from which binding constants could be determined. A flat line resulted if there was no binding and an “S”-shaped curve if binding was evident. Binding constants were determined from the inflection point of the curves. An amount of 100 µL of each TCR concentration plus 100 µL of buffer; mixtures of 100 µL MBP with 100 µL of buffer; and 100 µL of MBP with 100 µL of each TCR concentration were made in a crystal 96-well plate and a complete spectrum from 190 nm to 250 nm of each well recorded on a Spectromax Plus spectrophotometer. Data were collected using SoftMax Pro 4.0 software. Every combination was done in duplicate and the results averaged. These curves were examined for the absorbance at which the greatest spectral shifts occurred (usually 205 nm). The absorbance of a mixture of the hormone or IR peptide with TCR peptide was compared with the sum of the absorbances of the hormone or IR peptide (plus buffer) and TCR (plus buffer), and the differences plotted as a function of the concentration of the TCR peptide.

### 2.9. Enzyme-Linked Immunoadsorption Assay (ELISA)

Quantitative ELISA was used to determine whether the various antibodies used in the study cross-reacted with proteins other than the ones to which they were raised. As with the UV spectrometry studies, the proteins were serially diluted to be able to obtain Scatchard plots from which binding constants could be determined. Proteins were made up at 2 mg/mL in buffer solution (pH 7.0 except for insulin and glucagon, which were solubilized in pH 4.63 buffer) and serially diluted ten times by thirds. An amount of 100 µL of each dilution was put in duplicate wells in a 96-well ELISA plate. Plates were incubated at room temperature on a shaker for 1 h and then triple-washed with 2% Tween 20 in pH 7.0 phosphate buffer. Then 200 µL of polyvinyl alcohol blocking agent (1 g/L of buffer) was added to each well; the plate incubated for 1 h; and triple washed with 2% Tween 20 in phosphate buffer. Next, 100 µL of primary antibody diluted to 1/500 in buffer was added to each well; the plate incubated for 1 h; and triple washed with 2% Tween 20 in phosphate buffer. Then, 100 µL of the appropriate secondary antibody (HRP-labelled) diluted to 1/1000 in buffer was added to each well; the plate incubated for 1 h; and triple washed with 2% Tween 20 in phosphate buffer. Finally, 100 µL of ABTS single reagent was added to each well, incubated for 15 min, and the plate read in the spectrophotometer at 405 nm.

### 2.10. Proteonomics

Homologies between T1D TCR sequences and autoimmune myocarditis (AM) TCR sequences were determined by using BLAST. Seventy-three T1D TCR sequences [37,38,45,46,47] were entered into BLAST, searching the human UNIPROT database (taxid 9606) for MS TCR homologies (see Supplementary Material S1). The same operation was performed with 34 autoimmune myocarditis (AM) TCR (complete list of sequences at [48] and see Supplementary Material S2) and with a random selection of 150 TCR sequences from healthy individuals [49] (sequences provided in Supplementary Material S3), again using BLAST and searching the human UNIPROT database for MS TCR homologies. In this way, the probability of T1D TCR mimicking MS TCR could be compared with the probability of AM TCR and healthy TCR sequences mimicking MS TCR. The significance of the differences was determined by Fisher’s exact Chi Square test (see Statistics below).

Protein–protein BLAST: National Library of Medicine, USA. https://blast.ncbi.nlm.nih.gov/Blast.cgi?PROGRAM=blastp&PAGE_TYPE=BlastSearch&LINK_LOC=blasthome Maximum sequences to show, 1000; Expected value 1.0; word size 2; BLOSUM80 to maximize identification of short similarities (accessed on 15 May 2026).

The possibility of sequential similarities between various antigens associated with T1D pathologies (e.g., insulin or PTPRN with human myelin basic protein) was explored for all the proteins listed above using LALIGN, a proteonomic comparison program that compares pairs of sequences.

LALIGN: https://fastademo.bioch.virginia.edu/fasta_www2/fasta_www.cgi using UNIPROTKB accession numbers and BLOSUM80 matrix or PAM250 (Accessed on 15 May 2026).

Results of both LALIGN and BLAST searches were triaged to eliminate any matches that lacked at least 6 identical amino acids within a 10 amino acid sequence. This added criterion was used to identify matches likely to be similar enough to be recognized by the same T cell receptor sequence or antibody sequence.

The choice of 6 identical amino acids in a sequence of 10 was made based on several criteria and was validated in previous studies. Briefly, the incidence of 6 identities in 10 amino acids in randomly selected proteins is 6% (*p* = 0.06) [50]. Secondly, raw scores (E) are used by LALIGN and BLAST to derive a function that calculates the probability that an alignment with such a score is likely to occur if any peptide of an equivalent length were used to search 10 000 proteins of equivalent length to the receptor or transporter sequence. Raw scores of 32 or above generally correspond to probabilities of less than 1 in 15 that such alignments occur by chance [*p* < 0.07] while raw scores above 40 generally correspond to probabilities of less than 1 in 50 that such alignments occur by chance (*p* < 0.02) [39]. Thus, 6/10 amino acid identities correspond to statistically significant similarities.

Additionally, this criterion was employed because various experimental studies have demonstrated that this degree of similarity is often sufficient to result in antibody cross-reactivity between peptides [51,52,53,54], and these criteria have been used in previously published mimicry studies by other laboratories (e.g., [55,56,57]).

Finally, with specific reference to TCR recognition of antigens, it is important to recognize that the average length of peptide recognized by a TCR is 8 to 11 amino acids [58] and in the case of myelin basic protein, peptides as short as 5 amino acids in length can be recognized with great specificity [59]. Secondly, any given TCR sequence, including those produced in T1D, may recognize up to a million peptides [60,61] so that the interactions are not very specific nor can they be highly sequence specific. And thirdly, X-ray crystallographic studies demonstrate that only about half of the amino acids in the TCR sequence are directly involved in peptide binding, and these amino acids are not necessarily, or even often, sequential, but often alternate, sometimes apparently randomly so [61,62,63]. Thus, using a criterion for TCR similarity of at least 6/10 amino acid identities in a sequence of 10 amino acids is justified by experimental evidence.

In this study, over half of the T1D TCR shared 8 or more amino acids in a sequence of 10 with MS TCR so that the homologies reported here generally transcend the selection criteria. Applied consistently, these criteria make possible statistical comparisons with the control groups.

### 2.11. Statistics

The statistical significance of the prevalence of T1D TCR mimicking MS TCR as compared with the prevalence of AM TCR and TCR sequences from healthy individuals was determined using Fisher’s exact Chi Square calculator: GraphPad https://www.graphpad.com/quickcalcs/chisquared2/ (accessed on 25 May 2026). A Bonferroni correction was applied for the three comparisons made which brought the calculated *p* values (all <0.0001) to a significance of *p* < 0.00033. https://www.statology.org/bonferroni-correction-calculator/ (accessed on 26 June 2026).

Since all ELISA experiments were performed only in duplicate due to very limited supplies of the key proteins, the results were evaluated for acceptability using the standard criterion in that the resulting values varied between duplicates by less than 10%. Error bars are not provided, also consistent with standard practice when averaging duplicates. In any event, the absence of error bars can alter the inflection point of the resulting ELISA curves only to the extent of the standard error, which is calculated as the difference between the two measurements (less than 10% difference) divided by two (the number of replicants). Thus, the most that the reported binding constants can be “off” is five percent, which can have no impact on the interpretation of the results reported here. The only important result is whether there is binding or not, and whether the binding to MBP is within an order of magnitude of the binding to T1D antigens. Statistics are not needed to make that type of determination and would imply a degree of confidence that these experiments do not warrant.

## 3. Results

**Figure 1:**
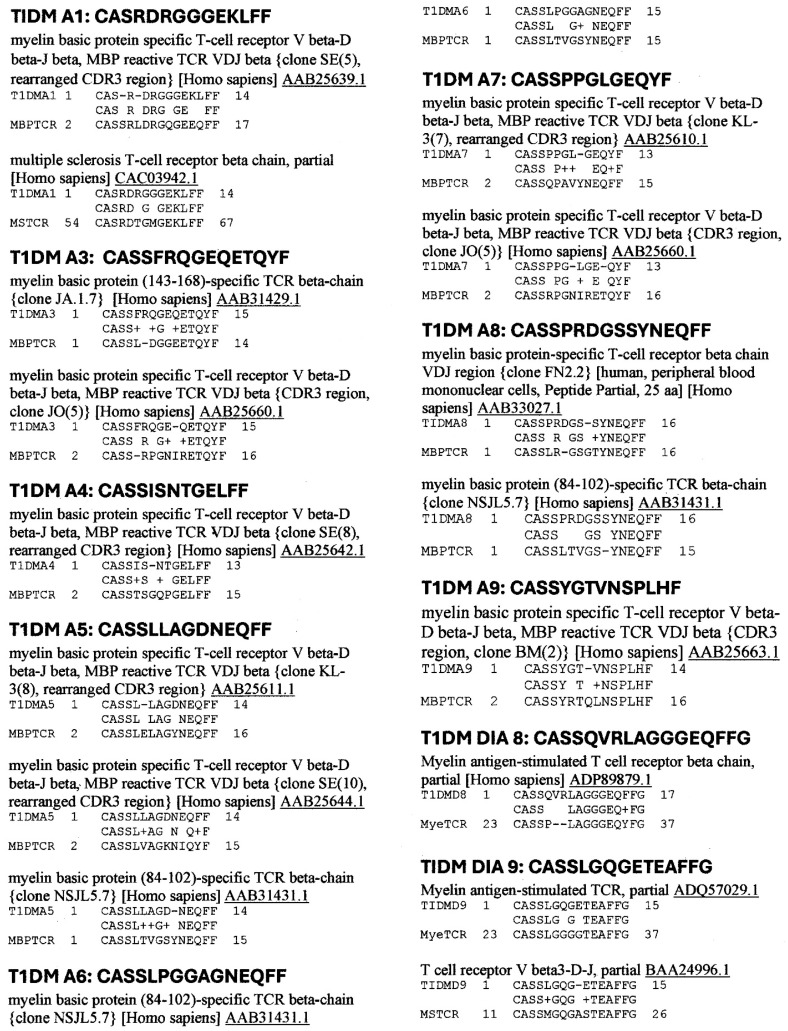

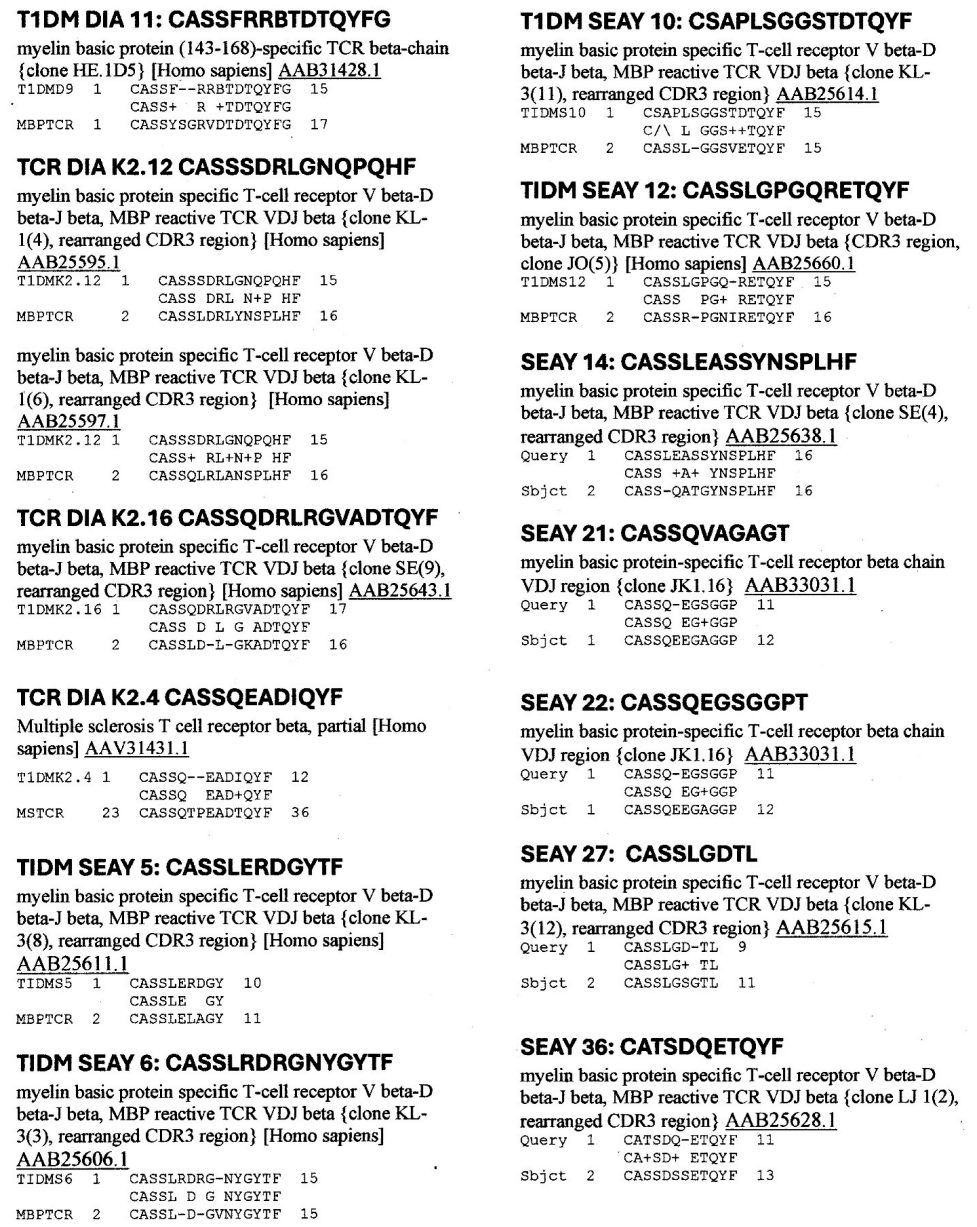
Selection of T1D TCR that mimic MS TCR. Note that many of the MS TCR are identified as being MBP specific. A complete listing of all the T1D–MS TCR matches found is supplied in the Supplementary Material. The titles of the TCR (e.g., T1D DIA 11) refer to the identities of the TCR sequences provided in Supplementary Material S1 and are followed by the TCR sequence. The underlined codes are the UNIPROT KB identification numbers of the MS TCR sequences.

The results of this study indicate that many diabetic patients produce both T cells and antibodies that can target myelin basic protein. Beginning with the data concerning T cell recognition of MBP, BLAST was used to compare 73 T cell receptor (TCR) sequences derived from T1D patients and 34 TCR sequences derived from autoimmune myocarditis (AM) patients to the UNIPROT human proteome database and evaluated for the number of matches to multiple sclerosis (MS) TCR sequences. Table 1 summarizes the results. The T1D TCR exhibited significantly more matches of higher quality to MS TCR than did the AM TCR (Chi squared = 41.564, *p* = 2.05 × 10^−8^ resulting in *p* < 0.00033 after Bonferroni correction). Over half of all the T1D TCR exhibited a sequence identity of at least 8 residues within a 10 amino acid stretch whereas only 20% of the AM TCR did so and none of the random protein sequences. Many of the T1D TCR matches extended well beyond 10 amino acids, making such T1D TCR almost identical to the MS TCR (see Figure 1 and Supplementary Material S1), which was never the case for the AM TCR or Healthy TCR (see Supplementary Materials S2 and S3). As Figure 1 shows, the majority of these matches involved MS TCR that were known to be specific for MBP. The high degree of similarity between the T1D TCR and the MS TCR suggested the possibility that some T1D TCR might therefore target MBP.

**Figure 2:**
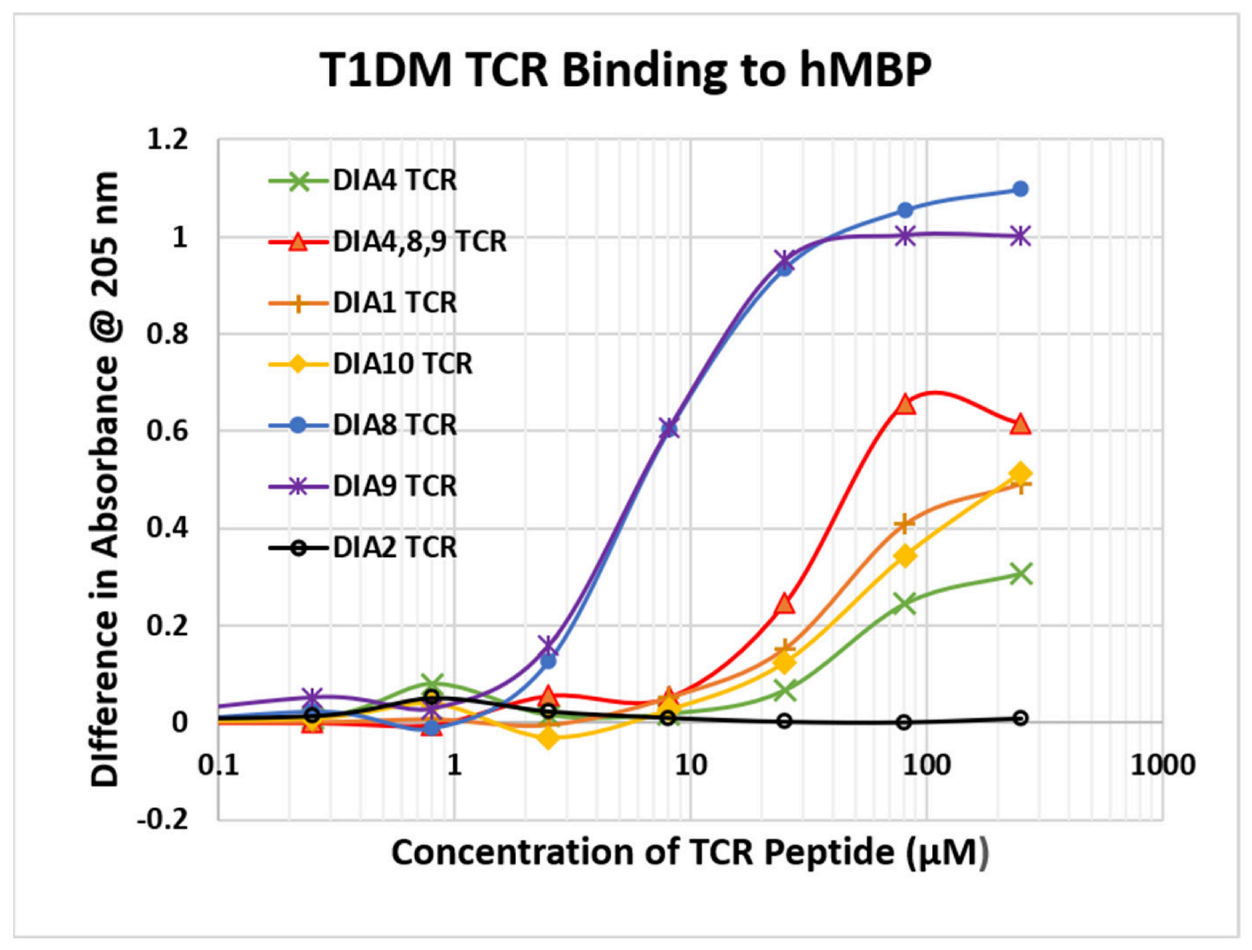
Ultraviolet spectroscopy experiment evaluating the binding of type 1 diabetic T cell receptor sequences (DIA TCR) to human myelin basic protein (hMBP). Binding constants can be approximated by finding the concentration of TCR peptide at the inflection point of each curve. However, because these experiments were run in solution, while actual TCR-MBP binding would involve cell-cell interactions, the calculated affinities probably significantly underestimate the actual affinity of the TCR for MBP in vivo. The TCR labels refer to the sequences listed in Figure 1 and Supplementary Material S1. TCR4,8,9 is a consensus sequence derived from DIA4, DIA8, and DIA9.

To test whether the T1D–MS TCR similarities predicted possible functional similarities as well, six of the T1D TCR sequences were synthesized. All these T1D TCR were sequences common to multiple T1D patients as well as NOD mice (see Materials and Methods for additional information) and all displayed some degree of similarity to MS TCR (see Figure 1). Ultraviolet spectroscopy was employed to determine whether these TCR sequences bound to human MBP. The results are shown in Figure 2 and Table 2. Binding constants varied between about 10 μM and 100 μM, which may not seem clinically significant, but it must be remembered that the UV experiments reported here involve TCR and protein free in solution. Thus, each protein has three degrees of thermodynamic freedom making the probability of aligning in such a way as to bind specifically fairly low, whereas within the body, both the TCR and MBP sequences are embedded in cell membranes and can exhibit only one degree of thermodynamic freedom each. Thus, the likelihood of binding and the actual affinity of these TCR for MBP are very probably much higher in vivo than measured here in vitro. Thus, T1D TCR can likely target MBP with significant affinity. Whether this binding results in T cell activation, however, is beyond the ability of this technique to address.

The approximate affinities of the diabetic TCR for MBP as calculated from the inflection points of the curves are summarized in Table 2, which also compares the affinity of the same TCR for other diabetes-related antigens, such as insulin glucagon, and regions of the insulin receptor that were reported in a previous study by this laboratory [40]. Note that there is a reasonably good correlation between the TCR binding to MBP and binding to insulin, but less so for the other antigens. Binding constants are approximate and valid within about 5% of the stated values (see Materials and Methods).

**Figure 3:**
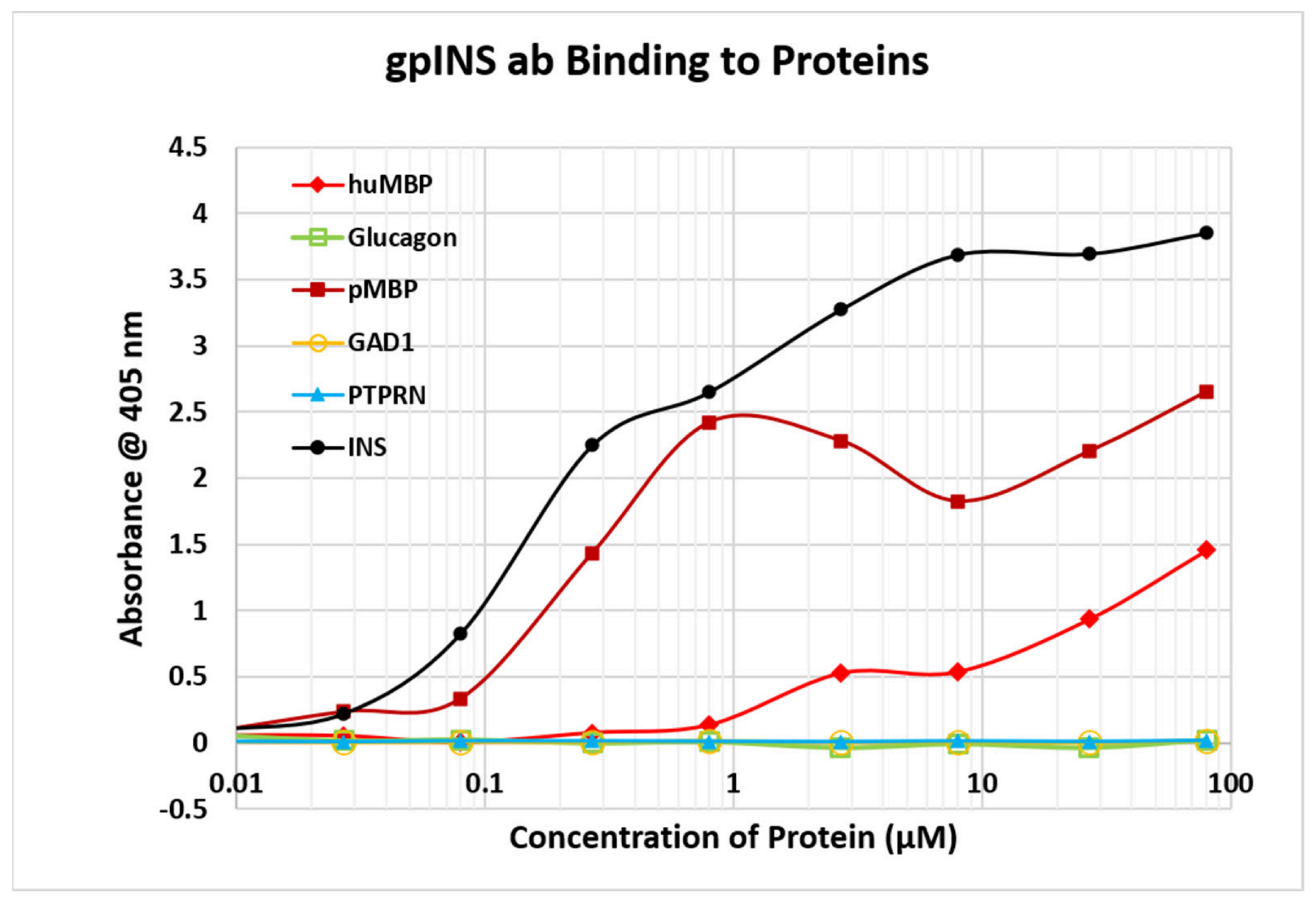
Results of enzyme-linked immunoadsorption assay using guinea pig anti-human insulin antibody binding to human myelin basic protein (huMBP), human glucagon, porcine myelin basic protein (pMBP), glutamic acid decarboxylase (GAD1), Protein Tyrosine Phosphatase Receptor Type N (PTPRN), and insulin (INS). The double curves for huMBP and pMBP suggest that insulin antibody recognizes one high affinity and one very low affinity site on MBP. Note that gpINS antibody displayed no observable binding to glucagon, GAD1, and PTPRN at the concentrations of peptide and antibody used in these experiments.

As noted in the Introduction, not only was it serendipitously observed that T1D and MS TCR seemed to be unusually similar but it was also serendipitously observed that insulin antibody bound significantly to MBP as well. These serendipitous results were replicated for this study and are presented in Figure 3. Note that insulin antibody does not bind very strongly to insulin [64,65,66] and that it bound to porcine MBP with almost equal affinity—thus the surprise that caught this investigator’s attention.

**Figure 4:**
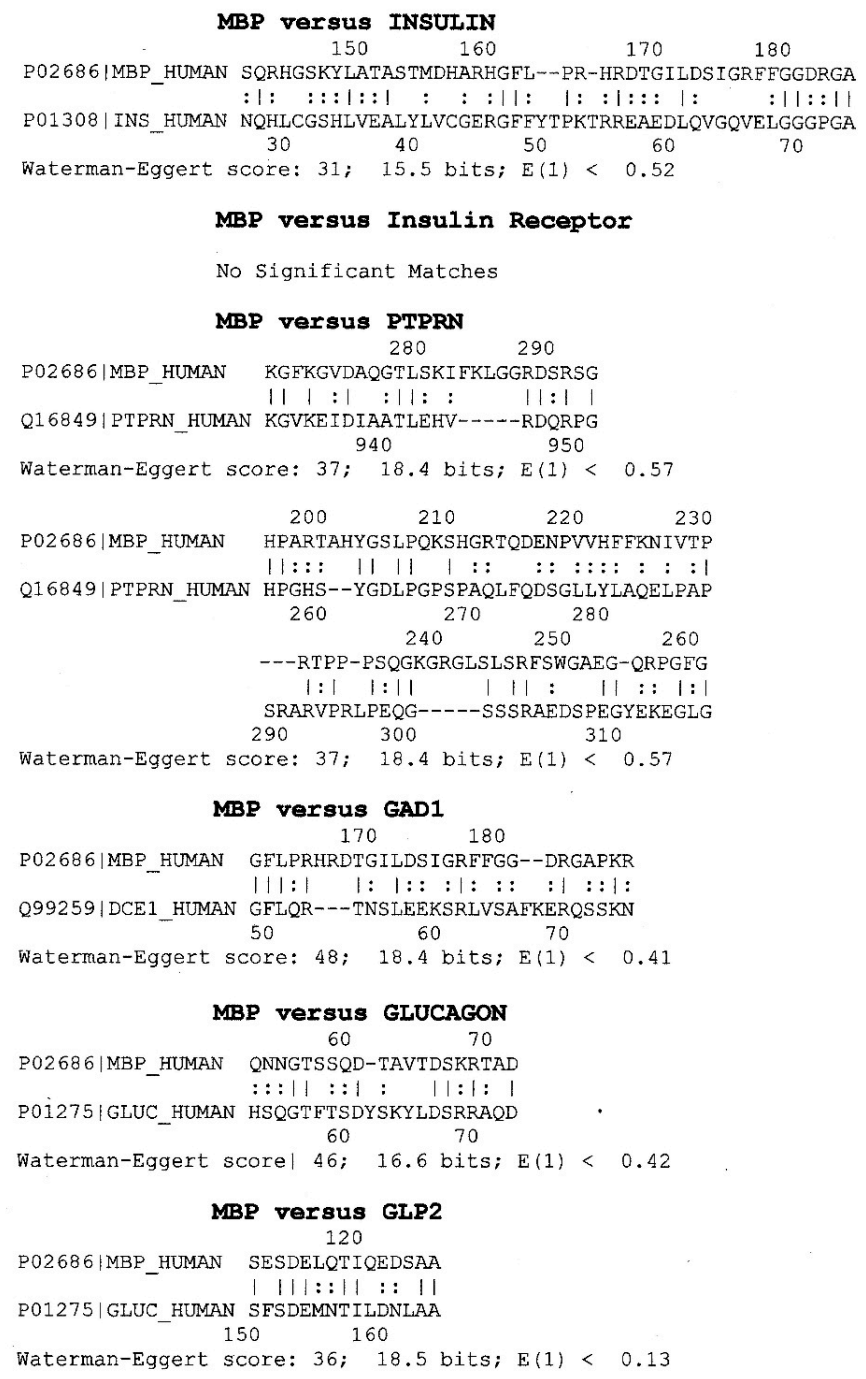
LALIGN results for myelin basic protein (MBP) homologies with diabetes-related proteins; INSULIN is the entire insulin protein including A, B, and C peptides; PTPRN is Protein Tyrosine Phosphatase Receptor Type N; GAD1 is glutamic acid decarboxylase type 1; GLP2 is glucagon-like peptide type 2. NB: No significant homologies were found to any diabetes-associated antigen and P02689|MYP2_HUMAN Myelin P2 protein, or between MBP with the insulin A or B chains, or with glucagon or glucagon-like peptide receptors.

One possible reason for the experimentally observed cross-reactivity between insulin antibody and MBP was explored by using LALIGN to determine whether the two proteins share significant sequence similarity. Figure 4 shows that while similarity between MBP and the insulin C-peptide region was observed, no sequence similarities were observed between MBP and the insulin A or B peptide regions. Significant similarities were observed between MBP and other diabetes-related proteins, however, including GAD1, PTPRN (IA-2), glucagon, and glucagon-like peptide 2.

**Figure 5:**
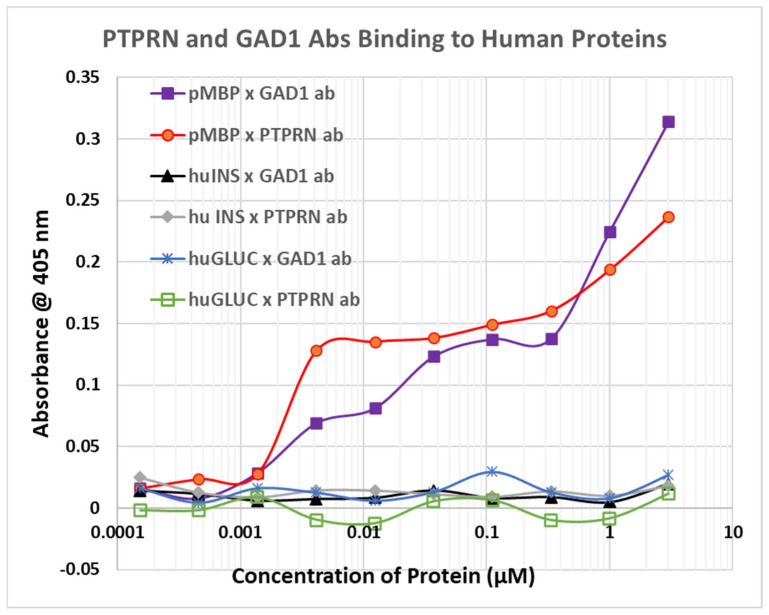
Results of enzyme-linked immunoadsorption assays involving the binding of guinea pig antibodies against glutamic acid decarboxylase type 1 (GAD1) and Protein Tyrosine Phosphatase Receptor Type N (PTPRN) to porcine myelin basic protein (pMBP), human insulin (huINS), and human glucagon (huGLUC). The GAD1 antibody curve suggests that it may recognize as many as three regions of pMBP with different affinities, while the PTPRN antibody reveals one high affinity binding site and one very low binding site. See Table 3 for binding constants. Note that neither GAD1 nor PTPRN antibodies bound to huGLUC or huINS at the concentrations of antibody and peptides used in these experiments.

Because similarities were observed between both GAD1 and PTPRN (IA-2) with MBP (Figure 4), polyclonal antibodies against GAD1 and PTPRN were tested for binding to MBP using ELISA. Both GAD1 and PTPRN (IA-2) recognized human MBP as an antigen but did not bind to human insulin or glucagon (Figure 5). These antibodies displayed higher affinity for MBP than did insulin antibodies (see Figure 4 and Table 3).

**Figure 6:**
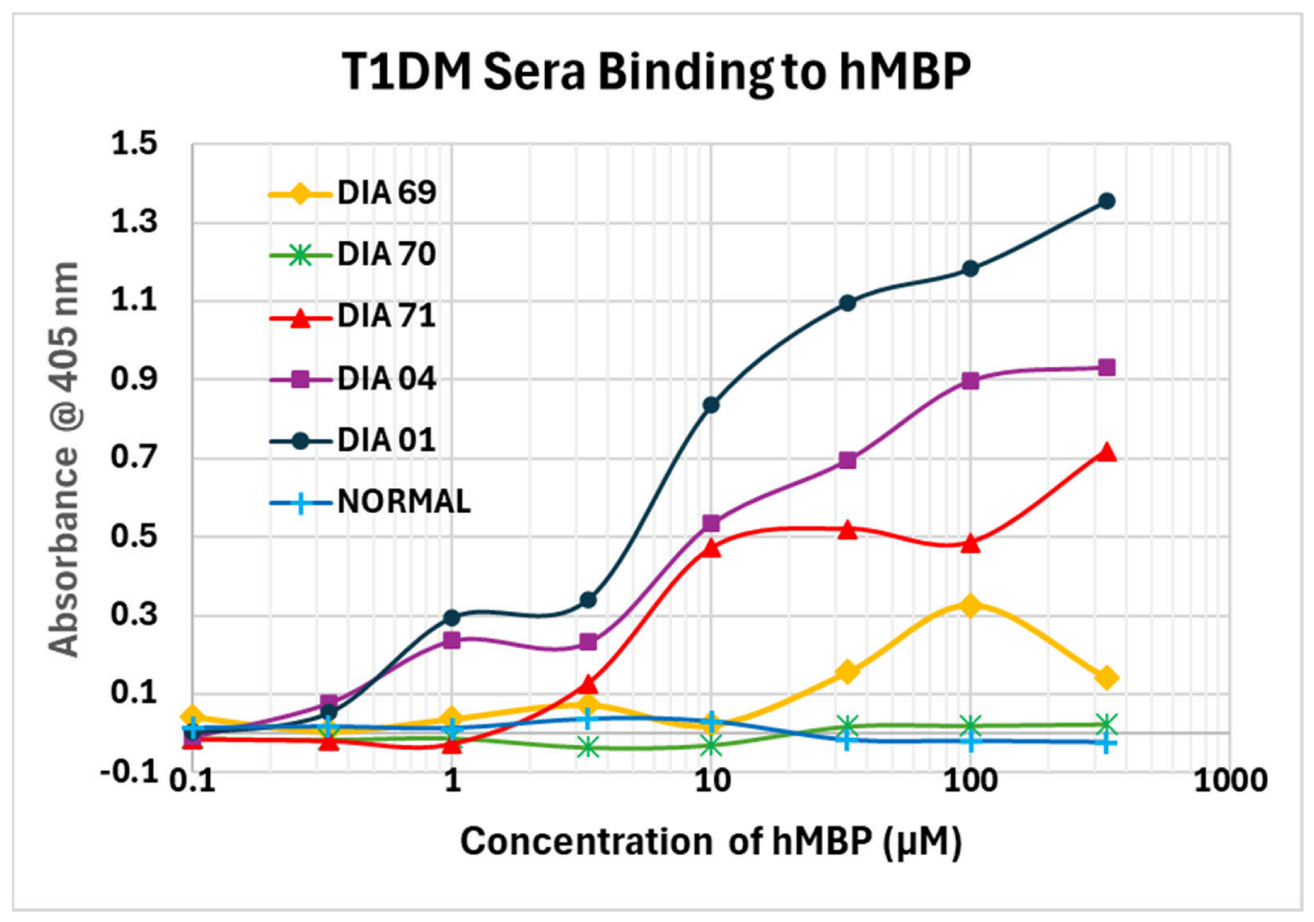
Results of enzyme-linked immunoadsorption assays involving sera from type 1 diabetes mellitus patients (T1D) or healthy controls binding to human myelin basic protein (hMBP). DIA 1, DIA 2, etc. refer to the sera codes (see Materials and Methods). Only some of the experimental curves are provided so that the differences in the curves can be seen clearly. In particular, most of the negative results (flat lines) were omitted because they overlay each other to such an extent that they cannot be differentiated. This omitted data and the resulting binding constants are shown in Table 4.

The next question was whether sera from type 1 diabetic patients also recognized MBP. Sera from eight T1D patients and five individuals without diabetes were tested using ELISA to determine their affinity for human MBP. Results are shown in Figure 6 and Table 4. None of the sera from healthy individuals bound to MBP. Sera from T1D patients varied in their affinity for MBP ranging from 0.6 to 0.8 μM to around 10 μM or, in some cases, not at all. It is not possible from these experiments to determine the original specificity of the antibodies binding to MBP nor was any information available concerning whether any of the donors exhibited symptoms of peripheral or other neuropathies.

**Figure 7:**
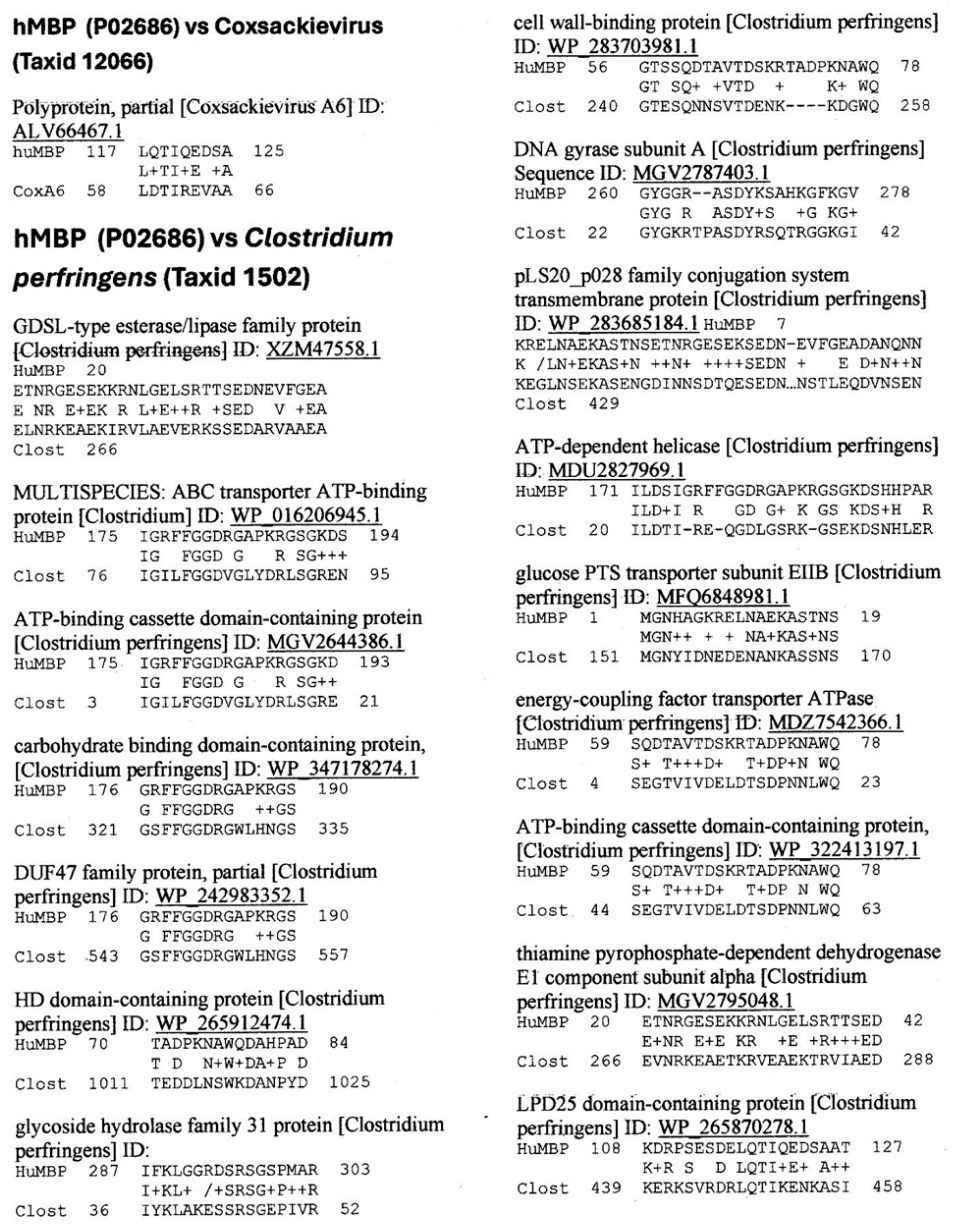
Results of a BLAST study of similarities between human myelin basic protein (huMBP) and coxsakieviruses or *Clostridium perfringens* (Clost). The BLAST sequences were triaged by eliminating any sequences that had less than six identical amino acids in a ten amino acid-long sequence. This selection procedure ensured that the resulting matches were sufficiently long and sufficiently similar so as to have a reasonable chance of being recognized by the same T cell receptor sequences (Figure 1). Only one coxsackievirus sequence satisfied this criterion (shown here). This Figure illustrates some of the many similarities between human MBP and *Clostridium perfringens,* while Supplementary Material S4 lists many additional similarities. Thus, *Clostridia* are a far more likely source of antibodies that might cross-react with MBP than are coxsackieviruses.

Finally, previous results from this laboratory linked T1D onset to combined coxsackievirus–*Clostridium* infections [48,52] and Table 4 includes data from one of those studies showing a correlation between T1D sera binding to both insulin and to *Clostridium sporogenes* antigens. Table 4 also reveals that T1D sera which bind to insulin and *Clostridium* antigens also tend to bind to hMBP. In the light of the fact that *Clostridia* have also recently been proposed to be triggers for MS [67,68,69,70], a further set of BLAST studies was performed to test whether *Clostridium perfringens* proteins might mimic hMBP. Figure 7 and Supplementary Material S4 demonstrate that they do. Several dozen *C. perfringens* proteins mimic hMBP such that within a sequence of ten amino acids (an approximation of the peptide length recognized by T cell receptors), six or more identical amino acids are shared by the hMBP and the bacterial protein. Figure 7 also shows that an identical search of coxsackieviruses, which are widely considered to be the major infectious triggers of T1D [71,72,73,74,75,76] yielded only one such similarity. Thus, while coxsackievirus proteins do not mimic either insulin or MBP, many *Clostridia* proteins mimic both.

## 4. Discussion

### 4.1. Summary of Results

The results presented here provide evidence that type 1 diabetes may induce antibodies and T cells that can cross-react with myelin basic protein thereby augmenting the demyelinization that accompanies diabetic peripheral neuropathies. While the impetus for this research was the serendipitous observation that insulin antibodies cross-reacted with porcine MBP, it is evident that antibodies against other T1D antigens, such as GAD1 and PTPRN (IA-2), may have higher affinity for human MBP and thus be of greater significance for understanding the pathogenesis of T1D peripheral neuropathies. The experiments involving sera derived from T1D patients demonstrate that such MBP-reactive antibodies are not present in all T1D patients, but most have antibodies that express some degree of cross-reactivity. A relationship clearly exists between sera that bind to MBP and sera that bind to insulin, but the correlation is not perfect, again suggesting that antibodies against other T1D antigens such as GAD1 and/or PTPRN (IA-2) may be responsible. Lack of sufficient antigens (and funding) prevented this possibility from being fully explored and these results should be considered preliminary tests of the hypothesis that T1D and MS share common immunologic features.

T cell-mediated autoimmunity may also be at work in the pathogenesis of T1D-associated neuropathies. BLAST studies revealed that an extraordinary number of T1D-associated TCR sequences were very similar and, in some cases, nearly identical to those associated with multiple sclerosis but shared significantly less similarity to TCR associated with autoimmune myocarditis or TCR expanded in healthy individuals. UV spectroscopy experiments confirmed that some of these T1D TCR sequences bound to MBP but, once again, these are preliminary experiments that must be interpreted carefully. Binding of antigens to TCR does not necessarily mean that the T cells will be activated, so cellular assays are necessary to explore whether T1D T cells from patients with neuropathies do, or do not respond actively to MBP.

Conversely, the possibility that TCR and antibodies specific for MBP in MS patients may cross-react with T1D autoantigens, such as insulin, the insulin receptor, GAD1, and/or PTPRN (IA-2), also needs to be tested with appropriate sera and T cells from MS patients.

Notably, coxsackieviruses, which are often cited as the most likely triggers for T1D, have almost no similarities with MBP, insulin, GAD1, or PTPRN (IA-2), while *Clostridia*, which do share such similarities with T1D antigens, also exhibits a very large number of similarities with MBP. Thus, the results reported here are inconsistent with coxsackieviruses being the main trigger for either anti-insulin or anti-MBP autoimmunity in T1D and point to *Clostridia*, or possibly other related bacteria, as possible triggers. Identification of *Clostridia* as a potential trigger for anti-MBP autoimmunity in T1D is consistent with recent research that identified *Clostridia* as likely triggers of MS as well.

In summary, the results reported here suggest that antibodies and TCR against T1D antigens may be cross-reactive with MBP providing an autoimmune mechanism linking T1D and MS and perhaps helping to explain why T1D patients have increased risks of developing MS and MS patients have increased risks of developing T1D.

The key questions raised by these results are why very few MS patients develop T1D and very few T1D patients develop MS. The answer likely involves access to the relevant hidden antigens, starting with T1D-associated peripheral neuropathies.

### 4.2. Possible Mechanisms of Demyelinization in T1D

The myelin sheath is a complex tissue comprised of lipids and multiple proteins that wraps around peripheral nerves. MBP is one of several “hidden antigens” embedded within the sheath. None of these proteins are directly accessible to the immune system. Thus, the cross-reactive antibodies, sera, and TCR identified in this study cannot directly cause the demyelinization that characterizes many T1D-associted peripheral neuropathies. The role of these antibodies and TCR is likely to augment myelin damage due to other more direct causes and to inhibit or prevent remyelinization. Thus, other mechanisms associated with T1D peripheral neuropathies, such as hyperglycemia leading to advanced glycation end products (AGEs) that impair myelin production and stability [72,73], are likely to be necessary precursors that permit autoantibodies and T cells to then further disrupt the myelin and prevent remyelinization. Thus, preventing or correcting the processes that initiate destabilization of the myelin sheath may prevent MBP-reactive autoantibodies and T cells from being able to effectuate myelin destruction.

Positing an indirect or secondary role played by anti-MBP autoimmunity in T1D would help to explain why demyelinization is limited in its expression to T1D-associated peripheral neuropathies without the vast majority of T1D patients developing MS. On the other hand, the link between T1D autoimmunity and cross-reactivity with MBP may also provide an explanation for the observed increased risk of MS in some T1D patients. There may be shared triggers such as *Clostridia* [48,52,77,78,79] that can induce both insulin and MBP-reactive antibodies and TCR (see Figure 7). The fact that MS and T1D autoimmune pathologies overlap only partially, however, suggests that perhaps in T1D, *Clostridia* synergize with other more widely recognized T1D triggers such as coxsackieviruses or other enteroviruses [80,81,82]. Only rarely would MS-specific triggers also be present in T1D patients.

### 4.3. Probable Mechanisms of Insulin Resistance in MS

The similarities between diabetes-associated TCR and between MBP and diabetes antigens reported here may also have implications for the observation that many people with MS suffer from insulin resistance. A logical implication of the results is that the anti-myelin autoimmunity that characterizes MS will bleed over to impair insulin functions, though whether the cross-reactivity is manifested at the level of the pancreas, insulin (and perhaps insulin-like growth factors), or the insulin receptor remains to be investigated and is beyond the scope of the present paper. These possible implications provide additional tests of the cross-reactivity hypothesis presented here.

The indirect role played by anti-insulin autoimmunity in MS would help to explain why insulin resistance can be common in MS without the vast majority of T1D patients developing overt T1D. The specific triggers of T1D, such as coxsackieviruses and enteroviruses more generally, may be lacking in MS patients, leaving only weak cross-reactivity associated with *Clostridia*. On the other hand, the link between MS autoimmunity and cross-reactivity with diabetes-related antigens also provides an explanation for the observed increased risk of T1D in a minority of MS patients. There are likely shared triggers such as *Clostridia* that induce both insulin and MBP-reactive antibodies and TCR. The fact that MS and T1D autoimmune pathologies overlap only partially, however, suggests that in MS, *Clostridia* synergize with other triggers to induce full autoimmune disease, perhaps Epstein–Barr virus, a widely cited putative cause of MS [83,84,85,86]. Only rarely would T1D-specific triggers also be present in MS patients.

### 4.4. Implications for Treating T1D Peripheral Neuropathies

The link between T1D and MS TCR and antibody revealed here may have important clinical implications. One possible implication is that drugs developed to treat the demyelinization that occurs in MS may also provide relief for the symptoms and causes of T1D neuropathies. Some studies have already pointed to such a possibility. Glatiramer acetate, a mixture of synthetic random copolymers composed of alanine, lysine, glutamic acid, and tyrosine with anti-inflammatory and immunomodulatory activities that have proven effective in treating some MS patients, has also been found to ameliorate insulitis, reduce the symptoms of diabetes, and increase the beneficial CD4 + CD25 + Foxp3+ T cell response in a mouse model of T1D [87] as well as improving wound healing in diabetic rats [88]. More importantly, glatiramer acetate also proved to have neuroprotective effects on diabetic patients following treatment for diabetic retinopathy [89]. It may be worth investigating whether these beneficial effects extend to preventing or ameliorating diabetic peripheral neuropathies as well.

Other MS treatments have also proven effective for autoimmune encephalitis and poor blood glucose control in T1D, especially monoclonal antibodies against CD20 such as Ofatumumab and Ocrelizumab [90,91], but they have apparently not yet been tested specifically for preventing or controlling diabetic peripheral neuropathies or retinopathies.

### 4.5. Implications for Treating MS-Associated Insulin Resistance

It follows logically from the T1D–MS shared antigen hypothesis that the potential for using MS treatments for T1D peripheral neuropathies is mirrored by the potential for using diabetes treatments to ameliorate insulin resistance in MS patients. Perhaps the most exciting of these treatments are the new GLP-1 agonists, which not only help to lower blood sugar but also exert significant anti-inflammatory and neuroprotective effects. According to Kay, et al. [92], “*In animal models, GLP-1 agonists have been shown to significantly delay the onset and severity of experimental autoimmune encephalopathy symptoms, as well as to increase nerve myelination and brain weight. In further experiments using animal models of nerve crush injury, specimens given GLP-1 agonists reported a significant increase in the rate and density of nerve regeneration compared to controls. Thus, GLP-1 agonists show promise as both prophylactic and symptomatic treatment for MS and may provide further utility in the treatment of other autoimmune, inflammatory, and neurodegenerative conditions*.” A review by Sango, et al. [93] also cites studies demonstrating improved axonal regeneration and remyelinization following nerve lesions in animal models. GLP-1 agonists have proven to be safe in studies of obese MS patients but the effects on insulin resistance and demyelinization have not yet been carried out [94].

### 4.6. In Sum

In conclusion, the Introduction provided a review of studies suggesting that some connection exists between the immunology of T1D and MS particularly as regards both demyelinization associated with diabetic neuropathies and the insulin resistance associated with some cases of MS. The pilot study results reported here suggest cross-reactivity between diabetic antibodies and myelin basic protein, providing new insights into the possible pathogenesis of diabetic neuropathies and the insulin resistance experienced by many multiple sclerosis patients. While all the experiments reported here are preliminary, they all consistently point to the existence of shared antigens that are cross-reactive in T1D and MS and which may be able to provoke cross-reactive antibodies and T cells. These data, in turn, are consistent with the literature summarized in the Introduction. However, like many serendipitous discoveries, this one raises more questions and possibilities than it answers so that many additional experiments and clinical studies will be clearly needed to validate or invalidate the hypothesis of shared immunologic reactivities. Beyond possibly explaining some of the clinical symptoms shared by T1D and MS, the proposed hypothesis also reveals novel therapeutic possibilities so that treatments found effective in one of these diseases may benefit patients suffering from the other as well—a therapeutic area that has only just begun to be explored.

## Figures and Tables

**Table 1:** Comparison of the number and percent of BLAST-derived matches between multiple sclerosis (MS) T cell receptor sequences (TCR) and TCR derived from type 1 diabetes mellitus (T1D), autoimmune myocarditis (AM) patients, and healthy controls. The matches (see Figure 1, Supplementary Materials S1–S3) were evaluated for the quality of the matches in terms of the number of identities with in a 10 amino acid stretch. If all of the amino acids in a stretch of 10 amino acids were identical, the score was 10 of 10; if nine amino acids matched within a stretch of 10 amino acids, the score was 9 of 10; etc. T1D TCR displayed a significantly higher percentage of higher quality matches than did either Healthy TCR (Chi square = 498.098 with 4 degrees of freedom, *p* < 0.0001 or *p* < 0.00033 after Bonferroni correction) or AM TCR (Chi squared = 1273.945 with 4 degrees of freedom, *p* < 0.0001 or *p* < 0.00033 after Bonferroni correction). AM TCR were also significantly different from Healthy TCR (403.093 with 3 degrees of freedom *p* < 0.0001 or *p* < 0.001 after Bonferroni correction). See Figure 1 and Supplementary Materials S1–S3 for examples.

MS TCR Matches	10 of 10	9 of 10	8 of 10	7 of 10
T1D TCR (n = 73)	1 (1.3%)	11 (15.1%)	30 (41.1%)	27 (36.9%)
HEALTHY TCR (n = 150)	0	2 (1.3%)	33 (22.2%)	99 (66.6%)
AM TCR (n = 34)	0	0	7 (20.0%)	18 (51.4%)

**Table 2:** Binding constants derived from the ultraviolet studies of diabetic T cell receptor sequences binding to human myelin basic protein (Figure 2). These binding constants are compared with those derived in a previous reference [40] to other diabetes related proteins: human insulin; human glucagon; and two peptides derived from the human insulin receptor (Ins Rec) alpha and beta chains. The TCR numbers refer to the sequences listed in Figure 1 and Supplementary Material S1. TCR DIA 4,8,9 is a consensus sequence derived from TCR DIA 4, TCR DIA 8, and TCR DIA 9. Note that the larger the binding constant (i.e., the greater the concentration of the varied molecule needed to half-saturate binding to the molecule held constant), the lower the affinity. Numbers greater than 1000 μM indicate that no binding was observable under the concentration conditions utilized in these experiments and therefore represent negative controls. Binding constants are approximate and valid within about 5% of the stated values (see Materials and Methods).

Binding Constants (µM)	Human MBP	Insulin	Glucagon	Ins Rec α 105–118	Ins Rec β 897–915
TCR DIA 1	40	80	>1000	125	300
TCR DIA 2	>1000	>1000	>1000	>1000	>1000
TCR DIA 4	60	15	>1000	>1000	>1000
TCR DIA 4,8,9	35	>1000	>1000	>1000	>1000
TCR DIA 8	8	130	140	120	130
TCR DIA 9	8	23	140	150	145
TCR DIA 10	>250	130	90	110	140

**Table 3:** Results of enzyme-linked immunadsorption assays involving guinea pig antibodies against human insulin (huINS), glutamic acid decarboxylase type 1 (GAD1), and Protein Tyrosine Phosphatase Receptor Type N (PTPRN) binding to human myelin basic protein (huMBP), porcine myelin basic protein (pMBP), huINS, human insulin (huGLUC), human PTPRN, and human GAD1. ND = Not Done. Data are from Figure 4 and Figure 5, and additional experiments are not shown in those Figures. Where there were two or more inflection points in the curves in Figure 5, binding constants were calculated for each. Note that the larger the binding constant, the lower the affinity. Numbers greater than 10 indicate that no binding was observable under the concentration conditions utilized in these experiments. This does not mean that the TCR does not bind to the antigen, but it does mean that any binding is very unlikely to have physiological significance as the affinity approaches zero. Binding constants are approximate and valid within about 5% of the stated values (see Materials and Methods).

Kd (μM)	huMBP	pMBP	huINS	huGLUC	PTPRN	GAD1
gpINS Ab	1.2	0.3	0.02	>100	ND	ND
gpGAD Ab	ND	0.0020 & 0.02	>10	>10	>10	0.001
gpPTPRN Ab	ND	0.0023	>10	>10	0.0008	>10

**Table 4:** Summary of binding constants of enzyme-linked immunoadsorption assays involving sera from type 1 diabetes mellitus patients (T1D) or healthy controls binding to human myelin basic protein (hMBP) from Figure 6 as well as additional experiments. Also shown for comparison are data from a previous study [52] using the same sera to measure binding to human insulin and *Clostridium sporogenes* antigens. Kd greater than 1000 for hMBP or greater than 1.0 for insulin and *C. sporogenes* indicates that no binding was observed under the concentration conditions utilized in these experiments. Binding constants are approximate and valid within about 5% of the stated values (see Materials and Methods).

	hMBP	INSULIN	C. Sporogenes
Disease Status	Kd (μM)	Kd (μM)	Kd (mg/mL antigen)
T1D DIA 69	50	>1.0	>1.0
T1D DIA 70	>1000	>1.0	>1.0
T1D DIA 71	4.5	>1.0	>1.0
T1D DIA 01	0.5 & 6	0.01	0.05
T1D DIA 02	2 & 60	0.04	0.04
T1D DIA 03	3	0.05	>1.0
T1D DIA 04	0.6 & 20	0.01	0.1
T1D DIA 05	9	0.08	>1.0
Healthy 1	>1000	>1.0	0.7
Healthy 86	>1000	>1.0	>1.0
Healthy 87	>1000	>1.0	>1.0
Healthy 88	>1000	>1.0	>1.0
Healthy 89	>1000	>1.0	>1.0

## Data Availability

The original contributions presented in this study are included in the article/Supplementary Materials. Further inquiries can be directed to the corresponding author.

## Supplementary Materials

The following supporting information can be downloaded at: https://www.mdpi.com/article/10.3390/cells15151384/s1, S1: BLAST Comparison of Diabetic TCR Similarities to Human Myelin Basic Protein; S2: Complete BLAST Results of Autoimmune Myocarditis TCR Simi-larities to Human Myelin Basic Protein; S3: BLAST Results for 150 Healthy TCR for Matches to Multiple Sclerosis TCR; S4: Full results of BLAST Search Comparing Human Myelin Basic Protein with Clostridium perfringens.
