# Supplementary material for: Type 1 Diabetes-Associated Autoantibodies, Sera and T-Cell Receptor Sequences Recognize Myelin Basic Protein: A Novel Peripheral Diabetic Neuropathy–Multiple Sclerosis Connection
Source: Cells. 2026 Jul 31;15(15):1384. doi: 10.3390/cells15151384 (PMC13465925; doi:10.3390/cells15151384)
Supplement: Supplementary file 1 [file cells-15-01384-s001.zip › cells-4377791-supplementary.pdf]

# S1: BLAST Comparison of Diabetic TCR Similarities to Human Myelin Basic Protein

Seay HR, Yusko E, Rothweiler SJ, Zhang L, Posgai AL, Campbell-Thompson M, Vignali M, Emerson RO, Kaddis JS, Ko D, Nakayama M, Smith MJ, Cambier JC, Pugliese A, Atkinson MA, Robins HS, Brusko TM. Tissue distribution and clonal diversity of the T and B cell repertoire in type 1 diabetes. JCI Insight. 2016 Dec 8;1(20):e88242. doi: 10.1172/jci.insight.88242. PMID: 27942583; PMCID: PMC5135280.

**DIABETES (Kent et al., 2005)**

**TCR diabetic 4: DSALYLCASSLATSGGGSDTQYFGPGTRL**

**TCR diabetic 5: TFCAVVNMDSNYQLIWGAGTKL**

**TCR diabetic 6: YFCADAGGTSYKLFGQGTIL**

**TCR diabetic 7: YYCIPGSEEYGNKLVFGAGTIL**

**TCR diabetic 8: DSALYLCASSQVRLAGGGEQFFGPGTRL**

**TCR diabetic 9: DSALYLCASSLGQGETEAFFGQGTRL**

**TCR diabetic 10: DSGVYCASRNLGLNTEAFFGQGARL**

**TCR diabetic 11: HTSMYLCASSFRRBTDTQYFGPGTRL**

**DIABETES (Durinovic-Bello et al., 1994)**

**TCR DIA K2.12 CASSSDRLGNQPQHF**

**TCR DIA K2.16 CASSQDRLRGVADTQYF**

**TCR DIA K2.4 CASSQEADIQYF**

## A1: CASRDRGGGEKLFF

myelin basic protein specific T-cell receptor V beta-D beta-J beta, MBP reactive TCR VDJ beta {clone SE(5), rearranged CDR3 region} [human, inflammatory brain lesions, HLA phenotype 1, Peptide Partial, 26 aa] [Homo sapiens]

Sequence ID: AAB25639.1Length: 26Number of Matches: 1

Range 1: 2 to 17GenPeptGraphics Next Match Previous Match

Alignment statistics for match #1

| Score | Expect | Identities | Positives | Gaps |
|---|---|---|---|---|
| 23.5 bits(48) | 30 | 11/16(69%) | 11/16(68%) | 2/16(12%) |

```
Query  1    CAS-R-DRGGGEKLFF  14
            CAS R DRG GE   FF
Sbjct  2    CASSRLDRGQGEEQFF  17
```

T-cell receptor beta chain, partial [Homo sapiens]

Sequence ID: CAC03942.1Length: 68Number of Matches: 1

Range 1: 54 to 67GenPeptGraphics Next Match Previous Match

Alignment statistics for match #1

| Score | Expect | Identities | Positives | Gaps |
|---|---|---|---|---|
| 35.4 bits(76) | 0.002 | 12/14(86%) | 12/14(85%) | 0/14(0%) |

```
Query  1    CASRDRGGGEKLFF  14
            CASRD G GEKLFF
Sbjct  54   CASRDTGMGEKLFF  67
```

Babbe,H., Roers,A., Waisman,A., Lassmann,H., Goebels,N.,
Hohlfeld,R., Friese,M., Schroder,R., Deckert,M., Schmidt,S.,
Ravid,R. and Rajewsky,K.

TITLE Clonal expansions of CD8(+) T cells dominate the T cell infiltrate in active multiple sclerosis lesions as shown by micromanipulation and single cell polymerase chain reaction

JOURNAL J. Exp. Med. 192 (3), 393-404 (2000)

PUBMED 10934227

## A2: CASSAETQYF

T-cell receptor beta chain variable region, partial [Homo sapiens]
Sequence ID: ANO54017.1Length: 37Number of Matches: 1
Range 1: 18 to 28GenPeptGraphics Next Match Previous Match

Alignment statistics for match #1

| Score | Expect | Identities | Positives | Gaps |
|---|---|---|---|---|
| 31.2 bits(66) | 0.030 | 10/11(91%) | 10/11(90%) | 1/11(9%) |

```
Query  1   CASS-AETQYF  10
           CASS AETQYF
Sbjct  18  CASSQAETQYF  28
```

```
Joachims,M.L., Leehan,K.M., Lawrence,C., Pelikan,R.C., Moore,J.S.,
            Pan,Z., Rasmussen,A., Radfar,L., Lewis,D.M., Grundahl,K.M.,
            Kelly,J.A., Wiley,G.B., Shugay,M., Chudakov,D.M., Lessard,C.J.,
            Stone,D.U., Scofield,R.H., Montgomery,C.G., Sivils,K.L.,
            Thompson,L.F. and Farris,A.D.
  TITLE     Single-cell analysis of glandular T cell receptors in Sjogren's
            syndrome
  JOURNAL   JCI Insight 1 (8) (2016)
```

## A3: CASSFRQGEQETQYF

T-cell receptor beta V21-1J2.5, TCRBV21-1J2.5 {complementarity determining region 3} [human, peripheral T-lymphocytes, insulin-dependent diabetes mellitus patient GAD65.05, Peptide Partial, 23 aa] [Homo sapiens]
Sequence ID: AAB46917.1Length: 23Number of Matches: 1
Range 1: 1 to 14GenPeptGraphics Next Match Previous Match

Alignment statistics for match #1

| Score | Expect | Identities | Positives | Gaps |
|---|---|---|---|---|
| 27.8 bits(58) | 0.86 | 10/15(67%) | 11/15(73%) | 1/15(6%) |

```
Query  1   CASSFRQGEQETQYF  15
           CASS+   G +ETQYF
Sbjct  1   CASSW-PGGHETQYF  14
```

T-cell receptor beta chain variable region, partial [Homo sapiens]
Sequence ID: ANO55312.1Length: 32Number of Matches: 1
Range 1: 11 to 23GenPeptGraphics Next Match Previous Match

Alignment statistics for match #1

---

| Score | Expect | Identities | Positives | Gaps |
|---|---|---|---|---|
| 28.2 bits(59) | 0.78 | 11/15(73%) | 11/15(73%) | 2/15(13%) |

```
Query  1   CASSFRQGEQETQYF  15
           CASS R G  ETQYF
Sbjct  11  CASS-RDG-PETQYF  23
```

T-cell receptor beta chain variable region, partial [Homo sapiens]
Sequence ID: ANO55884.1Length: 40Number of Matches: 1
Range 1: 18 to 25GenPeptGraphics Next Match Previous Match
Alignment statistics for match #1

| Score | Expect | Identities | Positives | Gaps |
|---|---|---|---|---|
| 28.2 bits(59) | 0.85 | 8/8(100%) | 8/8(100%) | 0/8(0%) |

```
Query  1   CASSFRQG  8
           CASSFRQG
Sbjct  18  CASSFRQG  25
```

```
Joachims,M.L., Leehan,K.M., Lawrence,C., Pelikan,R.C., Moore,J.S.,
            Pan,Z., Rasmussen,A., Radfar,L., Lewis,D.M., Grundahl,K.M.,
            Kelly,J.A., Wiley,G.B., Shugay,M., Chudakov,D.M., Lessard,C.J.,
            Stone,D.U., Scofield,R.H., Montgomery,C.G., Sivils,K.L.,
            Thompson,L.F. and Farris,A.D.
  TITLE     Single-cell analysis of glandular T cell receptors in Sjogren's
            syndrome
  JOURNAL   JCI Insight 1 (8) (2016
```

myelin basic protein specific T-cell receptor V beta-D beta-J beta, MBP reactive TCR VDJ beta {clone LJ 1(2), rearranged CDR3 region} [human, brain plaques, HLA phenotype 1, Peptide Partial, 22 aa] [Homo sapiens]
Sequence ID: AAB25628.1Length: 22Number of Matches: 1
Range 1: 2 to 13GenPeptGraphics Next Match Previous Match
Alignment statistics for match #1

| Score | Expect | Identities | Positives | Gaps |
|---|---|---|---|---|
| 28.2 bits(59) | 0.25 | 9/12(75%) | 9/12(75%) | 2/12(16%) |

```
Query  1   CASS--AETQYF  10
           CASS   ETQYF
Sbjct  2   CASSDSSETQYF  13
```

myelin basic protein (143-168)-specific TCR beta-chain {clone JA.1.7} [human, multiple sclerosis patient, T cells, Peptide Partial, 27 aa] [Homo sapiens]
Sequence ID: AAB31429.1Length: 27Number of Matches: 1
Range 1: 1 to 14GenPeptGraphics Next Match Previous Match
Alignment statistics for match #1

| Score | Expect | Identities | Positives | Gaps |
|---|---|---|---|---|
| 27.8 bits(58) | 1.00 | 10/15(67%) | 11/15(73%) | 1/15(6%) |

```
Query  1    CASSFRQGEQETQYF  15
            CASS   +G +ETQYF
Sbjct  1    CASSL-DGGEETQYF  14
```

myelin basic protein specific T-cell receptor V beta-D beta-J beta, MBP reactive TCR VDJ beta {CDR3 region, clone JO(5)} [human, normal peripheral blood lymphocytes, Peptide Partial, 25 aa] [Homo sapiens]
Wucherpfennig,K.W., Zhang,J., Witek,C., Matsui,M., Modabber,Y.,
Ota,K. and Hafler,D.A.
TITLE Clonal expansion and persistence of human T cells specific for an immunodominant myelin basic protein peptide
JOURNAL J. Immunol. 152 (11), 5581-5592 (1994)
PUBMED 7514641

Sequence ID: AAB25660.1Length: 25Number of Matches: 1
Range 1: 2 to 16GenPeptGraphics Next Match Previous Match

Alignment statistics for match #1

| Score | Expect | Identities | Positives | Gaps |
|---|---|---|---|---|
| 27.4 bits(57) | 1.3 | 11/16(69%) | 11/16(68%) | 2/16(12%) |

```
Query  1    CASSFRQGE-QETQYF  15
            CASS R G+ +ETQYF
Sbjct  2    CASS-RPGNIRETQYF  16
```

```
Oksenberg,J.R., Panzara,M.A., Begovich,A.B., Mitchell,D.,
            Erlich,H.A., Murray,R.S., Shimonkevitz,R., Sherritt,M.,
            Rothbard,J., Bernard,C.C. et al.
  TITLE     Selection for T-cell receptor V beta-D beta-J beta gene            rear-
rangements with specificity for a myelin basic protein peptide             in brain le-
sions of multiple sclerosis
  JOURNAL   Nature 362 (6415), 68-70 (1993)
```

## A4: CASSISNTGELFF

T-cell receptor beta V20-3J2.2, TCRBV20-3J2.2 {complementarity determining region 3} [human, peripheral T-lymphocytes, insulin-dependent diabetes mellitus patient GAD65.05, Peptide Partial, 24 aa] [Homo sapiens]
Sequence ID: AAB46916.1Length: 24Number of Matches: 1
Range 1: 1 to 15GenPeptGraphics Next Match Previous Match

Alignment statistics for match #1

| Score | Expect | Identities | Positives | Gaps |
|---|---|---|---|---|
| 31.2 bits(66) | 0.034 | 11/15(73%) | 11/15(73%) | 2/15(13%) |

```
Query  1    CASSIS--NTGELFF  13
            CA SI \ NTGELFF
Sbjct  1    CAWSIGSRNTGELFF  15
```

T-cell receptor beta V21-2J2.7, TCRBV21-2J2.7 {complementarity determining region 3} [human, peripheral T-lymphocytes, insulin-dependent diabetes mellitus patient GAD65.05, Peptide Partial, 27 aa] [Homo sapiens]
Sequence ID: AAB46919.1Length: 27Number of Matches: 1
Range 1: 1 to 10GenPeptGraphics Next Match Previous Match

Alignment statistics for match #1

| Score | Expect | Identities | Positives | Gaps |
|---|---|---|---|---|
| 20.2 bits(40) | 450 | 7/10(70%) | 7/10(70%) | 0/10(0%) |

```
Query  1   CASSISNTGE  10
           CASS S++GE
Sbjct  1   CASSQSHSGE  10
```

myelin basic protein specific T-cell receptor V beta-D beta-J beta, MBP reactive TCR VDJ beta {clone SE(8), rearranged CDR3 region} [human, inflammatory brain lesions, HLA phenotype 1, Peptide Partial, 24 aa] [Homo sapiens]
Sequence ID: AAB25642.1Length: 24Number of Matches: 1
Range 1: 2 to 15GenPeptGraphics Next Match Previous Match

Alignment statistics for match #1

| Score | Expect | Identities | Positives | Gaps |
|---|---|---|---|---|
| 26.1 bits(54) | 2.9 | 10/14(71%) | 10/14(71%) | 1/14(7%) |

```
Query  1    CASSIS-NTGELFF  13
            CASS+S + GELFF
Sbjct  2    CASSTSGQPGELFF  15
```

# A5: CASSLLAGDNEQFF

T-cell receptor beta chain variable region, partial [Homo sapiens]
Sequence ID: ANO55149.1Length: 48Number of Matches: 1
Range 1: 24 to 39GenPeptGraphics Next Match Previous Match

Alignment statistics for match #1

| Score | Expect | Method | Identities | Positives | Gaps |
|---|---|---|---|---|---|
| 34.1 bits(73) | 0.006 | Composition-based stats. | 13/16(81%) | 14/16(87%) | 2/16(12%) |

```
Query  1    CASSL-LAG-DNEQFF  14
            CASSL LAG +NEQFF
Sbjct  24   CASSLSLAGSNNEQFF  39
```

```
Joachims,M.L., Leehan,K.M., Lawrence,C., Pelikan,R.C., Moore,J.S.,
            Pan,Z., Rasmussen,A., Radfar,L., Lewis,D.M., Grundahl,K.M.,
            Kelly,J.A., Wiley,G.B., Shugay,M., Chudakov,D.M., Lessard,C.J.,
            Stone,D.U., Scofield,R.H., Montgomery,C.G., Sivils,K.L.,
            Thompson,L.F. and Farris,A.D.
  TITLE     Single-cell analysis of glandular T cell receptors in Sjogren's
            syndrome
  JOURNAL   JCI Insight 1 (8) (2016
```

myelin basic protein specific T-cell receptor V beta-D beta-J beta, MBP reactive TCR VDJ beta {clone KL-3(8), rearranged CDR3 region} [human, brain plaques, HLA phenotype 1, Peptide Partial, 25 aa] [Homo sapiens]

Sequence ID: AAB25611.1Length: 25Number of Matches: 1

Range 1: 2 to 16GenPeptGraphics Next Match Previous Match

Alignment statistics for match #1

| Score | Expect | Identities | Positives | Gaps |
|---|---|---|---|---|
| 35.0 bits(75) | 0.001 | 13/15(87%) | 13/15(86%) | 1/15(6%) |

```
Query  1    CASSL-LAGDNEQFF  14
            CASSL LAG NEQFF
Sbjct  2    CASSLELAGYNEQFF  16
```

myelin basic protein specific T-cell receptor V beta-D beta-J beta, MBP reactive TCR VDJ beta {clone SE(10), rearranged CDR3 region} [human, inflammatory brain lesions, HLA phenotype 1, Peptide Partial, 25 aa] [Homo sapiens]

Sequence ID: AAB25644.1Length: 25Number of Matches: 1

Range 1: 2 to 15GenPeptGraphics Next Match Previous Match

Alignment statistics for match #1

| Score | Expect | Identities | Positives | Gaps |
|---|---|---|---|---|
| 29.9 bits(63) | 0.13 | 10/14(71%) | 11/14(78%) | 0/14(0%) |

```
Query  1    CASSLLAGDNEQFF  14
            CASSL+AG N Q+F
Sbjct  2    CASSLVAGKNIQYF  15
```

myelin basic protein (84-102)-specific TCR beta-chain {clone NSJL5.7} [human, multiple sclerosis patient, T cells, Peptide Partial, 28 aa] [Homo sapiens]

Sequence ID: AAB31431.1Length: 28Number of Matches: 1

Range 1: 1 to 15GenPeptGraphics Next Match Previous Match

Alignment statistics for match #1

| Score | Expect | Identities | Positives | Gaps |
|---|---|---|---|---|
| 28.6 bits(60) | 0.43 | 11/15(73%) | 11/15(73%) | 1/15(6%) |

```
Query  1    CASSLLAGD-NEQFF  14
            CASSL +G+ NEQFF
Sbjct  1    CASSLTVGSYNEQFF  15
```

## A6: CASSLPGGAGNEQFF

T-cell receptor beta V21-1J2.5, TCRBV21-1J2.5 {complementarity determining region 3} [human, peripheral T-lymphocytes, insulin-dependent diabetes mellitus patient GAD65.05, Peptide Partial, 23 aa] [Homo sapiens]
Sequence ID: AAB46917.1Length: 23Number of Matches: 1
Range 1: 1 to 14GenPeptGraphics Next Match Previous Match

Alignment statistics for match #1

| Score | Expect | Method | Identities | Positives | Gaps |
|---|---|---|---|---|---|
| 20.2 bits(40) | 594 | Composition-based stats. | 10/16(63%) | 11/16(68%) | 3/16(18%) |

```
Query  1    CASSLPGGAGNE-QFF  15
            CASS PG  G+E Q+F
Sbjct  1    CASSWPG--GHETQYF  14
```

T-cell receptor beta V6-2J1.4, TCRBV6-2J1.4 {complementarity determining region 3} [human, peripheral T-lymphocytes, insulin-dependent diabetes mellitus patient GAD65.05, Peptide Partial, 25 aa] [Homo sapiens]
Sequence ID: AAB46915.1Length: 25Number of Matches: 1
Range 1: 1 to 16GenPeptGraphics Next Match Previous Match

Alignment statistics for match #1

| Score | Expect | Method | Identities | Positives | Gaps |
|---|---|---|---|---|---|
| 18.9 bits(37) | 1432 | Composition-based stats. | 11/16(69%) | 12/16(75%) | 1/16(6%) |

```
Query  1    CASSLPGGA-GNEQFF  15
            CASS PG A G+E FF
Sbjct  1    CASSPPGRADGDELFF  16
```

myelin basic protein (84-102)-specific TCR beta-chain {clone NSJL5.7} [human, multiple sclerosis patient, T cells, Peptide Partial, 28 aa] [Homo sapiens]
Sequence ID: AAB31431.1Length: 28Number of Matches: 1
Range 1: 1 to 15GenPeptGraphics Next Match Previous Match

Alignment statistics for match #1

| Score | Expect | Identities | Positives | Gaps |
|---|---|---|---|---|
| 28.6 bits(60) | 0.50 | 11/15(73%) | 11/15(73%) | 0/15(0%) |

```
Query  1    CASSLPGGAGNEQFF  15
            CASSL  G  NEQFF
```

---

```
Sbjct  1   CASSLTVGSYNEQFF  15
```

## A7: CASSPPGLGEQYF

T-cell receptor beta V6-2J1.4, TCRBV6-2J1.4 {complementarity determining region 3} [human, peripheral T-lymphocytes, insulin-dependent diabetes mellitus patient GAD65.05, Peptide Partial, 25 aa] [Homo sapiens]
Sequence ID: AAB46915.1Length: 25Number of Matches: 1
Range 1: 1 to 7GenPeptGraphics Next Match Previous Match

Alignment statistics for match #1

| Score | Expect | Identities | Positives | Gaps |
|---|---|---|---|---|
| 24.4 bits(50) | 13 | 7/7(100%) | 7/7(100%) | 0/7(0%) |

```
Query  1  CASSPPG  7
          CASSPPG
Sbjct  1  CASSPPG  7
```

T-cell receptor beta chain variable region, partial [Homo sapiens]
Sequence ID: ANO54361.1Length: 89Number of Matches: 1
Range 1: 68 to 80GenPeptGraphics Next Match Previous Match

Alignment statistics for match #1

| Score | Expect | Identities | Positives | Gaps |
|---|---|---|---|---|
| 33.3 bits(71) | 0.010 | 11/13(85%) | 11/13(84%) | 0/13(0%) |

```
Query  1   CASSPPGLGEQYF  13
           CASSP GLG QYF
Sbjct  68  CASSPGGLGIQYF  80
```

```
Joachims,M.L., Leehan,K.M., Lawrence,C., Pelikan,R.C., Moore,J.S.,
            Pan,Z., Rasmussen,A., Radfar,L., Lewis,D.M., Grundahl,K.M.,
            Kelly,J.A., Wiley,G.B., Shugay,M., Chudakov,D.M., Lessard,C.J.,
            Stone,D.U., Scofield,R.H., Montgomery,C.G., Sivils,K.L.,
            Thompson,L.F. and Farris,A.D.
  TITLE     Single-cell analysis of glandular T cell receptors in Sjogren's
            syndrome
  JOURNAL   JCI Insight 1 (8) (2016)
```

myelin basic protein (84-102)-specific TCR beta-chain {clone NB17.8} [human, multiple sclerosis patient, T cells, Peptide Partial, 27 aa] [Homo sapiens]
Sequence ID: AAB31433.1Length: 27Number of Matches: 1
Range 1: 1 to 14GenPeptGraphics Next Match Previous Match

Alignment statistics for match #1

| Score | Expect | Identities | Positives | Gaps |
|---|---|---|---|---|
| 21.8 bits(44) | 110 | 10/16(63%) | 11/16(68%) | 5/16(31%) |

```
Query  1    CAS--SPPGLG-EQYF  13
            CAS  SP  LG EQ+F
Sbjct  1    CASSYSP--LGNEQFF  14
```

myelin basic protein specific T-cell receptor V beta-D beta-J beta, MBP reactive TCR VDJ beta {clone KL-3(7), rearranged CDR3 region} [human, brain plaques, HLA phenotype 1, Peptide Partial, 24 aa] [Homo sapiens]
Sequence ID: AAB25610.1Length: 24Number of Matches: 1
Range 1: 2 to 15GenPeptGraphics Next Match Previous Match

Alignment statistics for match #1

| Score | Expect | Identities | Positives | Gaps |
|---|---|---|---|---|
| 21.4 bits(43) | 151 | 8/14(57%) | 9/14(64%) | 1/14(7%) |

```
Query  1    CASSPPGL-GEQYF  13
            CASS P++  EQ+F
Sbjct  2    CASSQPAVYNEQFF  15
```

myelin basic protein specific T-cell receptor V beta-D beta-J beta, MBP reactive TCR VDJ beta {CDR3 region, clone JO(5)} [human, normal peripheral blood lymphocytes, Peptide Partial, 25 aa] [Homo sapiens]
Sequence ID: AAB25660.1Length: 25Number of Matches: 1
Range 1: 2 to 16GenPeptGraphics Next Match Previous Match

Alignment statistics for match #1

| Score | Expect | Identities | Positives | Gaps |
|---|---|---|---|---|
| 21.4 bits(43) | 153 | 10/15(67%) | 10/15(66%) | 2/15(13%) |

```
Query  1    CASSPPG-LGE-QYF  13
            CASS PG + E QYF
Sbjct  2    CASSRPGNIRETQYF  16
```

## A8: CASSPRDGSSYNEQFF

T cell receptor beta chain, partial [Homo sapiens]
Sequence ID: AAG15826.1Length: 35Number of Matches: 1
Range 1: 10 to 26GenPeptGraphics Next Match Previous Match

Alignment statistics for match #1

| Score | Expect | Identities | Positives | Gaps |
|---|---|---|---|---|
| 42.6 bits(93) | 4e-06 | 15/18(83%) | 15/18(83%) | 3/18(16%) |

```
Query  1    CASSP--RDGSSYNEQFF  16
            CASSP  R GSSYNEQFF
```

---

```
Sbjct  10  CASSPHSR-GSSYNEQFF  26
```

```
Slachta,C.A., Jeevanandam,V., Goldman,B., Lin,W.L. and
            Platsoucas,C.D.
  TITLE     Coronary arteries from human cardiac allografts with chronic
            rejection contain oligoclonal T cells: persistence of identical
            clonally expanded TCR transcripts from the early
            post-transplantation period (endomyocardial biopsies) to chronic
            rejection (coronary arteries)
  JOURNAL   J. Immunol. 165 (6), 3469-3483 (2000)
```

T-cell receptor beta chain variable region, partial [Homo sapiens]
Sequence ID: ANO56240.1Length: 42Number of Matches: 1
Range 1: 18 to 33GenPeptGraphics Next Match Previous Match

Alignment statistics for match #1

| Score | Expect | Identities | Positives | Gaps |
|---|---|---|---|---|
| 41.4 bits(90) | 2e-05 | 13/16(81%) | 13/16(81%) | 0/16(0%) |

```
Query  1   CASSPRDGSSYNEQFF  16
           CASS R G SYNEQFF
Sbjct  18  CASSLRGGTSYNEQFF  33
```

```
Joachims,M.L., Leehan,K.M., Lawrence,C., Pelikan,R.C., Moore,J.S.,
            Pan,Z., Rasmussen,A., Radfar,L., Lewis,D.M., Grundahl,K.M.,
            Kelly,J.A., Wiley,G.B., Shugay,M., Chudakov,D.M., Lessard,C.J.,
            Stone,D.U., Scofield,R.H., Montgomery,C.G., Sivils,K.L.,
            Thompson,L.F. and Farris,A.D.
  TITLE     Single-cell analysis of glandular T cell receptors in Sjogren's
            syndrome
  JOURNAL   JCI Insight 1 (8) (2016)
```

myelin basic protein-specific T-cell receptor beta chain VDJ region {clone FN2.2} [human, peripheral blood mononuclear cells, Peptide Partial, 25 aa] [Homo sapiens]
Sequence ID: AAB33027.1Length: 25Number of Matches: 1
Range 1: 1 to 16GenPeptGraphics Next Match Previous Match

Alignment statistics for match #1

| Score | Expect | Identities | Positives | Gaps |
|---|---|---|---|---|
| 34.6 bits(74) | 0.003 | 11/16(69%) | 11/16(68%) | 0/16(0%) |

```
Query  1   CASSPRDGS-SYNEQFF  16
           CASS R GS +YNEQFF
Sbjct  1   CASSLR-GSGTYNEQFF  16
```

myelin basic protein (84-102)-specific TCR beta-chain {clone NSJL5.7} [human, multiple sclerosis patient, T cells, Peptide Partial, 28 aa] [Homo sapiens]
Sequence ID: AAB31431.1Length: 28Number of Matches: 1
Range 1: 1 to 15GenPeptGraphics Next Match Previous Match

Alignment statistics for match #1

| Score | Expect | Identities | Positives | Gaps |
|---|---|---|---|---|
| 29.1 bits(61) | 0.40 | 12/16(75%) | 12/16(75%) | 1/16(6%) |

```
Query  1   CASSPRDGSSYNEQFF  16
           CASS   GS YNEQFF
Sbjct  1   CASSLTVGS-YNEQFF  15
```

## A9: CASSYGTVNSPLHF

T cell receptor beta chain, partial [Homo sapiens]
Sequence ID: ABO26911.1Length: 43Number of Matches: 1
Range 1: 19 to 32GenPeptGraphics Next Match Previous Match

Alignment statistics for match #1

| Score | Expect | Identities | Positives | Gaps |
|---|---|---|---|---|
| 34.1 bits(73) | 0.005 | 11/14(79%) | 12/14(85%) | 0/14(0%) |

```
Query  1   CASSYGTVNSPLHF  14
           CASS+ T NSPLHF
Sbjct  19  CASSFDTNNSPLHF  32
```

```
Wu,H.D., Maurer,M.S., Friedman,R.A., Marboe,C.C.,
            Ruiz-Vazquez,E.M., Ramakrishnan,R., Schwartz,A., Tilson,M.D.,
            Stewart,A.S. and Winchester,R.
  TITLE     The lymphocytic infiltration in calcific aortic stenosis
            predominantly consists of clonally expanded T cells
  JOURNAL   J. Immunol. 178 (8), 5329-5339 (2007)
```

T-cell receptor beta chain variable region, partial [Homo sapiens]
Sequence ID: ANO54677.1Length: 45Number of Matches: 1
Range 1: 22 to 36GenPeptGraphics Next Match Previous Match

Alignment statistics for match #1

| Score | Expect | Identities | Positives | Gaps |
|---|---|---|---|---|
| 32.9 bits(70) | 0.016 | 12/15(80%) | 12/15(80%) | 1/15(6%) |

```
Query  1   CASSY-GTVNSPLHF  14
           CASS  GT NSPLHF
Sbjct  22  CASSITGTANSPLHF  36
```

---

```
Joachims,M.L., Leehan,K.M., Lawrence,C., Pelikan,R.C., Moore,J.S.,
            Pan,Z., Rasmussen,A., Radfar,L., Lewis,D.M., Grundahl,K.M.,
            Kelly,J.A., Wiley,G.B., Shugay,M., Chudakov,D.M., Lessard,C.J.,
            Stone,D.U., Scofield,R.H., Montgomery,C.G., Sivils,K.L.,
            Thompson,L.F. and Farris,A.D.
  TITLE     Single-cell analysis of glandular T cell receptors in Sjogren's
            syndrome
  JOURNAL   JCI Insight 1 (8) (2016)
```

myelin basic protein specific T-cell receptor V beta-D beta-J beta, MBP reactive TCR VDJ beta {CDR3 region, clone BM(2)} [human, peripheral blood lymphocytes, HLA phenotype 1, Peptide Partial, 25 aa] [Homo sapiens]

Sequence ID: AAB25663.1Length: 25Number of Matches: 1

Range 1: 2 to 16GenPeptGraphics Next Match Previous Match

Alignment statistics for match #1

| Score | Expect | Identities | Positives | Gaps |
|---|---|---|---|---|
| 33.3 bits(71) | 0.007 | 12/15(80%) | 12/15(80%) | 1/15(6%) |

```
Query  1   CASSYGT-VNSPLHF  14
           CASSY T +NSPLHF
Sbjct  2   CASSYRTQLNSPLHF  16
```

myelin basic protein specific T-cell receptor V beta-D beta-J beta, MBP reactive TCR VDJ beta {clone SE(12), rearranged CDR3 region} [human, inflammatory brain lesions, HLA phenotype 1, Peptide Partial, 25 aa] [Homo sapiens]

Sequence ID: AAB25646.1Length: 25Number of Matches: 1

Range 1: 2 to 8GenPeptGraphics Next Match Previous Match

Alignment statistics for match #1

| Score | Expect | Identities | Positives | Gaps |
|---|---|---|---|---|
| 24.8 bits(51) | 10 | 7/7(100%) | 7/7(100%) | 0/7(0%) |

```
Query  1  CASSYGT  7
          CASSYGT
Sbjct  2  CASSYGT  8
```

myelin basic protein specific T-cell receptor V beta-D beta-J beta, MBP reactive TCR VDJ beta {clone KL-1(3), rearranged CDR3 region} [human, brain plaques, HLA phenotype 1, Peptide Partial, 24 aa] [Homo sapiens]

Sequence ID: AAB25594.1Length: 24Number of Matches: 1

Range 1: 2 to 15GenPeptGraphics Next Match Previous Match

Alignment statistics for match #1

| Score | Expect | Identities | Positives | Gaps |
|---|---|---|---|---|

| | | | | |
|---|---|---|---|---|
| 24.4 bits(50) | 14 | 10/14(71%) | 10/14(71%) | 0/14(0%) |

```
Query  1    CASSYGTVNSPLHF  14
            CASS    +NSPLHF
Sbjct  2    CASSLRLANSPLHF  15
```

myelin basic protein specific T-cell receptor V beta-D beta-J beta, MBP reactive TCR VDJ beta {clone KL-1(5), rearranged CDR3 region} [human, brain plaques, HLA phenotype 1, Peptide Partial, 24 aa] [Homo sapiens]
Sequence ID: AAB25596.1Length: 24Number of Matches: 1
Range 1: 10 to 15GenPeptGraphics Next Match Previous Match

Alignment statistics for match #1

| Score | Expect | Identities | Positives | Gaps |
|---|---|---|---|---|
| 23.1 bits(47) | 42 | 6/6(100%) | 6/6(100%) | 0/6(0%) |

```
Query  9    NSPLHF  14
            NSPLHF
Sbjct  10   NSPLHF  15
```

TCR V beta {V-J junction, clone MS7-1B10} [human, multiple sclerosis patient, myelin basic protein-reactive T cells after vaccination, Peptide Partial, 29 aa] [Homo sapiens]
Sequence ID: AAB35720.1Length: 29Number of Matches: 1
Range 1: 3 to 15GenPeptGraphics Next Match Previous Match

Alignment statistics for match #1

| Score | Expect | Identities | Positives | Gaps |
|---|---|---|---|---|
| 19.7 bits(39) | 746 | 9/15(60%) | 9/15(60%) | 3/15(20%) |

```
Query  1    CASS-YGTVNSPLHF  14
            CASS Y + N+P HF
Sbjct  3    CASSLY-S-NQPQHF  15
```

## A10: CATGLAANTGELFF

T cell receptor beta chain, partial [Homo sapiens]
Sequence ID: AEK75986.1Length: 87Number of Matches: 1
Range 1: 22 to 35GenPeptGraphics Next Match Previous Match

Alignment statistics for match #1

| Score | Expect | Identities | Positives | Gaps |
|---|---|---|---|---|

---

33.7 bits(72) 0.008 10/14(71%) 10/14(71%) 0/14(0%)

```
Query  1   CATGLAANTGELFF  14
           CA+ L +NTGELFF
Sbjct  22  CASSLETNTGELFF  35
```

T cell receptor beta chain, partial [Homo sapiens]
Sequence ID: AEK76062.1Length: 71Number of Matches: 1
Range 1: 38 to 52GenPeptGraphics Next Match Previous Match

Alignment statistics for match #1

| Score | Expect | Identities | Positives | Gaps |
|---|---|---|---|---|
| 32.5 bits(69) | 0.023 | 11/15(73%) | 11/15(73%) | 1/15(6%) |

```
Query  1   CATGLAA-NTGELFF  14
           CA+ L+A NTGELFF
Sbjct  38  CASSLVAVNTGELFF  52
```

```
Winchester,R., Wiesendanger,M., O'Brien,W., Zhang,H.Z.,
            Maurer,M.S., Gillam,L.D., Schwartz,A., Marboe,C. and Stewart,A.S.
  TITLE     Circulating activated and effector memory T cells are associated
            with calcification and clonal expansions in bicuspid and ricuspid
            valves of calcific aortic stenosis
  JOURNAL   J. Immunol. 187 (2), 1006-1014 (2011)
```

myelin basic protein-specific T-cell receptor beta chain VDJ region {clone NN2.23} [human, peripheral blood mononuclear cells, Peptide Partial, 25 aa] [Homo sapiens]
Sequence ID: AAB33028.1Length: 25Number of Matches: 1
Range 1: 11 to 16GenPeptGraphics Next Match Previous Match

Alignment statistics for match #1

| Score | Expect | Identities | Positives | Gaps |
|---|---|---|---|---|
| 22.7 bits(46) | 61 | 6/6(100%) | 6/6(100%) | 0/6(0%) |

```
Query  9   TGELFF  14
           TGELFF
Sbjct  11  TGELFF  16
```

## A11: CSVNRGGTDTQYF

T cell receptor beta chain, partial [Homo sapiens]
Sequence ID: AAG15662.1Length: 28Number of Matches: 1

---

Range 1: 7 to 20GenPeptGraphics Next Match Previous Match

Alignment statistics for match #1

| Score | Expect | Identities | Positives | Gaps |
|---|---|---|---|---|
| 33.3 bits(71) | 0.007 | 11/14(79%) | 11/14(78%) | 1/14(7%) |

```
Query  1   CSVN-RGGTDTQYF  13
           CSV+ R GTDTQYF
Sbjct  7   CSVEFRDGTDTQYF  20
```

```
Slachta,C.A., Jeevanandam,V., Goldman,B., Lin,W.L. and
            Platsoucas,C.D.
  TITLE     Coronary arteries from human cardiac allografts with chronic
            rejection contain oligoclonal T cells: persistence of identical
            clonally expanded TCR transcripts from the early
            post-transplantation period (endomyocardial biopsies) to chronic
            rejection (coronary arteries)
  JOURNAL   J. Immunol. 165 (6), 3469-3483 (2000)
```

T-cell receptor beta chain variable region, partial [Homo sapiens]
Sequence ID: ANO55141.1Length: 61Number of Matches: 1
Range 1: 41 to 52GenPeptGraphics Next Match Previous Match

Alignment statistics for match #1

| Score | Expect | Identities | Positives | Gaps |
|---|---|---|---|---|
| 31.6 bits(67) | 0.039 | 9/12(75%) | 10/12(83%) | 0/12(0%) |

```
Query  2   SVNRGGTDTQYF  13
           S++RG+TDTQYF
Sbjct  41  SADRGATDTQYF  52
```

```
Joachims,M.L., Leehan,K.M., Lawrence,C., Pelikan,R.C., Moore,J.S.,
            Pan,Z., Rasmussen,A., Radfar,L., Lewis,D.M., Grundahl,K.M.,
            Kelly,J.A., Wiley,G.B., Shugay,M., Chudakov,D.M., Lessard,C.J.,
            Stone,D.U., Scofield,R.H., Montgomery,C.G., Sivils,K.L.,
            Thompson,L.F. and Farris,A.D.
  TITLE     Single-cell analysis of glandular T cell receptors in Sjogren's
            syndrome
  JOURNAL   JCI Insight 1 (8) (2016)
```

myelin basic protein specific T-cell receptor V beta-D beta-J beta, MBP TCR reactive VDJ beta {CDR3 region, muscle infiltrating lymphocytes(4)} [human, muscle infiltrating lymphocytes, HLA phenotype 1, Peptide Partial, 27 aa] [Homo sapiens]
Sequence ID: AAB25652.1Length: 27Number of Matches: 1

Range 1: 13 to 18GenPeptGraphics Next Match Previous Match

Alignment statistics for match #1

| Score | Expect | Identities | Positives | Gaps |
|---|---|---|---|---|
| 24.0 bits(49) | 19 | 6/6(100%) | 6/6(100%) | 0/6(0%) |

```
Query  8   TDTQYF  13
           TDTQYF
Sbjct  13  TDTQYF  18
```

myelin basic protein specific T-cell receptor V beta-D beta-J beta, MBP reactive TCR VDJ beta {clone KL-3(9), rearranged CDR3 region} [human, brain plaques, HLA phenotype 1, Peptide Partial, 25 aa] [Homo sapiens]

Sequence ID: AAB25612.1Length: 25Number of Matches: 1

Range 1: 7 to 16GenPeptGraphics Next Match Previous Match

Alignment statistics for match #1

| Score | Expect | Identities | Positives | Gaps |
|---|---|---|---|---|
| 21.4 bits(43) | 153 | 7/10(70%) | 7/10(70%) | 2/10(20%) |

```
Query  6   GGT--DTQYF  13
           GG+  DTQYF
Sbjct  7   GGSEEDTQYF  16
```

myelin basic protein specific T-cell receptor V beta-D beta-J beta, MBP reactive TCR VDJ beta {clone KL-3(11), rearranged CDR3 region} [human, brain plaques, HLA phenotype 1, Peptide Partial, 24 aa] [Homo sapiens]

Sequence ID: AAB25614.1Length: 24Number of Matches: 1

Range 1: 7 to 15GenPeptGraphics Next Match Previous Match

Alignment statistics for match #1

| Score | Expect | Identities | Positives | Gaps |
|---|---|---|---|---|
| 19.3 bits(38) | 890 | 6/9(67%) | 7/9(77%) | 1/9(11%) |

```
Query  6   GGT-DTQYF  13
           GG+ +TQYF
Sbjct  7   GGSVETQYF  15
```

myelin basic protein specific T-cell receptor V beta-D beta-J beta, MBP reactive TCR VDJ beta {clone KL-3(10), rearranged CDR3 region} [human, brain plaques, HLA phenotype 1, Peptide Partial, 24 aa] [Homo sapiens]

Sequence ID: AAB25613.1Length: 24Number of Matches: 1

Range 1: 7 to 15GenPeptGraphics Next Match Previous Match

Alignment statistics for match #1

| Score | Expect | Identities | Positives | Gaps |
|---|---|---|---|---|
| 19.3 bits(38) | 890 | 6/9(67%) | 7/9(77%) | 1/9(11%) |

```
Query  6   GGTD-TQYF  13
```

```
           GG++ TQYF
Sbjct  7   GGSEETQYF  15
```

# TCR DIA 1

# CSVEATRADTQYFG

T-cell receptor beta V21-1J2.5, TCRBV21-1J2.5 {complementarity determining region 3} [human, peripheral T-lymphocytes, insulin-dependent diabetes mellitus patient GAD65.05, Peptide Partial, 23 aa] [Homo sapiens]
Sequence ID: AAB46917.1Length: 23Number of Matches: 1
Range 1: 10 to 15GenPeptGraphics Next Match Previous Match

Alignment statistics for match #1

| Score | Expect | Identities | Positives | Gaps |
|---|---|---|---|---|
| 21.0 bits(42) | 245 | 5/6(83%) | 6/6(100%) | 0/6(0%) |

```
Query  9   DTQYFG  14
           +TQYFG
Sbjct  10  ETQYFG  15
```

T-cell receptor beta chain variable region, partial [Homo sapiens]
Sequence ID: ANO56670.1Length: 38Number of Matches: 1
Range 1: 16 to 30GenPeptGraphics Next Match Previous Match

Alignment statistics for match #1

| Score | Expect | Identities | Positives | Gaps |
|---|---|---|---|---|
| 34.6 bits(74) | 0.004 | 11/15(73%) | 11/15(73%) | 1/15(6%) |

```
Query  1   CSVEA-TRADTQYFG  14
           CSVE+ +R+DTQYFG
Sbjct  16  CSVETQSRGDTQYFG  30
```

T-cell receptor beta chain variable region, partial [Homo sapiens]
Sequence ID: ANO54381.1Length: 77Number of Matches: 1
Range 1: 57 to 69GenPeptGraphics Next Match Previous Match

Alignment statistics for match #1

| Score | Expect | Identities | Positives | Gaps |
|---|---|---|---|---|
| 33.3 bits(71) | 0.011 | 11/14(79%) | 11/14(78%) | 1/14(7%) |

```
Query  1   CSVEATRADTQYFG  14
           CSV ++RADTQYFG
Sbjct  57  CSV-VIRADTQYFG  69
```

Joachims,M.L., Leehan,K.M., Lawrence,C., Pelikan,R.C., Moore,J.S.,

```
           Pan,Z., Rasmussen,A., Radfar,L., Lewis,D.M., Grundahl,K.M.,
           Kelly,J.A., Wiley,G.B., Shugay,M., Chudakov,D.M., Lessard,C.J.,
           Stone,D.U., Scofield,R.H., Montgomery,C.G., Sivils,K.L.,
           Thompson,L.F. and Farris,A.D.
  TITLE    Single-cell analysis of glandular T cell receptors in Sjogren's
           syndrome
  JOURNAL  JCI Insight 1 (8) (2016)
```

T cell receptor beta chain, partial [Homo sapiens]
Sequence ID: AEK76018.1Length: 111Number of Matches: 1
Range 1: 86 to 93GenPeptGraphics Next Match Previous Match

Alignment statistics for match #1

| Score | Expect | Identities | Positives | Gaps |
|---|---|---|---|---|
| 29.5 bits(62) | 0.26 | 8/8(100%) | 8/8(100%) | 0/8(0%) |

```
Query  7   RADTQYFG  14
           RADTQYFG
Sbjct  86  RADTQYFG  93
```

```
Winchester,R., Wiesendanger,M., O'Brien,W., Zhang,H.Z.,
           Maurer,M.S., Gillam,L.D., Schwartz,A., Marboe,C. and Stewart,A.S.
  TITLE    Circulating activated and effector memory T cells are associated
           with calcification and clonal expansions in bicuspid and ricuspid
           valves of calcific aortic stenosis
  JOURNAL  J. Immunol. 187 (2), 1006-1014 (2011)
```

## TCR DIA 2   CASSLAVIRTDTQYFG

RecName: Full=Myosin-7B; AltName: Full=Antigen MLAA-21; AltName: Full=Myosin cardiac muscle beta chain; AltName: Full=Myosin heavy chain 7B, cardiac muscle beta isoform; AltName: Full=Slow A MYH14 [Homo sapiens]
Sequence ID: A7E2Y1.4Length: 1983Number of Matches: 3

- See 2 more title(s) See all Identical Proteins(IPG)

Related Information
Gene-associated gene details
AlphaFold Structure-3D structure displays
Identical Proteins-Identical proteins to A7E2Y1.4
Range 1: 468 to 473GenPeptGraphics Next Match Previous Match

Alignment statistics for match #1

| Score | Expect | Identities | Positives | Gaps |
|---|---|---|---|---|

19.3 bits(38) 1362 6/6(100%) 6/6(100%) 0/6(0%)

```
Query  2    AVGALA  7
            AVGALA
Sbjct  468  AVGALA  473
```

Range 2: 392 to 401GenPeptGraphics Next Match Previous Match First Match

Alignment statistics for match #2

| Score | Expect | Identities | Positives | Gaps |
|---|---|---|---|---|
| 15.5 bits(29) | 32254 | 6/10(60%) | 6/10(60%) | 4/10(40%) |

```
Query  1    CA----VGAL  6
            CA    VGAL
Sbjct  392  CACYKIVGAL  401
```

Range 3: 910 to 918GenPeptGraphics Next Match Previous Match First Match

Alignment statistics for match #3

| Score | Expect | Identities | Positives | Gaps |
|---|---|---|---|---|
| 14.6 bits(27) | 64971 | 5/9(56%) | 5/9(55%) | 0/9(0%) |

```
Query  4    GALAGTASK  12
            GALA++ \K
Sbjct  910  GALAAAEAK  918
```

T-cell receptor beta V21-1J2.5, TCRBV21-1J2.5 {complementarity determining region 3} [human, peripheral T-lymphocytes, insulin-dependent diabetes mellitus patient GAD65.05, Peptide Partial, 23 aa] [Homo sapiens]

Sequence ID: AAB46917.1Length: 23Number of Matches: 1

Range 1: 10 to 15GenPeptGraphics Next Match Previous Match

Alignment statistics for match #1

| Score | Expect | Identities | Positives | Gaps |
|---|---|---|---|---|
| 21.0 bits(42) | 317 | 5/6(83%) | 6/6(100%) | 0/6(0%) |

```
Query  11  DTQYFG  16
            +TQYFG
Sbjct  10  ETQYFG  15
```

T-cell receptor beta chain variable region, partial [Homo sapiens]

Sequence ID: ANO54693.1Length: 84Number of Matches: 1

Range 1: 61 to 76GenPeptGraphics Next Match Previous Match

Alignment statistics for match #1

| Score | Expect | Identities | Positives | Gaps |
|---|---|---|---|---|
| 44.8 bits(98) | 1e-06 | 14/16(88%) | 14/16(87%) | 0/16(0%) |

```
Query  1    CASSLAVIRTDTQYFG  16
```

```
            CASSLAV  TDTQYFG
Sbjct  61   CASSLAVTSTDTQYFG  76
```

T-cell receptor beta chain variable region, partial [Homo sapiens]
Sequence ID: ANO56108.1Length: 42Number of Matches: 1
Range 1: 17 to 34GenPeptGraphics Next Match Previous Match

Alignment statistics for match #1

| Score | Expect | Identities | Positives | Gaps |
|---|---|---|---|---|
| 38.0 bits(82) | 3e-04 | 13/18(72%) | 14/18(77%) | 2/18(11%) |

```
Query  1    CASS--LAVIRTDTQYFG  16
            CASS  LA + TDTQYFG
Sbjct  17   CASSRRLAGVKTDTQYFG  34
```

```
Joachims,M.L., Leehan,K.M., Lawrence,C., Pelikan,R.C., Moore,J.S.,
            Pan,Z., Rasmussen,A., Radfar,L., Lewis,D.M., Grundahl,K.M.,
            Kelly,J.A., Wiley,G.B., Shugay,M., Chudakov,D.M., Lessard,C.J.,
            Stone,D.U., Scofield,R.H., Montgomery,C.G., Sivils,K.L.,
            Thompson,L.F. and Farris,A.D.
  TITLE     Single-cell analysis of glandular T cell receptors in Sjogren's
            syndrome
  JOURNAL   JCI Insight 1 (8) (2016)
```

T cell receptor beta chain, partial [Homo sapiens]
Sequence ID: AEK76027.1Length: 83Number of Matches: 1
Range 1: 17 to 32GenPeptGraphics Next Match Previous Match

Alignment statistics for match #1

| Score | Expect | Identities | Positives | Gaps |
|---|---|---|---|---|
| 36.7 bits(79) | 0.001 | 14/17(82%) | 14/17(82%) | 2/17(11%) |

```
Query  1    CASSLAVIR-TDTQYFG  16
            CASSLA  R TDTQYFG
Sbjct  17   CASSLAG-RFTDTQYFG  32
```

```
Winchester,R., Wiesendanger,M., O'Brien,W., Zhang,H.Z.,
            Maurer,M.S., Gillam,L.D., Schwartz,A., Marboe,C. and Stewart,A.S.
  TITLE     Circulating activated and effector memory T cells are associated
            with calcification and clonal expansions in bicuspid and tricuspid valves
of calcific aortic stenosis
  JOURNAL   J. Immunol. 187 (2), 1006-1014 (2011)
```

## TCR DIA 3    CAVGALAGTASKLTG

myosin, heavy polypeptide 7B, cardiac muscle, beta, isoform CRA_c [Homo sapiens]

Sequence ID: EAW76238.1Length: 1961Number of Matches: 4
Related Information
Gene-associated gene details
Range 1: 418 to 423GenPeptGraphics Next Match Previous Match

Alignment statistics for match #1

| Score | Expect | Identities | Positives | Gaps |
|---|---|---|---|---|
| 19.3 bits(38) | 1362 | 6/6(100%) | 6/6(100%) | 0/6(0%) |

```
Query  2    AVGALA  7
            AVGALA
Sbjct  418  AVGALA  423
```

immunoglobulin heavy chain junction region [Homo sapiens]
Sequence ID: MCD62183.1Length: 15Number of Matches: 1
Range 1: 1 to 11GenPeptGraphics Next Match Previous Match

Alignment statistics for match #1

| Score | Expect | Identities | Positives | Gaps |
|---|---|---|---|---|
| 26.5 bits(55) | 1.6 | 9/11(82%) | 9/11(81%) | 0/11(0%) |

```
Query  1    CAVGALAGTAS  11
            CA+GA+AGTAS
Sbjct  1    CAAGAVAGTAS  11
```

```
Vander Heiden,J.A., Stathopoulos,P., Zhou,J.Q., Chen,L.,
            Gilbert,T.J., Bolen,C.R., Barohn,R.J., Dimachkie,M.M.,
            Ciafaloni,E., Broering,T.J., Vigneault,F., Nowak,R.J.,
            Kleinstein,S.H. and O'Connor,K.C.
  TITLE     Dysregulation of B Cell Repertoire Formation in Myasthenia Gravis
            Patients Revealed through Deep Sequencing
  JOURNAL   J. Immunol. 198 (4), 1460-1473 (2017)
```

## TCR diabetic 4: CASSLATSGGGSDTQYFG

Chain E, T-CELL-RECEPTOR, A3.10-beta chain [Homo sapiens]
Sequence ID: 6XC9_ELength: 245Number of Matches: 1

- See 1 more title(s) See all Identical Proteins(IPG)     Chain E, T-CELL-RECEPTOR, A3.10-beta chain [Homo sapiens]
- Sequence ID: 6XC9_ELength: 245Number of Matches: 1
- See 1 more title(s) See all Identical Proteins(IPG)
- Related Information
- Structure-3D structure displays
- Identical Proteins-Identical proteins to 6XC9_E

---

- Range 1: 90 to 107GenPeptGraphics Next Match Previous Match
- Alignment statistics for match #1
- Score Expect Identities Positives Gaps
- 44.8 bits(98) 2e-06 14/18(78%) 14/18(77%) 0/18(0%)
- Query 1 CASSLATSGGGSDTQYFG 18
- CASSL SGG DTQYFG
- Sbjct 90 CASSLSASGGATDTQYFG 107
-
-
-

Related Information

Structure-3D structure displays

Identical Proteins-Identical proteins to 6XC9_E

Range 1: 90 to 107GenPeptGraphics Next Match Previous Match

Alignment statistics for match #1

| Score | Expect | Identities | Positives | Gaps |
|---|---|---|---|---|
| 44.8 bits(98) | 2e-06 | 14/18(78%) | 14/18(77%) | 0/18(0%) |

```
Query  1     CASSLATSGGGSDTQYFG  18
             CASSL   SGG  DTQYFG
Sbjct  90    CASSLSASGGATDTQYFG  107
```


```
Tran,M.T., Faridi,P., Lim,J.J., Ting,Y.T., Onwukwe,G.,
           Bhattacharjee,P., Jones,C.M., Tresoldi,E., Cameron,F.J., La
           Gruta,N.L., Purcell,A.W., Mannering,S.I., Rossjohn,J. and Reid,H.H.
  TITLE    T cell receptor recognition of hybrid insulin peptides bound to
           HLA-DQ8
  JOURNAL  Nat Commun 12 (1), 5110 (2021)
```

T-cell receptor beta chain variable region, partial [Homo sapiens]

Sequence ID: ANO55144.1Length: 50Number of Matches: 1

Range 1: 26 to 42GenPeptGraphics Next Match Previous Match

Alignment statistics for match #1

| Score | Expect | Identities | Positives | Gaps |
|---|---|---|---|---|
| 38.4 bits(83) | 3e-04 | 14/18(78%) | 14/18(77%) | 1/18(5%) |

```
Query  1    CASSLATSGGGSDTQYFG  18
            CASSL   SGG  DTQYFG
Sbjct  26   CASSLV-SGGPTDTQYFG  42
```

```
Joachims,M.L., Leehan,K.M., Lawrence,C., Pelikan,R.C., Moore,J.S.,
           Pan,Z., Rasmussen,A., Radfar,L., Lewis,D.M., Grundahl,K.M.,
           Kelly,J.A., Wiley,G.B., Shugay,M., Chudakov,D.M., Lessard,C.J.,
           Stone,D.U., Scofield,R.H., Montgomery,C.G., Sivils,K.L.,
```

```
            Thompson,L.F. and Farris,A.D.
  TITLE     Single-cell analysis of glandular T cell receptors in Sjogren's
            syndrome
  JOURNAL   JCI Insight 1 (8) (2016)
```

## TCR diabetic 5: CAVVNMDSNYQ

## TCR diabetic 6: CADAGGTSYKLFG

immunoglobulin heavy chain junction region [Homo sapiens]
Sequence ID: MBN4234100.1Length: 18Number of Matches: 1
Range 1: 1 to 18GenPeptGraphics Next Match Previous Match

Alignment statistics for match #1

| Score | Expect | Identities | Positives | Gaps |
|---|---|---|---|---|
| 29.5 bits(62) | 0.11 | 13/18(72%) | 13/18(72%) | 5/18(27%) |

```
Query  1   CA---DAGGTSY-KL-FG  13
           CA   DAGGTSY KL FG
Sbjct  1   CAFMKDAGGTSYGKLTFG  18
```

```
Jiang,R., Hoehn,K.B., Lee,C.S., Pham,M.C., Homer,R.J.,
            Detterbeck,F.C., Aban,I., Jacobson,L., Vincent,A., Nowak,R.J.,
            Kaminski,H.J., Kleinstein,S.H. and O'Connor,K.C.
  TITLE     Thymus-derived B cell clones persist in the circulation after
            thymectomy in myasthenia gravis
  JOURNAL   Proc Natl Acad Sci U S A 117 (48), 30649-30660 (2020)
```

T cell receptor V alpha 3.1=specific for mycobacterial heat shock protein 60-derived peptide 691 {clone 1.4, complementarity-determining region 3} [human, peripheral blood T cells, Peptide Partial, 31 aa] [Homo sapiens]
Sequence ID: AAB35076.1Length: 31Number of Matches: 1

---

- See 1 more title(s) See all Identical Proteins(IPG)

Related Information
Identical Proteins-Identical proteins to AAB35076.1
Range 1: 3 to 19GenPeptGraphics Next Match Previous Match

Alignment statistics for match #1

| Score | Expect | Identities | Positives | Gaps |
|---|---|---|---|---|
| 29.9 bits(63) | 0.13 | 13/17(76%) | 13/17(76%) | 4/17(23%) |

```
Query  1   CAD--AGGTSY-KL-FG  13
           CAD  AGGTSY KL FG
Sbjct  3   CADPNAGGTSYGKLTFG  19
```

T-cell receptor alpha chain variable region, partial [Homo sapiens]
Sequence ID: ANO55750.1Length: 93Number of Matches: 1
Range 1: 74 to 87GenPeptGraphics Next Match Previous Match

Alignment statistics for match #1

| Score | Expect | Identities | Positives | Gaps |
|---|---|---|---|---|
| 27.8 bits(58) | 0.87 | 11/14(79%) | 12/14(85%) | 2/14(14%) |

```
Query  2   ADAGGTSY-KL-FG  13
           A+AGGTSY KL FG
Sbjct  74  ANAGGTSYGKLTFG  87
```

T-cell receptor alpha chain variable region, partial [Homo sapiens]
Sequence ID: ANO55754.1Length: 88Number of Matches: 1
Range 1: 67 to 82GenPeptGraphics Next Match Previous Match

Alignment statistics for match #1

| Score | Expect | Identities | Positives | Gaps |
|---|---|---|---|---|
| 27.8 bits(58) | 0.87 | 12/16(75%) | 13/16(81%) | 3/16(18%) |

```
Query  1   CA-DAGGTSY-KL-FG  13
           CA +AGGTSY KL FG
Sbjct  67  CAPNAGGTSYGKLTFG  82
```

T-cell receptor alpha chain variable region, partial [Homo sapiens]
Sequence ID: ANO54765.1Length: 90Number of Matches: 1
Range 1: 68 to 84GenPeptGraphics Next Match Previous Match

Alignment statistics for match #1

| Score | Expect | Identities | Positives | Gaps |
|---|---|---|---|---|
| 29.9 bits(63) | 0.15 | 13/17(76%) | 13/17(76%) | 4/17(23%) |

```
Query  1   CA--DAGGTSY-KL-FG  13
           CA  DAGGTSY KL FG
Sbjct  68  CAVRDAGGTSYGKLTFG  84
```

---

T-cell receptor alpha chain variable region, partial [Homo sapiens]
Sequence ID: ANO55122.1Length: 76Number of Matches: 1
Range 1: 51 to 67GenPeptGraphics Next Match Previous Match

Alignment statistics for match #1

| Score | Expect | Identities | Positives | Gaps |
|---|---|---|---|---|
| 29.9 bits(63) | 0.15 | 13/17(76%) | 13/17(76%) | 4/17(23%) |

```
Query  1   CA--DAGGTSY-KL-FG  13
           CA  DAGGTSY KL FG
Sbjct  51  CALSDAGGTSYGKLTFG  67
```

```
Joachims,M.L., Leehan,K.M., Lawrence,C., Pelikan,R.C., Moore,J.S.,
            Pan,Z., Rasmussen,A., Radfar,L., Lewis,D.M., Grundahl,K.M.,
            Kelly,J.A., Wiley,G.B., Shugay,M., Chudakov,D.M., Lessard,C.J.,
            Stone,D.U., Scofield,R.H., Montgomery,C.G., Sivils,K.L.,
            Thompson,L.F. and Farris,A.D.
  TITLE     Single-cell analysis of glandular T cell receptors in Sjogren's
            syndrome
  JOURNAL   JCI Insight 1 (8) (2016)
```

## TCR diabetic 7: CIPGSEEYGNKLVFG

human diabetogenic TCR T1D3 in complex with DQ8-p8E9E peptide [Homo sapiens]
Sequence ID: 6DFX_GLength: 207Number of Matches: 1

- See 1 more title(s) See all Identical Proteins(IPG)

Related Information
Structure-3D structure displays
Identical Proteins-Identical proteins to 6DFX_G
Range 1: 98 to 104GenPeptGraphics Next Match Previous Match

Alignment statistics for match #1

| Score | Expect | Identities | Positives | Gaps |
|---|---|---|---|---|
| 17.6 bits(34) | 5474 | 5/7(71%) | 6/7(85%) | 0/7(0%) |

```
Query  9    GNKLVFG  15
            G KL+FG
Sbjct  98   GGKLIFG  104
```

```
Wang,Y., Sosinowski,T., Novikov,A., Crawford,F., White,J., Jin,N.,
            Liu,Z., Zou,J., Neau,D., Davidson,H.W., Nakayama,M., Kwok,W.W.,
```

---

```
            Gapin,L., Marrack,P., Kappler,J.W. and Dai,S.
  TITLE     How C-terminal additions to insulin B-chain fragments create
            superagonists for T cells in mouse and human type 1 diabetes
  JOURNAL   Sci Immunol 4 (34) (2019)
   PUBMED   30952805
```

T cell receptor alpha chain, partial [Homo sapiens]
Sequence ID: ACX42473.1Length: 67Number of Matches: 1
Range 1: 28 to 38GenPeptGraphics Next Match Previous Match

Alignment statistics for match #1

| Score | Expect | Identities | Positives | Gaps |
|---|---|---|---|---|
| 32.9 bits(70) | 0.019 | 10/11(91%) | 10/11(90%) | 0/11(0%) |

```
Query  5   SEEYGNKLVFG  15
           S EYGNKLVFG
Sbjct  28  SGEYGNKLVFG  38
```

```
Dong,L., Li,P., Oenema,T., McClurkan,C.L. and Koelle,D.M.
  TITLE     Public TCR use by herpes simplex virus-2-specific human CD8 CTLs
  JOURNAL   J. Immunol. 184 (6), 3063-3071 (2010)
   PUBMED   20139278
```

T-cell receptor alpha chain variable region, partial [Homo sapiens]
Sequence ID: ANO54959.1Length: 42Number of Matches: 1
Range 1: 25 to 33GenPeptGraphics Next Match Previous Match

Alignment statistics for match #1

| Score | Expect | Identities | Positives | Gaps |
|---|---|---|---|---|
| 32.0 bits(68) | 0.037 | 9/9(100%) | 9/9(100%) | 0/9(0%) |

```
Query  7   EYGNKLVFG  15
           EYGNKLVFG
Sbjct  25  EYGNKLVFG  33
```

```
Joachims,M.L., Leehan,K.M., Lawrence,C., Pelikan,R.C., Moore,J.S.,
            Pan,Z., Rasmussen,A., Radfar,L., Lewis,D.M., Grundahl,K.M.,
            Kelly,J.A., Wiley,G.B., Shugay,M., Chudakov,D.M., Lessard,C.J.,
            Stone,D.U., Scofield,R.H., Montgomery,C.G., Sivils,K.L.,
            Thompson,L.F. and Farris,A.D.
  TITLE     Single-cell analysis of glandular T cell receptors in Sjogren's
            syndrome
  JOURNAL   JCI Insight 1 (8) (2016)
   PUBMED   27358913
```

immunoglobulin heavy chain junction region [Homo sapiens]
Sequence ID: MBN4642704.1Length: 15Number of Matches: 1
Range 1: 3 to 11GenPeptGraphics Next Match Previous Match

Alignment statistics for match #1

| Score | Expect | Identities | Positives | Gaps |
|---|---|---|---|---|
| 24.0 bits(49) | 16 | 7/9(78%) | 8/9(88%) | 0/9(0%) |

```
Query  4   GSEEYGNKL  12
           GSE+YG KL
Sbjct  3   GSEDYGGKL  11
```


```
Jiang,R., Hoehn,K.B., Lee,C.S., Pham,M.C., Homer,R.J.,
            Detterbeck,F.C., Aban,I., Jacobson,L., Vincent,A., Nowak,R.J.,
            Kaminski,H.J., Kleinstein,S.H. and O'Connor,K.C.
  TITLE     Thymus-derived B cell clones persist in the circulation after
            thymectomy in myasthenia gravis
  JOURNAL   Proc Natl Acad Sci U S A 117 (48), 30649-30660 (2020)
   PUBMED   33199596
```


herpes simplex virus antigen specific T-cell receptor alpha chain VJ region {clone PT4} [human, Peptide Partial, 32 aa] [Homo sapiens]
Sequence ID: AAB31867.1Length: 32Number of Matches: 1
Range 1: 4 to 20GenPeptGraphics Next Match Previous Match

Alignment statistics for match #1

| Score | Expect | Identities | Positives | Gaps |
|---|---|---|---|---|
| 24.8 bits(51) | 13 | 9/17(53%) | 9/17(52%) | 8/17(47%) |

```
Query  7   EY--------GNKLVFG  15
           EY        GNKLVFG
Sbjct  4   EYFCAVSDRSGNKLVFG  20
```

# TCR diabetic 8: CASSQVRLAGGGEQFFG

T-cell receptor beta V6-2J1.4, TCRBV6-2J1.4 {complementarity determining region 3} [human, peripheral T-lymphocytes, insulin-dependent diabetes mellitus patient GAD65.05, Peptide Partial, 25 aa] [Homo sapiens]
Sequence ID: AAB46915.1Length: 25Number of Matches: 1

---

Range 1: 1 to 17GenPeptGraphics Next Match Previous Match

Alignment statistics for match #1

| Score | Expect | Identities | Positives | Gaps |
|---|---|---|---|---|
| 20.6 bits(41) | 522 | 11/18(61%) | 11/18(61%) | 2/18(11%) |

```
Query  1   CASS-QVRLAGGGEQFFG  17
           CASS   R A G+E FFG
Sbjct  1   CASSPPGR-ADGDELFFG  17
```

T-cell receptor beta V5-2J2.1, TCRBV5-2J2.1 {complementarity determining region 3} [human, peripheral T-lymphocytes, insulin-dependent diabetes mellitus patient GAD65.05, Peptide Partial, 25 aa] [Homo sapiens]

Sequence ID: AAB46921.1Length: 25Number of Matches: 1

Range 1: 1 to 17GenPeptGraphics Next Match Previous Match

Alignment statistics for match #1

| Score | Expect | Identities | Positives | Gaps |
|---|---|---|---|---|
| 31.2 bits(66) | 0.066 | 14/20(70%) | 14/20(70%) | 6/20(30%) |

```
Query  1   CASSQVRL-AGGG--EQFFG  17
           CASS   L AGGG  EQFFG
Sbjct  1   CASS---LGAGGGGNEQFFG  17
```

T-cell receptor beta chain, partial [Homo sapiens]

Sequence ID: AAS21503.1Length: 39Number of Matches: 1

Range 1: 18 to 32GenPeptGraphics Next Match Previous Match

Alignment statistics for match #1

| Score | Expect | Identities | Positives | Gaps |
|---|---|---|---|---|
| 35.0 bits(75) | 0.004 | 12/17(71%) | 14/17(82%) | 2/17(11%) |

```
Query  1   CASSQVRLAGGGEQFFG  17
           CASSQ+R  G GEQ+FG
Sbjct  18  CASSQIR--GVGEQYFG  32
```


```
Skulina,C., Schmidt,S., Dornmair,K., Babbe,H., Roers,A.,
            Rajewsky,K., Wekerle,H., Hohlfeld,R. and Goebels,N.
  TITLE     Multiple sclerosis: Brain-infiltrating CD8+ T cells persist as
            clonal expansions in the cerebrospinal fluid and blood
  JOURNAL   Proc. Natl. Acad. Sci. U.S.A. 101 (8), 2428-2433 (2004)
   PUBMED   14970318
```


T cell receptor beta chain, partial [Homo sapiens]

Sequence ID: ADP89879.1Length: 50Number of Matches: 1

Range 1: 23 to 37GenPeptGraphics Next Match Previous Match

Alignment statistics for match #1

| Score | Expect | Identities | Positives | Gaps |
|---|---|---|---|---|
| 36.3 bits(78) | 0.002 | 13/17(76%) | 14/17(82%) | 2/17(11%) |

```
Query  1   CASSQVRLAGGGEQFFG  17
           CASS   LAGGGEQ+FG
Sbjct  23  CASSP--LAGGGEQYFG  37
Biegler,B.W.
  TITLE     T cells stimulated with myelin antigens produce a varied set of
            sequence data in MS and healthy subjects
  JOURNAL   Unpublished
```

**https://www.ncbi.nlm.nih.gov/protein/ADP89879.1?report=genbank&log$=protalign&blast_rank=6&RID=HVKB12C9016 accessed 4 Oct 2023**

## TCR diabetic 9: CASSLGQGETEAFFG

T-cell receptor beta V5-2J1.1, TCRBV5-2J1.1 {complementarity determining region 3} [human, peripheral T-lymphocytes, insulin-dependent diabetes mellitus patient GAD65.05, Peptide Partial, 23 aa] [Homo sapiens]
Sequence ID: AAB46914.1Length: 23Number of Matches: 1
Range 1: 1 to 15GenPeptGraphics Next Match Previous Match

Alignment statistics for match #1

| Score | Expect | Identities | Positives | Gaps |
|---|---|---|---|---|
| 26.5 bits(55) | 2.6 | 10/15(67%) | 11/15(73%) | 0/15(0%) |

```
Query  1   CASSLGQGETEAFFG  15
           CASS  \++TEAFFG
Sbjct  1   CASSYEGADTEAFFG  15
```

T-cell receptor beta chain variable region, partial [Homo sapiens]
Sequence ID: ANO55681.1Length: 52Number of Matches: 1
Range 1: 30 to 44GenPeptGraphics Next Match Previous Match

Alignment statistics for match #1

| Score | Expect | Identities | Positives | Gaps |
|---|---|---|---|---|
| 43.9 bits(96) | 2e-06 | 14/15(93%) | 14/15(93%) | 0/15(0%) |

```
Query  1   CASSLGQGETEAFFG  15
           CASSLGQG TEAFFG
Sbjct  30  CASSLGQGSTEAFFG  44
```

T-cell receptor beta chain variable region, partial [Homo sapiens]
Sequence ID: ANO54657.1Length: 39Number of Matches: 1
Range 1: 17 to 31GenPeptGraphics Next Match Previous Match

Alignment statistics for match #1

| Score | Expect | Identities | Positives | Gaps |
|---|---|---|---|---|
| 39.7 bits(86) | 6e-05 | 13/15(87%) | 13/15(86%) | 0/15(0%) |

```
Query  1   CASSLGQGETEAFFG  15
           CASS GQG TEAFFG
Sbjct  17  CASSFGQGGTEAFFG  31
```

T-cell receptor beta chain variable region, partial [Homo sapiens]
Sequence ID: ANO56362.1Length: 33Number of Matches: 1
Range 1: 11 to 25GenPeptGraphics Next Match Previous Match

Alignment statistics for match #1

| Score | Expect | Identities | Positives | Gaps |
|---|---|---|---|---|
| 39.2 bits(85) | 7e-05 | 13/15(87%) | 13/15(86%) | 0/15(0%) |

```
Query  1   CASSLGQGETEAFFG  15
           CASS GQG TEAFFG
Sbjct  11  CASSWGQGNTEAFFG  25
```

T-cell receptor beta chain variable region, partial [Homo sapiens]
Sequence ID: ANO55651.1Length: 80Number of Matches: 2
Range 1: 58 to 72GenPeptGraphics Next Match Previous Match

Alignment statistics for match #1

| Score | Expect | Identities | Positives | Gaps |
|---|---|---|---|---|
| 38.8 bits(84) | 2e-04 | 13/15(87%) | 13/15(86%) | 0/15(0%) |

```
Query  1   CASSLGQGETEAFFG  15
           CASSLG G TEAFFG
Sbjct  58  CASSLGAGKTEAFFG  72
```

T-cell receptor beta chain variable region, partial [Homo sapiens]
Sequence ID: ANO54305.1Length: 41Number of Matches: 1
Range 1: 18 to 33GenPeptGraphics Next Match Previous Match

Alignment statistics for match #1

| Score | Expect | Identities | Positives | Gaps |
|---|---|---|---|---|

40.5 bits(88) 3e-05 14/16(88%) 14/16(87%) 1/16(6%)

```
Query  1   CASSL-GQGETEAFFG  15
           CASSL G GETEAFFG
Sbjct  18  CASSLRGRGETEAFFG  33
```

T-cell receptor beta chain variable region, partial [Homo sapiens]
Sequence ID: ANO54543.1Length: 112Number of Matches: 2
Range 1: 91 to 104GenPeptGraphics Next Match Previous Match

Alignment statistics for match #1

| Score | Expect | Identities | Positives | Gaps |
|---|---|---|---|---|
| 38.0 bits(82) | 3e-04 | 13/15(87%) | 13/15(86%) | 1/15(6%) |

```
Query  1    CASSLGQGETEAFFG  15
            CASSLGQ  TEAFFG
Sbjct  91   CASSLGQ-NTEAFFG  104
```

```
Joachims,M.L., Leehan,K.M., Lawrence,C., Pelikan,R.C., Moore,J.S.,
            Pan,Z., Rasmussen,A., Radfar,L., Lewis,D.M., Grundahl,K.M.,
            Kelly,J.A., Wiley,G.B., Shugay,M., Chudakov,D.M., Lessard,C.J.,
            Stone,D.U., Scofield,R.H., Montgomery,C.G., Sivils,K.L.,
            Thompson,L.F. and Farris,A.D.
  TITLE     Single-cell analysis of glandular T cell receptors in Sjogren's
            syndrome
  JOURNAL   JCI Insight 1 (8) (2016)
   PUBMED   27358913
```

T-cell receptor beta chain V-D-J region, partial [Homo sapiens]
Sequence ID: AAB24846.1Length: 17Number of Matches: 1
Range 1: 1 to 15GenPeptGraphics Next Match Previous Match

Alignment statistics for match #1

| Score | Expect | Identities | Positives | Gaps |
|---|---|---|---|---|
| 36.3 bits(78) | 3e-04 | 12/15(80%) | 12/15(80%) | 0/15(0%) |

```
Query  1   CASSLGQGETEAFFG  15
           CASSL  G TEAFFG
Sbjct  1   CASSLERGNTEAFFG  15
```

```
Wang,X.H., Ohmen,J.D., Uyemura,K., Rea,T.H., Kronenberg,M. and
            Modlin,R.L.
  TITLE     Selection of T lymphocytes bearing limited T-cell receptor beta
            chains in the response to a human pathogen
  JOURNAL   Proc. Natl. Acad. Sci. U.S.A. 90 (1), 188-192 (1993)
   PUBMED   8419921
```

Delayed-type hypersensitivity (DTH) is a classic measure of T-cell responsiveness to foreign antigen. To estimate the extent of the T-cell repertoire in the DTH response to a human pathogen, we measured T-cell receptor (TCR) beta-chain variable-region (V beta) gene usage in reversal reactions in leprosy. Reversal reactions represent naturally occurring DTH responses in leprosy, in which augmentation of T-cell responses to Mycobacterium leprae is concomitant with clearance of bacilli from lesions. T cells using the V beta 6-, V beta 12-, V beta 14-, and V beta 19-encoded TCRs were strikingly overrepresented in the lesions of patients as compared to blood and pre-DTH lesions from the same individuals. Furthermore, these data indicate a possible association between the predominant expression of a V beta gene segment in lesions and the major histocompatibility complex class II haplotype of the individual. V beta 6 was prominent in the lesions of four patients who were DR15, a marker of resistance in leprosy infection. Sequence analysis of V beta 6 TCRs showed frequent use of V beta 6.1 and J beta 2.7 gene segments and a conserved amino acid motif in the V-J junction in a reversal-reaction lesion, but not in blood from the same patient. The limited TCR repertoire expressed by the infiltrating T cells suggests that a limited set of antigens is recognized in the DTH response to a human pathogen. We suggest that the mechanism by which major histocompatibility complex haplotype influences DTH in this disease involves the presentation of specific peptides, with subsequent selection of specific TCRs followed by local oligoclonal expansion.

T cell receptor V beta3-D-J, partial [Homo sapiens]
Sequence ID: BAA24996.1Length: 33Number of Matches: 1
Range 1: 11 to 26GenPeptGraphics Next Match Previous Match

Alignment statistics for match #1

| Score | Expect | Identities | Positives | Gaps |
|---|---|---|---|---|
| 38.0 bits(82) | 2e-04 | 13/16(81%) | 14/16(87%) | 1/16(6%) |

```
Query  1   CASSLGQG-ETEAFFG  15
           CASS+GQG +TEAFFG
Sbjct  11  CASSMGQGASTEAFFG  26
```

```
Illes,Z., Kondo,T., Yokoyama,K., Ohashi,T., Tabira,T. and
            Yamamura,T.
  TITLE     Identification of autoimmune T cells among in vivo expanded CD25+ T cells
in multiple sclerosis
  JOURNAL   J. Immunol. 162 (3), 1811-1817 (1999)
   PUBMED   9973446
```

TCR, partial [Homo sapiens]
Sequence ID: ADQ57029.1Length: 50Number of Matches: 1

- See 1 more title(s) See all Identical Proteins(IPG)

Related Information
Identical Proteins-Identical proteins to ADQ57029.1
Range 1: 23 to 37GenPeptGraphics Next Match Previous Match

Alignment statistics for match #1

| Score | Expect | Identities | Positives | Gaps |
|---|---|---|---|---|
| 37.5 bits(81) | 4e-04 | 13/15(87%) | 13/15(86%) | 0/15(0%) |

```
Query  1   CASSLGQGETEAFFG  15
           CASSLG G TEAFFG
Sbjct  23  CASSLGGGGTEAFFG  37
```

Biegler,B.W.

```
  TITLE     T cells stimulated with myelin antigens produce a varied set of
            sequence data in MS and healthy subjects
  JOURNAL   Unpublished
```

https://www.ncbi.nlm.nih.gov/protein/ADQ57029.1?report=genbank&log$=protalign&blast_rank=14&RID=HVYMB9S2016

accessed 4 Oct 2023

## TCR diabetic 10: CASRNLGLNTEAFFG

T-cell receptor beta chain variable region, partial [Homo sapiens]
Sequence ID: ANO54635.1Length: 33Number of Matches: 1
Range 1: 11 to 25GenPeptGraphics Next Match Previous Match

Alignment statistics for match #1

| Score | Expect | Identities | Positives | Gaps |
|---|---|---|---|---|
| 34.1 bits(73) | 0.005 | 12/15(80%) | 12/15(80%) | 0/15(0%) |

```
Query  1   CASRNLGLNTEAFFG  15
           CASR   LNTEAFFG
Sbjct  11  CASRAGDLNTEAFFG  25
```

T-cell receptor beta chain variable region, partial [Homo sapiens]
Sequence ID: ANO56640.1Length: 50Number of Matches: 1
Range 1: 29 to 42GenPeptGraphics Next Match Previous Match

Alignment statistics for match #1

| Score | Expect | Identities | Positives | Gaps |
|---|---|---|---|---|
| 34.1 bits(73) | 0.007 | 12/15(80%) | 12/15(80%) | 1/15(6%) |

```
Query  1   CASRNLGLNTEAFFG  15
           CA+RN G NTEAFFG
Sbjct  29  CATRNRG-NTEAFFG  42
```

T-cell receptor beta chain variable region, partial [Homo sapiens]
Sequence ID: ANO54965.1Length: 82Number of Matches: 1
Range 1: 61 to 74GenPeptGraphics Next Match Previous Match
Alignment statistics for match #1

| Score | Expect | Identities | Positives | Gaps |
|---|---|---|---|---|
| 34.1 bits(73) | 0.007 | 12/15(80%) | 12/15(80%) | 1/15(6%) |

```
Query  1   CASRNLGLNTEAFFG  15
           CAS  LG NTEAFFG
Sbjct  61  CAS-SLGQNTEAFFG  74
```

```
Joachims,M.L., Leehan,K.M., Lawrence,C., Pelikan,R.C., Moore,J.S.,
            Pan,Z., Rasmussen,A., Radfar,L., Lewis,D.M., Grundahl,K.M.,
            Kelly,J.A., Wiley,G.B., Shugay,M., Chudakov,D.M., Lessard,C.J.,
            Stone,D.U., Scofield,R.H., Montgomery,C.G., Sivils,K.L.,
            Thompson,L.F. and Farris,A.D.
  TITLE     Single-cell analysis of glandular T cell receptors in Sjogren's
            syndrome
  JOURNAL   JCI Insight 1 (8) (2016)
```

T cell receptor beta chain, partial [Homo sapiens]
Sequence ID: ABO26891.1Length: 109Number of Matches: 1
Range 1: 85 to 99GenPeptGraphics Next Match Previous Match
Alignment statistics for match #1

| Score | Expect | Identities | Positives | Gaps |
|---|---|---|---|---|
| 34.1 bits(73) | 0.007 | 13/16(81%) | 13/16(81%) | 2/16(12%) |

```
Query  1   CAS-RNLGLNTEAFFG  15
           CAS R   GLNTEAFFG
Sbjct  85  CASKRG-GLNTEAFFG  99
```

```
Wu,H.D., Maurer,M.S., Friedman,R.A., Marboe,C.C.,
            Ruiz-Vazquez,E.M., Ramakrishnan,R., Schwartz,A., Tilson,M.D.,
            Stewart,A.S. and Winchester,R.
  TITLE     The lymphocytic infiltration in calcific aortic stenosis
            predominantly consists of clonally expanded T cells
```

JOURNAL   J. Immunol. 178 (8), 5329-5339 (2007)
PUBMED   17404318

## TCR diabetic 11: CASSFRRBTDTQYFG

myelin basic protein (143-168)-specific TCR beta-chain {clone HE.1D5} [human, multiple sclerosis patient, T cells, Peptide Partial, 29 aa] [Homo sapiens]
Sequence ID: AAB31428.1Length: 29Number of Matches: 1
Range 1: 1 to 17GenPeptGraphics Next Match Previous Match

Alignment statistics for match #1

| Score | Expect | Identities | Positives | Gaps |
|---|---|---|---|---|
| 37.1 bits(80) | 3e-04 | 12/17(71%) | 14/17(82%) | 2/17(11%) |

```
Query  1    CASSF--RRBTDTQYFG  15
            CASS+  R +TDTQYFG
Sbjct  1    CASSYSGRVDTDTQYFG  17
```

## TCR DIA K2.12 CASSSDRLGNQPQHF

T-cell receptor beta V23-2J1.5, TCRBV23-2J1.5 {complementarity determining region 3} [human, peripheral T-lymphocytes, insulin-dependent diabetes mellitus patient GAD65.05, Peptide Partial, 25 aa] [Homo sapiens]
Sequence ID: AAB46923.1Length: 25Number of Matches: 1
Range 1: 1 to 16GenPeptGraphics Next Match Previous Match

Alignment statistics for match #1

| Score | Expect | Identities | Positives | Gaps |
|---|---|---|---|---|
| 27.8 bits(58) | 0.93 | 12/17(71%) | 13/17(76%) | 3/17(17%) |

```
Query  1    CASS-SDR-LGNQPQHF  15
            CASS  DR +G QPQHF
Sbjct  1    CASSLWDRGMG-QPQHF  16
```

myelin basic protein specific T-cell receptor V beta-D beta-J beta, MBP reactive TCR VDJ beta {clone KL-1(4), rearranged CDR3 region} [human, brain plaques, HLA phenotype 1, Peptide Partial, 25 aa] [Homo sapiens]
Sequence ID: AAB25595.1Length: 25Number of Matches: 1
Range 1: 2 to 16GenPeptGraphics Next Match Previous Match

Alignment statistics for match #1

| Score | Expect | Identities | Positives | Gaps |
|---|---|---|---|---|
| 25.7 bits(53) | 5.7 | 11/15(73%) | 11/15(73%) | 0/15(0%) |

```
Query  1    CASSSDRLGNQPQHF  15
            CASS DRL N+P HF
Sbjct  2    CASSLDRLYNSPLHF  16
```

myelin basic protein specific T-cell receptor V beta-D beta-J beta, MBP reactive TCR VDJ beta {clone KL-1(6), rearranged CDR3 region} [human, brain plaques, HLA phenotype 1, Peptide Partial, 25 aa] [Homo sapiens]

Sequence ID: AAB25597.1Length: 25Number of Matches: 1

Range 1: 2 to 16GenPeptGraphics Next Match Previous Match

Alignment statistics for match #1

| Score | Expect | Identities | Positives | Gaps |
|---|---|---|---|---|
| 23.5 bits(48) | 34 | 10/15(67%) | 10/15(66%) | 0/15(0%) |

```
Query  1    CASSSDRLGNQPQHF  15
            CASS+ RL+N+P HF
Sbjct  2    CASSQLRLANSPLHF  16
```

myelin basic protein specific T-cell receptor V beta-D beta-J beta, MBP reactive TCR VDJ beta {clone KL-1(3), rearranged CDR3 region} [human, brain plaques, HLA phenotype 1, Peptide Partial, 24 aa] [Homo sapiens]

Sequence ID: AAB25594.1Length: 24Number of Matches: 1

Range 1: 2 to 15GenPeptGraphics Next Match Previous Match

Alignment statistics for match #1

| Score | Expect | Identities | Positives | Gaps |
|---|---|---|---|---|
| 23.1 bits(47) | 48 | 10/15(67%) | 10/15(66%) | 1/15(6%) |

```
Query  1    CASSSDRLGNQPQHF  15
            CASS  RL+N+P HF
Sbjct  2    CASSL-RLANSPLHF  15
```

myelin basic protein specific T-cell receptor V beta-D beta-J beta, MBP reactive TCR VDJ beta {clone KL-1(9), rearranged CDR3 region} [human, brain plaques, HLA phenotype 1, Peptide Partial, 24 aa] [Homo sapiens]

Sequence ID: AAB25600.1Length: 24Number of Matches: 1

Range 1: 2 to 13GenPeptGraphics Next Match Previous Match

Alignment statistics for match #1

| Score | Expect | Identities | Positives | Gaps |
|---|---|---|---|---|
| 22.3 bits(45) | 98 | 8/12(67%) | 9/12(75%) | 1/12(8%) |

```
Query  1    CASSSD-RLGNQ  11
            CASSSD R+ +Q
Sbjct  2    CASSSDGRMSTQ  13
```

---

TCR V beta {V-J junction, clone MS7-1B10} [human, multiple sclerosis patient, myelin basic protein-reactive T cells after vaccination, Peptide Partial, 29 aa] [Homo sapiens]
Sequence ID: AAB35720.1Length: 29Number of Matches: 1
Range 1: 3 to 15GenPeptGraphics Next Match Previous Match

Alignment statistics for match #1

| Score | Expect | Identities | Positives | Gaps |
|---|---|---|---|---|
| 29.1 bits(61) | 0.36 | 11/16(69%) | 11/16(68%) | 4/16(25%) |

```
Query  1    CASSSDRL-GNQPQHF  15
            CASS    L  NQPQHF
Sbjct  3    CASS---LYSNQPQHF  15
```

T-cell receptor beta chain variable region, partial [Homo sapiens]
Sequence ID: ANO54807.1Length: 90Number of Matches: 1
Range 1: 67 to 81GenPeptGraphics Next Match Previous Match

Alignment statistics for match #1

| Score | Expect | Identities | Positives | Gaps |
|---|---|---|---|---|
| 43.5 bits(95) | 3e-06 | 13/15(87%) | 13/15(86%) | 0/15(0%) |

```
Query  1    CASSSDRLGNQPQHF  15
            CASS  RLGNQPQHF
Sbjct  67   CASSGQRLGNQPQHF  81
```

T-cell receptor beta chain variable region, partial [Homo sapiens]
Sequence ID: ANO54529.1Length: 38Number of Matches: 1
Range 1: 15 to 29GenPeptGraphics Next Match Previous Match

Alignment statistics for match #1

| Score | Expect | Identities | Positives | Gaps |
|---|---|---|---|---|
| 40.5 bits(88) | 3e-05 | 13/15(87%) | 13/15(86%) | 0/15(0%) |

```
Query  1    CASSSDRLGNQPQHF  15
            CASS DR GNQPQHF
Sbjct  15   CASSPDRGGNQPQHF  29
```

T-cell receptor beta chain variable region, partial [Homo sapiens]
Sequence ID: ANO54774.1Length: 35Number of Matches: 1
Range 1: 11 to 26GenPeptGraphics Next Match Previous Match

Alignment statistics for match #1

| Score | Expect | Identities | Positives | Gaps |
|---|---|---|---|---|
| 35.4 bits(76) | 0.002 | 12/16(75%) | 12/16(75%) | 1/16(6%) |

```
Query  1    CASS-SDRLGNQPQHF  15
            CASS  DR  NQPQHF
Sbjct  11   CASSQEDRVSNQPQHF  26
```

T-cell receptor beta chain variable region, partial [Homo sapiens]
Sequence ID: ANO55590.1Length: 45Number of Matches: 1
Range 1: 23 to 36GenPeptGraphics Next Match Previous Match

Alignment statistics for match #1

| Score | Expect | Identities | Positives | Gaps |
|---|---|---|---|---|
| 34.6 bits(74) | 0.005 | 12/15(80%) | 12/15(80%) | 1/15(6%) |

```
Query  1   CASSSDRLGNQPQHF  15
           CASS DR G QPQHF
Sbjct  23  CASSDDR-GYQPQHF  36
```

```
Joachims,M.L., Leehan,K.M., Lawrence,C., Pelikan,R.C., Moore,J.S.,
            Pan,Z., Rasmussen,A., Radfar,L., Lewis,D.M., Grundahl,K.M.,
            Kelly,J.A., Wiley,G.B., Shugay,M., Chudakov,D.M., Lessard,C.J.,
            Stone,D.U., Scofield,R.H., Montgomery,C.G., Sivils,K.L.,
            Thompson,L.F. and Farris,A.D.
  TITLE     Single-cell analysis of glandular T cell receptors in Sjogren's
            syndrome
  JOURNAL   JCI Insight 1 (8) (2016)
```

# TCR DIA K2.16 CASSQDRLRGVADTQYF

T-cell receptor beta chain variable region, partial [Homo sapiens]
Sequence ID: ANO54804.1Length: 52Number of Matches: 1
Range 1: 29 to 43GenPeptGraphics Next Match Previous Match

Alignment statistics for match #1

| Score | Expect | Identities | Positives | Gaps |
|---|---|---|---|---|
| 40.1 bits(87) | 7e-05 | 14/17(82%) | 14/17(82%) | 2/17(11%) |

```
Query  1   CASSQDRLRGVADTQYF  17
           CASSQ   RGVADTQYF
Sbjct  29  CASSQ--YRGVADTQYF  43
```

```
Joachims,M.L., Leehan,K.M., Lawrence,C., Pelikan,R.C., Moore,J.S.,
            Pan,Z., Rasmussen,A., Radfar,L., Lewis,D.M., Grundahl,K.M.,
```

Kelly,J.A., Wiley,G.B., Shugay,M., Chudakov,D.M., Lessard,C.J., Stone,D.U., Scofield,R.H., Montgomery,C.G., Sivils,K.L., Thompson,L.F. and Farris,A.D.
TITLE Single-cell analysis of glandular T cell receptors in Sjogren's syndrome
JOURNAL JCI Insight 1 (8) (2016)
PUBMED 27358913

T cell receptor beta chain, partial [Homo sapiens]
Sequence ID: ABO26954.1Length: 77Number of Matches: 1
Range 1: 52 to 66GenPeptGraphics Next Match Previous Match

Alignment statistics for match #1

| Score | Expect | Identities | Positives | Gaps |
|---|---|---|---|---|
| 35.4 bits(76) | 0.003 | 12/17(71%) | 12/17(70%) | 2/17(11%) |

```
Query  1   CASSQDRLRGVADTQYF  17
           CAS QDR  G  DTQYF
Sbjct  52  CASRQDR--GLPDTQYF  66
```

Wu,H.D., Maurer,M.S., Friedman,R.A., Marboe,C.C., Ruiz-Vazquez,E.M., Ramakrishnan,R., Schwartz,A., Tilson,M.D., Stewart,A.S. and Winchester,R.
TITLE The lymphocytic infiltration in calcific aortic stenosis predominantly consists of clonally expanded T cells
JOURNAL J. Immunol. 178 (8), 5329-5339 (2007)
PUBMED 17404318

myelin basic protein specific T-cell receptor V beta-D beta-J beta, MBP reactive TCR VDJ beta {clone SE(9), rearranged CDR3 region} [human, inflammatory brain lesions, HLA phenotype 1, Peptide Partial, 25 aa] [Homo sapiens]
Sequence ID: AAB25643.1Length: 25Number of Matches: 1
Range 1: 2 to 16GenPeptGraphics Next Match Previous Match

Alignment statistics for match #1

| Score | Expect | Identities | Positives | Gaps |
|---|---|---|---|---|
| 29.9 bits(63) | 0.20 | 13/17(76%) | 13/17(76%) | 2/17(11%) |

```
Query  1   CASSQDRLRGVADTQYF  17
           CASS D L G ADTQYF
Sbjct  2   CASSLD-L-GKADTQYF  16
```

T cell receptor beta, partial [Homo sapiens]
Sequence ID: AAV32381.1Length: 50Number of Matches: 1

---

Range 1: 20 to 36GenPeptGraphics Next Match Previous Match

Alignment statistics for match #1

| Score | Expect | Identities | Positives | Gaps |
|---|---|---|---|---|
| 37.1 bits(80) | 8e-04 | 13/17(76%) | 13/17(76%) | 0/17(0%) |

```
Query  1   CASSQDRLRGVADTQYF  17
           CASSQD L+   ADTQYF
Sbjct  20  CASSQDPLNSYADTQYF  36
```

```
Biegler,B.W., Yan,S.X., Ortega,S.B., Tennakoon,D.K., Racke,M.K. and
            Karandikar,N.J.
  TITLE     Glatiramer acetate (GA) therapy induces a focused, oligoclonal CD8+
            T-cell repertoire in multiple sclerosis
  JOURNAL   J. Neuroimmunol. 180 (1-2), 159-171 (2006)
   PUBMED   16935352
```

TCR, partial [Homo sapiens]
Sequence ID: ADQ57201.1Length: 50Number of Matches: 1
Range 1: 19 to 36GenPeptGraphics Next Match Previous Match

Alignment statistics for match #1

| Score | Expect | Identities | Positives | Gaps |
|---|---|---|---|---|
| 33.7 bits(72) | 0.013 | 13/18(72%) | 13/18(72%) | 1/18(5%) |

```
Query  1   CASSQD-RLRGVADTQYF  17
           CASSQD  L G +DTQYF
Sbjct  19  CASSQDPPLAGGTDTQYF  36
```

```
Biegler,B.W.
  TITLE     T cells stimulated with myelin antigens produce a varied set of
            sequence data in MS and healthy subjects
  JOURNAL   Unpublished
```

**https://www.ncbi.nlm.nih.gov/protein/ADQ57201.1?report=genbank&log$=protalign&blast_rank=19&RID=HXFDJSPW013**

**ACCESSED 5 OCT 2023**

**TCR DIA K2.4 CASSQEADIQYF**

---

T cell receptor beta chain, partial [Homo sapiens]
Sequence ID: BAC01027.1Length: 30Number of Matches: 1
Range 1: 3 to 16GenPeptGraphics Next Match Previous Match

Alignment statistics for match #1

| Score | Expect | Identities | Positives | Gaps |
|---|---|---|---|---|
| 31.2 bits(66) | 0.037 | 10/14(71%) | 11/14(78%) | 2/14(14%) |

```
Query  1   CASSQE--ADIQYF  12
           CASSQ+  AD QYF
Sbjct  3   CASSQDSGADTQYF  16
```

```
Saito,M., Higuchi,I., Saito,A., Izumo,S., Usuku,K., Bangham,C.R.
            and Osame,M.
  TITLE     Molecular analysis of T cell clonotypes in muscle-infiltrating
            lymphocytes from patients with human T lymphotropic virus type 1
            polymyositis
  JOURNAL   J. Infect. Dis. 186 (9), 1231-1241 (2002)
   PUBMED   12402192
```

T cell receptor beta, partial [Homo sapiens]
Sequence ID: AAV31431.1Length: 50Number of Matches: 1
Range 1: 23 to 36GenPeptGraphics Next Match Previous Match

Alignment statistics for match #1

| Score | Expect | Identities | Positives | Gaps |
|---|---|---|---|---|
| 33.7 bits(72) | 0.006 | 11/14(79%) | 11/14(78%) | 2/14(14%) |

```
Query  1   CASSQ--EADIQYF  12
           CASSQ  EAD QYF
Sbjct  23  CASSQTPEADTQYF  36
Biegler,B.W., Yan,S.X., Ortega,S.B., Tennakoon,D.K., Racke,M.K. and
            Karandikar,N.J.
  TITLE     Glatiramer acetate (GA) therapy induces a focused, oligoclonal CD8+
            T-cell repertoire in multiple sclerosis
  JOURNAL   J. Neuroimmunol. 180 (1-2), 159-171 (2006)
   PUBMED   16935352
```

Structure of the HLA-E-VMAPRTLIL/KK50.4 TCR complex [Homo sapiens]
Sequence ID: 2ESV_ELength: 240Number of Matches: 1
Related Information
Structure-3D structure displays
Range 1: 90 to 101GenPeptGraphics Next Match Previous Match

Alignment statistics for match #1

| Score | Expect | Identities | Positives | Gaps |
|---|---|---|---|---|
| 30.3 bits(64) | 0.088 | 9/12(75%) | 10/12(83%) | 0/12(0%) |

```
Query  1    CASSQEADIQYF  12
            CASSQ+ D QYF
Sbjct  90   CASSQDRDTQYF  10
```

Hoare,H.L., Sullivan,L.C., Pietra,G., Clements,C.S., Lee,E.J., Ely,L.K., Beddoe,T., Falco,M., Kjer-Nielsen,L., Reid,H.H., McCluskey,J., Moretta,L., Rossjohn,J. and Brooks,A.G.

TITLE Structural basis for a major histocompatibility complex class Ib-restricted T cell response [TO CMV]

JOURNAL Nat. Immunol. 7 (3), 256-264 (2006)

PUBMED 16474394

T cell receptor Vb7-D-J-C, partial [Homo sapiens]

Sequence ID: BAA06781.1Length: 27Number of Matches: 1

Related Information

Identical Proteins-Identical proteins to PH0232

Range 1: 2 to 13GenPeptGraphics Next Match Previous Match

Alignment statistics for match #1

| Score | Expect | Identities | Positives | Gaps |
|---|---|---|---|---|
| 29.9 bits(63) | 0.10 | 9/12(75%) | 9/12(75%) | 0/12(0%) |

```
Query  1   CASSQEADIQYF  12
           CASS  AD QYF
Sbjct  2   CASSRNADTQYF  13
```

Hara,H., Morita,M., Iwaki,T., Hatae,T., Itoyama,Y., Kitamoto,T., Akizuki,S., Goto,I. and Watanabe,T.

TITLE Detection of human T lymphotrophic virus type I (HTLV-I) proviral DNA and analysis of T cell receptor V beta CDR3 sequences in spinal cord lesions of HTLV-I-associated myelopathy/tropical spastic paraparesis

JOURNAL J. Exp. Med. 180 (3), 831-839 (1994)

PUBMED 8064235

Chain EEE, T cell receptor beta variable 14,T cell receptor beta joining 2-3,T cell receptor beta constant 2 [Homo sapiens]

Sequence ID: 7NDT_EEELength: 243Number of Matches: 1

- See 1 more title(s) See all Identical Proteins(IPG)

Related Information

---

Identical Proteins-Identical proteins to 7NDT_EEE
Range 1: 93 to 104GenPeptGraphics Next Match Previous Match

Alignment statistics for match #1

| Score | Expect | Identities | Positives | Gaps |
|---|---|---|---|---|
| 30.3 bits(64) | 0.088 | 9/12(75%) | 10/12(83%) | 0/12(0%) |

```
Query  1     CASSQEADIQYF  12
             CASSQ+ D+QYF
Sbjct  93    CASSQDRDTQYF  104
```

```
Barber,C., De Souza,V.A., Paterson,R.L., Martin-Urdiroz,M.,
            Mulakkal,N.C., Srikannathasan,V., Connolly,M., Phillips,G.,
            Foong-Leong,T., Pengelly,R., Karuppiah,V., Grant,T., Dembek,M.,
            Verma,A., Gibbs-Howe,D., Blicher,T.H., Knox,A., Robinson,R.A.,
            Cole,D.K. and Leonard,S.
  TITLE     Structure-guided stabilization of pathogen-derived peptide-HLA-E
            complexes using non-natural amino acids conserves native TCR
            recognition [OF M. TUBERCULOSIS]
  JOURNAL   Eur J Immunol 52 (4), 618-632 (2022)
   PUBMED   35108401
```

T cell receptor beta chain, partial [Homo sapiens]
Sequence ID: ABR67605.1Length: 17Number of Matches: 1
Range 1: 2 to 14GenPeptGraphics Next Match Previous Match

Alignment statistics for match #1

| Score | Expect | Identities | Positives | Gaps |
|---|---|---|---|---|
| 29.1 bits(61) | 0.12 | 9/13(69%) | 10/13(76%) | 1/13(7%) |

```
Query  1    CASSQEA-DIQYF  12
            CASSQ+   D QYF
Sbjct  2    CASSQDSTDTQYF  14
```

```
Menezes,C.A.S., Sullivan,A.K., Falta,M.T., Mack,D.G., Freed,B.M.,
            Rocha,M.O.C., Gollob,K.J., Fontenot,A.P. and Dutra,W.O.
  TITLE     Oligoclonal Expansions among Specific V Expressing T Cells in Human
            Chagas Disease
  JOURNAL   Unpublished
```

**https://www.ncbi.nlm.nih.gov/protein/ABR67605.1?report=genbank&log$=protalign&blast_rank=16&RID=HXH23S4B016 ACCESSED 5 OCT 2023**

---

T-cell receptor beta chain variable region, partial [Homo sapiens]
Sequence ID: ANO55208.1Length: 78Number of Matches: 1
Range 1: 57 to 69GenPeptGraphics Next Match Previous Match

Alignment statistics for match #1

| Score | Expect | Identities | Positives | Gaps |
|---|---|---|---|---|
| 29.9 bits(63) | 0.13 | 10/13(77%) | 10/13(76%) | 1/13(7%) |

```
Query  1   CASSQE-ADIQYF  12
           CASSQ+ AD+QYF
Sbjct  57  CASSQNRADTQYF  69
```

```
Joachims,M.L., Leehan,K.M., Lawrence,C., Pelikan,R.C., Moore,J.S.,
            Pan,Z., Rasmussen,A., Radfar,L., Lewis,D.M., Grundahl,K.M.,
            Kelly,J.A., Wiley,G.B., Shugay,M., Chudakov,D.M., Lessard,C.J.,
            Stone,D.U., Scofield,R.H., Montgomery,C.G., Sivils,K.L.,
            Thompson,L.F. and Farris,A.D.
  TITLE     Single-cell analysis of glandular T cell receptors in Sjogren's
            syndrome
  JOURNAL   JCI Insight 1 (8) (2016)
   PUBMED   27358913
```

myelin basic protein specific T-cell receptor V beta-D beta-J beta, MBP reactive TCR VDJ beta {CDR3 region, clone BM(5)} [human, peripheral blood lymphocytes, HLA phenotype 1, Peptide Partial, 23 aa] [Homo sapiens]
Sequence ID: AAB25666.1Length: 23Number of Matches: 1
Range 1: 2 to 14GenPeptGraphics Next Match Previous Match

Alignment statistics for match #1

| Score | Expect | Identities | Positives | Gaps |
|---|---|---|---|---|
| 27.8 bits(58) | 0.54 | 10/13(77%) | 10/13(76%) | 1/13(7%) |

```
Query  1   CASSQEAD-IQYF  12
           CASSQEA+  QYF
Sbjct  2   CASSQEASYEQYF  14
```

myelin basic protein specific T-cell receptor V beta-D beta-J beta, MBP reactive TCR VDJ beta {clone SE(9), rearranged CDR3 region} [human, inflammatory brain lesions, HLA phenotype 1, Peptide Partial, 25 aa] [Homo sapiens]
Sequence ID: AAB25643.1Length: 25Number of Matches: 1

Range 1: 2 to 16GenPeptGraphics Next Match Previous Match

Alignment statistics for match #1

| Score | Expect | Identities | Positives | Gaps |
|---|---|---|---|---|
| 25.2 bits(52) | 5.2 | 9/15(60%) | 10/15(66%) | 3/15(20%) |

```
Query  1    CASSQE---ADIQYF  12
            CASS +   AD QYF
Sbjct  2    CASSLDLGKADTQYF  16
```

## S2: Complete BLAST Results of Autoimmune Myocarditis TCR Similarities to Human Myelin Basic Protein

## 7 of 35 have an 8/10 identities sequence with no more than a single spacer; nothing better = 20%

## TCR Heart 2.1 CASSQDVAGLGFFG

myelin basic protein-specific T-cell receptor beta chain VDJ region {clone JK1.16} [human, peripheral blood mononuclear cells, Peptide Partial, 27 aa] [Homo sapiens]
Sequence ID: AAB33031.1Length: 27Number of Matches: 1
Range 1: 1 to 19GenPeptGraphics Next Match Previous Match

Alignment statistics for match #1

| Score | Expect | Identities | Positives | Gaps |
|---|---|---|---|---|
| 20.6 bits(41) | 367 | 10/20(50%) | 11/20(55%) | 7/20(35%) |

```
Query  1    CASSQDVAGLG------FFG  14
            CASSQ+  G G      FFG
Sbjct  1    CASSQE-EGAGGPFNEQFFG  19
```

myelin basic protein specific T-cell receptor V beta-D beta-J beta, MBP reactive TCR VDJ beta {CDR3 region, clone BM(5)} [human, peripheral blood lymphocytes, HLA phenotype 1, Peptide Partial, 23 aa] [Homo sapiens]
Sequence ID: AAB25666.1Length: 23Number of Matches: 1
Range 1: 2 to 7GenPeptGraphics Next Match Previous Match

Alignment statistics for match #1

| Score | Expect | Identities | Positives | Gaps |
|---|---|---|---|---|
| 19.3 bits(38) | 1013 | 5/6(83%) | 6/6(100%) | 0/6(0%) |

```
Query  1  CASSQD  6
```

```
          CASSQ+
Sbjct  2  CASSQE  7
```

myelin basic protein specific T-cell receptor V beta-D beta-J beta, MBP reactive TCR VDJ beta {clone KL-3(8), rearranged CDR3 region} [human, brain plaques, HLA phenotype 1, Peptide Partial, 25 aa] [Homo sapiens]

Sequence ID: AAB25611.1Length: 25Number of Matches: 1

Range 1: 2 to 17GenPeptGraphics Next Match Previous Match

Alignment statistics for match #1

| Score | Expect | Identities | Positives | Gaps |
|---|---|---|---|---|
| 19.3 bits(38) | 1034 | 9/16(56%) | 10/16(62%) | 2/16(12%) |

```
Query  1   CASSQDVAGLG--FFG  14
           CASS + AG    FFG
Sbjct  2   CASSLELAGYNEQFFG  17
```

myelin basic protein specific T-cell receptor V beta-D beta-J beta, MBP reactive TCR VDJ beta {clone KL-3(17), rearranged CDR3 region} [human, brain plaques, HLA phenotype 1, Peptide Partial, 19 aa] [Homo sapiens]

Sequence ID: AAB25620.1Length: 19Number of Matches: 1

Range 1: 2 to 6GenPeptGraphics Next Match Previous Match

Alignment statistics for match #1

| Score | Expect | Identities | Positives | Gaps |
|---|---|---|---|---|
| 18.5 bits(36) | 1925 | 5/5(100%) | 5/5(100%) | 0/5(0%) |

```
Query  1  CASSQ  5
          CASSQ
Sbjct  2  CASSQ  6
```

## TCR Heart 2.2 CASSQDVAGLAFFG

myelin basic protein specific T-cell receptor V beta-D beta-J beta, MBP reactive TCR VDJ beta {CDR3 region, clone BM(5)} [human, peripheral blood lymphocytes, HLA phenotype 1, Peptide Partial, 23 aa] [Homo sapiens]

Sequence ID: AAB25666.1Length: 23Number of Matches: 1

Range 1: 2 to 7GenPeptGraphics Next Match Previous Match

Alignment statistics for match #1

---

| Score | Expect | Identities | Positives | Gaps |
|---|---|---|---|---|
| 19.3 bits(38) | 1013 | 5/6(83%) | 6/6(100%) | 0/6(0%) |

```
Query  1  CASSQD  6
          CASSQ+
Sbjct  2  CASSQE  7
```

myelin basic protein-specific T-cell receptor beta chain VDJ region {clone JK1.16} [human, peripheral blood mononuclear cells, Peptide Partial, 27 aa] [Homo sapiens]

Sequence ID: AAB33031.1Length: 27Number of Matches: 1

Range 1: 1 to 6GenPeptGraphics Next Match Previous Match

Alignment statistics for match #1

| Score | Expect | Identities | Positives | Gaps |
|---|---|---|---|---|
| 19.3 bits(38) | 1049 | 5/6(83%) | 6/6(100%) | 0/6(0%) |

```
Query  1  CASSQD  6
          CASSQ+
Sbjct  1  CASSQE  6
```

myelin basic protein specific T-cell receptor V beta-D beta-J beta, MBP reactive TCR VDJ beta {clone KL-3(8), rearranged CDR3 region} [human, brain plaques, HLA phenotype 1, Peptide Partial, 25 aa] [Homo sapiens]

Sequence ID: AAB25611.1Length: 25Number of Matches: 1

Range 1: 2 to 17GenPeptGraphics Next Match Previous Match

Alignment statistics for match #1

| Score | Expect | Identities | Positives | Gaps |
|---|---|---|---|---|
| 18.9 bits(37) | 1468 | 9/16(56%) | 10/16(62%) | 2/16(12%) |

```
Query  1   CASSQDVAGL--AFFG  14
           CASS + AG    FFG
Sbjct  2   CASSLELAGYNEQFFG  17
```

**TCR MYO 2.3**

**NONE**

## TCR Heart 2.4 CASSALRGVYFFG

myelin basic protein-specific T-cell receptor beta chain VDJ region {clone FN2.2} [human, peripheral blood mononuclear cells, Peptide Partial, 25 aa] [Homo sapiens]
Sequence ID: AAB33027.1Length: 25Number of Matches: 1
Range 1: 1 to 17GenPeptGraphics Next Match Previous Match

Alignment statistics for match #1

| Score | Expect | Identities | Positives | Gaps |
|---|---|---|---|---|
| 23.1 bits(47) | 37 | 11/18(61%) | 11/18(61%) | 6/18(33%) |

```
Query  1    CASSALR--GVY---FFG  13
            CASS LR  G Y   FFG
Sbjct  1    CASS-LRGSGTYNEQFFG  17
```

myelin basic protein-specific T-cell receptor beta chain VDJ region {clone HOO24} [human, peripheral blood mononuclear cells, Peptide Partial, 25 aa] [Homo sapiens]
Sequence ID: AAB33029.1Length: 25Number of Matches: 1
Range 1: 1 to 16GenPeptGraphics Next Match Previous Match

Alignment statistics for match #1

| Score | Expect | Identities | Positives | Gaps |
|---|---|---|---|---|
| 21.4 bits(43) | 154 | 9/16(56%) | 9/16(56%) | 5/16(31%) |

```
Query  1    CASSAL-RGV----YF  11
            CASS L RG     YF
Sbjct  1    CASSSLARGASYEQYF  16
```

myelin basic protein-specific T-cell receptor beta chain VDJ region {clone HOO24} [human, peripheral blood mononuclear cells, Peptide Partial, 25 aa] [Homo sapiens]
Sequence ID: AAB33029.1Length: 25Number of Matches: 1
Range 1: 1 to 16GenPeptGraphics Next Match Previous Match

Alignment statistics for match #1

| Score | Expect | Identities | Positives | Gaps |
|---|---|---|---|---|
| 21.4 bits(43) | 154 | 9/16(56%) | 9/16(56%) | 5/16(31%) |

```
Query  1    CASSAL-RGV----YF  11
            CASS L RG     YF
Sbjct  1    CASSSLARGASYEQYF  16
```

## TCR Heart 2.5 CASSEGFDRDHLFG

myelin basic protein specific T-cell receptor V beta-D beta-J beta, MBP reactive TCR VDJ beta {clone KL-1(12), rearranged CDR3 region} [human, brain plaques, HLA phenotype 1, Peptide Partial, 23 aa] [Homo sapiens]
Sequence ID: AAB25603.1Length: 23Number of Matches: 1
Range 1: 2 to 15GenPeptGraphics Next Match Previous Match

Alignment statistics for match #1

| Score | Expect | Identities | Positives | Gaps |
|---|---|---|---|---|
| 17.6 bits(34) | 4131 | 9/16(56%) | 10/16(62%) | 4/16(25%) |

```
Query  1    CASS--EGFDRDHLFG  14
            CASS  EG  R + FG
Sbjct  2    CASSGGEG--RAQYFG  15
```

myelin basic protein-specific T-cell receptor beta chain VDJ region {clone JK1.16} [human, peripheral blood mononuclear cells, Peptide Partial, 27 aa] [Homo sapiens]
Sequence ID: AAB33031.1Length: 27Number of Matches: 1
Range 1: 1 to 19GenPeptGraphics Next Match Previous Match

Alignment statistics for match #1

| Score | Expect | Identities | Positives | Gaps |
|---|---|---|---|---|
| 17.6 bits(34) | 4246 | 9/20(45%) | 12/20(60%) | 7/20(35%) |

```
Query  1    CASS--EG----FDRDHLFG  14
            CASS  EG    F+ ++ FG
Sbjct  1    CASSQEEGAGGPFN-EQFFG  19
```

## TCR Heart 2.6 CASSQGLGYTFFG

myelin basic protein specific T-cell receptor V beta-D beta-J beta, MBP reactive TCR VDJ beta {CDR3 region, clone JO(1)} [human, normal peripheral blood lymphocytes, Peptide Partial, 25 aa] [Homo sapiens]
Sequence ID: AAB25656.1Length: 25Number of Matches: 1
Range 1: 2 to 16GenPeptGraphics Next Match Previous Match

Alignment statistics for match #1

| Score | Expect | Identities | Positives | Gaps |
|---|---|---|---|---|
| 22.3 bits(45) | 75 | 9/15(60%) | 9/15(60%) | 4/15(26%) |

```
Query  1    CASSQGL----GYTF  11
            CASS  L    GYTF
```

```
Sbjct  2   CASSVALQDRYGYTF  16
```

myelin basic protein specific T-cell receptor V beta-D beta-J beta, MBP reactive TCR VDJ beta {clone SE(4), rearranged CDR3 region} [human, inflammatory brain lesions, HLA phenotype 1, Peptide Partial, 25 aa] [Homo sapiens]

Sequence ID: AAB25638.1Length: 25Number of Matches: 1

Range 1: 2 to 10GenPeptGraphics Next Match Previous Match

Alignment statistics for match #1

| Score | Expect | Identities | Positives | Gaps |
|---|---|---|---|---|
| 21.4 bits(43) | 154 | 7/9(78%) | 7/9(77%) | 0/9(0%) |

```
Query  1   CASSQGLGY   9
           CASSQ+ GY
Sbjct  2   CASSQATGY   10
```

myelin basic protein specific T-cell receptor V beta-D beta-J beta, MBP reactive TCR VDJ beta {clone KL-3(8), rearranged CDR3 region} [human, brain plaques, HLA phenotype 1, Peptide Partial, 25 aa] [Homo sapiens]

Sequence ID: AAB25611.1Length: 25Number of Matches: 1

Range 1: 2 to 17GenPeptGraphics Next Match Previous Match

Alignment statistics for match #1

| Score | Expect | Identities | Positives | Gaps |
|---|---|---|---|---|
| 21.4 bits(43) | 154 | 10/16(63%) | 10/16(62%) | 3/16(18%) |

```
Query  1    CASSQGL-GYT--FFG  13
            CASS  L GY   FFG
Sbjct  2    CASSLELAGYNEQFFG  17
```

myelin basic protein (143-168)-specific TCR beta-chain {clone BM3.3} [human, multiple sclerosis patient, T cells, Peptide Partial, 26 aa] [Homo sapiens]

Sequence ID: AAB31427.1Length: 26Number of Matches: 1

Range 1: 1 to 14GenPeptGraphics Next Match Previous Match

Alignment statistics for match #1

| Score | Expect | Identities | Positives | Gaps |
|---|---|---|---|---|
| 21.4 bits(43) | 155 | 10/16(63%) | 11/16(68%) | 5/16(31%) |

```
Query  1    CASSQGL--GYT-FFG  13
            CASS  L  GYT +FG
Sbjct  1    CASS--LTRGYTQYFG  14
```

myelin basic protein specific T-cell receptor V beta-D beta-J beta, MBP reactive TCR VDJ beta {clone SE(1), rearranged CDR3 region} [human, inflammatory brain lesions, HLA phenotype 1, Peptide Partial, 26 aa] [Homo sapiens]

Sequence ID: AAB25635.1Length: 26Number of Matches: 1

Range 1: 2 to 17GenPeptGraphics Next Match Previous Match

Alignment statistics for match #1

| Score | Expect | Identities | Positives | Gaps |
|---|---|---|---|---|
| 21.4 bits(43) | 155 | 9/16(56%) | 9/16(56%) | 5/16(31%) |

```
Query  1   CASS-----QGLGYTF  11
           CASS       G GYTF
Sbjct  2   CASSPCLFNRGYGYTF  17
```

myelin basic protein specific T-cell receptor V beta-D beta-J beta, MBP reactive TCR VDJ beta {clone KL-3(17), rearranged CDR3 region} [human, brain plaques, HLA phenotype 1, Peptide Partial, 19 aa] [Homo sapiens]
Sequence ID: AAB25620.1Length: 19Number of Matches: 1
Range 1: 2 to 7GenPeptGraphics Next Match Previous Match

Alignment statistics for match #1

| Score | Expect | Identities | Positives | Gaps |
|---|---|---|---|---|
| 21.0 bits(42) | 194 | 6/6(100%) | 6/6(100%) | 0/6(0%) |

```
Query  1  CASSQG  6
          CASSQG
Sbjct  2  CASSQG  7
```

18.5-kDa MBP-specific T cell Receptor beta chain VJ region {DR2/DQw1 varient, clone 1F5 98-170} [human, multiple sclerosis patient MS-B2, Peptide Partial, 16 aa] [Homo sapiens]
Sequence ID: AAB32431.1Length: 16Number of Matches: 1
Range 1: 1 to 15GenPeptGraphics Next Match Previous Match

Alignment statistics for match #1

| Score | Expect | Identities | Positives | Gaps |
|---|---|---|---|---|
| 23.5 bits(48) | 18 | 9/15(60%) | 9/15(60%) | 4/15(26%) |

```
Query  1   CASSQGL----GYTF  11
           CAS  GL    GYTF
Sbjct  1   CASRPGLGRNYGYTF  15
```

## TCR Heart 2.7 CASSQDRGQGFFFG

myelin basic protein specific T-cell receptor V beta-D beta-J beta, MBP reactive TCR VDJ beta {clone SE(5), rearranged CDR3 region} [human, inflammatory brain lesions, HLA phenotype 1, Peptide Partial, 26 aa] [Homo sapiens]
Sequence ID: AAB25639.1Length: 26Number of Matches: 1
Range 1: 2 to 17GenPeptGraphics Next Match Previous Match

Alignment statistics for match #1

| Score | Expect | Identities | Positives | Gaps |
|---|---|---|---|---|
| 27.8 bits(58) | 0.83 | 11/16(69%) | 11/16(68%) | 4/16(25%) |

```
Query  1   CASSQ-DRGQG---FF  12
           CASS  DRGQG   FF
Sbjct  2   CASSRLDRGQGEEQFF  17
```

myelin basic protein-specific T-cell receptor beta chain VDJ region {clone JK1.16} [human, peripheral blood mononuclear cells, Peptide Partial, 27 aa] [Homo sapiens]

Sequence ID: AAB33031.1Length: 27Number of Matches: 1

Range 1: 1 to 19GenPeptGraphics Next Match Previous Match

Alignment statistics for match #1

| Score | Expect | Identities | Positives | Gaps |
|---|---|---|---|---|
| 23.1 bits(47) | 44 | 11/19(58%) | 12/19(63%) | 5/19(26%) |

```
Query  1   CASSQDRGQG--F---FFG  14
           CASSQ+ G G  F   FFG
Sbjct  1   CASSQEEGAGGPFNEQFFG  19
```

myelin basic protein specific T-cell receptor V beta-D beta-J beta, MBP reactive TCR VDJ beta {CDR3 region, clone BM(4)} [human, peripheral blood lymphocytes, HLA phenotype 1, Peptide Partial, 24 aa] [Homo sapiens]

Sequence ID: AAB25665.1Length: 24Number of Matches: 1

Range 1: 2 to 9GenPeptGraphics Next Match Previous Match

Alignment statistics for match #1

| Score | Expect | Identities | Positives | Gaps |
|---|---|---|---|---|
| 20.6 bits(41) | 356 | 6/8(75%) | 7/8(87%) | 0/8(0%) |

```
Query  1  CASSQDRG  8
          CASS +RG
Sbjct  2  CASSPERG  9
```

myelin basic protein specific T-cell receptor V beta-D beta-J beta, MBP reactive TCR VDJ beta {clone SE(5), rearranged CDR3 region} [human, inflammatory brain lesions, HLA phenotype 1, Peptide Partial, 26 aa] [Homo sapiens]

Sequence ID: AAB25639.1Length: 26Number of Matches: 1

Range 1: 2 to 17GenPeptGraphics Next Match Previous Match

Alignment statistics for match #1

| Score | Expect | Identities | Positives | Gaps |
|---|---|---|---|---|
| 27.8 bits(58) | 0.83 | 11/16(69%) | 11/16(68%) | 4/16(25%) |

```
Query  1   CASSQ-DRGQG---FF  12
           CASS  DRGQG   FF
Sbjct  2   CASSRLDRGQGEEQFF  17
```

myelin basic protein specific T-cell receptor V beta-D beta-J beta, MBP reactive TCR VDJ beta {CDR3 region, clone BM(5)} [human, peripheral blood lymphocytes, HLA phenotype 1, Peptide Partial, 23 aa] [Homo sapiens]
Sequence ID: AAB25666.1Length: 23Number of Matches: 1
Range 1: 2 to 7GenPeptGraphics Next Match Previous Match

Alignment statistics for match #1

| Score | Expect | Identities | Positives | Gaps |
|---|---|---|---|---|
| 19.3 bits(38) | 1013 | 5/6(83%) | 6/6(100%) | 0/6(0%) |

```
Query  1   CASSQD  6
           CASSQ+
Sbjct  2   CASSQE  7
```

TCR V beta {V-J junction, clone MS7-4G8} [human, multiple sclerosis patient, myelin basic protein-reactive T cells before vaccination, Peptide Partial, 30 aa] [Homo sapiens]
Sequence ID: AAB35719.1Length: 30Number of Matches: 1
Range 1: 3 to 17GenPeptGraphics Next Match Previous Match

Alignment statistics for match #1

| Score | Expect | Identities | Positives | Gaps |
|---|---|---|---|---|
| 18.9 bits(37) | 1509 | 9/17(53%) | 10/17(58%) | 5/17(29%) |

```
Query  1   CASSQDRGQG---FFFG  14
           CASS   GQG    +FG
Sbjct  3   CASS--SGQGSNTIYFG  17
```

## TCR Heart 2.8  CASSQDGVANQRSFFG

myelin basic protein specific T-cell receptor V beta-D beta-J beta, MBP reactive TCR VDJ beta {clone KL-3(7), rearranged CDR3 region} [human, brain plaques, HLA phenotype 1, Peptide Partial, 24 aa] [Homo sapiens]
Sequence ID: AAB25610.1Length: 24Number of Matches: 1
Range 1: 2 to 16GenPeptGraphics Next Match Previous Match

Alignment statistics for match #1

| Score | Expect | Identities | Positives | Gaps |
|---|---|---|---|---|
| 22.7 bits(46) | 78 | 10/16(63%) | 11/16(68%) | 1/16(6%) |

```
Query  1    CASSQDGVANQRSFFG  16
            CASSQ  V N+  FFG
Sbjct  2    CASSQPAVYNEQ-FFG  16
```

myelin basic protein specific T-cell receptor V beta-D beta-J beta, MBP reactive TCR VDJ beta {clone KL-3(1), rearranged CDR3 region} [human, brain plaques, HLA phenotype 1, Peptide Partial, 24 aa] [Homo sapiens]

Sequence ID: AAB25604.1Length: 24Number of Matches: 1

Range 1: 2 to 9GenPeptGraphics Next Match Previous Match

Alignment statistics for match #1

| Score | Expect | Identities | Positives | Gaps |
|---|---|---|---|---|
| 21.8 bits(44) | 159 | 7/8(88%) | 7/8(87%) | 0/8(0%) |

```
Query  1   CASSQDGV  8
           CASS DGV
Sbjct  2   CASSLDGV  9
```

myelin basic protein specific T-cell receptor V beta-D beta-J beta, MBP reactive TCR VDJ beta {clone KL-3(3), rearranged CDR3 region} [human, brain plaques, HLA phenotype 1, Peptide Partial, 24 aa] [Homo sapiens]

Sequence ID: AAB25606.1Length: 24Number of Matches: 1

Range 1: 2 to 9GenPeptGraphics Next Match Previous Match

Alignment statistics for match #1

| Score | Expect | Identities | Positives | Gaps |
|---|---|---|---|---|
| 21.8 bits(44) | 159 | 7/8(88%) | 7/8(87%) | 0/8(0%) |

```
Query  1   CASSQDGV  8
           CASS DGV
Sbjct  2   CASSLDGV  9
```

Download GenPeptGraphics Next Previous Descriptions

myelin basic protein specific T-cell receptor V beta-D beta-J beta, MBP reactive TCR VDJ beta {clone KL-3(2), rearranged CDR3 region} [human, brain plaques, HLA phenotype 1, Peptide Partial, 24 aa] [Homo sapiens]

Sequence ID: AAB25605.1Length: 24Number of Matches: 1

Range 1: 2 to 9GenPeptGraphics Next Match Previous Match

Alignment statistics for match #1

| Score | Expect | Identities | Positives | Gaps |
|---|---|---|---|---|

21.8 bits(44) 159 7/8(88%) 7/8(87%) 0/8(0%)

```
Query  1  CASSQDGV  8
          CASS DGV
Sbjct  2  CASSLDGV  9
```

myelin basic protein-specific T-cell receptor beta chain VDJ region {clone JK1.16} [human, peripheral blood mononuclear cells, Peptide Partial, 27 aa] [Homo sapiens]

Sequence ID: AAB33031.1Length: 27Number of Matches: 1

Range 1: 1 to 19GenPeptGraphics Next Match Previous Match

Alignment statistics for match #1

| Score | Expect | Identities | Positives | Gaps |
|---|---|---|---|---|
| 21.4 bits(43) | 237 | 10/20(50%) | 12/20(60%) | 5/20(25%) |

```
Query  1   CASSQDGVA----NQRSFFG  16
           CASSQ+  A     N+   FFG
Sbjct  1   CASSQEEGAGGPFNEQ-FFG  19
```

myelin basic protein specific T-cell receptor V beta-D beta-J beta, MBP reactive TCR VDJ beta {clone KL-1(8), rearranged CDR3 region} [human, brain plaques, HLA phenotype 1, Peptide Partial, 25 aa] [Homo sapiens]

Sequence ID: AAB25599.1Length: 25Number of Matches: 1

Range 1: 2 to 17GenPeptGraphics Next Match Previous Match

Alignment statistics for match #1

| Score | Expect | Identities | Positives | Gaps |
|---|---|---|---|---|
| 19.7 bits(39) | 937 | 11/18(61%) | 11/18(61%) | 4/18(22%) |

```
Query  1   CASSQDGVAN--QRSFFG  16
           CASSQ   V N   Q  FFG
Sbjct  2   CASSQPTVYNNEQ--FFG  17
```

myelin basic protein specific T-cell receptor V beta-D beta-J beta, MBP reactive TCR VDJ beta {CDR3 region, clone BM(5)} [human, peripheral blood lymphocytes, HLA phenotype 1, Peptide Partial, 23 aa] [Homo sapiens]

Sequence ID: AAB25666.1Length: 23Number of Matches: 1

Range 1: 2 to 7GenPeptGraphics Next Match Previous Match

Alignment statistics for match #1

| Score | Expect | Identities | Positives | Gaps |
|---|---|---|---|---|
| 19.3 bits(38) | 1295 | 5/6(83%) | 6/6(100%) | 0/6(0%) |

```
Query  1  CASSQD  6
          CASSQ+
Sbjct  2  CASSQE  7
```

myelin basic protein specific T-cell receptor V beta-D beta-J beta, MBP reactive TCR VDJ beta {clone KL-1(9), rearranged CDR3 region} [human, brain plaques, HLA phenotype 1, Peptide Partial, 24 aa] [Homo sapiens]

Sequence ID: AAB25600.1Length: 24Number of Matches: 1

Range 1: 2 to 16GenPeptGraphics Next Match Previous Match

Alignment statistics for match #1

| Score | Expect | Identities | Positives | Gaps |
|---|---|---|---|---|
| 19.3 bits(38) | 1312 | 9/17(53%) | 10/17(58%) | 3/17(17%) |

```
Query  1   CASSQDG-VANQRSFFG  16
           CASS DG     Q  +FG
Sbjct  2   CASSSDGRMSTQ--YFG  16
```

myelin basic protein (143-168)-specific TCR beta-chain {clone JA.1.7} [human, multiple sclerosis patient, T cells, Peptide Partial, 27 aa] [Homo sapiens]

Sequence ID: AAB31429.1Length: 27Number of Matches: 1

Range 1: 1 to 7GenPeptGraphics Next Match Previous Match

Alignment statistics for match #1

| Score | Expect | Identities | Positives | Gaps |
|---|---|---|---|---|
| 18.9 bits(37) | 1914 | 6/7(86%) | 6/7(85%) | 0/7(0%) |

```
Query  1  CASSQDG  7
          CASS DG
Sbjct  1  CASSLDG  7
```

## TCR Heart 2.9 CASSATGAGTGEFFG

myelin basic protein specific T-cell receptor V beta-D beta-J beta, MBP reactive TCR VDJ beta {clone KL-3(15), rearranged CDR3 region} [human, brain plaques, HLA phenotype 1, Peptide Partial, 23 aa] [Homo sapiens]

---

Sequence ID: AAB25618.1Length: 23Number of Matches: 1

- See 2 more title(s) See all Identical Proteins(IPG)

Related Information
Identical Proteins-Identical proteins to AAB25618.1
Range 1: 2 to 9GenPeptGraphics Next Match Previous Match

Alignment statistics for match #1

| Score | Expect | Identities | Positives | Gaps |
| --- | --- | --- | --- | --- |
| 22.3 bits(45) | 96 | 7/8(88%) | 7/8(87%) | 0/8(0%) |

```
Query  1  CASSATGA  8
          CASS TGA
Sbjct  2  CASSPTGA  9
```

myelin basic protein-specific T-cell receptor beta chain VDJ region {clone FN2.2} [human, peripheral blood mononuclear cells, Peptide Partial, 25 aa] [Homo sapiens]
Sequence ID: AAB33027.1Length: 25Number of Matches: 1
Range 1: 1 to 17GenPeptGraphics Next Match Previous Match

Alignment statistics for match #1

| Score | Expect | Identities | Positives | Gaps |
| --- | --- | --- | --- | --- |
| 22.3 bits(45) | 100 | 10/17(59%) | 11/17(64%) | 2/17(11%) |

```
Query  1   CASSATGAGT--GEFFG  15
           CASS  G GT   +FFG
Sbjct  1   CASSLRGSGTYNEQFFG  17
```

myelin basic protein specific T-cell receptor V beta-D beta-J beta, MBP reactive TCR VDJ beta {clone SE(8), rearranged CDR3 region} [human, inflammatory brain lesions, HLA phenotype 1, Peptide Partial, 24 aa] [Homo sapiens]
Sequence ID: AAB25642.1Length: 24Number of Matches: 1
Range 1: 2 to 16GenPeptGraphics Next Match Previous Match

Alignment statistics for match #1

| Score | Expect | Identities | Positives | Gaps |
| --- | --- | --- | --- | --- |
| 21.4 bits(43) | 200 | 10/16(63%) | 10/16(62%) | 2/16(12%) |

```
Query  1   CASSATGAGTGE-FFG  15
           CASS  G   GE FFG
Sbjct  2   CASSTSGQ-PGELFFG  16
```

myelin basic protein specific T-cell receptor V beta-D beta-J beta, MBP reactive TCR VDJ beta {clone SE(14), rearranged CDR3 region} [human, inflammatory brain lesions, HLA phenotype 1, Peptide Partial, 23 aa] [Homo sapiens]
Sequence ID: AAB25648.1Length: 23Number of Matches: 1
Range 1: 2 to 15GenPeptGraphics Next Match Previous Match

---

Alignment statistics for match #1

| Score | Expect | Identities | Positives | Gaps |
|---|---|---|---|---|
| 19.7 bits(39) | 809 | 9/16(56%) | 11/16(68%) | 3/16(18%) |

```
Query  1   CASSATGAGT-GEFFG  15
           CASS   AGT  ++FG
Sbjct  2   CASST--AGTYEQYFG  15
```

myelin basic protein specific T-cell receptor V beta-D beta-J beta, MBP TCR reactive VDJ beta {CDR3 region, muscle infiltrating lymphocytes(2)} [human, muscle infiltrating lymphocytes, HLA phenotype 1, Peptide Partial, 25 aa] [Homo sapiens]
Sequence ID: AAB25650.1Length: 25Number of Matches: 1
Range 1: 2 to 9GenPeptGraphics Next Match Previous Match

Alignment statistics for match #1

| Score | Expect | Identities | Positives | Gaps |
|---|---|---|---|---|
| 19.7 bits(39) | 830 | 7/8(88%) | 7/8(87%) | 0/8(0%) |

```
Query  1  CASSATGA  8
          CASS TGA
Sbjct  2  CASSFTGA  9
```

myelin basic protein specific T-cell receptor V beta-D beta-J beta, MBP reactive TCR VDJ beta {clone SE(11), rearranged CDR3 region} [human, inflammatory brain lesions, HLA phenotype 1, Peptide Partial, 21 aa] [Homo sapiens]
Sequence ID: AAB25645.1Length: 21Number of Matches: 1
Range 1: 2 to 15GenPeptGraphics Next Match Previous Match

Alignment statistics for match #1

| Score | Expect | Identities | Positives | Gaps |
|---|---|---|---|---|
| 18.9 bits(37) | 1587 | 8/14(57%) | 8/14(57%) | 5/14(35%) |

```
Query  1   CASSAT-----GAG  9
           CASS T     GAG
Sbjct  2   CASSGTLVLTFGAG  15
```

X2 MBP-specific T cell Receptor beta chain VJ region {DR2/DQw1 varient, clone 3E12} [human, multiple sclerosis patient MS-B1, Peptide Partial, 15 aa] [Homo sapiens]
Sequence ID: AAB32425.1Length: 15Number of Matches: 1
Range 1: 1 to 9GenPeptGraphics Next Match Previous Match

Alignment statistics for match #1

| Score | Expect | Identities | Positives | Gaps |
|---|---|---|---|---|
| 18.9 bits(37) | 1327 | 7/9(78%) | 7/9(77%) | 0/9(0%) |

```
Query  1  CASSATGAG  9
          CASS TG G
Sbjct  1  CASSYTGGG  9
```

18.5-kDa MBP-specific T cell Receptor beta chain VJ region {DR2/DQw1 varient, clone 2H5 1-97} [human, multiple sclerosis patient MS-B1, Peptide Partial, 16 aa] [Homo sapiens]
Sequence ID: AAB32427.1Length: 16Number of Matches: 1
Range 1: 1 to 16GenPeptGraphics Next Match Previous Match

Alignment statistics for match #1

| Score | Expect | Identities | Positives | Gaps |
|---|---|---|---|---|
| 18.5 bits(36) | 1986 | 10/17(59%) | 12/17(70%) | 3/17(17%) |

```
Query  1    CASSATGAG--TGEFFG  15
            CASS   GAG   T ++FG
Sbjct  1    CASSYRGAGRET-QYFG  16
```

## TCR Heart 2-10 CASSQDYRAADTRFFG

myelin basic protein specific T-cell receptor V beta-D beta-J beta, MBP reactive TCR VDJ beta {clone SE(9), rearranged CDR3 region} [human, inflammatory brain lesions, HLA phenotype 1, Peptide Partial, 25 aa] [Homo sapiens]
Sequence ID: AAB25643.1Length: 25Number of Matches: 1
Range 1: 2 to 17GenPeptGraphics Next Match Previous Match

Alignment statistics for match #1

| Score | Expect | Identities | Positives | Gaps |
|---|---|---|---|---|
| 21.8 bits(44) | 162 | 10/16(63%) | 11/16(68%) | 0/16(0%) |

```
Query  1    CASSQDYRAADTRFFG  16
            CASS D    ADT +FG
Sbjct  2    CASSLDLGKADTQYFG  17
```

myelin basic protein (143-168)-specific TCR beta-chain {clone HE.1D5} [human, multiple sclerosis patient, T cells, Peptide Partial, 29 aa] [Homo sapiens]
Sequence ID: AAB31428.1Length: 29Number of Matches: 1
Range 1: 1 to 17GenPeptGraphics Next Match Previous Match

Alignment statistics for match #1

| Score | Expect | Identities | Positives | Gaps |
|---|---|---|---|---|
| 20.6 bits(41) | 484 | 10/19(53%) | 11/19(57%) | 5/19(26%) |

```
Query  1    CASSQDY--RA-ADTRFFG  16
            CASS  Y  R    DT +FG
Sbjct  1    CASS--YSGRVDTDTQYFG  17
```

myelin basic protein specific T-cell receptor V beta-D beta-J beta, MBP reactive TCR VDJ beta {clone LJ 1(2), rearranged CDR3 region} [human, brain plaques, HLA phenotype 1, Peptide Partial, 22 aa] [Homo sapiens]
Sequence ID: AAB25628.1Length: 22Number of Matches: 1
Range 1: 2 to 14GenPeptGraphics Next Match Previous Match

Alignment statistics for match #1

| Score | Expect | Identities | Positives | Gaps |
|---|---|---|---|---|
| 19.7 bits(39) | 896 | 8/16(50%) | 10/16(62%) | 3/16(18%) |

```
Query  1    CASSQDYRAADTRFFG  16
            CASS D     +T +FG
Sbjct  2    CASS-D--SSETQYFG  14
```

myelin basic protein-specific T-cell receptor beta chain VDJ region {clone JK1.16} [human, peripheral blood mononuclear cells, Peptide Partial, 27 aa] [Homo sapiens]
Sequence ID: AAB33031.1Length: 27Number of Matches: 1
Range 1: 1 to 6GenPeptGraphics Next Match Previous Match

Alignment statistics for match #1

| Score | Expect | Identities | Positives | Gaps |
|---|---|---|---|---|
| 19.3 bits(38) | 1352 | 5/6(83%) | 6/6(100%) | 0/6(0%) |

```
Query  1  CASSQD  6
          CASSQ+
Sbjct  1  CASSQE  6
```

myelin basic protein specific T-cell receptor V beta-D beta-J beta, MBP TCR reactive VDJ beta {CDR3 region, muscle infiltrating lymphocytes(1)} [human, muscle infiltrating lymphocytes, HLA phenotype 1, Peptide Partial, 28 aa] [Homo sapiens]
Sequence ID: AAB25649.1Length: 28Number of Matches: 1
Range 1: 2 to 20GenPeptGraphics Next Match Previous Match

Alignment statistics for match #1

| Score | Expect | Identities | Positives | Gaps |
|---|---|---|---|---|
| 19.3 bits(38) | 1363 | 9/19(47%) | 10/19(52%) | 3/19(15%) |

```
Query  1    CAS---SQDYRAADTRFFG  16
            CAS   S  YR   +  FFG
Sbjct  2    CASSLGSPGYRTNEKLFFG  20
```

18.5-kDa MBP-specific T cell Receptor beta chain VJ region {DR2/DQw1 varient, clone 2H5 1-97} [human, multiple sclerosis patient MS-B1, Peptide Partial, 16 aa] [Homo sapiens]
Sequence ID: AAB32427.1Length: 16Number of Matches: 1
Range 1: 1 to 16GenPeptGraphics Next Match Previous Match

Alignment statistics for match #1

| Score | Expect | Identities | Positives | Gaps |
|---|---|---|---|---|

25.2 bits(52) 6.2 10/18(56%) 12/18(66%) 4/18(22%)

```
Query  1    CASSQDYRAA--DTRFFG  16
            CASS  YR A  +T +FG
Sbjct  1    CASS--YRGAGRETQYFG  16
```

myelin basic protein (143-168)-specific TCR beta-chain {clone HE.1D5} [human, multiple sclerosis patient, T cells, Peptide Partial, 29 aa] [Homo sapiens]
Sequence ID: AAB31428.1Length: 29Number of Matches: 1
Range 1: 1 to 17GenPeptGraphics Next Match Previous Match

Alignment statistics for match #1

| Score | Expect | Identities | Positives | Gaps |
|---|---|---|---|---|
| 20.6 bits(41) | 484 | 10/19(53%) | 11/19(57%) | 5/19(26%) |

```
Query  1    CASSQDY--RA-ADTRFFG  16
            CASS  Y  R   DT +FG
Sbjct  1    CASS--YSGRVDTDTQYFG  17
```

## TCR Heart 2-11 CASSQDRGGGAGFG

myelin basic protein specific T-cell receptor V beta-D beta-J beta, MBP reactive TCR VDJ beta {CDR3 region, clone BM(4)} [human, peripheral blood lymphocytes, HLA phenotype 1, Peptide Partial, 24 aa] [Homo sapiens]
Sequence ID: AAB25665.1Length: 24Number of Matches: 1
Range 1: 2 to 18GenPeptGraphics Next Match Previous Match

Alignment statistics for match #1

| Score | Expect | Identities | Positives | Gaps |
|---|---|---|---|---|
| 21.4 bits(43) | 175 | 9/17(53%) | 10/17(58%) | 5/17(29%) |

```
Query  1    CASSQDRGG-----GAG  12
            CASS +RG      GAG
Sbjct  2    CASSPERGANVLTFGAG  18
```

myelin basic protein specific T-cell receptor V beta-D beta-J beta, MBP reactive TCR VDJ beta {CDR3 region, clone JO(1)} [human, normal peripheral blood lymphocytes, Peptide Partial, 25 aa] [Homo sapiens]
Sequence ID: AAB25656.1Length: 25Number of Matches: 1
Range 1: 2 to 21GenPeptGraphics Next Match Previous Match

Alignment statistics for match #1

| Score | Expect | Identities | Positives | Gaps |
|---|---|---|---|---|
| 20.6 bits(41) | 360 | 10/20(50%) | 10/20(50%) | 8/20(40%) |

```
Query  1    CASS---QDR-----GGGAG  12
            CASS   QDR     G G G
```

```
Sbjct  2   CASSVALQDRYGYTFGSGTG  21
```

myelin basic protein-specific T-cell receptor beta chain VDJ region {clone JK1.16} [human, peripheral blood mononuclear cells, Peptide Partial, 27 aa] [Homo sapiens]

Sequence ID: AAB33031.1Length: 27Number of Matches: 1

Range 1: 1 to 10GenPeptGraphics Next Match Previous Match

Alignment statistics for match #1

| Score | Expect | Identities | Positives | Gaps |
|---|---|---|---|---|
| 20.6 bits(41) | 367 | 8/12(67%) | 9/12(75%) | 2/12(16%) |

```
Query  1   CASSQDRGGGAG  12
           CASSQ+   GAG
Sbjct  1   CASSQE--EGAG  10
```

myelin basic protein specific T-cell receptor V beta-D beta-J beta, MBP reactive TCR VDJ beta {clone KL-1(4), rearranged CDR3 region} [human, brain plaques, HLA phenotype 1, Peptide Partial, 25 aa] [Homo sapiens]

Sequence ID: AAB25595.1Length: 25Number of Matches: 1

Range 1: 2 to 8GenPeptGraphics Next Match Previous Match

Alignment statistics for match #1

| Score | Expect | Identities | Positives | Gaps |
|---|---|---|---|---|
| 19.7 bits(39) | 728 | 6/7(86%) | 6/7(85%) | 0/7(0%) |

```
Query  1  CASSQDR  7
          CASS DR
Sbjct  2  CASSLDR  8
```

myelin basic protein specific T-cell receptor V beta-D beta-J beta, MBP reactive TCR VDJ beta {CDR3 region, clone BM(5)} [human, peripheral blood lymphocytes, HLA phenotype 1, Peptide Partial, 23 aa] [Homo sapiens]

Sequence ID: AAB25666.1Length: 23Number of Matches: 1

Range 1: 2 to 7GenPeptGraphics Next Match Previous Match

Alignment statistics for match #1

| Score | Expect | Identities | Positives | Gaps |
|---|---|---|---|---|
| 19.3 bits(38) | 1013 | 5/6(83%) | 6/6(100%) | 0/6(0%) |

```
Query  1  CASSQD  6
          CASSQ+
Sbjct  2  CASSQE  7
```

myelin basic protein specific T-cell receptor V beta-D beta-J beta, MBP reactive TCR VDJ beta {clone SE(5), rearranged CDR3 region} [human, inflammatory brain lesions, HLA phenotype 1, Peptide Partial, 26 aa] [Homo sapiens]

Sequence ID: AAB25639.1Length: 26Number of Matches: 1

Range 1: 2 to 10GenPeptGraphics Next Match Previous Match

Alignment statistics for match #1

| Score | Expect | Identities | Positives | Gaps |
|---|---|---|---|---|
| 19.3 bits(38) | 1042 | 7/9(78%) | 7/9(77%) | 1/9(11%) |

```
Query  1  CASSQ-DRG  8
          CASS  DRG
Sbjct  2  CASSRLDRG  10
```

T-cell antigen receptor alpha-chain variable-region, TCR V alpha {complementarity-determining region 3} [human, multiple sclerosis patient D2*, peripheral blood leukocytes, Peptide Partial, 30 aa] [Homo sapiens]
Sequence ID: AAB30921.1Length: 30Number of Matches: 1
Range 1: 3 to 11GenPeptGraphics Next Match Previous Match

Alignment statistics for match #1

| Score | Expect | Identities | Positives | Gaps |
|---|---|---|---|---|
| 21.4 bits(43) | 186 | 8/11(73%) | 8/11(72%) | 2/11(18%) |

```
Query  1   CASSQDRGGGA  11
           CA S  RGGGA
Sbjct  3   CAAS--RGGGA  11
```

## TCR Heart 2-12 CASSQDYRAAYFG

myelin basic protein specific T-cell receptor V beta-D beta-J beta, MBP reactive TCR VDJ beta {CDR3 region, clone BM(5)} [human, peripheral blood lymphocytes, HLA phenotype 1, Peptide Partial, 23 aa] [Homo sapiens]
Sequence ID: AAB25666.1Length: 23Number of Matches: 1
Range 1: 2 to 15GenPeptGraphics Next Match Previous Match

Alignment statistics for match #1

| Score | Expect | Identities | Positives | Gaps |
|---|---|---|---|---|
| 22.7 bits(46) | 51 | 9/15(60%) | 10/15(66%) | 3/15(20%) |

```
Query  1   CASSQD--YRAAYFG  13
           CASSQ+   Y   YFG
Sbjct  2   CASSQEASY-EQYFG  15
```

---

myelin basic protein specific T-cell receptor V beta-D beta-J beta, MBP reactive TCR VDJ beta {clone KL-1(12), rearranged CDR3 region} [human, brain plaques, HLA phenotype 1, Peptide Partial, 23 aa] [Homo sapiens]
Sequence ID: AAB25603.1Length: 23Number of Matches: 1
Range 1: 2 to 15GenPeptGraphics Next Match Previous Match

Alignment statistics for match #1

| Score | Expect | Identities | Positives | Gaps |
|---|---|---|---|---|
| 20.2 bits(40) | 434 | 9/15(60%) | 10/15(66%) | 3/15(20%) |

```
Query  1    CASS--QDYRAAYFG  13
            CASS  +   RA YFG
Sbjct  2    CASSGGEG-RAQYFG  15
```

myelin basic protein specific T-cell receptor V beta-D beta-J beta, MBP reactive TCR VDJ beta {clone KL-1(11), rearranged CDR3 region} [human, brain plaques, HLA phenotype 1, Peptide Partial, 24 aa] [Homo sapiens]
Sequence ID: AAB25602.1Length: 24Number of Matches: 1
Range 1: 2 to 16GenPeptGraphics Next Match Previous Match

Alignment statistics for match #1

| Score | Expect | Identities | Positives | Gaps |
|---|---|---|---|---|
| 19.7 bits(39) | 626 | 9/15(60%) | 10/15(66%) | 2/15(13%) |

```
Query  1    CASSQDY--RAAYFG  13
            CASS +    RA YFG
Sbjct  2    CASSSEREGRAQYFG  16
```

myelin basic protein specific T-cell receptor V beta-D beta-J beta, MBP reactive TCR VDJ beta {CDR3 region, clone BM(3)} [human, peripheral blood lymphocytes, HLA phenotype 1, Peptide Partial, 27 aa] [Homo sapiens]
Sequence ID: AAB25664.1Length: 27Number of Matches: 1
Range 1: 2 to 19GenPeptGraphics Next Match Previous Match

Alignment statistics for match #1

| Score | Expect | Identities | Positives | Gaps |
|---|---|---|---|---|
| 19.3 bits(38) | 910 | 8/18(44%) | 9/18(50%) | 5/18(27%) |

```
Query  1    CASSQDYRAA-----YFG  13
            CASS + R       YFG
Sbjct  2    CASSLEHRPTAKNIQYFG  19
```

18.5-kDa MBP-specific T cell Receptor beta chain VJ region {DR2/DQw1 varient, clone 2H5 1-97} [human, multiple sclerosis patient MS-B1, Peptide Partial, 16 aa] [Homo sapiens]
Sequence ID: AAB32427.1Length: 16Number of Matches: 1
Range 1: 1 to 16GenPeptGraphics Next Match Previous Match

Alignment statistics for match #1

| Score | Expect | Identities | Positives | Gaps |
|---|---|---|---|---|
| 24.4 bits(50) | 8.7 | 10/18(56%) | 10/18(55%) | 7/18(38%) |

```
Query  1   CASSQDYRAA-----YFG  13
           CASS  YR A     YFG
Sbjct  1   CASS--YRGAGRETQYFG  16
```

# TCR Heart 2.13 CASSPRTGRYEQYF

myelin basic protein specific T-cell receptor V beta-D beta-J beta, MBP reactive TCR VDJ beta {clone SE(14), rearranged CDR3 region} [human, inflammatory brain lesions, HLA phenotype 1, Peptide Partial, 23 aa] [Homo sapiens]
Sequence ID: AAB25648.1Length: 23Number of Matches: 1
Range 1: 2 to 14GenPeptGraphics Next Match Previous Match

Alignment statistics for match #1

| Score | Expect | Identities | Positives | Gaps |
|---|---|---|---|---|
| 28.2 bits(59) | 0.51 | 11/15(73%) | 11/15(73%) | 3/15(20%) |

```
Query  1   CASSPRT-GRYEQYF  14
           CASS  T G YEQYF
Sbjct  2   CASS--TAGTYEQYF  14
```

myelin basic protein specific T-cell receptor V beta-D beta-J beta, MBP reactive TCR VDJ beta {clone LJ 1(6), rearranged CDR3 region} [human, brain plaques, HLA phenotype 1, Peptide Partial, 24 aa] [Homo sapiens]
Sequence ID: AAB25632.1Length: 24Number of Matches: 1
Range 1: 2 to 15GenPeptGraphics Next Match Previous Match

Alignment statistics for match #1

| Score | Expect | Identities | Positives | Gaps |
|---|---|---|---|---|
| 27.8 bits(58) | 0.77 | 10/14(71%) | 10/14(71%) | 0/14(0%) |

```
Query  1   CASSPRTGRYEQYF  14
           CASS   G YEQYF
Sbjct  2   CASSLVAGIYEQYF  15
```

myelin basic protein-specific T-cell receptor beta chain VDJ region {clone HOO24} [human, peripheral blood mononuclear cells, Peptide Partial, 25 aa] [Homo sapiens]
Sequence ID: AAB33029.1Length: 25Number of Matches: 1
Range 1: 1 to 16GenPeptGraphics Next Match Previous Match

---

Alignment statistics for match #1

| Score | Expect | Identities | Positives | Gaps |
|---|---|---|---|---|
| 27.4 bits(57) | 1.2 | 10/16(63%) | 10/16(62%) | 2/16(12%) |

```
Query  1   CASSP--RTGRYEQYF  14
           CASS    R   YEQYF
Sbjct  1   CASSSLARGASYEQYF  16
```

myelin basic protein (143-168)-specific TCR beta-chain {clone JA.1.7} [human, multiple sclerosis patient, T cells, Peptide Partial, 28 aa] [Homo sapiens]
Sequence ID: AAB31430.1Length: 28Number of Matches: 1
Range 1: 1 to 9GenPeptGraphics Next Match Previous Match

Alignment statistics for match #1

| Score | Expect | Identities | Positives | Gaps |
|---|---|---|---|---|
| 25.7 bits(53) | 5.3 | 8/9(89%) | 8/9(88%) | 0/9(0%) |

```
Query  1  CASSPRTGR  9
          CASSP TGR
Sbjct  1  CASSPMTGR  9
```

myelin basic protein specific T-cell receptor V beta-D beta-J beta, MBP reactive TCR VDJ beta {CDR3 region, clone BM(5)} [human, peripheral blood lymphocytes, HLA phenotype 1, Peptide Partial, 23 aa] [Homo sapiens]
Sequence ID: AAB25666.1Length: 23Number of Matches: 1
Range 1: 2 to 14GenPeptGraphics Next Match Previous Match

Alignment statistics for match #1

| Score | Expect | Identities | Positives | Gaps |
|---|---|---|---|---|
| 24.4 bits(50) | 14 | 9/14(64%) | 9/14(64%) | 1/14(7%) |

```
Query  1   CASSPRTGRYEQYF  14
           CASS     YEQYF
Sbjct  2   CASS-QEASYEQYF  14
```

myelin basic protein specific T-cell receptor V beta-D beta-J beta, MBP reactive TCR VDJ beta {clone LJ 1(7), rearranged CDR3 region} [human, brain plaques, HLA phenotype 1, Peptide Partial, 25 aa] [Homo sapiens]
Sequence ID: AAB25633.1Length: 25Number of Matches: 1

- See 1 more title(s) See all Identical Proteins(IPG)

Related Information
Identical Proteins-Identical proteins to AAB25633.1
Range 1: 2 to 16GenPeptGraphics Next Match Previous Match

Alignment statistics for match #1

| Score | Expect | Identities | Positives | Gaps |
|---|---|---|---|---|

24.4 bits(50) 15 10/15(67%) 10/15(66%) 1/15(6%)

```
Query  1   CASSPRTGR-YEQYF  14
           CASS   G  YEQYF
Sbjct  2   CASSLVAGSIYEQYF  16
```

Download GenPeptGraphics Next Previous Descriptions
myelin basic protein specific T-cell receptor V beta-D beta-J beta, MBP reactive TCR VDJ beta {clone KL-1(10), rearranged CDR3 region} [human, brain plaques, HLA phenotype 1, Peptide Partial, 25 aa] [Homo sapiens]
Sequence ID: AAB25601.1Length: 25Number of Matches: 1

- See 3 more title(s) See all Identical Proteins(IPG)

Related Information
Identical Proteins-Identical proteins to AAB25601.1
Range 1: 2 to 16GenPeptGraphics Next Match Previous Match

Alignment statistics for match #1

| Score | Expect | Identities | Positives | Gaps |
|---|---|---|---|---|
| 24.4 bits(50) | 15 | 10/15(67%) | 10/15(66%) | 1/15(6%) |

```
Query  1   CASSPRTGR-YEQYF  14
           CASS   G  YEQYF
Sbjct  2   CASSLVAGSIYEQYF  16
```

18.5-kDa MBP-specific T cell Receptor beta chain VJ region {DR2/DQw1 varient, clone 2H5 1-97} [human, multiple sclerosis patient MS-B1, Peptide Partial, 16 aa] [Homo sapiens]
Sequence ID: AAB32427.1Length: 16Number of Matches: 1
Range 1: 1 to 15GenPeptGraphics Next Match Previous Match

Alignment statistics for match #1

| Score | Expect | Identities | Positives | Gaps |
|---|---|---|---|---|
| 20.6 bits(41) | 285 | 11/16(69%) | 11/16(68%) | 3/16(18%) |

```
Query  1   CASSPR-TGRYE-QYF  14
           CASS R  GR E QYF
Sbjct  1   CASSYRGAGR-ETQYF  15
```

---

# TCR Heart 2.14 CASSPRTGRYEQYF

## SAME AS 2.13!!!!

myelin basic protein specific T-cell receptor V beta-D beta-J beta, MBP reactive TCR VDJ beta {clone SE(14), rearranged CDR3 region} [human, inflammatory brain lesions, HLA phenotype 1, Peptide Partial, 23 aa] [Homo sapiens]
Sequence ID: AAB25648.1Length: 23Number of Matches: 1
Range 1: 2 to 14GenPeptGraphics Next Match Previous Match

Alignment statistics for match #1

| Score | Expect | Identities | Positives | Gaps |
|---|---|---|---|---|
| 28.2 bits(59) | 0.51 | 11/15(73%) | 11/15(73%) | 3/15(20%) |

```
Query  1    CASSPRT-GRYEQYF  14
            CASS   T G YEQYF
Sbjct  2    CASS--TAGTYEQYF  14
```

myelin basic protein specific T-cell receptor V beta-D beta-J beta, MBP reactive TCR VDJ beta {clone LJ 1(6), rearranged CDR3 region} [human, brain plaques, HLA phenotype 1, Peptide Partial, 24 aa] [Homo sapiens]
Sequence ID: AAB25632.1Length: 24Number of Matches: 1
Range 1: 2 to 15GenPeptGraphics Next Match Previous Match

Alignment statistics for match #1

| Score | Expect | Identities | Positives | Gaps |
|---|---|---|---|---|
| 27.8 bits(58) | 0.77 | 10/14(71%) | 10/14(71%) | 0/14(0%) |

```
Query  1    CASSPRTGRYEQYF  14
            CASS    G YEQYF
Sbjct  2    CASSLVAGIYEQYF  15
```

myelin basic protein-specific T-cell receptor beta chain VDJ region {clone HOO24} [human, peripheral blood mononuclear cells, Peptide Partial, 25 aa] [Homo sapiens]
Sequence ID: AAB33029.1Length: 25Number of Matches: 1
Range 1: 1 to 16GenPeptGraphics Next Match Previous Match

Alignment statistics for match #1

| Score | Expect | Identities | Positives | Gaps |
|---|---|---|---|---|
| 27.4 bits(57) | 1.2 | 10/16(63%) | 10/16(62%) | 2/16(12%) |

```
Query  1    CASSP--RTGRYEQYF  14
            CASS    R    YEQYF
```

```
Sbjct  1   CASSSLARGASYEQYF  16
```

myelin basic protein (143-168)-specific TCR beta-chain {clone JA.1.7} [human, multiple sclerosis patient, T cells, Peptide Partial, 28 aa] [Homo sapiens]

Sequence ID: AAB31430.1Length: 28Number of Matches: 1

Range 1: 1 to 9GenPeptGraphics Next Match Previous Match

Alignment statistics for match #1

| Score | Expect | Identities | Positives | Gaps |
|---|---|---|---|---|
| 25.7 bits(53) | 5.3 | 8/9(89%) | 8/9(88%) | 0/9(0%) |

```
Query  1  CASSPRTGR  9
          CASSP TGR
Sbjct  1  CASSPMTGR  9
```

## TCR Heart 2.15   CASSQDVGQLTNYEQYF

myelin basic protein specific T-cell receptor V beta-D beta-J beta, MBP reactive TCR VDJ beta {clone SE(14), rearranged CDR3 region} [human, inflammatory brain lesions, HLA phenotype 1, Peptide Partial, 23 aa] [Homo sapiens]

Sequence ID: AAB25648.1Length: 23Number of Matches: 1

Range 1: 2 to 14GenPeptGraphics Next Match Previous Match

Alignment statistics for match #1

| Score | Expect | Identities | Positives | Gaps |
|---|---|---|---|---|
| 25.2 bits(52) | 10 | 10/19(53%) | 10/19(52%) | 8/19(42%) |

```
Query  1   CASSQDVGQLT--NYEQYF  17
           CASS       T    YEQYF
Sbjct  2   CASS------TAGTYEQYF  14
```

myelin basic protein specific T-cell receptor V beta-D beta-J beta, MBP reactive TCR VDJ beta {clone SE(14), rearranged CDR3 region} [human, inflammatory brain lesions, HLA phenotype 1, Peptide Partial, 23 aa] [Homo sapiens]

Sequence ID: AAB25648.1Length: 23Number of Matches: 1

Range 1: 2 to 14GenPeptGraphics Next Match Previous Match

Alignment statistics for match #1

| Score | Expect | Identities | Positives | Gaps |
|---|---|---|---|---|
| 25.2 bits(52) | 10 | 10/19(53%) | 10/19(52%) | 8/19(42%) |

```
Query  1    CASSQDVGQLT--NYEQYF  17
            CASS       T    YEQYF
Sbjct  2    CASS------TAGTYEQYF  14
```

myelin basic protein-specific T-cell receptor beta chain VDJ region {clone HOO24} [human, peripheral blood mononuclear cells, Peptide Partial, 25 aa] [Homo sapiens]
Sequence ID: AAB33029.1Length: 25Number of Matches: 1
Range 1: 1 to 16GenPeptGraphics Next Match Previous Match

Alignment statistics for match #1

| Score | Expect | Identities | Positives | Gaps |
|---|---|---|---|---|
| 24.0 bits(49) | 31 | 10/20(50%) | 10/20(50%) | 7/20(35%) |

```
Query  1    CASSQDVGQLT---NYEQYF  17
            CASS      L      YEQYF
Sbjct  1    CASSS----LARGASYEQYF  16
```

Download GenPeptGraphics Next Previous Descriptions
myelin basic protein specific T-cell receptor V beta-D beta-J beta, MBP reactive TCR VDJ beta {clone LJ 1(7), rearranged CDR3 region} [human, brain plaques, HLA phenotype 1, Peptide Partial, 25 aa] [Homo sapiens]
Sequence ID: AAB25633.1Length: 25Number of Matches: 1

- See 1 more title(s) See all Identical Proteins(IPG)

Related Information
Identical Proteins-Identical proteins to AAB25633.1
Range 1: 2 to 16GenPeptGraphics Next Match Previous Match

Alignment statistics for match #1

| Score | Expect | Identities | Positives | Gaps |
|---|---|---|---|---|
| 24.0 bits(49) | 31 | 10/20(50%) | 10/20(50%) | 8/20(40%) |

```
Query  1    CASSQDVGQL---TNYEQYF  17
            CASS      L      YEQYF
Sbjct  2    CASS-----LVAGSIYEQYF  16
```

Download GenPeptGraphics Next Previous Descriptions
myelin basic protein specific T-cell receptor V beta-D beta-J beta, MBP reactive TCR VDJ beta {clone KL-1(10), rearranged CDR3 region} [human, brain plaques, HLA phenotype 1, Peptide Partial, 25 aa] [Homo sapiens]
Sequence ID: AAB25601.1Length: 25Number of Matches: 1

- See 3 more title(s) See all Identical Proteins(IPG)

Related Information
Identical Proteins-Identical proteins to AAB25601.1
Range 1: 2 to 16GenPeptGraphics Next Match Previous Match

Alignment statistics for match #1

| Score | Expect | Identities | Positives | Gaps |
|---|---|---|---|---|
| 24.0 bits(49) | 31 | 10/20(50%) | 10/20(50%) | 8/20(40%) |

```
Query  1    CASSQDVGQL---TNYEQYF  17
            CASS      L      YEQYF
Sbjct  2    CASS-----LVAGSIYEQYF  16
```

myelin basic protein specific T-cell receptor V beta-D beta-J beta, MBP reactive TCR VDJ beta {clone LJ 1(6), rearranged CDR3 region} [human, brain plaques, HLA phenotype 1, Peptide Partial, 24 aa] [Homo sapiens]
Sequence ID: AAB25632.1Length: 24Number of Matches: 1
Range 1: 2 to 15GenPeptGraphics Next Match Previous Match

Alignment statistics for match #1

| Score | Expect | Identities | Positives | Gaps |
|---|---|---|---|---|
| 23.5 bits(48) | 43 | 10/19(53%) | 10/19(52%) | 7/19(36%) |

```
Query  1    CASSQDVGQLTN--YEQYF  17
            CASS      L     YEQYF
Sbjct  2    CASS-----LVAGIYEQYF  15
```

myelin basic protein (143-168)-specific TCR beta-chain {clone BM3.3} [human, multiple sclerosis patient, T cells, Peptide Partial, 26 aa] [Homo sapiens]
Sequence ID: AAB31427.1Length: 26Number of Matches: 1
Range 1: 1 to 13GenPeptGraphics Next Match Previous Match

Alignment statistics for match #1

| Score | Expect | Identities | Positives | Gaps |
|---|---|---|---|---|
| 22.7 bits(46) | 92 | 10/18(56%) | 10/18(55%) | 6/18(33%) |

```
Query  1    CASSQDVGQLT-NYEQYF  17
            CASS      LT   Y QYF
Sbjct  1    CASS-----LTRGYTQYF  13
```

**TCR Heart 2.16 CASSQGGAGAYEQYF**

myelin basic protein specific T-cell receptor V beta-D beta-J beta, MBP reactive TCR VDJ beta {clone SE(14), rearranged CDR3 region} [human, inflammatory brain lesions, HLA phenotype 1, Peptide Partial, 23 aa] [Homo sapiens]
Sequence ID: AAB25648.1Length: 23Number of Matches: 1
Range 1: 2 to 14GenPeptGraphics Next Match Previous Match

Alignment statistics for match #1

| Score | Expect | Identities | Positives | Gaps |
|---|---|---|---|---|
| 32.0 bits(68) | 0.021 | 11/15(73%) | 11/15(73%) | 2/15(13%) |

```
Query  1   CASSQGGAGAYEQYF  15
           CASS    AG+YEQYF
Sbjct  2   CASST--AGTYEQYF  14
```

myelin basic protein specific T-cell receptor V beta-D beta-J beta, MBP reactive TCR VDJ beta {CDR3 region, clone BM(5)} [human, peripheral blood lymphocytes, HLA phenotype 1, Peptide Partial, 23 aa] [Homo sapiens]
Sequence ID: AAB25666.1Length: 23Number of Matches: 1
Range 1: 2 to 14GenPeptGraphics Next Match Previous Match

Alignment statistics for match #1

| Score | Expect | Identities | Positives | Gaps |
|---|---|---|---|---|
| 31.2 bits(66) | 0.044 | 10/15(67%) | 10/15(66%) | 2/15(13%) |

```
Query  1   CASSQGGAGAYEQYF  15
           CASSQ      YEQYF
Sbjct  2   CASSQ--EASYEQYF  14
```

myelin basic protein specific T-cell receptor V beta-D beta-J beta, MBP reactive TCR VDJ beta {clone LJ 1(6), rearranged CDR3 region} [human, brain plaques, HLA phenotype 1, Peptide Partial, 24 aa] [Homo sapiens]
Sequence ID: AAB25632.1Length: 24Number of Matches: 1
Range 1: 2 to 15GenPeptGraphics Next Match Previous Match

Alignment statistics for match #1

| Score | Expect | Identities | Positives | Gaps |
|---|---|---|---|---|
| 28.6 bits(60) | 0.43 | 11/15(73%) | 11/15(73%) | 1/15(6%) |

```
Query  1   CASSQGGAGAYEQYF  15
           CASS    AG+YEQYF
Sbjct  2   CASSLV-AGIYEQYF  15
```

myelin basic protein specific T-cell receptor V beta-D beta-J beta, MBP reactive TCR VDJ beta {clone SE(14), rearranged CDR3 region} [human, inflammatory brain lesions, HLA phenotype 1, Peptide Partial, 23 aa] [Homo sapiens]
Sequence ID: AAB25648.1Length: 23Number of Matches: 1

Range 1: 2 to 14GenPeptGraphics Next Match Previous Match

Alignment statistics for match #1

| Score | Expect | Identities | Positives | Gaps |
|---|---|---|---|---|
| 32.0 bits(68) | 0.021 | 11/15(73%) | 11/15(73%) | 2/15(13%) |

```
Query  1    CASSQGGAGAYEQYF  15
            CASS   AG+YEQYF
Sbjct  2    CASST--AGTYEQYF  14
```

myelin basic protein-specific T-cell receptor beta chain VDJ region {clone HOO24} [human, peripheral blood mononuclear cells, Peptide Partial, 25 aa] [Homo sapiens]

Sequence ID: AAB33029.1Length: 25Number of Matches: 1

Range 1: 1 to 16GenPeptGraphics Next Match Previous Match

Alignment statistics for match #1

| Score | Expect | Identities | Positives | Gaps |
|---|---|---|---|---|
| 25.2 bits(52) | 8.2 | 10/18(56%) | 10/18(55%) | 5/18(27%) |

```
Query  1    CASS---QGGAGAYEQYF  15
            CASS    G    YEQYF
Sbjct  1    CASSSLARGAS--YEQYF  16
```

# TCR Heart 2.17  CASSQEPPGTRAYHDYEQYF

myelin basic protein specific T-cell receptor V beta-D beta-J beta, MBP reactive TCR VDJ beta {CDR3 region, clone BM(5)} [human, peripheral blood lymphocytes, HLA phenotype 1, Peptide Partial, 23 aa] [Homo sapiens]

Sequence ID: AAB25666.1Length: 23Number of Matches: 1

Range 1: 2 to 14GenPeptGraphics Next Match Previous Match

Alignment statistics for match #1

| Score | Expect | Identities | Positives | Gaps |
|---|---|---|---|---|
| 32.5 bits(69) | 0.029 | 11/20(55%) | 11/20(55%) | 7/20(35%) |

```
Query  1    CASSQEPPGTRAYHDYEQYF  20
```

```
             CASSQE          YEQYF
Sbjct  2    CASSQEAS-------YEQYF  14
```

myelin basic protein specific T-cell receptor V beta-D beta-J beta, MBP reactive TCR VDJ beta {clone SE(14), rearranged CDR3 region} [human, inflammatory brain lesions, HLA phenotype 1, Peptide Partial, 23 aa] [Homo sapiens]

Sequence ID: AAB25648.1Length: 23Number of Matches: 1

Range 1: 2 to 14GenPeptGraphics Next Match Previous Match

Alignment statistics for match #1

| Score | Expect | Identities | Positives | Gaps |
|---|---|---|---|---|
| 26.5 bits(55) | 4.8 | 11/20(55%) | 11/20(55%) | 7/20(35%) |

```
Query  1    CASSQEPPGTRAYHDYEQYF  20
            CASS     GT      YEQYF
Sbjct  2    CASS--TAGT-----YEQYF  14
```

myelin basic protein-specific T-cell receptor beta chain VDJ region {clone JK1.16} [human, peripheral blood mononuclear cells, Peptide Partial, 27 aa] [Homo sapiens]

Sequence ID: AAB33031.1Length: 27Number of Matches: 1

Range 1: 1 to 23GenPeptGraphics Next Match Previous Match

Alignment statistics for match #1

| Score | Expect | Identities | Positives | Gaps |
|---|---|---|---|---|
| 24.8 bits(51) | 23 | 11/23(48%) | 11/23(47%) | 12/23(52%) |

```
Query  1    CASSQE-----P-------PGTR  11
            CASSQE     P       PGTR
Sbjct  1    CASSQEEGAGGPFNEQFFGPGTR  23
```

myelin basic protein (143-168)-specific TCR beta-chain {clone BM3.3} [human, multiple sclerosis patient, T cells, Peptide Partial, 26 aa] [Homo sapiens]

Sequence ID: AAB31427.1Length: 26Number of Matches: 2

Range 1: 1 to 13GenPeptGraphics Next Match Previous Match

Alignment statistics for match #1

| Score | Expect | Identities | Positives | Gaps |
|---|---|---|---|---|
| 21.0 bits(42) | 506 | 10/20(50%) | 10/20(50%) | 7/20(35%) |

```
Query  1    CASSQEPPGTRAYHDYEQYF  20
            CASS      TR     Y QYF
Sbjct  1    CASSL----TRG---YTQYF  13
```

Range 2: 15 to 18GenPeptGraphics Next Match Previous Match First Match

Alignment statistics for match #2

---

| Score | Expect | Identities | Positives | Gaps |
|---|---|---|---|---|
| 15.5 bits(29) | 42969 | 4/4(100%) | 4/4(100%) | 0/4(0%) |

```
Query  8   PGTR  11
           PGTR
Sbjct  15  PGTR  18
```

# TCR Heart 2.18 CASSRSEREQYEQYF

myelin basic protein specific T-cell receptor V beta-D beta-J beta, MBP reactive TCR VDJ beta {clone KL-1(11), rearranged CDR3 region} [human, brain plaques, HLA phenotype 1, Peptide Partial, 24 aa] [Homo sapiens]
Sequence ID: AAB25602.1Length: 24Number of Matches: 1
Range 1: 2 to 15GenPeptGraphics Next Match Previous Match

Alignment statistics for match #1

| Score | Expect | Identities | Positives | Gaps |
|---|---|---|---|---|
| 26.9 bits(56) | 1.9 | 11/15(73%) | 11/15(73%) | 1/15(6%) |

```
Query  1   CASSRSEREQYEQYF  15
           CASS SERE   QYF
Sbjct  2   CASS-SEREGRAQYF  15
```

myelin basic protein specific T-cell receptor V beta-D beta-J beta, MBP reactive TCR VDJ beta {CDR3 region, clone BM(5)} [human, peripheral blood lymphocytes, HLA phenotype 1, Peptide Partial, 23 aa] [Homo sapiens]
Sequence ID: AAB25666.1Length: 23Number of Matches: 1
Range 1: 2 to 14GenPeptGraphics Next Match Previous Match

Alignment statistics for match #1

| Score | Expect | Identities | Positives | Gaps |
|---|---|---|---|---|
| 26.5 bits(55) | 2.6 | 10/18(56%) | 10/18(55%) | 8/18(44%) |

```
Query  1   CASSRSEREQ---YEQYF  15
           CASS     Q   YEQYF
Sbjct  2   CASS-----QEASYEQYF  14
```

myelin basic protein-specific T-cell receptor beta chain VDJ region {clone HOO24} [human, peripheral blood mononuclear cells, Peptide Partial, 25 aa] [Homo sapiens]
Sequence ID: AAB33029.1Length: 25Number of Matches: 1
Range 1: 1 to 16GenPeptGraphics Next Match Previous Match

Alignment statistics for match #1

| Score | Expect | Identities | Positives | Gaps |
| --- | --- | --- | --- | --- |
| 24.8 bits(51) | 12 | 10/16(63%) | 10/16(62%) | 1/16(6%) |

```
Query  1   CASSRSER-EQYEQYF  15
           CASS    R    YEQYF
Sbjct  1   CASSSLARGASYEQYF  16
```

myelin basic protein specific T-cell receptor V beta-D beta-J beta, MBP reactive TCR VDJ beta {clone SE(5), rearranged CDR3 region} [human, inflammatory brain lesions, HLA phenotype 1, Peptide Partial, 26 aa] [Homo sapiens]

Sequence ID: AAB25639.1Length: 26Number of Matches: 1

Range 1: 2 to 17GenPeptGraphics Next Match Previous Match

Alignment statistics for match #1

| Score | Expect | Identities | Positives | Gaps |
| --- | --- | --- | --- | --- |
| 24.8 bits(51) | 12 | 10/16(63%) | 12/16(75%) | 1/16(6%) |

```
Query  1   CASSRSEREQ-YEQYF  15
           CASSR +R Q  EQ+F
Sbjct  2   CASSRLDRGQGEEQFF  17
```

myelin basic protein (143-168)-specific TCR beta-chain {clone BM3.3} [human, multiple sclerosis patient, T cells, Peptide Partial, 26 aa] [Homo sapiens]

Sequence ID: AAB31427.1Length: 26Number of Matches: 1

Range 1: 1 to 13GenPeptGraphics Next Match Previous Match

Alignment statistics for match #1

| Score | Expect | Identities | Positives | Gaps |
| --- | --- | --- | --- | --- |
| 20.6 bits(41) | 416 | 9/17(53%) | 9/17(52%) | 6/17(35%) |

```
Query  1   CASS--RSEREQYEQYF  15
           CASS  R      Y QYF
Sbjct  1   CASSLTRG----YTQYF  13
```

## TCR Heart 2.19 CASSQDGGYTYEQYF

myelin basic protein specific T-cell receptor V beta-D beta-J beta, MBP reactive TCR VDJ beta {CDR3 region, clone BM(5)} [human, peripheral blood lymphocytes, HLA phenotype 1, Peptide Partial, 23 aa] [Homo sapiens]
Sequence ID: AAB25666.1Length: 23Number of Matches: 1
Range 1: 2 to 14GenPeptGraphics Next Match Previous Match

Alignment statistics for match #1

| Score | Expect | Identities | Positives | Gaps |
|---|---|---|---|---|
| 32.9 bits(70) | 0.010 | 10/15(67%) | 11/15(73%) | 2/15(13%) |

```
Query  1    CASSQDGGYTYEQYF  15
            CASSQ+     YEQYF
Sbjct  2    CASSQEA--SYEQYF  14
```

myelin basic protein specific T-cell receptor V beta-D beta-J beta, MBP reactive TCR VDJ beta {clone SE(14), rearranged CDR3 region} [human, inflammatory brain lesions, HLA phenotype 1, Peptide Partial, 23 aa] [Homo sapiens]
Sequence ID: AAB25648.1Length: 23Number of Matches: 1
Range 1: 2 to 14GenPeptGraphics Next Match Previous Match

Alignment statistics for match #1

| Score | Expect | Identities | Positives | Gaps |
|---|---|---|---|---|
| 31.6 bits(67) | 0.031 | 11/15(73%) | 11/15(73%) | 2/15(13%) |

```
Query  1    CASSQDGGYTYEQYF  15
            CASS  G   TYEQYF
Sbjct  2    CASSTAG--TYEQYF  14
```

myelin basic protein specific T-cell receptor V beta-D beta-J beta, MBP reactive TCR VDJ beta {clone LJ 1(7), rearranged CDR3 region} [human, brain plaques, HLA phenotype 1, Peptide Partial, 25 aa] [Homo sapiens]
Sequence ID: AAB25633.1Length: 25Number of Matches: 1

- See 1 more title(s) See all Identical Proteins(IPG)

Related Information
Identical Proteins-Identical proteins to AAB25633.1
Range 1: 2 to 16GenPeptGraphics Next Match Previous Match

Alignment statistics for match #1

| Score | Expect | Identities | Positives | Gaps |
|---|---|---|---|---|
| 26.5 bits(55) | 2.8 | 10/15(67%) | 10/15(66%) | 0/15(0%) |

```
Query  1    CASSQDGGYTYEQYF  15
            CASS    G  YEQYF
Sbjct  2    CASSLVAGSIYEQYF  16
```

---

Download GenPeptGraphics Next Previous Descriptions
myelin basic protein specific T-cell receptor V beta-D beta-J beta, MBP reactive TCR VDJ beta {clone KL-1(10), rearranged CDR3 region} [human, brain plaques, HLA phenotype 1, Peptide Partial, 25 aa] [Homo sapiens]
Sequence ID: AAB25601.1Length: 25Number of Matches: 1

- See 3 more title(s) See all Identical Proteins(IPG)

Related Information
Identical Proteins-Identical proteins to AAB25601.1
Range 1: 2 to 16GenPeptGraphics Next Match Previous Match

Alignment statistics for match #1

| Score | Expect | Identities | Positives | Gaps |
|---|---|---|---|---|
| 26.5 bits(55) | 2.8 | 10/15(67%) | 10/15(66%) | 0/15(0%) |

```
Query  1   CASSQDGGYTYEQYF  15
           CASS    G   YEQYF
Sbjct  2   CASSLVAGSIYEQYF  16
```

myelin basic protein (143-168)-specific TCR beta-chain {clone JA.1.7} [human, multiple sclerosis patient, T cells, Peptide Partial, 27 aa] [Homo sapiens]
Sequence ID: AAB31429.1Length: 27Number of Matches: 1
Range 1: 1 to 14GenPeptGraphics Next Match Previous Match

Alignment statistics for match #1

| Score | Expect | Identities | Positives | Gaps |
|---|---|---|---|---|
| 26.5 bits(55) | 2.9 | 11/17(65%) | 11/17(64%) | 5/17(29%) |

```
Query  1   CASSQDGGYTYE--QYF  15
           CASS DGG    E  QYF
Sbjct  1   CASSLDGG---EETQYF  14
```

4-alpha-glucanotransferase [Clostridia bacterium]
Sequence ID: MBR2952274.1Length: 484Number of Matches: 1
Range 1: 33 to 40GenPeptGraphics Next Match Previous Match

Alignment statistics for match #1

| Score | Expect | Identities | Positives | Gaps |
|---|---|---|---|---|
| 24.8 bits(51) | 2794 | 7/8(88%) | 8/8(100%) | 0/8(0%) |

```
Query  1   LGRAGLTY  8
           LGRAG+TY
Sbjct  33  LGRAGMTY  40
```

## TCR Heart 7.1 CASSPVEPNMTEAFFG

TCR V beta {V-J junction, clone MS9-2C5} [human, multiple sclerosis patient, myelin basic protein-reactive T cells after vaccination, Peptide Partial, 30 aa] [Homo sapiens]
Sequence ID: AAB35724.1Length: 30Number of Matches: 1
Range 1: 12 to 17GenPeptGraphics Next Match Previous Match

Alignment statistics for match #1

| Score | Expect | Identities | Positives | Gaps |
|---|---|---|---|---|
| 22.3 bits(45) | 121 | 6/6(100%) | 6/6(100%) | 0/6(0%) |

```
Query  11  TEAFFG  16
           TEAFFG
Sbjct  12  TEAFFG  17
```

## TCR Heart 7.2 CASSTLTGPTEAFFG

TCR V beta {V-J junction, clone MS9-2C5} [human, multiple sclerosis patient, myelin basic protein-reactive T cells after vaccination, Peptide Partial, 30 aa] [Homo sapiens]
Sequence ID: AAB35724.1Length: 30Number of Matches: 1
Range 1: 12 to 17GenPeptGraphics Next Match Previous Match

Alignment statistics for match #1

| Score | Expect | Identities | Positives | Gaps |
|---|---|---|---|---|
| 22.3 bits(45) | 106 | 6/6(100%) | 6/6(100%) | 0/6(0%) |

```
Query  10  TEAFFG  15
           TEAFFG
Sbjct  12  TEAFFG  17
```

---

# TCR Heart 7.3 CASSQVLDNYGY

myelin basic protein specific T-cell receptor V beta-D beta-J beta, MBP reactive TCR VDJ beta {clone KL-3(3), rearranged CDR3 region} [human, brain plaques, HLA phenotype 1, Peptide Partial, 24 aa] [Homo sapiens]
Sequence ID: AAB25606.1Length: 24Number of Matches: 1
Range 1: 2 to 13GenPeptGraphics Next Match Previous Match

Alignment statistics for match #1

| Score | Expect | Identities | Positives | Gaps |
|---|---|---|---|---|
| 26.5 bits(55) | 1.7 | 10/14(71%) | 10/14(71%) | 4/14(28%) |

```
Query  1    CASSQVLD--NYGY  12
            CASS  LD  NYGY
Sbjct  2    CASS--LDGVNYGY  13
```

myelin basic protein specific T-cell receptor V beta-D beta-J beta, MBP reactive TCR VDJ beta {clone KL-3(1), rearranged CDR3 region} [human, brain plaques, HLA phenotype 1, Peptide Partial, 24 aa] [Homo sapiens]
Sequence ID: AAB25604.1Length: 24Number of Matches: 1
Range 1: 2 to 13GenPeptGraphics Next Match Previous Match

Alignment statistics for match #1

| Score | Expect | Identities | Positives | Gaps |
|---|---|---|---|---|
| 21.8 bits(44) | 91 | 9/14(64%) | 9/14(64%) | 4/14(28%) |

```
Query  1    CASSQVLDN--YGY  12
            CASS  LD   YGY
Sbjct  2    CASS--LDGVPYGY  13
```

Download GenPeptGraphics Next Previous Descriptions
myelin basic protein specific T-cell receptor V beta-D beta-J beta, MBP reactive TCR VDJ beta {clone KL-3(2), rearranged CDR3 region} [human, brain plaques, HLA phenotype 1, Peptide Partial, 24 aa] [Homo sapiens]
Sequence ID: AAB25605.1Length: 24Number of Matches: 1
Range 1: 2 to 13GenPeptGraphics Next Match Previous Match

Alignment statistics for match #1

| Score | Expect | Identities | Positives | Gaps |
|---|---|---|---|---|
| 21.8 bits(44) | 91 | 9/14(64%) | 9/14(64%) | 4/14(28%) |

```
Query  1    CASSQVLDN--YGY  12
            CASS  LD   YGY
```

```
Sbjct  2    CASS--LDGVPYGY  13
```

myelin basic protein specific T-cell receptor V beta-D beta-J beta, MBP reactive TCR VDJ beta {CDR3 region, clone JO(1)} [human, normal peripheral blood lymphocytes, Peptide Partial, 25 aa] [Homo sapiens]
Sequence ID: AAB25656.1Length: 25Number of Matches: 1
Range 1: 2 to 14GenPeptGraphics Next Match Previous Match

Alignment statistics for match #1

| Score | Expect | Identities | Positives | Gaps |
|---|---|---|---|---|
| 21.8 bits(44) | 92 | 9/13(69%) | 9/13(69%) | 1/13(7%) |

```
Query  1    CASSQVL-DNYGY  12
            CASS  L D YGY
Sbjct  2    CASSVALQDRYGY  14
```

18.5-kDa MBP-specific T cell Receptor beta chain VJ region {DR2/DQw1 varient, clone 1F5 98-170} [human, multiple sclerosis patient MS-B2, Peptide Partial, 16 aa] [Homo sapiens]
Sequence ID: AAB32431.1Length: 16Number of Matches: 1
Range 1: 1 to 13GenPeptGraphics Next Match Previous Match

Alignment statistics for match #1

| Score | Expect | Identities | Positives | Gaps |
|---|---|---|---|---|
| 19.7 bits(39) | 451 | 8/13(62%) | 8/13(61%) | 1/13(7%) |

```
Query  1    CASSQVLD-NYGY  12
            CAS    L  NYGY
Sbjct  1    CASRPGLGRNYGY  13
```

## TCR Heart 7.4 CASSQDSGRVYSRYGY

myelin basic protein specific T-cell receptor V beta-D beta-J beta, MBP reactive TCR VDJ beta {CDR3 region, clone JO(1)} [human, normal peripheral blood lymphocytes, Peptide Partial, 25 aa] [Homo sapiens]
Sequence ID: AAB25656.1Length: 25Number of Matches: 1
Range 1: 2 to 14GenPeptGraphics Next Match Previous Match

Alignment statistics for match #1

| Score | Expect | Identities | Positives | Gaps |
|---|---|---|---|---|
| 24.8 bits(51) | 13 | 10/19(53%) | 10/19(52%) | 9/19(47%) |

```
Query  1    CASS---QDSGRVYSRYGY  16
            CASS   QD      RYGY
Sbjct  2    CASSVALQD------RYGY  14
```

## TCR Heart 7.5 CASSQDKGAYFG

myelin basic protein-specific T-cell receptor beta chain VDJ region {clone JK1.16} [human, peripheral blood mononuclear cells, Peptide Partial, 27 aa] [Homo sapiens]
Sequence ID: AAB33031.1Length: 27Number of Matches: 1
Range 1: 1 to 19GenPeptGraphics Next Match Previous Match

Alignment statistics for match #1

| Score | Expect | Identities | Positives | Gaps |
|---|---|---|---|---|
| 23.1 bits(47) | 32 | 9/19(47%) | 11/19(57%) | 7/19(36%) |

```
Query  1    CASSQDKGA-------YFG  12
            CASSQ+ GA       +FG
Sbjct  1    CASSQEEGAGGPFNEQFFG  19
```

myelin basic protein specific T-cell receptor V beta-D beta-J beta, MBP reactive TCR VDJ beta {CDR3 region, clone BM(5)} [human, peripheral blood lymphocytes, HLA phenotype 1, Peptide Partial, 23 aa] [Homo sapiens]
Sequence ID: AAB25666.1Length: 23Number of Matches: 1
Range 1: 2 to 15GenPeptGraphics Next Match Previous Match

Alignment statistics for match #1

| Score | Expect | Identities | Positives | Gaps |
|---|---|---|---|---|
| 22.3 bits(45) | 62 | 9/16(56%) | 10/16(62%) | 6/16(37%) |

```
Query  1    CASSQDKGA----YFG  12
            CASSQ+  A    YFG
Sbjct  2    CASSQE--ASYEQYFG  15
```

myelin basic protein (143-168)-specific TCR beta-chain {clone JA.1.7} [human, multiple sclerosis patient, T cells, Peptide Partial, 27 aa] [Homo sapiens]
Sequence ID: AAB31429.1Length: 27Number of Matches: 1
Range 1: 1 to 15GenPeptGraphics Next Match Previous Match

Alignment statistics for match #1

| Score | Expect | Identities | Positives | Gaps |
|---|---|---|---|---|
| 20.6 bits(41) | 272 | 9/15(60%) | 9/15(60%) | 3/15(20%) |

```
Query  1    CASSQDKGA---YFG  12
```

```
             CASS D G    YFG
Sbjct   1    CASSLDGGEETQYFG  15
```

## TCR Heart 7.6 CASSQDRGQGFNEQFFG

myelin basic protein-specific T-cell receptor beta chain VDJ region {clone JK1.16} [human, peripheral blood mononuclear cells, Peptide Partial, 27 aa] [Homo sapiens]
Sequence ID: AAB33031.1Length: 27Number of Matches: 1
Range 1: 1 to 19GenPeptGraphics Next Match Previous Match

Alignment statistics for match #1

| Score | Expect | Identities | Positives | Gaps |
|---|---|---|---|---|
| 38.4 bits(83) | 1e-04 | 14/19(74%) | 15/19(78%) | 2/19(10%) |

```
Query   1    CASSQDRGQG--FNEQFFG  17
             CASSQ+ G G  FNEQFFG
Sbjct   1    CASSQEEGAGGPFNEQFFG  19
```

myelin basic protein specific T-cell receptor V beta-D beta-J beta, MBP reactive TCR VDJ beta {clone SE(5), rearranged CDR3 region} [human, inflammatory brain lesions, HLA phenotype 1, Peptide Partial, 26 aa] [Homo sapiens]
Sequence ID: AAB25639.1Length: 26Number of Matches: 1
Range 1: 2 to 18GenPeptGraphics Next Match Previous Match

Alignment statistics for match #1

| Score | Expect | Identities | Positives | Gaps |
|---|---|---|---|---|
| 37.1 bits(80) | 4e-04 | 14/18(78%) | 14/18(77%) | 2/18(11%) |

```
Query   1    CASSQ-DRGQGFNEQFFG  17
             CASS  DRGQG  EQFFG
Sbjct   2    CASSRLDRGQG-EEQFFG  18
```

myelin basic protein-specific T-cell receptor beta chain VDJ region {clone FN2.2} [human, peripheral blood mononuclear cells, Peptide Partial, 25 aa] [Homo sapiens]
Sequence ID: AAB33027.1Length: 25Number of Matches: 1
Range 1: 1 to 17GenPeptGraphics Next Match Previous Match

Alignment statistics for match #1

| Score | Expect | Identities | Positives | Gaps |
|---|---|---|---|---|
| 32.0 bits(68) | 0.032 | 13/18(72%) | 14/18(77%) | 2/18(11%) |

```
Query  1    CASSQDRGQG-FNEQFFG  17
            CASS   RG G +NEQFFG
Sbjct  1    CASSL-RGSGTYNEQFFG  17
```

myelin basic protein specific T-cell receptor V beta-D beta-J beta, MBP reactive TCR VDJ beta {clone KL-3(8), rearranged CDR3 region} [human, brain plaques, HLA phenotype 1, Peptide Partial, 25 aa] [Homo sapiens]

Sequence ID: AAB25611.1Length: 25Number of Matches: 1

Range 1: 2 to 17GenPeptGraphics Next Match Previous Match

Alignment statistics for match #1

| Score | Expect | Identities | Positives | Gaps |
|---|---|---|---|---|
| 29.1 bits(61) | 0.41 | 11/17(65%) | 13/17(76%) | 1/17(5%) |

```
Query  1    CASSQDRGQGFNEQFFG  17
            CASS +    G+NEQFFG
Sbjct  2    CASSLELA-GYNEQFFG  17
```

myelin basic protein specific T-cell receptor V beta-D beta-J beta, MBP reactive TCR VDJ beta {clone KL-3(7), rearranged CDR3 region} [human, brain plaques, HLA phenotype 1, Peptide Partial, 24 aa] [Homo sapiens]

Sequence ID: AAB25610.1Length: 24Number of Matches: 1

Range 1: 2 to 16GenPeptGraphics Next Match Previous Match

Alignment statistics for match #1

| Score | Expect | Identities | Positives | Gaps |
|---|---|---|---|---|
| 28.6 bits(60) | 0.57 | 11/20(55%) | 12/20(60%) | 8/20(40%) |

```
Query  1    CASSQDRGQG---FNEQFFG  17
            CASSQ        +NEQFFG
Sbjct  2    CASSQ-----PAVYNEQFFG  16
```

myelin basic protein (84-102)-specific TCR beta-chain {clone NSJL5.7} [human, multiple sclerosis patient, T cells, Peptide Partial, 28 aa] [Homo sapiens]

Sequence ID: AAB31431.1Length: 28Number of Matches: 1

Range 1: 1 to 16GenPeptGraphics Next Match Previous Match

Alignment statistics for match #1

| Score | Expect | Identities | Positives | Gaps |
|---|---|---|---|---|
| 26.1 bits(54) | 5.6 | 11/17(65%) | 12/17(70%) | 1/17(5%) |

```
Query  1    CASSQDRGQGFNEQFFG  17
            CASS    G   +NEQFFG
Sbjct  1    CASSLTVG-SYNEQFFG  16
```

## TCR Heart 7.7 CASSQNIGASGNEQFFG

myelin basic protein-specific T-cell receptor beta chain VDJ region {clone JK1.16} [human, peripheral blood mononuclear cells, Peptide Partial, 27 aa] [Homo sapiens]
Sequence ID: AAB33031.1Length: 27Number of Matches: 1
Range 1: 1 to 19GenPeptGraphics Next Match Previous Match

Alignment statistics for match #1

| Score | Expect | Identities | Positives | Gaps |
|---|---|---|---|---|
| 38.0 bits(82) | 2e-04 | 14/19(74%) | 14/19(73%) | 2/19(10%) |

```
Query  1   CASSQNIGASG--NEQFFG  17
           CASSQ  GA G  NEQFFG
Sbjct  1   CASSQEEGAGGPFNEQFFG  19
```

myelin basic protein specific T-cell receptor V beta-D beta-J beta, MBP reactive TCR VDJ beta {clone KL-1(8), rearranged CDR3 region} [human, brain plaques, HLA phenotype 1, Peptide Partial, 25 aa] [Homo sapiens]
Sequence ID: AAB25599.1Length: 25Number of Matches: 1
Range 1: 2 to 17GenPeptGraphics Next Match Previous Match

Alignment statistics for match #1

| Score | Expect | Identities | Positives | Gaps |
|---|---|---|---|---|
| 30.8 bits(65) | 0.095 | 12/21(57%) | 12/21(57%) | 9/21(42%) |

```
Query  1   CASSQ----NIGASGNEQFFG  17
           CASSQ    N     NEQFFG
Sbjct  2   CASSQPTVYN-----NEQFFG  17
```

myelin basic protein (84-102)-specific TCR beta-chain {clone NSJL5.7} [human, multiple sclerosis patient, T cells, Peptide Partial, 28 aa] [Homo sapiens]
Sequence ID: AAB31431.1Length: 28Number of Matches: 1
Range 1: 1 to 16GenPeptGraphics Next Match Previous Match

Alignment statistics for match #1

| Score | Expect | Identities | Positives | Gaps |
|---|---|---|---|---|
| 28.6 bits(60) | 0.66 | 11/17(65%) | 12/17(70%) | 1/17(5%) |

```
Query  1   CASSQNIGASGNEQFFG  17
           CASS  +G   NEQFFG
Sbjct  1   CASSLTVGSY-NEQFFG  16
```

myelin basic protein specific T-cell receptor V beta-D beta-J beta, MBP reactive TCR VDJ beta {clone KL-3(7), rearranged CDR3 region} [human, brain plaques, HLA phenotype 1, Peptide Partial, 24 aa] [Homo sapiens]

---

Sequence ID: AAB25610.1Length: 24Number of Matches: 1

Range 1: 2 to 16GenPeptGraphics Next Match Previous Match

Alignment statistics for match #1

| Score | Expect | Identities | Positives | Gaps |
|---|---|---|---|---|
| 26.9 bits(56) | 2.4 | 11/21(52%) | 11/21(52%) | 10/21(47%) |

```
Query  1    CASSQNIGASG----NEQFFG  17
            CASSQ          NEQFFG
Sbjct  2    CASSQ------PAVYNEQFFG  16
```

myelin basic protein (84-102)-specific TCR beta-chain {clone NB17.8} [human, multiple sclerosis patient, T cells, Peptide Partial, 27 aa] [Homo sapiens]

Sequence ID: AAB31433.1Length: 27Number of Matches: 1

Range 1: 1 to 15GenPeptGraphics Next Match Previous Match

Alignment statistics for match #1

| Score | Expect | Identities | Positives | Gaps |
|---|---|---|---|---|
| 26.9 bits(56) | 2.7 | 12/19(63%) | 12/19(63%) | 6/19(31%) |

```
Query  1    CASSQNIGAS--GNEQFFG  17
            CASS     S  GNEQFFG
Sbjct  1    CASS----YSPLGNEQFFG  15
```

myelin basic protein (84-102)-specific TCR beta-chain {clone NSJL5.7} [human, multiple sclerosis patient, T cells, Peptide Partial, 28 aa] [Homo sapiens]

Sequence ID: AAB31431.1Length: 28Number of Matches: 1

Range 1: 1 to 16GenPeptGraphics Next Match Previous Match

Alignment statistics for match #1

| Score | Expect | Identities | Positives | Gaps |
|---|---|---|---|---|
| 28.6 bits(60) | 0.66 | 11/17(65%) | 12/17(70%) | 1/17(5%) |

```
Query  1    CASSQNIGASGNEQFFG  17
            CASS  +G   NEQFFG
Sbjct  1    CASSLTVGSY-NEQFFG  16
```

myelin basic protein (84-102)-specific TCR beta-chain {clone NB17.8} [human, multiple sclerosis patient, T cells, Peptide Partial, 27 aa] [Homo sapiens]

Sequence ID: AAB31433.1Length: 27Number of Matches: 1

Range 1: 1 to 15GenPeptGraphics Next Match Previous Match

Alignment statistics for match #1

| Score | Expect | Identities | Positives | Gaps |
|---|---|---|---|---|
| 26.9 bits(56) | 2.7 | 12/19(63%) | 12/19(63%) | 6/19(31%) |

```
Query  1    CASSQNIGAS--GNEQFFG  17
            CASS     S  GNEQFFG
```

---

```
Sbjct  1   CASS----YSPLGNEQFFG  15
```

## TCR Heart 7.8 CASSQDWQNCYFG

## NONE

## TCR Heart 7.9  CASSYGYEQYF

myelin basic protein specific T-cell receptor V beta-D beta-J beta, MBP reactive TCR VDJ beta {clone SE(13), rearranged CDR3 region} [human, inflammatory brain lesions, HLA phenotype 1, Peptide Partial, 23 aa] [Homo sapiens]
Sequence ID: AAB25647.1Length: 23Number of Matches: 1
Range 1: 2 to 14GenPeptGraphics Next Match Previous Match

Alignment statistics for match #1

| Score | Expect | Identities | Positives | Gaps |
|---|---|---|---|---|
| 32.9 bits(70) | 0.005 | 10/13(77%) | 11/13(84%) | 2/13(15%) |

```
Query  1   CASSY--GYEQYF  11
           CASS+  GYEQYF
Sbjct  2   CASSFIWGYEQYF  14
```

myelin basic protein specific T-cell receptor V beta-D beta-J beta, MBP reactive TCR VDJ beta {clone SE(12), rearranged CDR3 region} [human, inflammatory brain lesions, HLA phenotype 1, Peptide Partial, 25 aa] [Homo sapiens]
Sequence ID: AAB25646.1Length: 25Number of Matches: 1
Range 1: 2 to 15GenPeptGraphics Next Match Previous Match

Alignment statistics for match #1

---

| Score | Expect | Identities | Positives | Gaps |
|---|---|---|---|---|
| 31.6 bits(67) | 0.018 | 10/14(71%) | 10/14(71%) | 4/14(28%) |

```
Query  1    CASSYG----YEQY  10
            CASSYG    YEQY
Sbjct  2    CASSYGTSGIYEQY  15
```

myelin basic protein (84-102)-specific TCR beta-chain {clone NB17.8} [human, multiple sclerosis patient, T cells, Peptide Partial, 27 aa] [Homo sapiens]

Sequence ID: AAB31433.1Length: 27Number of Matches: 1

Range 1: 1 to 14GenPeptGraphics Next Match Previous Match

Alignment statistics for match #1

| Score | Expect | Identities | Positives | Gaps |
|---|---|---|---|---|
| 26.5 bits(55) | 1.6 | 9/14(64%) | 10/14(71%) | 3/14(21%) |

```
Query  1    CASSY---GYEQYF  11
            CASSY   G EQ+F
Sbjct  1    CASSYSPLGNEQFF  14
```

T-cell receptor beta chain variable region {junctional region} [human, monozygous twin, myelin basic protein-specific T cell line, Peptide Partial, 22 aa] [Homo sapiens]

Sequence ID: AAB37680.1Length: 22Number of Matches: 1

Range 1: 1 to 12GenPeptGraphics Next Match Previous Match

Alignment statistics for match #1

| Score | Expect | Identities | Positives | Gaps |
|---|---|---|---|---|
| 26.1 bits(54) | 1.9 | 9/13(69%) | 10/13(76%) | 3/13(23%) |

```
Query  1    CASSYG--YEQYF  11
            CAS +G  YEQYF
Sbjct  1    CAS-FGSSYEQYF  12
```

myelin basic protein specific T-cell receptor V beta-D beta-J beta, MBP reactive TCR VDJ beta {clone SE(14), rearranged CDR3 region} [human, inflammatory brain lesions, HLA phenotype 1, Peptide Partial, 23 aa] [Homo sapiens]

Sequence ID: AAB25648.1Length: 23Number of Matches: 1

Range 1: 2 to 14GenPeptGraphics Next Match Previous Match

Alignment statistics for match #1

| Score | Expect | Identities | Positives | Gaps |
|---|---|---|---|---|
| 26.1 bits(54) | 2.0 | 9/13(69%) | 9/13(69%) | 2/13(15%) |

```
Query  1    CASSYG--YEQYF  11
            CASS    YEQYF
Sbjct  2    CASSTAGTYEQYF  14
```

## TCR Heart 7.10 CASSQGAEPLQYEQYF

myelin basic protein specific T-cell receptor V beta-D beta-J beta, MBP reactive TCR VDJ beta {CDR3 region, clone BM(5)} [human, peripheral blood lymphocytes, HLA phenotype 1, Peptide Partial, 23 aa] [Homo sapiens]
Sequence ID: AAB25666.1Length: 23Number of Matches: 1
Range 1: 2 to 14GenPeptGraphics Next Match Previous Match

Alignment statistics for match #1

| Score | Expect | Identities | Positives | Gaps |
|---|---|---|---|---|
| 31.6 bits(67) | 0.035 | 11/16(69%) | 11/16(68%) | 3/16(18%) |

```
Query  1   CASSQGAEPLQYEQYF  16
           CASSQ A     YEQYF
Sbjct  2   CASSQEAS---YEQYF  14
```

myelin basic protein-specific T-cell receptor beta chain VDJ region {clone HOO24} [human, peripheral blood mononuclear cells, Peptide Partial, 25 aa] [Homo sapiens]
Sequence ID: AAB33029.1Length: 25Number of Matches: 1
Range 1: 1 to 16GenPeptGraphics Next Match Previous Match

Alignment statistics for match #1

| Score | Expect | Identities | Positives | Gaps |
|---|---|---|---|---|
| 26.5 bits(55) | 3.2 | 11/19(58%) | 11/19(57%) | 6/19(31%) |

```
Query  1   CASS---QGAEPLQYEQYF  16
           CASS    GA     YEQYF
Sbjct  1   CASSSLARGAS---YEQYF  16
```

myelin basic protein specific T-cell receptor V beta-D beta-J beta, MBP reactive TCR VDJ beta {clone LJ 1(7), rearranged CDR3 region} [human, brain plaques, HLA phenotype 1, Peptide Partial, 25 aa] [Homo sapiens]
Sequence ID: AAB25633.1Length: 25Number of Matches: 1

- See 1 more title(s) See all Identical Proteins(IPG)

Related Information
Identical Proteins-Identical proteins to AAB25633.1
Range 1: 2 to 16GenPeptGraphics Next Match Previous Match

Alignment statistics for match #1

| Score | Expect | Identities | Positives | Gaps |
|---|---|---|---|---|
| 25.2 bits(52) | 9.4 | 10/16(63%) | 10/16(62%) | 1/16(6%) |

```
Query  1    CASSQGAEPLQYEQYF  16
            CASS  A    YEQYF
Sbjct  2    CASSLVAGSI-YEQYF  16
```

Download GenPeptGraphics Next Previous Descriptions

myelin basic protein specific T-cell receptor V beta-D beta-J beta, MBP reactive TCR VDJ beta {clone KL-1(10), rearranged CDR3 region} [human, brain plaques, HLA phenotype 1, Peptide Partial, 25 aa] [Homo sapiens]

Sequence ID: AAB25601.1Length: 25Number of Matches: 1

- See 3 more title(s) See all Identical Proteins(IPG)

Related Information

Identical Proteins-Identical proteins to AAB25601.1

Range 1: 2 to 16GenPeptGraphics Next Match Previous Match

Alignment statistics for match #1

| Score | Expect | Identities | Positives | Gaps |
|---|---|---|---|---|
| 25.2 bits(52) | 9.4 | 10/16(63%) | 10/16(62%) | 1/16(6%) |

```
Query  1    CASSQGAEPLQYEQYF  16
            CASS  A    YEQYF
Sbjct  2    CASSLVAGSI-YEQYF  16
```

myelin basic protein specific T-cell receptor V beta-D beta-J beta, MBP reactive TCR VDJ beta {clone SE(14), rearranged CDR3 region} [human, inflammatory brain lesions, HLA phenotype 1, Peptide Partial, 23 aa] [Homo sapiens]

Sequence ID: AAB25648.1Length: 23Number of Matches: 1

Range 1: 2 to 14GenPeptGraphics Next Match Previous Match

Alignment statistics for match #1

| Score | Expect | Identities | Positives | Gaps |
|---|---|---|---|---|
| 24.8 bits(51) | 13 | 10/17(59%) | 10/17(58%) | 5/17(29%) |

```
Query  1    CASS-QGAEPLQYEQYF  16
            CASS  G     YEQYF
Sbjct  2    CASSTAGT----YEQYF  14
```

myelin basic protein specific T-cell receptor V beta-D beta-J beta, MBP reactive TCR VDJ beta {clone KL-3(12), rearranged CDR3 region} [human, brain plaques, HLA phenotype 1, Peptide Partial, 26 aa] [Homo sapiens]

Sequence ID: AAB25615.1Length: 26Number of Matches: 1

Range 1: 2 to 17GenPeptGraphics Next Match Previous Match

Alignment statistics for match #1

| Score | Expect | Identities | Positives | Gaps |
|---|---|---|---|---|

---

24.8 bits(51) 14 11/17(65%) 11/17(64%) 2/17(11%)

```
Query  1    CASSQGAEPLQYE-QYF  16
            CASS G   LQ E QYF
Sbjct  2    CASSLGSGTLQ-ETQYF  17
```

## TCR Heart 7.11  CASSQDKNYEQYF

myelin basic protein specific T-cell receptor V beta-D beta-J beta, MBP reactive TCR VDJ beta {CDR3 region, clone BM(5)} [human, peripheral blood lymphocytes, HLA phenotype 1, Peptide Partial, 23 aa] [Homo sapiens]
Sequence ID: AAB25666.1Length: 23Number of Matches: 1
Range 1: 2 to 14GenPeptGraphics Next Match Previous Match

| Alignment statistics for match #1 | | | | |
|---|---|---|---|---|
| **Score** | **Expect** | **Identities** | **Positives** | **Gaps** |
| 35.4 bits(76) | 8e-04 | 10/13(77%) | 11/13(84%) | 0/13(0%) |

```
Query  1    CASSQDKNYEQYF  13
            CASSQ+   YEQYF
Sbjct  2    CASSQEASYEQYF  14
```

myelin basic protein specific T-cell receptor V beta-D beta-J beta, MBP reactive TCR VDJ beta {clone SE(14), rearranged CDR3 region} [human, inflammatory brain lesions, HLA phenotype 1, Peptide Partial, 23 aa] [Homo sapiens]
Sequence ID: AAB25648.1Length: 23Number of Matches: 1
Range 1: 2 to 14GenPeptGraphics Next Match Previous Match

| Alignment statistics for match #1 | | | | |
|---|---|---|---|---|
| **Score** | **Expect** | **Identities** | **Positives** | **Gaps** |
| 26.9 bits(56) | 1.3 | 9/13(69%) | 9/13(69%) | 0/13(0%) |

```
Query  1    CASSQDKNYEQYF  13
```

```
            CASS    YEQYF
Sbjct  2    CASSTAGTYEQYF  14
```

T-cell receptor beta chain variable region {junctional region} [human, monozygous twin, myelin basic protein-specific T cell line, Peptide Partial, 22 aa] [Homo sapiens]
Sequence ID: AAB37680.1Length: 22Number of Matches: 1
Range 1: 1 to 12GenPeptGraphics Next Match Previous Match

Alignment statistics for match #1

| Score | Expect | Identities | Positives | Gaps |
|---|---|---|---|---|
| 24.4 bits(50) | 12 | 9/15(60%) | 9/15(60%) | 5/15(33%) |

```
Query  1    CAS--SQDKNYEQYF  13
            CAS  S    YEQYF
Sbjct  1    CASFGS---SYEQYF  12
```

myelin basic protein-specific T-cell receptor beta chain VDJ region {clone HOO24} [human, peripheral blood mononuclear cells, Peptide Partial, 25 aa] [Homo sapiens]
Sequence ID: AAB33029.1Length: 25Number of Matches: 1
Range 1: 1 to 16GenPeptGraphics Next Match Previous Match

Alignment statistics for match #1

| Score | Expect | Identities | Positives | Gaps |
|---|---|---|---|---|
| 24.0 bits(49) | 18 | 9/16(56%) | 9/16(56%) | 3/16(18%) |

```
Query  1    CASS---QDKNYEQYF  13
            CASS       YEQYF
Sbjct  1    CASSSLARGASYEQYF  16
```

myelin basic protein (143-168)-specific TCR beta-chain {clone BM3.3} [human, multiple sclerosis patient, T cells, Peptide Partial, 26 aa] [Homo sapiens]
Sequence ID: AAB31427.1Length: 26Number of Matches: 1
Range 1: 1 to 13GenPeptGraphics Next Match Previous Match

Alignment statistics for match #1

| Score | Expect | Identities | Positives | Gaps |
|---|---|---|---|---|
| 22.7 bits(46) | 54 | 8/13(62%) | 8/13(61%) | 0/13(0%) |

```
Query  1    CASSQDKNYEQYF  13
            CASS    Y QYF
Sbjct  1    CASSLTRGYTQYF  13
```

## TCR Heart 7.12 CASSFNREAYEQYF

myelin basic protein specific T-cell receptor V beta-D beta-J beta, MBP reactive TCR VDJ beta {CDR3 region, clone BM(5)} [human, peripheral blood lymphocytes, HLA phenotype 1, Peptide Partial, 23 aa] [Homo sapiens]
Sequence ID: AAB25666.1Length: 23Number of Matches: 1
Range 1: 2 to 14GenPeptGraphics Next Match Previous Match

Alignment statistics for match #1

| Score | Expect | Identities | Positives | Gaps |
|---|---|---|---|---|
| 30.3 bits(64) | 0.080 | 11/15(73%) | 11/15(73%) | 3/15(20%) |

```
Query  1    CASSFNREA-YEQYF  14
            CASS    EA YEQYF
Sbjct  2    CASS--QEASYEQYF  14
```

myelin basic protein specific T-cell receptor V beta-D beta-J beta, MBP reactive TCR VDJ beta {clone SE(13), rearranged CDR3 region} [human, inflammatory brain lesions, HLA phenotype 1, Peptide Partial, 23 aa] [Homo sapiens]
Sequence ID: AAB25647.1Length: 23Number of Matches: 1
Range 1: 2 to 14GenPeptGraphics Next Match Previous Match

Alignment statistics for match #1

| Score | Expect | Identities | Positives | Gaps |
|---|---|---|---|---|
| 29.9 bits(63) | 0.12 | 10/14(71%) | 10/14(71%) | 1/14(7%) |

```
Query  1    CASSFNREAYEQYF  14
            CASSF     YEQYF
Sbjct  2    CASSFIW-GYEQYF  14
```

myelin basic protein-specific T-cell receptor beta chain VDJ region {clone HOO24} [human, peripheral blood mononuclear cells, Peptide Partial, 25 aa] [Homo sapiens]
Sequence ID: AAB33029.1Length: 25Number of Matches: 1
Range 1: 1 to 16GenPeptGraphics Next Match Previous Match

Alignment statistics for match #1

| Score | Expect | Identities | Positives | Gaps |
|---|---|---|---|---|
| 26.9 bits(56) | 1.7 | 11/16(69%) | 11/16(68%) | 2/16(12%) |

```
Query  1    CASS-FNREA-YEQYF  14
            CASS    R A YEQYF
Sbjct  1    CASSSLARGASYEQYF  16
```

myelin basic protein specific T-cell receptor V beta-D beta-J beta, MBP TCR reactive VDJ beta {CDR3 region, muscle infiltrating lymphocytes(2)} [human, muscle infiltrating lymphocytes, HLA phenotype 1, Peptide Partial, 25 aa] [Homo sapiens]
Sequence ID: AAB25650.1Length: 25Number of Matches: 1
Range 1: 2 to 16GenPeptGraphics Next Match Previous Match

Alignment statistics for match #1

| Score | Expect | Identities | Positives | Gaps |
|---|---|---|---|---|
| 25.7 bits(53) | 4.9 | 10/16(63%) | 11/16(68%) | 3/16(18%) |

```
Query  1    CASSFNREAY--EQYF  14
            CASSF   AY  EQ+F
Sbjct  2    CASSFT-GAYYNEQFF  16
```

myelin basic protein specific T-cell receptor V beta-D beta-J beta, MBP reactive TCR VDJ beta {clone SE(14), rearranged CDR3 region} [human, inflammatory brain lesions, HLA phenotype 1, Peptide Partial, 23 aa] [Homo sapiens]
Sequence ID: AAB25648.1Length: 23Number of Matches: 1
Range 1: 2 to 14GenPeptGraphics Next Match Previous Match

Alignment statistics for match #1

| Score | Expect | Identities | Positives | Gaps |
|---|---|---|---|---|
| 24.4 bits(50) | 14 | 10/16(63%) | 10/16(62%) | 5/16(31%) |

```
Query  1    CASSFNREA--YEQYF  14
            CASS    A  YEQYF
Sbjct  2    CASS---TAGTYEQYF  14
```

myelin basic protein (143-168)-specific TCR beta-chain {clone BM3.3} [human, multiple sclerosis patient, T cells, Peptide Partial, 26 aa] [Homo sapiens]
Sequence ID: AAB31427.1Length: 26Number of Matches: 1
Range 1: 1 to 13GenPeptGraphics Next Match Previous Match

Alignment statistics for match #1

| Score | Expect | Identities | Positives | Gaps |
|---|---|---|---|---|
| 24.4 bits(50) | 15 | 9/14(64%) | 9/14(64%) | 1/14(7%) |

```
Query  1    CASSFNREAYEQYF  14
            CASS  R  Y QYF
Sbjct  1    CASSLTR-GYTQYF  13
```

18.5-kDa MBP-specific T cell Receptor beta chain VJ region {DR2/DQw1 varient, clone 2H5 1-97} [human, multiple sclerosis patient MS-B1, Peptide Partial, 16 aa] [Homo sapiens]
Sequence ID: AAB32427.1Length: 16Number of Matches: 1
Range 1: 1 to 15GenPeptGraphics Next Match Previous Match

Alignment statistics for match #1

| Score | Expect | Identities | Positives | Gaps |
|---|---|---|---|---|
| 22.7 bits(46) | 45 | 9/17(53%) | 10/17(58%) | 5/17(29%) |

```
Query  1   CASSF---NREAYEQYF  14
           CASS+    RE   QYF
Sbjct  1   CASSYRGAGRET--QYF  15
```

# TCR Heart 7.13 CASSSGLAGYEQYF

myelin basic protein-specific T-cell receptor beta chain VDJ region {clone HOO24} [human, peripheral blood mononuclear cells, Peptide Partial, 25 aa] [Homo sapiens]
Sequence ID: AAB33029.1Length: 25Number of Matches: 1
Range 1: 1 to 16GenPeptGraphics Next Match Previous Match

Alignment statistics for match #1

| Score | Expect | Identities | Positives | Gaps |
|---|---|---|---|---|
| 32.0 bits(68) | 0.020 | 12/17(71%) | 12/17(70%) | 4/17(23%) |

```
Query  1   CASSSGLA---GYEQYF  14
           CASSS LA     YEQYF
Sbjct  1   CASSS-LARGASYEQYF  16
```

myelin basic protein specific T-cell receptor V beta-D beta-J beta, MBP reactive TCR VDJ beta {clone SE(14), rearranged CDR3 region} [human, inflammatory brain lesions, HLA phenotype 1, Peptide Partial, 23 aa] [Homo sapiens]
Sequence ID: AAB25648.1Length: 23Number of Matches: 1
Range 1: 2 to 14GenPeptGraphics Next Match Previous Match

Alignment statistics for match #1

| Score | Expect | Identities | Positives | Gaps |
|---|---|---|---|---|
| 30.3 bits(64) | 0.080 | 11/15(73%) | 11/15(73%) | 3/15(20%) |

```
Query  1   CASSSGLAG-YEQYF  14
           CASS   AG YEQYF
Sbjct  2   CASST--AGTYEQYF  14
```

myelin basic protein specific T-cell receptor V beta-D beta-J beta, MBP reactive TCR VDJ beta {clone KL-3(8), rearranged CDR3 region} [human, brain plaques, HLA phenotype 1, Peptide Partial, 25 aa] [Homo sapiens]
Sequence ID: AAB25611.1Length: 25Number of Matches: 1
Range 1: 2 to 16GenPeptGraphics Next Match Previous Match

Alignment statistics for match #1

| Score | Expect | Identities | Positives | Gaps |
|---|---|---|---|---|
| 29.5 bits(62) | 0.19 | 11/15(73%) | 12/15(80%) | 1/15(6%) |

```
Query  1   CASSSGLAGY-EQYF  14
           CASS  LAGY EQ+F
Sbjct  2   CASSLELAGYNEQFF  16
```

myelin basic protein specific T-cell receptor V beta-D beta-J beta, MBP reactive TCR VDJ beta {clone LJ 1(6), rearranged CDR3 region} [human, brain plaques, HLA phenotype 1, Peptide Partial, 24 aa] [Homo sapiens]
Sequence ID: AAB25632.1Length: 24Number of Matches: 1
Range 1: 2 to 15GenPeptGraphics Next Match Previous Match

Alignment statistics for match #1

| Score | Expect | Identities | Positives | Gaps |
|---|---|---|---|---|
| 29.1 bits(61) | 0.26 | 12/16(75%) | 12/16(75%) | 4/16(25%) |

```
Query  1   CASSSGL-AG-YEQYF  14
           CASS  L AG YEQYF
Sbjct  2   CASS--LVAGIYEQYF  15
```

myelin basic protein specific T-cell receptor V beta-D beta-J beta, MBP reactive TCR VDJ beta {clone LJ 1(7), rearranged CDR3 region} [human, brain plaques, HLA phenotype 1, Peptide Partial, 25 aa] [Homo sapiens]
Sequence ID: AAB25633.1Length: 25Number of Matches: 1

- See 1 more title(s) See all Identical Proteins(IPG)

Related Information
Identical Proteins-Identical proteins to AAB25633.1
Range 1: 2 to 16GenPeptGraphics Next Match Previous Match

Alignment statistics for match #1

| Score | Expect | Identities | Positives | Gaps |
|---|---|---|---|---|
| 28.6 bits(60) | 0.39 | 12/17(71%) | 12/17(70%) | 5/17(29%) |

```
Query  1   CASSSGL-AG--YEQYF  14
           CASS  L AG  YEQYF
Sbjct  2   CASS--LVAGSIYEQYF  16
```

Download GenPeptGraphics Next Previous Descriptions
myelin basic protein specific T-cell receptor V beta-D beta-J beta, MBP reactive TCR VDJ beta {clone KL-1(10), rearranged CDR3 region} [human, brain plaques, HLA phenotype 1, Peptide Partial, 25 aa] [Homo sapiens]
Sequence ID: AAB25601.1Length: 25Number of Matches: 1

- See 3 more title(s) See all Identical Proteins(IPG)

Related Information
Identical Proteins-Identical proteins to AAB25601.1
Range 1: 2 to 16GenPeptGraphics Next Match Previous Match

Alignment statistics for match #1

| Score | Expect | Identities | Positives | Gaps |
|---|---|---|---|---|
| 28.6 bits(60) | 0.39 | 12/17(71%) | 12/17(70%) | 5/17(29%) |

```
Query  1   CASSSGL-AG--YEQYF  14
           CASS  L AG  YEQYF
Sbjct  2   CASS--LVAGSIYEQYF  16
```

myelin basic protein specific T-cell receptor V beta-D beta-J beta, MBP reactive TCR VDJ beta {CDR3 region, clone BM(5)} [human, peripheral blood lymphocytes, HLA phenotype 1, Peptide Partial, 23 aa] [Homo sapiens]
Sequence ID: AAB25666.1Length: 23Number of Matches: 1
Range 1: 2 to 14GenPeptGraphics Next Match Previous Match

Alignment statistics for match #1

| Score | Expect | Identities | Positives | Gaps |
|---|---|---|---|---|
| 27.8 bits(58) | 0.74 | 10/14(71%) | 10/14(71%) | 1/14(7%) |

```
Query  1   CASSSGLAGYEQYF  14
           CASS    A YEQYF
Sbjct  2   CASSQE-ASYEQYF  14
```

myelin basic protein specific T-cell receptor V beta-D beta-J beta, MBP reactive TCR VDJ beta {clone SE(7), rearranged CDR3 region} [human, inflammatory brain lesions, HLA phenotype 1, Peptide Partial, 26 aa] [Homo sapiens]
Sequence ID: AAB25641.1Length: 26Number of Matches: 1
Range 1: 2 to 17GenPeptGraphics Next Match Previous Match

Alignment statistics for match #1

| Score | Expect | Identities | Positives | Gaps |
|---|---|---|---|---|
| 26.9 bits(56) | 1.7 | 11/16(69%) | 12/16(75%) | 2/16(12%) |

```
Query  1   CASSSGLAGY--EQYF  14
           CASS GLAG   EQ+F
Sbjct  2   CASSWGLAGARDEQFF  17
```

---

## TCR Heart 7.14 CASSQDRGQYEQYF

myelin basic protein specific T-cell receptor V beta-D beta-J beta, MBP reactive TCR VDJ beta {CDR3 region, clone BM(5)} [human, peripheral blood lymphocytes, HLA phenotype 1, Peptide Partial, 23 aa] [Homo sapiens]
Sequence ID: AAB25666.1Length: 23Number of Matches: 1
Range 1: 2 to 14GenPeptGraphics Next Match Previous Match

Alignment statistics for match #1

| Score | Expect | Identities | Positives | Gaps |
|---|---|---|---|---|
| 31.2 bits(66) | 0.038 | 10/14(71%) | 11/14(78%) | 1/14(7%) |

```
Query  1    CASSQDRGQYEQYF  14
            CASSQ+   YEQYF
Sbjct  2    CASSQE-ASYEQYF  14
```

myelin basic protein specific T-cell receptor V beta-D beta-J beta, MBP reactive TCR VDJ beta {clone SE(14), rearranged CDR3 region} [human, inflammatory brain lesions, HLA phenotype 1, Peptide Partial, 23 aa] [Homo sapiens]
Sequence ID: AAB25648.1Length: 23Number of Matches: 1
Range 1: 2 to 14GenPeptGraphics Next Match Previous Match

Alignment statistics for match #1

| Score | Expect | Identities | Positives | Gaps |
|---|---|---|---|---|
| 26.9 bits(56) | 1.5 | 10/14(71%) | 10/14(71%) | 1/14(7%) |

```
Query  1    CASSQDRGQYEQYF  14
            CASS    G YEQYF
Sbjct  2    CASSTA-GTYEQYF  14
```

myelin basic protein-specific T-cell receptor beta chain VDJ region {clone HOO24} [human, peripheral blood mononuclear cells, Peptide Partial, 25 aa] [Homo sapiens]
Sequence ID: AAB33029.1Length: 25Number of Matches: 1
Range 1: 1 to 16GenPeptGraphics Next Match Previous Match

Alignment statistics for match #1

| Score | Expect | Identities | Positives | Gaps |
|---|---|---|---|---|
| 26.5 bits(55) | 2.4 | 11/16(69%) | 11/16(68%) | 2/16(12%) |

```
Query  1    CASSQ-DRGQ-YEQYF  14
            CASS    RG   YEQYF
Sbjct  1    CASSSLARGASYEQYF  16
```

myelin basic protein specific T-cell receptor V beta-D beta-J beta, MBP reactive TCR VDJ beta {clone SE(5), rearranged CDR3 region} [human, inflammatory brain lesions, HLA phenotype 1, Peptide Partial, 26 aa] [Homo sapiens]

Sequence ID: AAB25639.1Length: 26Number of Matches: 1

Range 1: 2 to 17GenPeptGraphics Next Match Previous Match

Alignment statistics for match #1

| Score | Expect | Identities | Positives | Gaps |
|---|---|---|---|---|
| 26.5 bits(55) | 2.5 | 11/16(69%) | 12/16(75%) | 2/16(12%) |

```
Query  1    CASSQ-DRGQ-YEQYF  14
            CASS   DRGQ   EQ+F
Sbjct  2    CASSRLDRGQGEEQFF  17
```

myelin basic protein (143-168)-specific TCR beta-chain {clone BM3.3} [human, multiple sclerosis patient, T cells, Peptide Partial, 26 aa] [Homo sapiens]

Sequence ID: AAB31427.1Length: 26Number of Matches: 1

Range 1: 1 to 13GenPeptGraphics Next Match Previous Match

Alignment statistics for match #1

| Score | Expect | Identities | Positives | Gaps |
|---|---|---|---|---|
| 25.7 bits(53) | 5.1 | 10/14(71%) | 10/14(71%) | 1/14(7%) |

```
Query  1    CASSQDRGQYEQYF  14
            CASS   RG Y QYF
Sbjct  1    CASSLTRG-YTQYF  13
```

## TCR Heart 7.15 CASSQDLQGRYEQYF

myelin basic protein specific T-cell receptor V beta-D beta-J beta, MBP reactive TCR VDJ beta {CDR3 region, clone BM(5)} [human, peripheral blood lymphocytes, HLA phenotype 1, Peptide Partial, 23 aa] [Homo sapiens]

Sequence ID: AAB25666.1Length: 23Number of Matches: 1

Range 1: 2 to 14GenPeptGraphics Next Match Previous Match

Alignment statistics for match #1

| Score | Expect | Identities | Positives | Gaps |
|---|---|---|---|---|
| 31.6 bits(67) | 0.031 | 10/15(67%) | 11/15(73%) | 2/15(13%) |

```
Query  1    CASSQDLQGRYEQYF  15
            CASSQ+    YEQYF
Sbjct  2    CASSQE--ASYEQYF  14
```

myelin basic protein specific T-cell receptor V beta-D beta-J beta, MBP reactive TCR VDJ beta {clone LJ 1(6), rearranged CDR3 region} [human, brain plaques, HLA phenotype 1, Peptide Partial, 24 aa] [Homo sapiens]

Sequence ID: AAB25632.1Length: 24Number of Matches: 1

Range 1: 2 to 15GenPeptGraphics Next Match Previous Match

Alignment statistics for match #1

| Score | Expect | Identities | Positives | Gaps |
|---|---|---|---|---|
| 26.9 bits(56) | 1.9 | 11/16(69%) | 11/16(68%) | 3/16(18%) |

```
Query  1    CASSQDL-QGRYEQYF  15
            CASS  L  G YEQYF
Sbjct  2    CASS--LVAGIYEQYF  15
```

myelin basic protein specific T-cell receptor V beta-D beta-J beta, MBP reactive TCR VDJ beta {clone SE(14), rearranged CDR3 region} [human, inflammatory brain lesions, HLA phenotype 1, Peptide Partial, 23 aa] [Homo sapiens]

Sequence ID: AAB25648.1Length: 23Number of Matches: 1

Range 1: 2 to 14GenPeptGraphics Next Match Previous Match

Alignment statistics for match #1

| Score | Expect | Identities | Positives | Gaps |
|---|---|---|---|---|
| 26.1 bits(54) | 3.7 | 10/15(67%) | 10/15(66%) | 2/15(13%) |

```
Query  1    CASSQDLQGRYEQYF  15
            CASS    G YEQYF
Sbjct  2    CASSTA--GTYEQYF  14
```

myelin basic protein specific T-cell receptor V beta-D beta-J beta, MBP reactive TCR VDJ beta {clone KL-3(8), rearranged CDR3 region} [human, brain plaques, HLA phenotype 1, Peptide Partial, 25 aa] [Homo sapiens]

Sequence ID: AAB25611.1Length: 25Number of Matches: 1

Range 1: 2 to 16GenPeptGraphics Next Match Previous Match

Alignment statistics for match #1

| Score | Expect | Identities | Positives | Gaps |
|---|---|---|---|---|
| 24.8 bits(51) | 12 | 10/16(63%) | 12/16(75%) | 2/16(12%) |

```
Query  1    CASSQDLQGRY-EQYF  15
            CASS +L G Y EQ+F
Sbjct  2    CASSLELAG-YNEQFF  16
```

## TCR Heart 7.16 CASSQDKNYEQYF

myelin basic protein specific T-cell receptor V beta-D beta-J beta, MBP reactive TCR VDJ beta {CDR3 region, clone BM(5)} [human, peripheral blood lymphocytes, HLA phenotype 1, Peptide Partial, 23 aa] [Homo sapiens]
Sequence ID: AAB25666.1Length: 23Number of Matches: 1
Range 1: 2 to 14GenPeptGraphics Next Match Previous Match

Alignment statistics for match #1

| Score | Expect | Identities | Positives | Gaps |
|---|---|---|---|---|
| 35.4 bits(76) | 8e-04 | 10/13(77%) | 11/13(84%) | 0/13(0%) |

```
Query  1    CASSQDKNYEQYF  13
            CASSQ+   YEQYF
Sbjct  2    CASSQEASYEQYF  14
```

myelin basic protein specific T-cell receptor V beta-D beta-J beta, MBP reactive TCR VDJ beta {clone SE(14), rearranged CDR3 region} [human, inflammatory brain lesions, HLA phenotype 1, Peptide Partial, 23 aa] [Homo sapiens]
Sequence ID: AAB25648.1Length: 23Number of Matches: 1
Range 1: 2 to 14GenPeptGraphics Next Match Previous Match

Alignment statistics for match #1

| Score | Expect | Identities | Positives | Gaps |
|---|---|---|---|---|
| 26.9 bits(56) | 1.3 | 9/13(69%) | 9/13(69%) | 0/13(0%) |

```
Query  1    CASSQDKNYEQYF  13
            CASS    YEQYF
Sbjct  2    CASSTAGTYEQYF  14
```

T-cell receptor beta chain variable region {junctional region} [human, monozygous twin, myelin basic protein-specific T cell line, Peptide Partial, 22 aa] [Homo sapiens]
Sequence ID: AAB37680.1Length: 22Number of Matches: 1
Range 1: 1 to 12GenPeptGraphics Next Match Previous Match

Alignment statistics for match #1

| Score | Expect | Identities | Positives | Gaps |
|---|---|---|---|---|
| 24.4 bits(50) | 12 | 9/15(60%) | 9/15(60%) | 5/15(33%) |

```
Query  1    CAS--SQDKNYEQYF  13
            CAS  S     YEQYF
Sbjct  1    CASFGS---SYEQYF  12
```

myelin basic protein specific T-cell receptor V beta-D beta-J beta, MBP reactive TCR VDJ beta {clone SE(13), rearranged CDR3 region} [human, inflammatory brain lesions, HLA phenotype 1, Peptide Partial, 23 aa] [Homo sapiens]
Sequence ID: AAB25647.1Length: 23Number of Matches: 1
Range 1: 2 to 14GenPeptGraphics Next Match Previous Match

Alignment statistics for match #1

| Score | Expect | Identities | Positives | Gaps |
|---|---|---|---|---|
| 23.1 bits(47) | 35 | 9/17(53%) | 9/17(52%) | 8/17(47%) |

```
Query  1    CASSQDKN----YEQYF  13
            CASS        YEQYF
Sbjct  2    CASS----FIWGYEQYF  14
```

myelin basic protein (143-168)-specific TCR beta-chain {clone BM3.3} [human, multiple sclerosis patient, T cells, Peptide Partial, 26 aa] [Homo sapiens]
Sequence ID: AAB31427.1Length: 26Number of Matches: 1
Range 1: 1 to 13GenPeptGraphics Next Match Previous Match

Alignment statistics for match #1

| Score | Expect | Identities | Positives | Gaps |
|---|---|---|---|---|
| 22.7 bits(46) | 54 | 8/13(62%) | 8/13(61%) | 0/13(0%) |

```
Query  1    CASSQDKNYEQYF  13
            CASS    Y QYF
Sbjct  1    CASSLTRGYTQYF  13
```

## S3: BLAST Results for 150 Healthy TCR for Matches to Multiple Sclerosis TCR

Age-Related Decrease in TCR Repertoire

Dmitriy M. Chudakov

A. Bogdanova, Ilgar Z. Mamedov, Yuriy B. Lebedev and

Staroverov, Dmitriy A. Bolotin, Sergey Lukyanov, Ekaterina

Ekaterina M. Merzlyak, Maria A. Turchaninova, Dmitriy B.

Olga V. Britanova, Ekaterina V. Putintseva, Mikhail Shugay,

http://www.jimmunol.org/content/192/6/2689

doi: 10.4049/jimmunol.1302064

February 2014;

J Immunol 2014; 192:2689-2698; Prepublished online 7

Material

CASSVAGGGETQYF (one 7/10)

myelin basic protein (143-168)-specific TCR beta-chain {clone JA.1.7} [human, multiple sclerosis patient, T cells, Peptide Partial, 27 aa] [Homo sapiens]

Sequence ID: AAB31429.1Length: 27Number of Matches: 1

Range 1: 1 to 14GenPeptGraphics Next Match Previous Match

Alignment statistics for match #1

| Score | Expect | Identities | Positives | Gaps |
|---|---|---|---|---|
| 34.1 bits(73) | 0.002 | 11/14(79%) | 11/14(78%) | 0/14(0%) |

```
Query  1    CASSVAGGGETQYF  14
            CASS   GG ETQYF
Sbjct  1    CASSLDGGEETQYF  14
```

CASSPDSLGTDTQYF (one 7/10)

myelin basic protein specific T-cell receptor V beta-D beta-J beta, MBP reactive TCR VDJ beta {clone SE(9), rearranged CDR3 region} [human, inflammatory brain lesions, HLA phenotype 1, Peptide Partial, 25 aa] [Homo sapiens]

Sequence ID: AAB25643.1Length: 25Number of Matches: 1

Range 1: 2 to 16GenPeptGraphics Next Match Previous Match

Alignment statistics for match #1

| Score | Expect | Identities | Positives | Gaps |
|---|---|---|---|---|
| 29.9 bits(63) | 0.068 | 12/16(75%) | 12/16(75%) | 2/16(12%) |

```
Query   1    CASSPDSLG-TDTQYF  15
             CASS D LG  DTQYF
Sbjct   2    CASSLD-LGKADTQYF  16
```

CASSSPGETQYF (one 7/10)

myelin basic protein specific T-cell receptor V beta-D beta-J beta, MBP reactive TCR VDJ beta {clone LJ 1(2), rearranged CDR3 region} [human, brain plaques, HLA phenotype 1, Peptide Partial, 22 aa] [Homo sapiens]

Sequence ID: AAB25628.1Length: 22Number of Matches: 1

Range 1: 2 to 13GenPeptGraphics Next Match Previous Match

Alignment statistics for match #1

| Score | Expect | Identities | Positives | Gaps |
|---|---|---|---|---|
| 29.5 bits(62) | 0.053 | 9/12(75%) | 9/12(75%) | 0/12(0%) |

```
Query   1    CASSSPGETQYF  12
             CASS   ETQYF
Sbjct   2    CASSDSSETQYF  13
```

CASSPGDYEQYF (one 7/10)

myelin basic protein specific T-cell receptor V beta-D beta-J beta, MBP reactive TCR VDJ beta {clone SE(14), rearranged CDR3 region} [human, inflammatory brain lesions, HLA phenotype 1, Peptide Partial, 23 aa] [Homo sapiens]

Sequence ID: AAB25648.1Length: 23Number of Matches: 1

Range 1: 2 to 14GenPeptGraphics Next Match Previous Match

Alignment statistics for match #1

| Score | Expect | Identities | Positives | Gaps |
|---|---|---|---|---|
| 29.5 bits(62) | 0.056 | 10/13(77%) | 10/13(76%) | 1/13(7%) |

```
Query   1    CASS-PGDYEQYF  12
             CASS  G YEQYF
Sbjct   2    CASSTAGTYEQYF  14
```

CASSFLGGEQYF (one 7/10)

myelin basic protein specific T-cell receptor V beta-D beta-J beta, MBP reactive TCR VDJ beta {clone SE(13), rearranged CDR3 region} [human, inflammatory brain lesions, HLA phenotype 1, Peptide Partial, 23 aa] [Homo sapiens]

Sequence ID: AAB25647.1Length: 23Number of Matches: 1

Range 1: 2 to 14GenPeptGraphics Next Match Previous Match

Alignment statistics for match #1

| Score | Expect | Identities | Positives | Gaps |
|---|---|---|---|---|
| 25.7 bits(53) | 1.6 | 10/13(77%) | 10/13(76%) | 1/13(7%) |

```
Query   1    CASSFL-GGEQYF  12
             CASSF  G EQYF
Sbjct   2    CASSFIWGYEQYF  14
```

CASSPGHRQETQYF (0)

None

CASSPSGVTDTQYF (one 9/10)

myelin basic protein specific T-cell receptor V beta-D beta-J beta, MBP TCR reactive VDJ beta {CDR3 region, muscle infiltrating lymphocytes(5)} [human, muscle infiltrating lymphocytes, HLA phenotype 1, Peptide Partial, 28 aa] [Homo sapiens]

Sequence ID: AAB25653.1Length: 28Number of Matches: 1

Range 1: 2 to 19GenPeptGraphics Next Match Previous Match

Alignment statistics for match #1

| Score | Expect | Identities | Positives | Gaps |
|---|---|---|---|---|
| 31.2 bits(66) | 0.022 | 12/18(67%) | 12/18(66%) | 4/18(22%) |

```
Query  1    CASSP---SG-VTDTQYF  14
            CAS     SG VTDTQYF
Sbjct  2    CASTARRTSGFVTDTQYF  19
```

CASSLDRGSTDTQYF (two 7/10)

myelin basic protein specific T-cell receptor V beta-D beta-J beta, MBP reactive TCR VDJ beta {clone SE(9), rearranged CDR3 region} [human, inflammatory brain lesions, HLA phenotype 1, Peptide Partial, 25 aa] [Homo sapiens]

Sequence ID: AAB25643.1Length: 25Number of Matches: 1

Range 1: 2 to 16GenPeptGraphics Next Match Previous Match

Alignment statistics for match #1

| Score | Expect | Identities | Positives | Gaps |
|---|---|---|---|---|
| 36.3 bits(78) | 3e-04 | 12/15(80%) | 12/15(80%) | 0/15(0%) |

```
Query  1    CASSLDRGSTDTQYF  15
            CASSLD G  DTQYF
Sbjct  2    CASSLDLGKADTQYF  16
```

myelin basic protein (143-168)-specific TCR beta-chain {clone JA.1.7} [human, multiple sclerosis patient, T cells, Peptide Partial, 27 aa] [Homo sapiens]

Sequence ID: AAB31429.1Length: 27Number of Matches: 1

Range 1: 1 to 14GenPeptGraphics Next Match Previous Match

Alignment statistics for match #1

| Score | Expect | Identities | Positives | Gaps |
|---|---|---|---|---|
| 31.6 bits(67) | 0.017 | 11/15(73%) | 12/15(80%) | 1/15(6%) |

```
Query  1    CASSLDRGSTDTQYF  15
            CASSLD G  +TQYF
Sbjct  1    CASSLD-GGEETQYF  14
```

CASSLAGGGNQPQHF (0)

None

CASSLAGPSYNEQFF (one 7/10)

myelin basic protein (84-102)-specific TCR beta-chain {clone NSJL5.7} [human, multiple sclerosis patient, T cells, Peptide Partial, 28 aa] [Homo sapiens]

Sequence ID: AAB31431.1Length: 28Number of Matches: 1

Range 1: 1 to 15GenPeptGraphics Next Match Previous Match

Alignment statistics for match #1

| Score | Expect | Identities | Positives | Gaps |
|---|---|---|---|---|
| 37.5 bits(81) | 1e-04 | 12/15(80%) | 12/15(80%) | 0/15(0%) |

```
Query  1    CASSLAGPSYNEQFF  15
            CASSL   SYNEQFF
Sbjct  1    CASSLTVGSYNEQFF  15
```

CASSLQGQGYEQYF (0)

None

CASSLTGLTYEQYF (one 7/10)

myelin basic protein specific T-cell receptor V beta-D beta-J beta, MBP reactive TCR VDJ beta {clone SE(14), rearranged CDR3 region} [human, inflammatory brain lesions, HLA phenotype 1, Peptide Partial, 23 aa] [Homo sapiens]

Sequence ID: AAB25648.1Length: 23Number of Matches: 1

Range 1: 2 to 14GenPeptGraphics Next Match Previous Match

Alignment statistics for match #1

| Score | Expect | Identities | Positives | Gaps |
|---|---|---|---|---|
| 32.0 bits(68) | 0.008 | 11/14(79%) | 11/14(78%) | 1/14(7%) |

```
Query  1    CASSLTGLTYEQYF  14
            CASS  G TYEQYF
Sbjct  2    CASSTAG-TYEQYF  14
```

CASSSPGRGETQYF (one 7/10)

myelin basic protein specific T-cell receptor V beta-D beta-J beta, MBP reactive TCR VDJ beta {clone KL-1(9), rearranged CDR3 region} [human, brain plaques, HLA phenotype 1, Peptide Partial, 24 aa] [Homo sapiens]

Sequence ID: AAB25600.1Length: 24Number of Matches: 1

Range 1: 2 to 15GenPeptGraphics Next Match Previous Match

Alignment statistics for match #1

| Score | Expect | Identities | Positives | Gaps |
|---|---|---|---|---|
| 29.5 bits(62) | 0.081 | 11/14(79%) | 11/14(78%) | 0/14(0%) |

```
Query  1    CASSSPGRGETQYF  14
            CASSS GR  TQYF
Sbjct  2    CASSSDGRMSTQYF  15
```

CASSLGSAQETQYF (two 7/10)

myelin basic protein specific T-cell receptor V beta-D beta-J beta, MBP reactive TCR VDJ beta {clone KL-3(10), rearranged CDR3 region} [human, brain plaques, HLA phenotype 1, Peptide Partial, 24 aa] [Homo sapiens]

Sequence ID: AAB25613.1Length: 24Number of Matches: 1

Range 1: 2 to 15GenPeptGraphics Next Match Previous Match

Alignment statistics for match #1

| Score | Expect | Identities | Positives | Gaps |
|---|---|---|---|---|
| 38.0 bits(82) | 5e-05 | 11/14(79%) | 12/14(85%) | 0/14(0%) |

```
Query  1    CASSLGSAQETQYF  14
            CASSLG   +ETQYF
Sbjct  2    CASSLGGSEETQYF  15
```

myelin basic protein specific T-cell receptor V beta-D beta-J beta, MBP reactive TCR VDJ beta {clone KL-3(9), rearranged CDR3 region} [human, brain plaques, HLA phenotype 1, Peptide Partial, 25 aa] [Homo sapiens]

Sequence ID: AAB25612.1Length: 25Number of Matches: 1

Range 1: 2 to 16GenPeptGraphics Next Match Previous Match

Alignment statistics for match #1

| Score | Expect | Identities | Positives | Gaps |
|---|---|---|---|---|
| 33.7 bits(72) | 0.002 | 11/15(73%) | 13/15(86%) | 1/15(6%) |

```
Query  1    CASSLG-SAQETQYF  14
            CASSLG S ++TQYF
Sbjct  2    CASSLGGSEEDTQYF  16
```

CASSLQGGRETQYF (one 8/10 and two 7/10)

myelin basic protein (143-168)-specific TCR beta-chain {clone JA.1.7} [human, multiple sclerosis patient, T cells, Peptide Partial, 27 aa] [Homo sapiens]

Sequence ID: AAB31429.1Length: 27Number of Matches: 1

Range 1: 1 to 14GenPeptGraphics Next Match Previous Match

Alignment statistics for match #1

| Score | Expect | Identities | Positives | Gaps |
|---|---|---|---|---|
| 36.3 bits(78) | 2e-04 | 12/14(86%) | 12/14(85%) | 0/14(0%) |

```
Query  1    CASSLQGGRETQYF  14
            CASSL GG ETQYF
Sbjct  1    CASSLDGGEETQYF  14
```

myelin basic protein specific T-cell receptor V beta-D beta-J beta, MBP reactive TCR VDJ beta {clone KL-3(10), rearranged CDR3 region} [human, brain plaques, HLA phenotype 1, Peptide Partial, 24 aa] [Homo sapiens]

Sequence ID: AAB25613.1Length: 24Number of Matches: 1

Range 1: 2 to 15GenPeptGraphics Next Match Previous Match

Alignment statistics for match #1

| Score | Expect | Identities | Positives | Gaps |
|---|---|---|---|---|
| 31.2 bits(66) | 0.018 | 12/15(80%) | 12/15(80%) | 2/15(13%) |

```
Query  1    CASSLQGGR-ETQYF  14
            CASSL GG  ETQYF
Sbjct  2    CASSL-GGSEETQYF  15
```

myelin basic protein specific T-cell receptor V beta-D beta-J beta, MBP reactive TCR VDJ beta {clone KL-3(9), rearranged CDR3 region} [human, brain plaques, HLA phenotype 1, Peptide Partial, 25 aa] [Homo sapiens]

Sequence ID: AAB25612.1Length: 25Number of Matches: 1

Range 1: 2 to 16GenPeptGraphics Next Match Previous Match

Alignment statistics for match #1

| Score | Expect | Identities | Positives | Gaps |
|---|---|---|---|---|
| 30.8 bits(65) | 0.028 | 12/16(75%) | 12/16(75%) | 3/16(18%) |

```
Query  1    CASSLQGGRE--TQYF  14
            CASSL GG E  TQYF
Sbjct  2    CASSL-GGSEEDTQYF  16
```

CASSPGRSSGANVLTF (one 7/10)

myelin basic protein specific T-cell receptor V beta-D beta-J beta, MBP reactive TCR VDJ beta {CDR3 region, clone JO(6)} [human, normal peripheral blood lymphocytes, Peptide Partial, 28 aa] [Homo sapiens]

Sequence ID: AAB25661.1Length: 28Number of Matches: 1

Range 1: 2 to 19GenPeptGraphics Next Match Previous Match

Alignment statistics for match #1

| Score | Expect | Identities | Positives | Gaps |
|---|---|---|---|---|
| 32.0 bits(68) | 0.014 | 13/20(65%) | 13/20(65%) | 6/20(30%) |

```
Query  1    CASSPGRS----SGANVLTF  16
            CASS  RS     GANVLTF
Sbjct  2    CASS--RSQGARTGANVLTF  19
```

CASSQRGGSSYNEQFF (one 7/10)

myelin basic protein-specific T-cell receptor beta chain VDJ region {clone FN2.2} [human, peripheral blood mononuclear cells, Peptide Partial, 25 aa] [Homo sapiens]

Sequence ID: AAB33027.1Length: 25Number of Matches: 1

Range 1: 1 to 16GenPeptGraphics Next Match Previous Match

Alignment statistics for match #1

| Score | Expect | Identities | Positives | Gaps |
|---|---|---|---|---|
| 39.2 bits(85) | 2e-05 | 12/16(75%) | 12/16(75%) | 0/16(0%) |

```
Query  1    CASSQRGGSSYNEQFF  16
            CASS RG   YNEQFF
Sbjct  1    CASSLRGSGTYNEQFF  16
```

CASSYSPPSGIYNEQFF (one 8/10 and one 7/10)

myelin basic protein specific T-cell receptor V beta-D beta-J beta, MBP reactive TCR VDJ beta {clone KL-3(18), rearranged CDR3 region} [human, brain plaques, HLA phenotype 1, Peptide Partial, 22 aa] [Homo sapiens]

Sequence ID: AAB25621.1Length: 22Number of Matches: 1

Range 1: 2 to 13GenPeptGraphics Next Match Previous Match

Alignment statistics for match #1

| Score | Expect | Identities | Positives | Gaps |
|---|---|---|---|---|
| 33.3 bits(71) | 0.003 | 12/16(75%) | 12/16(75%) | 4/16(25%) |

```
Query  1    CASSPGRSSGANVLTF  16
            CASS    SGANVLTF
Sbjct  2    CASS----SGANVLTF  13
```

myelin basic protein specific T-cell receptor V beta-D beta-J beta, MBP reactive TCR VDJ beta {clone KL-3(15), rearranged CDR3 region} [human, brain plaques, HLA phenotype 1, Peptide Partial, 23 aa] [Homo sapiens]

Sequence ID: AAB25618.1Length: 23Number of Matches: 1

Range 1: 2 to 14GenPeptGraphics Next Match Previous Match

Alignment statistics for match #1

| Score | Expect | Identities | Positives | Gaps |
|---|---|---|---|---|
| 34.6 bits(74) | 0.001 | 12/16(75%) | 12/16(75%) | 3/16(18%) |

```
Query  1    CASSPGRSSGANVLTF  16
            CASSP    GANVLTF
Sbjct  2    CASSP---TGANVLTF  14
```

CASSSPGLAGGSSYNEQFF (one 8/10 and one 7/10)

myelin basic protein (84-102)-specific TCR beta-chain {clone NSJL5.7} [human, multiple sclerosis patient, T cells, Peptide Partial, 28 aa] [Homo sapiens]

Sequence ID: AAB31431.1Length: 28Number of Matches: 1

Range 1: 1 to 15GenPeptGraphics Next Match Previous Match

Alignment statistics for match #1

| Score | Expect | Identities | Positives | Gaps |
|---|---|---|---|---|
| 33.3 bits(71) | 0.007 | 13/19(68%) | 13/19(68%) | 4/19(21%) |

```
Query  1    CASSSPGLAGGSSYNEQFF  19
            CASS   L  GS YNEQFF
Sbjct  1    CASS---LTVGS-YNEQFF  15
```

myelin basic protein-specific T-cell receptor beta chain VDJ region {clone FN2.2} [human, peripheral blood mononuclear cells, Peptide Partial, 25 aa] [Homo sapiens]

Sequence ID: AAB33027.1Length: 25Number of Matches: 1

Range 1: 1 to 16GenPeptGraphics Next Match Previous Match

Alignment statistics for match #1

---

| Score | Expect | Identities | Positives | Gaps |
|---|---|---|---|---|
| 32.9 bits(70) | 0.009 | 12/19(63%) | 12/19(63%) | 3/19(15%) |

```
Query  1    CASSSPGLAGGSSYNEQFF  19
            CASS   L G   YNEQFF
Sbjct  1    CASS---LRGSGTYNEQFF  16
```

CASSLGVGEQYF (three 7/10)

myelin basic protein specific T-cell receptor V beta-D beta-J beta, MBP reactive TCR VDJ beta {clone KL-3(9), rearranged CDR3 region} [human, brain plaques, HLA phenotype 1, Peptide Partial, 25 aa] [Homo sapiens]

Sequence ID: AAB25612.1Length: 25Number of Matches: 1

Range 1: 2 to 16GenPeptGraphics Next Match Previous Match

Alignment statistics for match #1

| Score | Expect | Identities | Positives | Gaps |
|---|---|---|---|---|
| 27.4 bits(57) | 0.38 | 10/15(67%) | 10/15(66%) | 3/15(20%) |

```
Query  1    CASSLGVGE---QYF  12
            CASSLG  E   QYF
Sbjct  2    CASSLGGSEEDTQYF  16
```

myelin basic protein (143-168)-specific TCR beta-chain {clone JA.1.7} [human, multiple sclerosis patient, T cells, Peptide Partial, 27 aa] [Homo sapiens]

Sequence ID: AAB31429.1Length: 27Number of Matches: 1

Range 1: 1 to 14GenPeptGraphics Next Match Previous Match

Alignment statistics for match #1

| Score | Expect | Identities | Positives | Gaps |
|---|---|---|---|---|
| 27.4 bits(57) | 0.41 | 10/14(71%) | 10/14(71%) | 2/14(14%) |

```
Query  1    CASSLGVGE--QYF  12
            CASSL  GE  QYF
Sbjct  1    CASSLDGGEETQYF  14
```

Download GenPeptGraphics Next Previous Descriptions

myelin basic protein (84-102)-specific TCR beta-chain {clone NSJL5.7} [human, multiple sclerosis patient, T cells, Peptide Partial, 28 aa] [Homo sapiens]

Sequence ID: AAB31431.1Length: 28Number of Matches: 1

Range 1: 1 to 15GenPeptGraphics Next Match Previous Match

Alignment statistics for match #1

| Score | Expect | Identities | Positives | Gaps |
|---|---|---|---|---|
| 27.4 bits(57) | 0.42 | 10/15(67%) | 11/15(73%) | 3/15(20%) |

```
Query  1    CASSLGVG---EQYF  12
            CASSL VG   EQ+F
Sbjct  1    CASSLTVGSYNEQFF  15
```

CASSLGGGRTDTQYF (two 7/10)

myelin basic protein specific T-cell receptor V beta-D beta-J beta, MBP reactive TCR VDJ beta {clone KL-3(9), rearranged CDR3 region} [human, brain plaques, HLA phenotype 1, Peptide Partial, 25 aa] [Homo sapiens]

Sequence ID: AAB25612.1Length: 25Number of Matches: 1

Range 1: 2 to 16GenPeptGraphics Next Match Previous Match

Alignment statistics for match #1

| Score | Expect | Identities | Positives | Gaps |
|---|---|---|---|---|
| 33.7 bits(72) | 0.002 | 12/15(80%) | 12/15(80%) | 0/15(0%) |

```
Query  1    CASSLGGGRTDTQYF  15
            CASSLGG   DTQYF
Sbjct  2    CASSLGGSEEDTQYF  16
```

myelin basic protein specific T-cell receptor V beta-D beta-J beta, MBP reactive TCR VDJ beta {clone KL-3(10), rearranged CDR3 region} [human, brain plaques, HLA phenotype 1, Peptide Partial, 24 aa] [Homo sapiens]

Sequence ID: AAB25613.1Length: 24Number of Matches: 1

Range 1: 2 to 15GenPeptGraphics Next Match Previous Match

Alignment statistics for match #1

| Score | Expect | Identities | Positives | Gaps |
|---|---|---|---|---|
| 30.8 bits(65) | 0.031 | 11/15(73%) | 12/15(80%) | 1/15(6%) |

```
Query  1    CASSLGGGRTDTQYF  15
            CASSLGG    +TQYF
Sbjct  2    CASSLGGS-EETQYF  15
```

CASSSTGTGAYEQYF (one 7/10)

myelin basic protein-specific T-cell receptor beta chain VDJ region {clone HOO24} [human, peripheral blood mononuclear cells, Peptide Partial, 25 aa] [Homo sapiens]

Sequence ID: AAB33029.1Length: 25Number of Matches: 1

Range 1: 1 to 16GenPeptGraphics Next Match Previous Match

Alignment statistics for match #1

| Score | Expect | Identities | Positives | Gaps |
|---|---|---|---|---|
| 31.2 bits(66) | 0.023 | 12/16(75%) | 12/16(75%) | 1/16(6%) |

```
Query  1    CASSSTGTGA-YEQYF  15
            CASSS    GA YEQYF
Sbjct  1    CASSSLARGASYEQYF  16
```

CASSQDGSSYNEQFF (two 7/10)

myelin basic protein (84-102)-specific TCR beta-chain {clone NSJL5.7} [human, multiple sclerosis patient, T cells, Peptide Partial, 28 aa] [Homo sapiens]

Sequence ID: AAB31431.1Length: 28Number of Matches: 1

Range 1: 1 to 15GenPeptGraphics Next Match Previous Match

Alignment statistics for match #1

| Score | Expect | Identities | Positives | Gaps |
|---|---|---|---|---|
| 32.5 bits(69) | 0.009 | 11/15(73%) | 11/15(73%) | 0/15(0%) |

```
Query  1    CASSQDGSSYNEQFF  15
            CASS    SYNEQFF
Sbjct  1    CASSLTVGSYNEQFF  15
```

myelin basic protein-specific T-cell receptor beta chain VDJ region {clone FN2.2} [human, peripheral blood mononuclear cells, Peptide Partial, 25 aa] [Homo sapiens]

Sequence ID: AAB33027.1Length: 25Number of Matches: 1

Range 1: 1 to 16GenPeptGraphics Next Match Previous Match

Alignment statistics for match #1

| Score | Expect | Identities | Positives | Gaps |
|---|---|---|---|---|
| 31.6 bits(67) | 0.016 | 12/16(75%) | 12/16(75%) | 1/16(6%) |

```
Query  1    CASSQDGS-SYNEQFF  15
            CASS  GS  YNEQFF
Sbjct  1    CASSLRGSGTYNEQFF  16
```

CASSLGAGSTDTQYF (one 8/10)

myelin basic protein specific T-cell receptor V beta-D beta-J beta, MBP reactive TCR VDJ beta {clone KL-3(10), rearranged CDR3 region} [human, brain plaques, HLA phenotype 1, Peptide Partial, 24 aa] [Homo sapiens]

Sequence ID: AAB25613.1Length: 24Number of Matches: 1

Range 1: 2 to 15GenPeptGraphics Next Match Previous Match

Alignment statistics for match #1

| Score | Expect | Identities | Positives | Gaps |
|---|---|---|---|---|
| 34.1 bits(73) | 0.002 | 12/15(80%) | 13/15(86%) | 1/15(6%) |

```
Query  1    CASSLGAGSTDTQYF  15
            CASSLG GS +TQYF
Sbjct  2    CASSLG-GSEETQYF  15
```

CASSLTGTGSYEQYF (one 8/10 and one 7/10)

myelin basic protein (84-102)-specific TCR beta-chain {clone NSJL5.7} [human, multiple sclerosis patient, T cells, Peptide Partial, 28 aa] [Homo sapiens]

Sequence ID: AAB31431.1Length: 28Number of Matches: 1

Range 1: 1 to 15GenPeptGraphics Next Match Previous Match

Alignment statistics for match #1

| Score | Expect | Identities | Positives | Gaps |
|---|---|---|---|---|
| 32.0 bits(68) | 0.012 | 12/16(75%) | 13/16(81%) | 2/16(12%) |

```
Query  1    CASSLTGTGSY-EQYF  15
            CASSLT  GSY EQ+F
Sbjct  1    CASSLT-VGSYNEQFF  15
```

myelin basic protein-specific T-cell receptor beta chain VDJ region {clone FN2.2} [human, peripheral blood mononuclear cells, Peptide Partial, 25 aa] [Homo sapiens]

Sequence ID: AAB33027.1Length: 25Number of Matches: 1

Range 1: 1 to 16GenPeptGraphics Next Match Previous Match

Alignment statistics for match #1

| Score | Expect | Identities | Positives | Gaps |
|---|---|---|---|---|
| 32.0 bits(68) | 0.011 | 11/16(69%) | 12/16(75%) | 1/16(6%) |

```
Query  1    CASSLTGTGSY-EQYF  15
            CASSL G G Y EQ+F
Sbjct  1    CASSLRGSGTYNEQFF  16
```

CASSRGTGNYGYTF (one 7/10)

myelin basic protein specific T-cell receptor V beta-D beta-J beta, MBP reactive TCR VDJ beta {clone KL-3(6), rearranged CDR3 region} [human, brain plaques, HLA phenotype 1, Peptide Partial, 24 aa] [Homo sapiens]

Sequence ID: AAB25609.1Length: 24Number of Matches: 1

Range 1: 2 to 15GenPeptGraphics Next Match Previous Match

Alignment statistics for match #1

| Score | Expect | Identities | Positives | Gaps |
|---|---|---|---|---|
| 30.8 bits(65) | 0.027 | 12/15(80%) | 12/15(80%) | 2/15(13%) |

```
Query  1    CASS-RGTGNYGYTF  14
            CASS RGT  YGYTF
Sbjct  2    CASSLRGTP-YGYTF  15
```

CASSRTGGEETQYF (one 8/10 and one 7/10)

myelin basic protein (143-168)-specific TCR beta-chain {clone JA.1.7} [human, multiple sclerosis patient, T cells, Peptide Partial, 27 aa] [Homo sapiens]

Sequence ID: AAB31429.1Length: 27Number of Matches: 1

Range 1: 1 to 14GenPeptGraphics Next Match Previous Match

Alignment statistics for match #1

| Score | Expect | Identities | Positives | Gaps |
|---|---|---|---|---|
| 35.8 bits(77) | 4e-04 | 12/14(86%) | 12/14(85%) | 0/14(0%) |

```
Query  1    CASSRTGGEETQYF  14
            CASS  GGEETQYF
Sbjct  1    CASSLDGGEETQYF  14
```

myelin basic protein specific T-cell receptor V beta-D beta-J beta, MBP reactive TCR VDJ beta {clone KL-3(10), rearranged CDR3 region} [human, brain plaques, HLA phenotype 1, Peptide Partial, 24 aa] [Homo sapiens]

Sequence ID: AAB25613.1Length: 24Number of Matches: 1

Range 1: 2 to 15GenPeptGraphics Next Match Previous Match

Alignment statistics for match #1

| Score | Expect | Identities | Positives | Gaps |
|---|---|---|---|---|
| 32.0 bits(68) | 0.009 | 11/14(79%) | 11/14(78%) | 0/14(0%) |

```
Query  1    CASSRTGGEETQYF  14
            CASS  G EETQYF
Sbjct  2    CASSLGGSEETQYF  15
```

CASSLDGPLDTQYF (two 7/10)

myelin basic protein (143-168)-specific TCR beta-chain {clone JA.1.7} [human, multiple sclerosis patient, T cells, Peptide Partial, 27 aa] [Homo sapiens]

Sequence ID: AAB31429.1Length: 27Number of Matches: 1

Range 1: 1 to 14GenPeptGraphics Next Match Previous Match

Alignment statistics for match #1

| Score | Expect | Identities | Positives | Gaps |
|---|---|---|---|---|
| 32.9 bits(70) | 0.005 | 11/14(79%) | 12/14(85%) | 0/14(0%) |

```
Query  1    CASSLDGPLDTQYF  14
            CASSLDG   +TQYF
Sbjct  1    CASSLDGGEETQYF  14
```

myelin basic protein specific T-cell receptor V beta-D beta-J beta, MBP reactive TCR VDJ beta {clone SE(9), rearranged CDR3 region} [human, inflammatory brain lesions, HLA phenotype 1, Peptide Partial, 25 aa] [Homo sapiens]

Sequence ID: AAB25643.1Length: 25Number of Matches: 1

Range 1: 2 to 16GenPeptGraphics Next Match Previous Match

Alignment statistics for match #1

| Score | Expect | Identities | Positives | Gaps |
|---|---|---|---|---|
| 32.5 bits(69) | 0.006 | 12/15(80%) | 12/15(80%) | 1/15(6%) |

```
Query  1    CASSLD-GPLDTQYF  14
            CASSLD G  DTQYF
Sbjct  2    CASSLDLGKADTQYF  16
```

CASSLDLSTDTQYF (one 7/10)

myelin basic protein-specific T-cell receptor beta chain VDJ region {clone HOO24} [human, peripheral blood mononuclear cells, Peptide Partial, 25 aa] [Homo sapiens]

Sequence ID: AAB33029.1Length: 25Number of Matches: 1

Range 1: 1 to 16GenPeptGraphics Next Match Previous Match

Alignment statistics for match #1

| Score | Expect | Identities | Positives | Gaps |
|---|---|---|---|---|

30.3 bits(64) 0.041 12/16(75%) 12/16(75%) 2/16(12%)

```
Query  1    CASS-LDR-RSYEQYF  14
            CASS L R  SYEQYF
Sbjct  1    CASSSLARGASYEQYF  16
```

CASSLDRRSYEQYF (one 7/10)

myelin basic protein specific T-cell receptor V beta-D beta-J beta, MBP reactive TCR VDJ beta {clone SE(9), rearranged CDR3 region} [human, inflammatory brain lesions, HLA phenotype 1, Peptide Partial, 25 aa] [Homo sapiens]

Sequence ID: AAB25643.1Length: 25Number of Matches: 1

Range 1: 2 to 16GenPeptGraphics Next Match Previous Match

Alignment statistics for match #1

| Score | Expect | Identities | Positives | Gaps |
|---|---|---|---|---|
| 36.7 bits(79) | 2e-04 | 12/15(80%) | 12/15(80%) | 1/15(6%) |

```
Query  1    CASSLDLS-TDTQYF  14
            CASSLDL   DTQYF
Sbjct  2    CASSLDLGKADTQYF  16
```

CASTRTGTYEQYF (one 7/10)

myelin basic protein specific T-cell receptor V beta-D beta-J beta, MBP reactive TCR VDJ beta {clone SE(14), rearranged CDR3 region} [human, inflammatory brain lesions, HLA phenotype 1, Peptide Partial, 23 aa] [Homo sapiens]

Sequence ID: AAB25648.1Length: 23Number of Matches: 1

Range 1: 2 to 14GenPeptGraphics Next Match Previous Match

Alignment statistics for match #1

| Score | Expect | Identities | Positives | Gaps |
|---|---|---|---|---|
| 34.1 bits(73) | 0.001 | 10/13(77%) | 10/13(76%) | 0/13(0%) |

```
Query  1    CASTRTGTYEQYF  13
            CAS   GTYEQYF
Sbjct  2    CASSTAGTYEQYF  14
```

CASSFLGGETQYF (one 7/10)

myelin basic protein specific T-cell receptor V beta-D beta-J beta, MBP reactive TCR VDJ beta {clone KL-3(10), rearranged CDR3 region} [human, brain plaques, HLA phenotype 1, Peptide Partial, 24 aa] [Homo sapiens]

Sequence ID: AAB25613.1Length: 24Number of Matches: 1

Range 1: 2 to 15GenPeptGraphics Next Match Previous Match

Alignment statistics for match #1

| Score | Expect | Identities | Positives | Gaps |
|---|---|---|---|---|
| 32.0 bits(68) | 0.007 | 12/15(80%) | 12/15(80%) | 3/15(20%) |

```
Query  1    CASSFLGG--ETQYF  13
            CASS LGG  ETQYF
Sbjct  2    CASS-LGGSEETQYF  15
```

CASSLEAVYGYTF (one 7/10)

---

myelin basic protein specific T-cell receptor V beta-D beta-J beta, MBP reactive TCR VDJ beta {clone KL-3(1), rearranged CDR3 region} [human, brain plaques, HLA phenotype 1, Peptide Partial, 24 aa] [Homo sapiens]

Sequence ID: AAB25604.1Length: 24Number of Matches: 1

Range 1: 2 to 15GenPeptGraphics Next Match Previous Match

Alignment statistics for match #1

| Score | Expect | Identities | Positives | Gaps |
|---|---|---|---|---|
| 34.6 bits(74) | 8e-04 | 11/14(79%) | 12/14(85%) | 1/14(7%) |

```
Query  1    CASSLEAV-YGYTF  13
            CASSL+ V YGYTF
Sbjct  2    CASSLDGVPYGYTF  15
```

CASSLGGGGEQYF (three 8/10)

myelin basic protein specific T-cell receptor V beta-D beta-J beta, MBP reactive TCR VDJ beta {clone KL-3(10), rearranged CDR3 region} [human, brain plaques, HLA phenotype 1, Peptide Partial, 24 aa] [Homo sapiens]

Sequence ID: AAB25613.1Length: 24Number of Matches: 1

Range 1: 2 to 15GenPeptGraphics Next Match Previous Match

Alignment statistics for match #1

| Score | Expect | Identities | Positives | Gaps |
|---|---|---|---|---|
| 31.2 bits(66) | 0.016 | 11/14(79%) | 11/14(78%) | 1/14(7%) |

```
Query  1    CASSLGGGGE-QYF  13
            CASSLGG   E QYF
Sbjct  2    CASSLGGSEETQYF  15
```

myelin basic protein specific T-cell receptor V beta-D beta-J beta, MBP reactive TCR VDJ beta {clone KL-3(9), rearranged CDR3 region} [human, brain plaques, HLA phenotype 1, Peptide Partial, 25 aa] [Homo sapiens]

Sequence ID: AAB25612.1Length: 25Number of Matches: 1

Range 1: 2 to 16GenPeptGraphics Next Match Previous Match

Alignment statistics for match #1

| Score | Expect | Identities | Positives | Gaps |
|---|---|---|---|---|
| 30.8 bits(65) | 0.024 | 11/15(73%) | 11/15(73%) | 2/15(13%) |

```
Query  1    CASSLGGGGE--QYF  13
            CASSLGG   E  QYF
Sbjct  2    CASSLGGSEEDTQYF  16
```

myelin basic protein (143-168)-specific TCR beta-chain {clone JA.1.7} [human, multiple sclerosis patient, T cells, Peptide Partial, 27 aa] [Homo sapiens]

Sequence ID: AAB31429.1Length: 27Number of Matches: 1

Range 1: 1 to 14GenPeptGraphics Next Match Previous Match

Alignment statistics for match #1

| Score | Expect | Identities | Positives | Gaps |
|---|---|---|---|---|
| 30.8 bits(65) | 0.026 | 11/14(79%) | 11/14(78%) | 1/14(7%) |

```
Query  1    CASSLGGGGE-QYF  13
            CASSL GG E QYF
Sbjct  1    CASSLDGGEETQYF  14
```

CASSPGAGNEQFF (one 7/10)

myelin basic protein (84-102)-specific TCR beta-chain {clone NB17.8} [human, multiple sclerosis patient, T cells, Peptide Partial, 27 aa] [Homo sapiens]

Sequence ID: AAB31433.1Length: 27Number of Matches: 1

Range 1: 1 to 14GenPeptGraphics Next Match Previous Match

Alignment statistics for match #1

| Score | Expect | Identities | Positives | Gaps |
|---|---|---|---|---|
| 27.4 bits(57) | 0.48 | 11/15(73%) | 11/15(73%) | 3/15(20%) |

```
Query  1    CASS--PGAGNEQFF  13
            CASS  P   GNEQFF
Sbjct  1    CASSYSP-LGNEQFF  14
```

CASSPLAGSSYNEQFF (one 8/10 and two 7/10)

myelin basic protein-specific T-cell receptor beta chain VDJ region {clone FN2.2} [human, peripheral blood mononuclear cells, Peptide Partial, 25 aa] [Homo sapiens]

Sequence ID: AAB33027.1Length: 25Number of Matches: 1

Range 1: 1 to 16GenPeptGraphics Next Match Previous Match

Alignment statistics for match #1

| Score | Expect | Identities | Positives | Gaps |
|---|---|---|---|---|
| 33.7 bits(72) | 0.003 | 13/17(76%) | 13/17(76%) | 2/17(11%) |

```
Query  1    CASSPLAGS-SYNEQFF  16
            CASS L GS  YNEQFF
Sbjct  1    CASS-LRGSGTYNEQFF  16
```

myelin basic protein (84-102)-specific TCR beta-chain {clone NSJL5.7} [human, multiple sclerosis patient, T cells, Peptide Partial, 28 aa] [Homo sapiens]

Sequence ID: AAB31431.1Length: 28Number of Matches: 1

Range 1: 1 to 15GenPeptGraphics Next Match Previous Match

Alignment statistics for match #1

| Score | Expect | Identities | Positives | Gaps |
|---|---|---|---|---|
| 35.0 bits(75) | 0.001 | 12/16(75%) | 12/16(75%) | 1/16(6%) |

```
Query  1    CASSPLAGSSYNEQFF  16
            CASS L    SYNEQFF
Sbjct  1    CASS-LTVGSYNEQFF  15
```

myelin basic protein specific T-cell receptor V beta-D beta-J beta, MBP reactive TCR VDJ beta {clone KL-3(8), rearranged CDR3 region} [human, brain plaques, HLA phenotype 1, Peptide Partial, 25 aa] [Homo sapiens]

Sequence ID: AAB25611.1Length: 25Number of Matches: 1

Range 1: 2 to 16GenPeptGraphics Next Match Previous Match

Alignment statistics for match #1

| Score | Expect | Identities | Positives | Gaps |
|---|---|---|---|---|
| 34.1 bits(73) | 0.002 | 13/17(76%) | 13/17(76%) | 3/17(17%) |

```
Query  1    CASS-PLAGSSYNEQFF  16
            CASS  LAG   YNEQFF
Sbjct  2    CASSLELAG--YNEQFF  16
```

CASSLGLAGNQETQYF (two 7/10)

myelin basic protein specific T-cell receptor V beta-D beta-J beta, MBP reactive TCR VDJ beta {clone KL-3(13), rearranged CDR3 region} [human, brain plaques, HLA phenotype 1, Peptide Partial, 26 aa] [Homo sapiens]

Sequence ID: AAB25616.1Length: 26Number of Matches: 1

Range 1: 2 to 17GenPeptGraphics Next Match Previous Match

Alignment statistics for match #1

| Score | Expect | Identities | Positives | Gaps |
|---|---|---|---|---|
| 31.6 bits(67) | 0.019 | 12/17(71%) | 12/17(70%) | 2/17(11%) |

```
Query  1    CASSLGLAGN-QETQYF  16
            CASSL   G  QETQYF
Sbjct  2    CASSLA-SGTLQETQYF  17
```

myelin basic protein specific T-cell receptor V beta-D beta-J beta, MBP reactive TCR VDJ beta {clone SE(9), rearranged CDR3 region} [human, inflammatory brain lesions, HLA phenotype 1, Peptide Partial, 25 aa] [Homo sapiens]

Sequence ID: AAB25643.1Length: 25Number of Matches: 1

Range 1: 2 to 16GenPeptGraphics Next Match Previous Match

Alignment statistics for match #1

| Score | Expect | Identities | Positives | Gaps |
|---|---|---|---|---|
| 31.2 bits(66) | 0.026 | 11/16(69%) | 12/16(75%) | 1/16(6%) |

```
Query  1    CASSLGLAGNQETQYF  16
            CASSL L G  +TQYF
Sbjct  2    CASSLDL-GKADTQYF  16
```

CASSLSGGSSYNEQFF (one 8/10 and 7/10)

myelin basic protein (84-102)-specific TCR beta-chain {clone NSJL5.7} [human, multiple sclerosis patient, T cells, Peptide Partial, 28 aa] [Homo sapiens]

Sequence ID: AAB31431.1Length: 28Number of Matches: 1

Range 1: 1 to 15GenPeptGraphics Next Match Previous Match

Alignment statistics for match #1

| Score | Expect | Identities | Positives | Gaps |
|---|---|---|---|---|
| 38.8 bits(84) | 4e-05 | 13/16(81%) | 13/16(81%) | 1/16(6%) |

```
Query  1    CASSLSGGSSYNEQFF  16
            CASSL  GS YNEQFF
Sbjct  1    CASSLTVGS-YNEQFF  15
```

myelin basic protein-specific T-cell receptor beta chain VDJ region {clone FN2.2} [human, peripheral blood mononuclear cells, Peptide Partial, 25 aa] [Homo sapiens]

Sequence ID: AAB33027.1Length: 25Number of Matches: 1

Range 1: 1 to 16GenPeptGraphics Next Match Previous Match

Alignment statistics for match #1

| Score | Expect | Identities | Positives | Gaps |
|---|---|---|---|---|
| 39.7 bits(86) | 2e-05 | 12/16(75%) | 12/16(75%) | 0/16(0%) |

```
Query  1    CASSLSGGSSYNEQFF  16
            CASSL G   YNEQFF
Sbjct  1    CASSLRGSGTYNEQFF  16
```

CASSSLAGGSYNEQFF (two 8/10 and one 7/10)

myelin basic protein (84-102)-specific TCR beta-chain {clone NSJL5.7} [human, multiple sclerosis patient, T cells, Peptide Partial, 28 aa] [Homo sapiens]

Sequence ID: AAB31431.1Length: 28Number of Matches: 1

Range 1: 1 to 15GenPeptGraphics Next Match Previous Match

Alignment statistics for match #1

| Score | Expect | Identities | Positives | Gaps |
|---|---|---|---|---|
| 38.4 bits(83) | 6e-05 | 13/16(81%) | 13/16(81%) | 1/16(6%) |

```
Query  1    CASSSLAGGSYNEQFF  16
            CASS L  GSYNEQFF
Sbjct  1    CASS-LTVGSYNEQFF  15
```

myelin basic protein-specific T-cell receptor beta chain VDJ region {clone HOO24} [human, peripheral blood mononuclear cells, Peptide Partial, 25 aa] [Homo sapiens]

Sequence ID: AAB33029.1Length: 25Number of Matches: 1

Range 1: 1 to 16GenPeptGraphics Next Match Previous Match

Alignment statistics for match #1

| Score | Expect | Identities | Positives | Gaps |
|---|---|---|---|---|
| 34.6 bits(74) | 0.001 | 13/17(76%) | 14/17(82%) | 2/17(11%) |

```
Query  1    CASSSLA-GGSYNEQFF  16
            CASSSLA G SY EQ+F
Sbjct  1    CASSSLARGASY-EQYF  16
```

myelin basic protein specific T-cell receptor V beta-D beta-J beta, MBP reactive TCR VDJ beta {clone KL-3(8), rearranged CDR3 region} [human, brain plaques, HLA phenotype 1, Peptide Partial, 25 aa] [Homo sapiens]

Sequence ID: AAB25611.1Length: 25Number of Matches: 1

Range 1: 2 to 16GenPeptGraphics Next Match Previous Match

Alignment statistics for match #1

| Score | Expect | Identities | Positives | Gaps |
|---|---|---|---|---|
| 34.6 bits(74) | 0.001 | 13/17(76%) | 13/17(76%) | 3/17(17%) |

```
Query  1    CASS-SLAGGSYNEQFF  16
            CASS  LAG  YNEQFF
Sbjct  2    CASSLELAG--YNEQFF  16
```

CASSLGGRSSYNEQFF (one 7/10)

myelin basic protein-specific T-cell receptor beta chain VDJ region {clone FN2.2} [human, peripheral blood mononuclear cells, Peptide Partial, 25 aa] [Homo sapiens]

Sequence ID: AAB33027.1Length: 25Number of Matches: 1

Range 1: 1 to 16GenPeptGraphics Next Match Previous Match

Alignment statistics for match #1

| Score | Expect | Identities | Positives | Gaps |
|---|---|---|---|---|
| 36.7 bits(79) | 2e-04 | 12/16(75%) | 12/16(75%) | 0/16(0%) |

```
Query  1    CASSLGGRSSYNEQFF  16
            CASSL G   YNEQFF
Sbjct  1    CASSLRGSGTYNEQFF  16
```

CASSLGAGATYEQYF (two 7/10)

myelin basic protein specific T-cell receptor V beta-D beta-J beta, MBP reactive TCR VDJ beta {clone LJ 1(6), rearranged CDR3 region} [human, brain plaques, HLA phenotype 1, Peptide Partial, 24 aa] [Homo sapiens]

Sequence ID: AAB25632.1Length: 24Number of Matches: 1

Range 1: 2 to 15GenPeptGraphics Next Match Previous Match

Alignment statistics for match #1

| Score | Expect | Identities | Positives | Gaps |
|---|---|---|---|---|
| 35.0 bits(75) | 7e-04 | 12/15(80%) | 12/15(80%) | 1/15(6%) |

```
Query  1    CASSLGAGATYEQYF  15
            CASSL AG  YEQYF
Sbjct  2    CASSLVAG-IYEQYF  15
```

myelin basic protein-specific T-cell receptor beta chain VDJ region {clone HOO24} [human, peripheral blood mononuclear cells, Peptide Partial, 25 aa] [Homo sapiens]

Sequence ID: AAB33029.1Length: 25Number of Matches: 1

Range 1: 1 to 16GenPeptGraphics Next Match Previous Match

Alignment statistics for match #1

| Score | Expect | Identities | Positives | Gaps |
|---|---|---|---|---|

34.1 bits(73) 0.002 12/16(75%) 12/16(75%) 1/16(6%)

```
Query  1    CASS-LGAGATYEQYF  15
            CASS L  GA YEQYF
Sbjct  1    CASSSLARGASYEQYF  16
```

CASSLGGSGTDTQYF (two 8/10)

myelin basic protein specific T-cell receptor V beta-D beta-J beta, MBP reactive TCR VDJ beta {clone KL-3(9), rearranged CDR3 region} [human, brain plaques, HLA phenotype 1, Peptide Partial, 25 aa] [Homo sapiens]

Sequence ID: AAB25612.1Length: 25Number of Matches: 1

Range 1: 2 to 16GenPeptGraphics Next Match Previous Match

Alignment statistics for match #1

| Score | Expect | Identities | Positives | Gaps |
|---|---|---|---|---|
| 39.2 bits(85) | 2e-05 | 13/15(87%) | 13/15(86%) | 0/15(0%) |

```
Query  1    CASSLGGSGTDTQYF  15
            CASSLGGS  DTQYF
Sbjct  2    CASSLGGSEEDTQYF  16
```

myelin basic protein specific T-cell receptor V beta-D beta-J beta, MBP reactive TCR VDJ beta {clone KL-3(10), rearranged CDR3 region} [human, brain plaques, HLA phenotype 1, Peptide Partial, 24 aa] [Homo sapiens]

Sequence ID: AAB25613.1Length: 24Number of Matches: 1

Range 1: 2 to 15GenPeptGraphics Next Match Previous Match

Alignment statistics for match #1

| Score | Expect | Identities | Positives | Gaps |
|---|---|---|---|---|
| 35.0 bits(75) | 7e-04 | 12/15(80%) | 13/15(86%) | 1/15(6%) |

```
Query  1    CASSLGGSGTDTQYF  15
            CASSLGGS  +TQYF
Sbjct  2    CASSLGGSE-ETQYF  15
```

CASSLRDRGQETQYF (two 7/10)

myelin basic protein (143-168)-specific TCR beta-chain {clone JA.1.7} [human, multiple sclerosis patient, T cells, Peptide Partial, 27 aa] [Homo sapiens]

Sequence ID: AAB31429.1Length: 27Number of Matches: 1

Range 1: 1 to 14GenPeptGraphics Next Match Previous Match

Alignment statistics for match #1

| Score | Expect | Identities | Positives | Gaps |
|---|---|---|---|---|
| 34.1 bits(73) | 0.002 | 12/15(80%) | 13/15(86%) | 1/15(6%) |

```
Query  1    CASSLRDRGQETQYF  15
            CASSL D G+ETQYF
Sbjct  1    CASSL-DGGEETQYF  14
```

myelin basic protein specific T-cell receptor V beta-D beta-J beta, MBP reactive TCR VDJ beta {CDR3 region, clone JO(4)} [human, normal peripheral blood lymphocytes, Peptide Partial, 25 aa] [Homo sapiens]

Sequence ID: AAB25659.1Length: 25Number of Matches: 1

Range 1: 2 to 16GenPeptGraphics Next Match Previous Match

Alignment statistics for match #1

| Score | Expect | Identities | Positives | Gaps |
|---|---|---|---|---|
| 31.6 bits(67) | 0.016 | 12/17(71%) | 12/17(70%) | 4/17(23%) |

```
Query  1    CASSLRDR--GQETQYF  15
            CASS  D   GQETQYF
Sbjct  2    CASS--DSPSGQETQYF  16
```

CASSLRQGGSYEQYF (two 7/10)

myelin basic protein-specific T-cell receptor beta chain VDJ region {clone HOO24} [human, peripheral blood mononuclear cells, Peptide Partial, 25 aa] [Homo sapiens]

Sequence ID: AAB33029.1Length: 25Number of Matches: 1

Range 1: 1 to 16GenPeptGraphics Next Match Previous Match

Alignment statistics for match #1

| Score | Expect | Identities | Positives | Gaps |
|---|---|---|---|---|
| 33.3 bits(71) | 0.004 | 12/16(75%) | 12/16(75%) | 1/16(6%) |

```
Query  1    CASS-LRQGGSYEQYF  15
            CASS L  G SYEQYF
Sbjct  1    CASSSLARGASYEQYF  16
```

myelin basic protein specific T-cell receptor V beta-D beta-J beta, MBP reactive TCR VDJ beta {CDR3 region, clone BM(5)} [human, peripheral blood lymphocytes, HLA phenotype 1, Peptide Partial, 23 aa] [Homo sapiens]

Sequence ID: AAB25666.1Length: 23Number of Matches: 1

Range 1: 2 to 14GenPeptGraphics Next Match Previous Match

Alignment statistics for match #1

| Score | Expect | Identities | Positives | Gaps |
|---|---|---|---|---|
| 32.9 bits(70) | 0.005 | 11/15(73%) | 11/15(73%) | 2/15(13%) |

```
Query  1    CASSLRQGGSYEQYF  15
            CASS  Q  SYEQYF
Sbjct  2    CASS--QEASYEQYF  14
```

CASSPGQGVSYEQYF (two 7/10)

myelin basic protein specific T-cell receptor V beta-D beta-J beta, MBP reactive TCR VDJ beta {CDR3 region, clone BM(5)} [human, peripheral blood lymphocytes, HLA phenotype 1, Peptide Partial, 23 aa] [Homo sapiens]

Sequence ID: AAB25666.1Length: 23Number of Matches: 1

Range 1: 2 to 14GenPeptGraphics Next Match Previous Match

Alignment statistics for match #1

| Score | Expect | Identities | Positives | Gaps |
|---|---|---|---|---|
| 32.9 bits(70) | 0.005 | 11/15(73%) | 11/15(73%) | 2/15(13%) |

```
Query  1    CASSPGQGVSYEQYF  15
            CASS  Q  SYEQYF
Sbjct  2    CASS--QEASYEQYF  14
```

myelin basic protein-specific T-cell receptor beta chain VDJ region {clone HOO24} [human, peripheral blood mononuclear cells, Peptide Partial, 25 aa] [Homo sapiens]

Sequence ID: AAB33029.1Length: 25Number of Matches: 1

Range 1: 1 to 16GenPeptGraphics Next Match Previous Match

Alignment statistics for match #1

| Score | Expect | Identities | Positives | Gaps |
|---|---|---|---|---|
| 31.6 bits(67) | 0.016 | 11/16(69%) | 11/16(68%) | 1/16(6%) |

```
Query  1    CASSP-GQGVSYEQYF  15
            CASS    G SYEQYF
Sbjct  1    CASSSLARGASYEQYF  16
```

CASSLGALSYEQYF (one 8/10 and one 7/10)

myelin basic protein-specific T-cell receptor beta chain VDJ region {clone HOO24} [human, peripheral blood mononuclear cells, Peptide Partial, 25 aa] [Homo sapiens]

Sequence ID: AAB33029.1Length: 25Number of Matches: 1

Range 1: 1 to 16GenPeptGraphics Next Match Previous Match

Alignment statistics for match #1

| Score | Expect | Identities | Positives | Gaps |
|---|---|---|---|---|
| 31.6 bits(67) | 0.013 | 13/17(76%) | 13/17(76%) | 4/17(23%) |

```
Query  1    CASS-L--GALSYEQYF  14
            CASS L  GA SYEQYF
Sbjct  1    CASSSLARGA-SYEQYF  16
```

myelin basic protein specific T-cell receptor V beta-D beta-J beta, MBP reactive TCR VDJ beta {CDR3 region, clone BM(5)} [human, peripheral blood lymphocytes, HLA phenotype 1, Peptide Partial, 23 aa] [Homo sapiens]

Sequence ID: AAB25666.1Length: 23Number of Matches: 1

Range 1: 2 to 14GenPeptGraphics Next Match Previous Match

Alignment statistics for match #1

| Score | Expect | Identities | Positives | Gaps |
|---|---|---|---|---|
| 31.2 bits(66) | 0.017 | 11/14(79%) | 11/14(78%) | 1/14(7%) |

```
Query  1    CASSLGALSYEQYF  14
            CASS  A SYEQYF
Sbjct  2    CASSQEA-SYEQYF  14
```

CASSLGGPADTQYF (two 7/10)

myelin basic protein specific T-cell receptor V beta-D beta-J beta, MBP reactive TCR VDJ beta {clone KL-3(10), rearranged CDR3 region} [human, brain plaques, HLA phenotype 1, Peptide Partial, 24 aa] [Homo sapiens]

Sequence ID: AAB25613.1Length: 24Number of Matches: 1

Range 1: 2 to 15GenPeptGraphics Next Match Previous Match

Alignment statistics for match #1

| Score | Expect | Identities | Positives | Gaps |
|---|---|---|---|---|
| 36.7 bits(79) | 1e-04 | 11/14(79%) | 12/14(85%) | 0/14(0%) |

```
Query  1    CASSLGGPADTQYF  14
            CASSLGG   +TQYF
Sbjct  2    CASSLGGSEETQYF  15
```

myelin basic protein specific T-cell receptor V beta-D beta-J beta, MBP reactive TCR VDJ beta {clone KL-3(9), rearranged CDR3 region} [human, brain plaques, HLA phenotype 1, Peptide Partial, 25 aa] [Homo sapiens]

Sequence ID: AAB25612.1Length: 25Number of Matches: 1

Range 1: 2 to 16GenPeptGraphics Next Match Previous Match

Alignment statistics for match #1

| Score | Expect | Identities | Positives | Gaps |
|---|---|---|---|---|
| 35.0 bits(75) | 7e-04 | 12/15(80%) | 12/15(80%) | 1/15(6%) |

```
Query  1    CASSLGGP-ADTQYF  14
            CASSLGG    DTQYF
Sbjct  2    CASSLGGSEEDTQYF  16
```

CASSLGQGHYEQYF (one 7/10)

myelin basic protein specific T-cell receptor V beta-D beta-J beta, MBP reactive TCR VDJ beta {clone LJ 1(6), rearranged CDR3 region} [human, brain plaques, HLA phenotype 1, Peptide Partial, 24 aa] [Homo sapiens]

Sequence ID: AAB25632.1Length: 24Number of Matches: 1

Range 1: 2 to 15GenPeptGraphics Next Match Previous Match

Alignment statistics for match #1

| Score | Expect | Identities | Positives | Gaps |
|---|---|---|---|---|
| 32.0 bits(68) | 0.009 | 11/14(79%) | 11/14(78%) | 0/14(0%) |

```
Query  1    CASSLGQGHYEQYF  14
            CASSL  G YEQYF
Sbjct  2    CASSLVAGIYEQYF  15
```

CASSLGTARETQYF (two 7/10)

myelin basic protein specific T-cell receptor V beta-D beta-J beta, MBP reactive TCR VDJ beta {clone KL-3(13), rearranged CDR3 region} [human, brain plaques, HLA phenotype 1, Peptide Partial, 26 aa] [Homo sapiens]

Sequence ID: AAB25616.1Length: 26Number of Matches: 1

Range 1: 2 to 17GenPeptGraphics Next Match Previous Match

Alignment statistics for match #1

| Score | Expect | Identities | Positives | Gaps |
|---|---|---|---|---|
| 33.3 bits(71) | 0.003 | 12/16(75%) | 12/16(75%) | 2/16(12%) |

```
Query  1    CASSL--GTARETQYF  14
            CASSL  GT   ETQYF
```

```
Sbjct  2   CASSLASGTLQETQYF  17
```

myelin basic protein specific T-cell receptor V beta-D beta-J beta, MBP reactive TCR VDJ beta {clone KL-3(10), rearranged CDR3 region} [human, brain plaques, HLA phenotype 1, Peptide Partial, 24 aa] [Homo sapiens]

Sequence ID: AAB25613.1Length: 24Number of Matches: 1

Range 1: 2 to 15GenPeptGraphics Next Match Previous Match

Alignment statistics for match #1

| Score | Expect | Identities | Positives | Gaps |
|---|---|---|---|---|
| 32.0 bits(68) | 0.009 | 11/14(79%) | 11/14(78%) | 0/14(0%) |

```
Query  1   CASSLGTARETQYF  14
           CASSLG   ETQYF
Sbjct  2   CASSLGGSEETQYF  15
```

CASSLVGQNYGYTF (three 7/10)

myelin basic protein specific T-cell receptor V beta-D beta-J beta, MBP reactive TCR VDJ beta {clone KL-3(4), rearranged CDR3 region} [human, brain plaques, HLA phenotype 1, Peptide Partial, 25 aa] [Homo sapiens]

Sequence ID: AAB25607.1Length: 25Number of Matches: 1

Range 1: 2 to 16GenPeptGraphics Next Match Previous Match

Alignment statistics for match #1

| Score | Expect | Identities | Positives | Gaps |
|---|---|---|---|---|
| 35.0 bits(75) | 7e-04 | 12/15(80%) | 12/15(80%) | 1/15(6%) |

```
Query  1   CASSLVGQN-YGYTF  14
           CASSLVG   YGYTF
Sbjct  2   CASSLVGRGPYGYTF  16
```

myelin basic protein specific T-cell receptor V beta-D beta-J beta, MBP reactive TCR VDJ beta {clone KL-3(6), rearranged CDR3 region} [human, brain plaques, HLA phenotype 1, Peptide Partial, 24 aa] [Homo sapiens]

Sequence ID: AAB25609.1Length: 24Number of Matches: 1

Range 1: 2 to 15GenPeptGraphics Next Match Previous Match

Alignment statistics for match #1

| Score | Expect | Identities | Positives | Gaps |
|---|---|---|---|---|
| 30.3 bits(64) | 0.039 | 11/14(79%) | 11/14(78%) | 0/14(0%) |

```
Query  1   CASSLVGQNYGYTF  14
           CASSL G  YGYTF
Sbjct  2   CASSLRGTPYGYTF  15
```

myelin basic protein specific T-cell receptor V beta-D beta-J beta, MBP reactive TCR VDJ beta {clone KL-3(1), rearranged CDR3 region} [human, brain plaques, HLA phenotype 1, Peptide Partial, 24 aa] [Homo sapiens]

Sequence ID: AAB25604.1Length: 24Number of Matches: 1

Range 1: 2 to 15GenPeptGraphics Next Match Previous Match

Alignment statistics for match #1

| Score | Expect | Identities | Positives | Gaps |
|---|---|---|---|---|
| 29.5 bits(62) | 0.081 | 11/14(79%) | 11/14(78%) | 0/14(0%) |

```
Query  1    CASSLVGQNYGYTF  14
            CASSL G  YGYTF
Sbjct  2    CASSLDGVPYGYTF  15
```

CASSPGTSGDNEQFF (0)
None
CASSPASGGYEQYF (one 7/10)
myelin basic protein specific T-cell receptor V beta-D beta-J beta, MBP reactive TCR VDJ beta {clone SE(14), rearranged CDR3 region} [human, inflammatory brain lesions, HLA phenotype 1, Peptide Partial, 23 aa] [Homo sapiens]
Sequence ID: AAB25648.1Length: 23Number of Matches: 1
Range 1: 2 to 14GenPeptGraphics Next Match Previous Match

Alignment statistics for match #1

| Score | Expect | Identities | Positives | Gaps |
|---|---|---|---|---|
| 30.8 bits(65) | 0.025 | 11/14(79%) | 11/14(78%) | 1/14(7%) |

```
Query  1    CASSPASGGYEQYF  14
            CASS A G YEQYF
Sbjct  2    CASSTA-GTYEQYF  14
```

CASSPGTGPYGYTF (two 8/10)
myelin basic protein specific T-cell receptor V beta-D beta-J beta, MBP reactive TCR VDJ beta {clone KL-3(4), rearranged CDR3 region} [human, brain plaques, HLA phenotype 1, Peptide Partial, 25 aa] [Homo sapiens]
Sequence ID: AAB25607.1Length: 25Number of Matches: 1
Range 1: 2 to 16GenPeptGraphics Next Match Previous Match

Alignment statistics for match #1

| Score | Expect | Identities | Positives | Gaps |
|---|---|---|---|---|
| 32.0 bits(68) | 0.009 | 12/15(80%) | 12/15(80%) | 1/15(6%) |

```
Query  1    CASS-PGTGPYGYTF  14
            CASS  G GPYGYTF
Sbjct  2    CASSLVGRGPYGYTF  16
```

myelin basic protein specific T-cell receptor V beta-D beta-J beta, MBP reactive TCR VDJ beta {clone KL-1(1), rearranged CDR3 region} [human, brain plaques, HLA phenotype 1, Peptide Partial, 23 aa] [Homo sapiens]
Sequence ID: AAB25592.1Length: 23Number of Matches: 1
Range 1: 2 to 13GenPeptGraphics Next Match Previous Match

Alignment statistics for match #1

| Score | Expect | Identities | Positives | Gaps |
|---|---|---|---|---|
| 29.9 bits(63) | 0.053 | 11/13(85%) | 11/13(84%) | 2/13(15%) |

```
Query  1    CASS-PGTGPYGY  12
```

```
             CASS PGT PYGY
Sbjct  2    CASSLPGT-PYGY  13
```

CASSPNRGPYEQYF (one 7/10)

myelin basic protein-specific T-cell receptor beta chain VDJ region {clone HOO24} [human, peripheral blood mononuclear cells, Peptide Partial, 25 aa] [Homo sapiens]

Sequence ID: AAB33029.1Length: 25Number of Matches: 1

Range 1: 1 to 16GenPeptGraphics Next Match Previous Match

Alignment statistics for match #1

| Score | Expect | Identities | Positives | Gaps |
|---|---|---|---|---|
| 28.2 bits(59) | 0.25 | 11/16(69%) | 11/16(68%) | 2/16(12%) |

```
Query  1    CASSP-NRGP-YEQYF  14
            CASS   RG  YEQYF
Sbjct  1    CASSSLARGASYEQYF  16
```

CASSPTGERSPLHF (one 7/10)

myelin basic protein (143-168)-specific TCR beta-chain {clone JA.1.7} [human, multiple sclerosis patient, T cells, Peptide Partial, 28 aa] [Homo sapiens]

Sequence ID: AAB31430.1Length: 28Number of Matches: 1

Range 1: 1 to 15GenPeptGraphics Next Match Previous Match

Alignment statistics for match #1

| Score | Expect | Identities | Positives | Gaps |
|---|---|---|---|---|
| 31.6 bits(67) | 0.015 | 12/15(80%) | 12/15(80%) | 1/15(6%) |

```
Query  1    CASSP-TGERSPLHF  14
            CASSP TG  SPLHF
Sbjct  1    CASSPMTGRWSPLHF  15
```

CASSPWTGGYEQYF (one 7/10)

myelin basic protein specific T-cell receptor V beta-D beta-J beta, MBP reactive TCR VDJ beta {clone SE(14), rearranged CDR3 region} [human, inflammatory brain lesions, HLA phenotype 1, Peptide Partial, 23 aa] [Homo sapiens]

Sequence ID: AAB25648.1Length: 23Number of Matches: 1

Range 1: 2 to 14GenPeptGraphics Next Match Previous Match

Alignment statistics for match #1

| Score | Expect | Identities | Positives | Gaps |
|---|---|---|---|---|
| 29.9 bits(63) | 0.053 | 11/15(73%) | 11/15(73%) | 3/15(20%) |

```
Query  1    CASSPWTGG-YEQYF  14
            CASS  T G YEQYF
Sbjct  2    CASS--TAGTYEQYF  14
```

CASSSTASTDTQYF (0)

None

CASSTGTANYGYTF (one 7/10)

myelin basic protein specific T-cell receptor V beta-D beta-J beta, MBP reactive TCR VDJ beta {clone KL-3(6), rearranged CDR3 region} [human, brain plaques, HLA phenotype 1, Peptide Partial, 24 aa] [Homo sapiens]

Sequence ID: AAB25609.1Length: 24Number of Matches: 1

Range 1: 2 to 15GenPeptGraphics Next Match Previous Match

Alignment statistics for match #1

| Score | Expect | Identities | Positives | Gaps |
|---|---|---|---|---|
| 26.5 bits(55) | 1.1 | 11/15(73%) | 11/15(73%) | 2/15(13%) |

```
Query  1   CASS-TGTANYGYTF  14
           CASS  GT  YGYTF
Sbjct  2   CASSLRGTP-YGYTF  15
```

CASSVAGGRNEQFF (one 8/10 and one 7/10)

myelin basic protein-specific T-cell receptor beta chain VDJ region {clone JK1.16} [human, peripheral blood mononuclear cells, Peptide Partial, 27 aa] [Homo sapiens]

Sequence ID: AAB33031.1Length: 27Number of Matches: 1

Range 1: 1 to 18GenPeptGraphics Next Match Previous Match

Alignment statistics for match #1

| Score | Expect | Identities | Positives | Gaps |
|---|---|---|---|---|
| 27.4 bits(57) | 0.56 | 12/18(67%) | 12/18(66%) | 4/18(22%) |

```
Query  1   CASS---VAGGR-NEQFF  14
           CASS    AGG  NEQFF
Sbjct  1   CASSQEEGAGGPFNEQFF  18
```

myelin basic protein specific T-cell receptor V beta-D beta-J beta, MBP reactive TCR VDJ beta {clone SE(6), rearranged CDR3 region} [human, inflammatory brain lesions, HLA phenotype 1, Peptide Partial, 28 aa] [Homo sapiens]

Sequence ID: AAB25640.1Length: 28Number of Matches: 1

Range 1: 2 to 19GenPeptGraphics Next Match Previous Match

Alignment statistics for match #1

| Score | Expect | Identities | Positives | Gaps |
|---|---|---|---|---|
| 33.3 bits(71) | 0.004 | 11/18(61%) | 13/18(72%) | 4/18(22%) |

```
Query  1   CASSV----AGGRNEQFF  14
           CASS+    AG R+EQFF
Sbjct  2   CASSITCGLAGARDEQFF  19
```

CASSVGASTDTQYF (one 7/10)

myelin basic protein specific T-cell receptor V beta-D beta-J beta, MBP reactive TCR VDJ beta {clone KL-3(9), rearranged CDR3 region} [human, brain plaques, HLA phenotype 1, Peptide Partial, 25 aa] [Homo sapiens]

Sequence ID: AAB25612.1Length: 25Number of Matches: 1

Range 1: 2 to 16GenPeptGraphics Next Match Previous Match

Alignment statistics for match #1

| Score | Expect | Identities | Positives | Gaps |
|---|---|---|---|---|

29.5 bits(62) 0.085 11/15(73%) 11/15(73%) 1/15(6%)

```
Query  1    CASSVGAST-DTQYF  14
            CASS G S  DTQYF
Sbjct  2    CASSLGGSEEDTQYF  16
```

CASSYSGQGNEQYF (one 8/10 and one 7/10)

myelin basic protein (84-102)-specific TCR beta-chain {clone NB17.8} [human, multiple sclerosis patient, T cells, Peptide Partial, 27 aa] [Homo sapiens]

Sequence ID: AAB31433.1Length: 27Number of Matches: 1

Range 1: 1 to 14GenPeptGraphics Next Match Previous Match

Alignment statistics for match #1

| Score | Expect | Identities | Positives | Gaps |
|---|---|---|---|---|
| 34.6 bits(74) | 0.001 | 11/14(79%) | 12/14(85%) | 0/14(0%) |

```
Query  1    CASSYSGQGNEQYF  14
            CASSYS  GNEQ+F
Sbjct  1    CASSYSPLGNEQFF  14
```

myelin basic protein (143-168)-specific TCR beta-chain {clone HE.1D5} [human, multiple sclerosis patient, T cells, Peptide Partial, 29 aa] [Homo sapiens]

Sequence ID: AAB31428.1Length: 29Number of Matches: 1

Range 1: 1 to 16GenPeptGraphics Next Match Previous Match

Alignment statistics for match #1

| Score | Expect | Identities | Positives | Gaps |
|---|---|---|---|---|
| 26.5 bits(55) | 1.2 | 10/16(63%) | 11/16(68%) | 2/16(12%) |

```
Query  1    CASSYSGQG--NEQYF  14
            CASSYSG    + QYF
Sbjct  1    CASSYSGRVDTDTQYF  16
```

CASSPGTGGNEQYF (0)

None

CASSPNRGQETQYF (one 8/10)

myelin basic protein specific T-cell receptor V beta-D beta-J beta, MBP reactive TCR VDJ beta {CDR3 region, clone JO(4)} [human, normal peripheral blood lymphocytes, Peptide Partial, 25 aa] [Homo sapiens]

Sequence ID: AAB25659.1Length: 25Number of Matches: 1

Range 1: 2 to 16GenPeptGraphics Next Match Previous Match

Alignment statistics for match #1

| Score | Expect | Identities | Positives | Gaps |
|---|---|---|---|---|
| 33.3 bits(71) | 0.003 | 12/16(75%) | 12/16(75%) | 3/16(18%) |

```
Query  1    CASS--PNRGQETQYF  14
            CASS  P  GQETQYF
Sbjct  2    CASSDSPS-GQETQYF  16
```

CASSPRTANYGYTF (0)

None

CASSPRTDQETQYF (one 7/10)

myelin basic protein specific T-cell receptor V beta-D beta-J beta, MBP reactive TCR VDJ beta {CDR3 region, clone JO(4)} [human, normal peripheral blood lymphocytes, Peptide Partial, 25 aa] [Homo sapiens]

Sequence ID: AAB25659.1Length: 25Number of Matches: 1

Range 1: 2 to 16GenPeptGraphics Next Match Previous Match

Alignment statistics for match #1

| Score | Expect | Identities | Positives | Gaps |
|---|---|---|---|---|
| 29.5 bits(62) | 0.085 | 11/16(69%) | 11/16(68%) | 3/16(18%) |

```
Query  1    CASS--PRTDQETQYF  14
            CASS   P   QETQYF
Sbjct  2    CASSDSP-SGQETQYF  16
```

CASSPRTGGDEQYF (one 7/10)

myelin basic protein specific T-cell receptor V beta-D beta-J beta, MBP TCR reactive VDJ beta {CDR3 region, muscle infiltrating lymphocytes(3)} [human, muscle infiltrating lymphocytes, HLA phenotype 1, Peptide Partial, 27 aa] [Homo sapiens]

Sequence ID: AAB25651.1Length: 27Number of Matches: 1

Range 1: 2 to 18GenPeptGraphics Next Match Previous Match

Alignment statistics for match #1

| Score | Expect | Identities | Positives | Gaps |
|---|---|---|---|---|
| 28.6 bits(60) | 0.19 | 11/17(65%) | 11/17(64%) | 3/17(17%) |

```
Query  1    CASSPRTGG---DEQYF  14
            CASS RT G    D QYF
Sbjct  2    CASSRRTSGFVHDTQYF  18
```

**Probert, et al., 1996**

CASSEGQRTEAFFG (0)

None

CASSQGLMTEAFFG (one 8/10)

TCR V beta {V-J junction, clone MS9-2C5} [human, multiple sclerosis patient, myelin basic protein-reactive T cells after vaccination, Peptide Partial, 30 aa] [Homo sapiens]

Sequence ID: AAB35724.1Length: 30Number of Matches: 1

Range 1: 3 to 17GenPeptGraphics Next Match Previous Match

Alignment statistics for match #1

| Score | Expect | Identities | Positives | Gaps |
|---|---|---|---|---|
| 26.1 bits(54) | 1.8 | 11/15(73%) | 11/15(73%) | 1/15(6%) |

```
Query  1    CAS-SQGLMTEAFFG  14
```

```
             CAS   QG   TEAFFG
Sbjct  3     CASLGQGWNTEAFFG  17
```

CASSTREILTEAFFG (0)

None

CASSWIRGVGTEAFF (one 7/10)

myelin basic protein-specific T-cell receptor beta chain VDJ region {clone FN2.2} [human, peripheral blood mononuclear cells, Peptide Partial, 25 aa] [Homo sapiens]

Sequence ID: AAB33027.1Length: 25Number of Matches: 1

Range 1: 1 to 16GenPeptGraphics Next Match Previous Match

Alignment statistics for match #1

| Score | Expect | Identities | Positives | Gaps |
|---|---|---|---|---|
| 24.0 bits(49) | 11 | 11/17(65%) | 11/17(64%) | 3/17(17%) |

```
Query  1     CASSWIRGVGT--EAFF  15
             CASS   RG GT   E FF
Sbjct  1     CASS-LRGSGTYNEQFF  16
```

CASSGTGTSVYGYTFG   (one 8/10 and two 7/10)

myelin basic protein specific T-cell receptor V beta-D beta-J beta, MBP reactive TCR VDJ beta {clone KL-3(6), rearranged CDR3 region} [human, brain plaques, HLA phenotype 1, Peptide Partial, 24 aa] [Homo sapiens]

Sequence ID: AAB25609.1Length: 24Number of Matches: 1

Range 1: 2 to 16GenPeptGraphics Next Match Previous Match

Alignment statistics for match #1

| Score | Expect | Identities | Positives | Gaps |
|---|---|---|---|---|
| 29.1 bits(61) | 0.16 | 12/16(75%) | 12/16(75%) | 1/16(6%) |

```
Query  1     CASSGTGTSVYGYTFG  16
             CASS   GT   YGYTFG
Sbjct  2     CASSLRGTP-YGYTFG  16
```

myelin basic protein specific T-cell receptor V beta-D beta-J beta, MBP reactive TCR VDJ beta {clone KL-3(1), rearranged CDR3 region} [human, brain plaques, HLA phenotype 1, Peptide Partial, 24 aa] [Homo sapiens]

Sequence ID: AAB25604.1Length: 24Number of Matches: 1

Range 1: 2 to 16GenPeptGraphics Next Match Previous Match

Alignment statistics for match #1

| Score | Expect | Identities | Positives | Gaps |
|---|---|---|---|---|
| 26.5 bits(55) | 1.4 | 12/19(63%) | 12/19(63%) | 7/19(36%) |

```
Query  1     CASS--GTGTSV-YGYTFG  16
             CASS  G     V YGYTFG
Sbjct  2     CASSLDG----VPYGYTFG  16
```

myelin basic protein specific T-cell receptor V beta-D beta-J beta, MBP reactive TCR VDJ beta {clone SE(2), rearranged CDR3 region} [human, inflammatory brain lesions, HLA phenotype 1, Peptide Partial, 24 aa] [Homo sapiens]

Sequence ID: AAB25636.1Length: 24Number of Matches: 1

Range 1: 2 to 16GenPeptGraphics Next Match Previous Match

Alignment statistics for match #1

| Score | Expect | Identities | Positives | Gaps |
| --- | --- | --- | --- | --- |
| 26.5 bits(55) | 1.4 | 12/17(71%) | 12/17(70%) | 3/17(17%) |

```
Query  1    CASSG-TGTSVYGYTFG  16
            CASSG  GT   YG TFG
Sbjct  2    CASSGRSGTN-YG-TFG  16
```

CASSDLEGGNEKLFFG (one 7/10)

myelin basic protein specific T-cell receptor V beta-D beta-J beta, MBP TCR reactive VDJ beta {CDR3 region, muscle infiltrating lymphocytes(1)} [human, muscle infiltrating lymphocytes, HLA phenotype 1, Peptide Partial, 28 aa] [Homo sapiens]

Sequence ID: AAB25649.1Length: 28Number of Matches: 1

Range 1: 2 to 20GenPeptGraphics Next Match Previous Match

Alignment statistics for match #1

| Score | Expect | Identities | Positives | Gaps |
| --- | --- | --- | --- | --- |
| 28.2 bits(59) | 0.38 | 13/22(59%) | 13/22(59%) | 9/22(40%) |

```
Query  1    CASSDLEGG------NEKLFFG  16
            CASS L  G      NEKLFFG
Sbjct  2    CASS-L--GSPGYRTNEKLFFG  20
```

CASSGTAAPQPQHFG (0)

None

CASSGNRVPQPQHFG (two 7/10)

myelin basic protein (84-102)-specific TCR beta-chain {clone NSJL14.5} [human, multiple sclerosis patient, T cells, Peptide Partial, 25 aa] [Homo sapiens]

Sequence ID: AAB31432.1Length: 25Number of Matches: 1

Range 1: 1 to 13GenPeptGraphics Next Match Previous Match

Alignment statistics for match #1

| Score | Expect | Identities | Positives | Gaps |
| --- | --- | --- | --- | --- |
| 30.3 bits(64) | 0.047 | 11/16(69%) | 12/16(75%) | 4/16(25%) |

```
Query  1    CASSGNRVP-QPQHFG  15
            CASS    +P QPQHFG
Sbjct  1    CASS---IPGQPQHFG  13
```

myelin basic protein specific T-cell receptor V beta-D beta-J beta, MBP reactive TCR VDJ beta {CDR3 region, clone JO(2)} [human, normal peripheral blood lymphocytes, Peptide Partial, 24 aa] [Homo sapiens]

Sequence ID: AAB25657.1Length: 24Number of Matches: 1
Range 1: 2 to 16GenPeptGraphics Next Match Previous Match

Alignment statistics for match #1

| Score | Expect | Identities | Positives | Gaps |
|---|---|---|---|---|
| 29.9 bits(63) | 0.065 | 11/15(73%) | 11/15(73%) | 0/15(0%) |

```
Query  1    CASSGNRVPQPQHFG  15
            CASS   R  QPQHFG
Sbjct  2    CASSTVRGSQPQHFG  16
```

CASSSGGSSQGQPQHFG (one 7/10)
myelin basic protein (84-102)-specific TCR beta-chain {clone NSJL14.5} [human, multiple sclerosis patient, T cells, Peptide Partial, 25 aa] [Homo sapiens]
Sequence ID: AAB31432.1Length: 25Number of Matches: 1
Range 1: 1 to 13GenPeptGraphics Next Match Previous Match

Alignment statistics for match #1

| Score | Expect | Identities | Positives | Gaps |
|---|---|---|---|---|
| 28.2 bits(59) | 0.39 | 11/17(65%) | 11/17(64%) | 4/17(23%) |

```
Query  1    CASSSGGSSQGQPQHFG  17
            CASS      GQPQHFG
Sbjct  1    CASSI----PGQPQHFG  13
```

CASSSGGGDQPQHFG (two 7/10)
myelin basic protein specific T-cell receptor V beta-D beta-J beta, MBP reactive TCR VDJ beta {CDR3 region, clone JO(2)} [human, normal peripheral blood lymphocytes, Peptide Partial, 24 aa] [Homo sapiens]
Sequence ID: AAB25657.1Length: 24Number of Matches: 1
Range 1: 2 to 16GenPeptGraphics Next Match Previous Match

Alignment statistics for match #1

| Score | Expect | Identities | Positives | Gaps |
|---|---|---|---|---|
| 30.3 bits(64) | 0.045 | 11/15(73%) | 11/15(73%) | 0/15(0%) |

```
Query  1    CASSSGGGDQPQHFG  15
            CASS   G QPQHFG
Sbjct  2    CASSTVRGSQPQHFG  16
```

myelin basic protein (84-102)-specific TCR beta-chain {clone NSJL14.5} [human, multiple sclerosis patient, T cells, Peptide Partial, 25 aa] [Homo sapiens]
Sequence ID: AAB31432.1Length: 25Number of Matches: 1
Range 1: 1 to 13GenPeptGraphics Next Match Previous Match

Alignment statistics for match #1

| Score | Expect | Identities | Positives | Gaps |
|---|---|---|---|---|
| 27.8 bits(58) | 0.43 | 11/15(73%) | 11/15(73%) | 2/15(13%) |

```
Query  1    CASSSGGGDQPQHFG  15
```

```
            CASS  G  QPQHFG
Sbjct  1    CASSIPG--QPQHFG  13
```

CASSADRISPLHFG (two 7/10)

myelin basic protein (143-168)-specific TCR beta-chain {clone JA.1.7} [human, multiple sclerosis patient, T cells, Peptide Partial, 28 aa] [Homo sapiens]

Sequence ID: AAB31430.1Length: 28Number of Matches: 1

Range 1: 1 to 16GenPeptGraphics Next Match Previous Match

Alignment statistics for match #1

| Score | Expect | Identities | Positives | Gaps |
|---|---|---|---|---|
| 25.2 bits(52) | 3.5 | 11/16(69%) | 11/16(68%) | 2/16(12%) |

```
Query  1    CASS--ADRISPLHFG  14
            CASS    R SPLHFG
Sbjct  1    CASSPMTGRWSPLHFG  16
```

myelin basic protein specific T-cell receptor V beta-D beta-J beta, MBP reactive TCR VDJ beta {clone KL-1(9), rearranged CDR3 region} [human, brain plaques, HLA phenotype 1, Peptide Partial, 24 aa] [Homo sapiens]

Sequence ID: AAB25600.1Length: 24Number of Matches: 1

Range 1: 2 to 16GenPeptGraphics Next Match Previous Match

Alignment statistics for match #1

| Score | Expect | Identities | Positives | Gaps |
|---|---|---|---|---|
| 21.0 bits(42) | 114 | 9/15(60%) | 9/15(60%) | 1/15(6%) |

```
Query  1    CASSAD-RISPLHFG  14
            CASS D R S    FG
Sbjct  2    CASSSDGRMSTQYFG  16
```

CASSGESQFFG (0)

None

CASSSSGGYIQFFG (one 7/10)

myelin basic protein-specific T-cell receptor beta chain VDJ region {clone FN2.2} [human, peripheral blood mononuclear cells, Peptide Partial, 25 aa] [Homo sapiens]

Sequence ID: AAB33027.1Length: 25Number of Matches: 1

Range 1: 1 to 17GenPeptGraphics Next Match Previous Match

Alignment statistics for match #1

| Score | Expect | Identities | Positives | Gaps |
|---|---|---|---|---|
| 23.5 bits(48) | 14 | 11/17(65%) | 11/17(64%) | 3/17(17%) |

```
Query  1    CASS--SSGGYI-QFFG  14
            CASS   SG Y  QFFG
Sbjct  1    CASSLRGSGTYNEQFFG  17
```

CASSTGQGGDQFFG (one 8/10 and two 7/10)

myelin basic protein specific T-cell receptor V beta-D beta-J beta, MBP reactive TCR VDJ beta {clone SE(8), rearranged CDR3 region} [human, inflammatory brain lesions, HLA phenotype 1, Peptide Partial, 24 aa] [Homo sapiens]
Sequence ID: AAB25642.1Length: 24Number of Matches: 1
Range 1: 2 to 16GenPeptGraphics Next Match Previous Match

Alignment statistics for match #1

| Score | Expect | Identities | Positives | Gaps |
|---|---|---|---|---|
| 28.6 bits(60) | 0.17 | 11/15(73%) | 12/15(80%) | 1/15(6%) |

```
Query  1    CASST-GQGGDQFFG  14
            CASST GQ G+ FFG
Sbjct  2    CASSTSGQPGELFFG  16
```

myelin basic protein specific T-cell receptor V beta-D beta-J beta, MBP reactive TCR VDJ beta {clone SE(5), rearranged CDR3 region} [human, inflammatory brain lesions, HLA phenotype 1, Peptide Partial, 26 aa] [Homo sapiens]
Sequence ID: AAB25639.1Length: 26Number of Matches: 1
Range 1: 2 to 18GenPeptGraphics Next Match Previous Match

Alignment statistics for match #1

| Score | Expect | Identities | Positives | Gaps |
|---|---|---|---|---|
| 28.6 bits(60) | 0.18 | 11/17(65%) | 12/17(70%) | 3/17(17%) |

```
Query  1    CASST---GQGGDQFFG  14
            CASS    GQG +QFFG
Sbjct  2    CASSRLDRGQGEEQFFG  18
```

TCR V beta {V-J junction, clone MS7-4G8} [human, multiple sclerosis patient, myelin basic protein-reactive T cells before vaccination, Peptide Partial, 30 aa] [Homo sapiens]
Sequence ID: AAB35719.1Length: 30Number of Matches: 1
Range 1: 3 to 17GenPeptGraphics Next Match Previous Match

Alignment statistics for match #1

| Score | Expect | Identities | Positives | Gaps |
|---|---|---|---|---|
| 24.4 bits(50) | 7.3 | 9/15(60%) | 11/15(73%) | 1/15(6%) |

```
Query  1    CASSTGQGGDQ-FFG  14
            CASS GQG +  +FG
Sbjct  3    CASSSGQGSNTIYFG  17
```

CASSDRDRTTDTQYFG (one 7/10)

myelin basic protein specific T-cell receptor V beta-D beta-J beta, MBP TCR reactive VDJ beta {CDR3 region, muscle infiltrating lymphocytes(5)} [human, muscle infiltrating lymphocytes, HLA phenotype 1, Peptide Partial, 28 aa] [Homo sapiens]
Sequence ID: AAB25653.1Length: 28Number of Matches: 1
Range 1: 2 to 20GenPeptGraphics Next Match Previous Match

Alignment statistics for match #1

| Score | Expect | Identities | Positives | Gaps |
|---|---|---|---|---|
| 34.6 bits(74) | 0.002 | 13/20(65%) | 13/20(65%) | 5/20(25%) |

```
Query  1    CASSDRDRT----TDTQYFG  16
            CAS   R RT    TDTQYFG
Sbjct  2    CASTAR-RTSGFVTDTQYFG  20
```

CASSGMAGGTDTQYFG (two 7/10)

myelin basic protein specific T-cell receptor V beta-D beta-J beta, MBP reactive TCR VDJ beta {clone KL-3(13), rearranged CDR3 region} [human, brain plaques, HLA phenotype 1, Peptide Partial, 26 aa] [Homo sapiens]

Sequence ID: AAB25616.1Length: 26Number of Matches: 1

Range 1: 2 to 18GenPeptGraphics Next Match Previous Match

Alignment statistics for match #1

| Score | Expect | Identities | Positives | Gaps |
|---|---|---|---|---|
| 31.6 bits(67) | 0.019 | 12/18(67%) | 14/18(77%) | 3/18(16%) |

```
Query  1    CASSGMAGGT--DTQYFG  16
            CASS +A GT  +TQYFG
Sbjct  2    CASS-LASGTLQETQYFG  18
```

myelin basic protein (143-168)-specific TCR beta-chain {clone JA.1.7} [human, multiple sclerosis patient, T cells, Peptide Partial, 27 aa] [Homo sapiens]

Sequence ID: AAB31429.1Length: 27Number of Matches: 1

Range 1: 1 to 15GenPeptGraphics Next Match Previous Match

Alignment statistics for match #1

| Score | Expect | Identities | Positives | Gaps |
|---|---|---|---|---|
| 30.3 bits(64) | 0.060 | 11/16(69%) | 13/16(81%) | 1/16(6%) |

```
Query  1    CASSGMAGGTDTQYFG  16
            CASS + GG +TQYFG
Sbjct  1    CASS-LDGGEETQYFG  15
```

myelin basic protein (143-168)-specific TCR beta-chain {clone HE.1D5} [human, multiple sclerosis patient, T cells, Peptide Partial, 29 aa] [Homo sapiens]

Sequence ID: AAB31428.1Length: 29Number of Matches: 1

Range 1: 1 to 17GenPeptGraphics Next Match Previous Match

Alignment statistics for match #1

| Score | Expect | Identities | Positives | Gaps |
|---|---|---|---|---|
| 28.2 bits(59) | 0.39 | 12/18(67%) | 12/18(66%) | 3/18(16%) |

```
Query  1    CASS--GMAGGTDTQYFG  16
            CASS  G    TDTQYFG
Sbjct  1    CASSYSGRVD-TDTQYFG  17
```

CASSALKRKDYEIGYFG (0)

None

CASSLGLAGVSYEQYFG (four 8/10 and two 7/10)

myelin basic protein specific T-cell receptor V beta-D beta-J beta, MBP reactive TCR VDJ beta {clone LJ 1(6), rearranged CDR3 region} [human, brain plaques, HLA phenotype 1, Peptide Partial, 24 aa] [Homo sapiens]

Sequence ID: AAB25632.1Length: 24Number of Matches: 1

Range 1: 2 to 16GenPeptGraphics Next Match Previous Match

Alignment statistics for match #1

| Score | Expect | Identities | Positives | Gaps |
|---|---|---|---|---|
| 36.3 bits(78) | 3e-04 | 13/17(76%) | 14/17(82%) | 2/17(11%) |

```
Query  1   CASSLGLAGVSYEQYFG  17
           CASSL  AG+ YEQYFG
Sbjct  2   CASSL-VAGI-YEQYFG  16
```

myelin basic protein-specific T-cell receptor beta chain VDJ region {clone HOO24} [human, peripheral blood mononuclear cells, Peptide Partial, 25 aa] [Homo sapiens]

Sequence ID: AAB33029.1Length: 25Number of Matches: 1

Range 1: 1 to 17GenPeptGraphics Next Match Previous Match

Alignment statistics for match #1

| Score | Expect | Identities | Positives | Gaps |
|---|---|---|---|---|
| 37.1 bits(80) | 2e-04 | 14/18(78%) | 14/18(77%) | 2/18(11%) |

```
Query  1   CASSLGLA-GVSYEQYFG  17
           CASS  LA G SYEQYFG
Sbjct  1   CASS-SLARGASYEQYFG  1
```

myelin basic protein specific T-cell receptor V beta-D beta-J beta, MBP reactive TCR VDJ beta {clone KL-3(8), rearranged CDR3 region} [human, brain plaques, HLA phenotype 1, Peptide Partial, 25 aa] [Homo sapiens]

Sequence ID: AAB25611.1Length: 25Number of Matches: 1

Range 1: 2 to 17GenPeptGraphics Next Match Previous Match

Alignment statistics for match #1

| Score | Expect | Identities | Positives | Gaps |
|---|---|---|---|---|
| 33.7 bits(72) | 0.003 | 13/18(72%) | 14/18(77%) | 3/18(16%) |

```
Query  1   CASSLGLAGVSY-EQYFG  17
           CASSL LAG   Y EQ+FG
Sbjct  2   CASSLELAG--YNEQFFG  17
```

myelin basic protein specific T-cell receptor V beta-D beta-J beta, MBP reactive TCR VDJ beta {clone SE(7), rearranged CDR3 region} [human, inflammatory brain lesions, HLA phenotype 1, Peptide Partial, 26 aa] [Homo sapiens]
Sequence ID: AAB25641.1Length: 26Number of Matches: 1
Range 1: 2 to 18GenPeptGraphics Next Match Previous Match

Alignment statistics for match #1

| Score | Expect | Identities | Positives | Gaps |
|---|---|---|---|---|
| 30.3 bits(64) | 0.066 | 12/17(71%) | 13/17(76%) | 0/17(0%) |

```
Query  1    CASSLGLAGVSYEQYFG  17
            CASS GLAG    EQ+FG
Sbjct  2    CASSWGLAGARDEQFFG  18
```

myelin basic protein specific T-cell receptor V beta-D beta-J beta, MBP reactive TCR VDJ beta {CDR3 region, clone BM(5)} [human, peripheral blood lymphocytes, HLA phenotype 1, Peptide Partial, 23 aa] [Homo sapiens]
Sequence ID: AAB25666.1Length: 23Number of Matches: 1
Range 1: 2 to 15GenPeptGraphics Next Match Previous Match

Alignment statistics for match #1

| Score | Expect | Identities | Positives | Gaps |
|---|---|---|---|---|
| 29.9 bits(63) | 0.082 | 11/17(65%) | 11/17(64%) | 3/17(17%) |

```
Query  1    CASSLGLAGVSYEQYFG  17
            CASS      SYEQYFG
Sbjct  2    CASS---QEASYEQYFG  15
```

Download GenPeptGraphics Next Previous Descriptions
myelin basic protein specific T-cell receptor V beta-D beta-J beta, MBP reactive TCR VDJ beta {clone SE(14), rearranged CDR3 region} [human, inflammatory brain lesions, HLA phenotype 1, Peptide Partial, 23 aa] [Homo sapiens]
Sequence ID: AAB25648.1Length: 23Number of Matches: 1
Range 1: 2 to 15GenPeptGraphics Next Match Previous Match

Alignment statistics for match #1

| Score | Expect | Identities | Positives | Gaps |
|---|---|---|---|---|
| 29.9 bits(63) | 0.082 | 12/17(71%) | 12/17(70%) | 3/17(17%) |

```
Query  1    CASSLGLAGVSYEQYFG  17
            CASS   AG  YEQYFG
Sbjct  2    CASST--AG-TYEQYFG  15
```

(18 sequences)

CSAEGRGTEAFFG (one 7/10)

TCR V beta {V-J junction, clone MS9-2C5} [human, multiple sclerosis patient, myelin basic protein-reactive T cells after vaccination, Peptide Partial, 30 aa] [Homo sapiens]

Sequence ID: AAB35724.1Length: 30Number of Matches: 1

Range 1: 8 to 17GenPeptGraphics Next Match Previous Match

Alignment statistics for match #1

| Score | Expect | Identities | Positives | Gaps |
|---|---|---|---|---|
| 23.1 bits(47) | 18 | 7/10(70%) | 8/10(80%) | 0/10(0%) |

```
Query  4    EGRGTEAFFG  13
            +G  TEAFFG
Sbjct  8    QGWNTEAFFG  17
```

CSASPWTQTEAFFG (one 7/10)

TCR V beta {V-J junction, clone MS9-2C5} [human, multiple sclerosis patient, myelin basic protein-reactive T cells after vaccination, Peptide Partial, 30 aa] [Homo sapiens]

Sequence ID: AAB35724.1Length: 30Number of Matches: 1

Range 1: 10 to 17GenPeptGraphics Next Match Previous Match

Alignment statistics for match #1

| Score | Expect | Identities | Positives | Gaps |
|---|---|---|---|---|
| 22.7 bits(46) | 30 | 7/9(78%) | 7/9(77%) | 1/9(11%) |

```
Query  6    WTQTEAFFG  14
            W  TEAFFG
Sbjct  10   WN-TEAFFG  17
```

CASRPGFQPQHFG (two 8/10)

myelin basic protein (84-102)-specific TCR beta-chain {clone NSJL14.5} [human, multiple sclerosis patient, T cells, Peptide Partial, 25 aa] [Homo sapiens]

Sequence ID: AAB31432.1Length: 25Number of Matches: 1

Range 1: 1 to 13GenPeptGraphics Next Match Previous Match

Alignment statistics for match #1

| Score | Expect | Identities | Positives | Gaps |
|---|---|---|---|---|
| 29.1 bits(61) | 0.10 | 11/14(79%) | 11/14(78%) | 2/14(14%) |

```
Query  1    CASR-PGFQPQHFG  13
            CAS  PG QPQHFG
Sbjct  1    CASSIPG-QPQHFG  13
```

myelin basic protein specific T-cell receptor V beta-D beta-J beta, MBP reactive TCR VDJ beta {CDR3 region, clone JO(2)} [human, normal peripheral blood lymphocytes, Peptide Partial, 24 aa] [Homo sapiens]

Sequence ID: AAB25657.1Length: 24Number of Matches: 1

Range 1: 2 to 16GenPeptGraphics Next Match Previous Match

Alignment statistics for match #1

| Score | Expect | Identities | Positives | Gaps |
|---|---|---|---|---|
| 26.9 bits(56) | 0.63 | 11/16(69%) | 11/16(68%) | 4/16(25%) |

```
Query  1    CAS---RPGFQPQHFG  13
            CAS   R G QPQHFG
Sbjct  2    CASSTVR-GSQPQHFG  16
```

CSARGSAQPQHFG (one 9/10 and one 8/10)

myelin basic protein specific T-cell receptor V beta-D beta-J beta, MBP reactive TCR VDJ beta {CDR3 region, clone JO(2)} [human, normal peripheral blood lymphocytes, Peptide Partial, 24 aa] [Homo sapiens]

Sequence ID: AAB25657.1Length: 24Number of Matches: 1

Range 1: 8 to 16GenPeptGraphics Next Match Previous Match

Alignment statistics for match #1

| Score | Expect | Identities | Positives | Gaps |
|---|---|---|---|---|
| 27.8 bits(58) | 0.30 | 9/10(90%) | 9/10(90%) | 1/10(10%) |

```
Query  4    RGSAQPQHFG  13
            RGS QPQHFG
Sbjct  8    RGS-QPQHFG  16
```

Download GenPeptGraphics Next Previous Descriptions

X2 MBP-specific T cell Receptor beta chain VJ region {DR2/DQw1 varient, clone 1G1} [human, healthy sibling NL-B3, Peptide Partial, 15 aa] [Homo sapiens]

Sequence ID: AAB32433.1Length: 15Number of Matches: 1

Range 1: 7 to 15GenPeptGraphics Next Match Previous Match

Alignment statistics for match #1

| Score | Expect | Identities | Positives | Gaps |
|---|---|---|---|---|
| 26.9 bits(56) | 0.38 | 8/9(89%) | 8/9(88%) | 0/9(0%) |

```
Query  5    GSAQPQHFG  13
            GS QPQHFG
Sbjct  7    GSNQPQHFG  15
```

CSAIGQTVEQPQHFG (0)

None

CSASRWQGGNQPQHFG (two 7/10)

myelin basic protein (84-102)-specific TCR beta-chain {clone Wk.2G3.8} [human, multiple sclerosis patient, T cells, Peptide Partial, 32 aa] [Homo sapiens]

Sequence ID: AAB31434.1Length: 32Number of Matches: 1

Range 1: 4 to 20GenPeptGraphics Next Match Previous Match

Alignment statistics for match #1

| Score | Expect | Identities | Positives | Gaps |
|---|---|---|---|---|
| 33.3 bits(71) | 0.006 | 12/17(71%) | 12/17(70%) | 2/17(11%) |

```
Query  2    SASR-WQG-GNQPQHFG  16
```

```
           S SR WQ   NQPQHFG
Sbjct  4   STSRDWQDYSNQPQHFG  20
```

TCR V beta {V-J junction, clone MS7-1B10} [human, multiple sclerosis patient, myelin basic protein-reactive T cells after vaccination, Peptide Partial, 29 aa] [Homo sapiens]
Sequence ID: AAB35720.1Length: 29Number of Matches: 1
Range 1: 10 to 16GenPeptGraphics Next Match Previous Match

Alignment statistics for match #1

| Score | Expect | Identities | Positives | Gaps |
|---|---|---|---|---|
| 26.9 bits(56) | 1.1 | 7/7(100%) | 7/7(100%) | 0/7(0%) |

```
Query  10  NQPQHFG  16
           NQPQHFG
Sbjct  10  NQPQHFG  16
```

CSARRPGTGWVQGQPQHFG (one 8/10 and one 7/10)
myelin basic protein specific T-cell receptor V beta-D beta-J beta, MBP reactive TCR VDJ beta {CDR3 region, clone JO(2)} [human, normal peripheral blood lymphocytes, Peptide Partial, 24 aa] [Homo sapiens]
Sequence ID: AAB25657.1Length: 24Number of Matches: 1
Range 1: 7 to 16GenPeptGraphics Next Match Previous Match

Alignment statistics for match #1

| Score | Expect | Identities | Positives | Gaps |
|---|---|---|---|---|
| 24.0 bits(49) | 17 | 8/10(80%) | 8/10(80%) | 1/10(10%) |

```
Query  11  VQG-QPQHFG  19
           V G QPQHFG
Sbjct  7   VRGSQPQHFG  16
```

myelin basic protein (84-102)-specific TCR beta-chain {clone NSJL14.5} [human, multiple sclerosis patient, T cells, Peptide Partial, 25 aa] [Homo sapiens]
Sequence ID: AAB31432.1Length: 25Number of Matches: 1
Range 1: 7 to 13GenPeptGraphics Next Match Previous Match

Alignment statistics for match #1

| Score | Expect | Identities | Positives | Gaps |
|---|---|---|---|---|
| 26.1 bits(54) | 3.0 | 7/7(100%) | 7/7(100%) | 0/7(0%) |

```
Query  13  GQPQHFG  19
           GQPQHFG
Sbjct  7   GQPQHFG  13
```

CSATSGQFFG (0)
None
CSAEGGTDQFFG (0)
None
CSAYSSGASQFFG (0)

None

CSARDGRDGQFFG (0)

None

CSASTGSGSQFFG (0)

None

CSARGLETGELFFG (one 7/10)

myelin basic protein-specific T-cell receptor beta chain VDJ region {clone NN2.23} [human, peripheral blood mononuclear cells, Peptide Partial, 25 aa] [Homo sapiens]

Sequence ID: AAB33028.1Length: 25Number of Matches: 1

Range 1: 11 to 17GenPeptGraphics Next Match Previous Match

Alignment statistics for match #1

| Score | Expect | Identities | Positives | Gaps |
|---|---|---|---|---|
| 25.2 bits(52) | 3.2 | 7/7(100%) | 7/7(100%) | 0/7(0%) |

```
Query  8   TGELFFG  14
           TGELFFG
Sbjct  11  TGELFFG  17
```

CSAGAATDTQYFG (two 7/10)

myelin basic protein-specific T-cell receptor beta chain VDJ region {clone HUL16} [human, peripheral blood mononuclear cells, Peptide Partial, 24 aa] [Homo sapiens]

Sequence ID: AAB33030.1Length: 24Number of Matches: 1

Range 1: 1 to 16GenPeptGraphics Next Match Previous Match

Alignment statistics for match #1

| Score | Expect | Identities | Positives | Gaps |
|---|---|---|---|---|
| 32.0 bits(68) | 0.007 | 11/16(69%) | 11/16(68%) | 3/16(18%) |

```
Query  1   CSAGA---ATDTQYFG  13
           CSAG    A DTQYFG
Sbjct  1   CSAGPGQVASDTQYFG  16
```

myelin basic protein specific T-cell receptor V beta-D beta-J beta, MBP TCR reactive VDJ beta {CDR3 region, muscle infiltrating lymphocytes(5)} [human, muscle infiltrating lymphocytes, HLA phenotype 1, Peptide Partial, 28 aa] [Homo sapiens]

Sequence ID: AAB25653.1Length: 28Number of Matches: 1

Range 1: 14 to 20GenPeptGraphics Next Match Previous Match

Alignment statistics for match #1

| Score | Expect | Identities | Positives | Gaps |
|---|---|---|---|---|
| 26.5 bits(55) | 1.0 | 7/7(100%) | 7/7(100%) | 0/7(0%) |

```
Query  7   TDTQYFG  13
           TDTQYFG
Sbjct  14  TDTQYFG  20
```

CSASLANTDTQYFG (one 7/10)

myelin basic protein (143-168)-specific TCR beta-chain {clone HE.1D5} [human, multiple sclerosis patient, T cells, Peptide Partial, 29 aa] [Homo sapiens]
Sequence ID: AAB31428.1Length: 29Number of Matches: 1
Range 1: 10 to 17GenPeptGraphics Next Match Previous Match

Alignment statistics for match #1

| Score | Expect | Identities | Positives | Gaps |
|---|---|---|---|---|
| 27.4 bits(57) | 0.59 | 7/8(88%) | 8/8(100%) | 0/8(0%) |

```
Query  7   NTDTQYFG  14
           +TDTQYFG
Sbjct  10  DTDTQYFG  17
```

CSAEVAGGTDTQYFG (one 7/10)
myelin basic protein specific T-cell receptor V beta-D beta-J beta, MBP TCR reactive VDJ beta {CDR3 region, muscle infiltrating lymphocytes(5)} [human, muscle infiltrating lymphocytes, HLA phenotype 1, Peptide Partial, 28 aa] [Homo sapiens]
Sequence ID: AAB25653.1Length: 28Number of Matches: 1
Range 1: 14 to 20GenPeptGraphics Next Match Previous Match

Alignment statistics for match #1

| Score | Expect | Identities | Positives | Gaps |
|---|---|---|---|---|
| 26.5 bits(55) | 1.4 | 7/7(100%) | 7/7(100%) | 0/7(0%) |

```
Query  9   TDTQYFG  15
           TDTQYFG
Sbjct  14  TDTQYFG  20
```

CSAVLLLATTDTQYFG (two 7/10)
myelin basic protein specific T-cell receptor V beta-D beta-J beta, MBP TCR reactive VDJ beta {CDR3 region, muscle infiltrating lymphocytes(5)} [human, muscle infiltrating lymphocytes, HLA phenotype 1, Peptide Partial, 28 aa] [Homo sapiens]
Sequence ID: AAB25653.1Length: 28Number of Matches: 1
Range 1: 14 to 20GenPeptGraphics Next Match Previous Match

Alignment statistics for match #1

| Score | Expect | Identities | Positives | Gaps |
|---|---|---|---|---|
| 26.5 bits(55) | 1.6 | 7/7(100%) | 7/7(100%) | 0/7(0%) |

```
Query  10  TDTQYFG  16
           TDTQYFG
Sbjct  14  TDTQYFG  20
```

myelin basic protein specific T-cell receptor V beta-D beta-J beta, MBP reactive TCR VDJ beta {clone SE(9), rearranged CDR3 region} [human, inflammatory brain lesions, HLA phenotype 1, Peptide Partial, 25 aa] [Homo sapiens]
Sequence ID: AAB25643.1Length: 25Number of Matches: 1
Range 1: 8 to 17GenPeptGraphics Next Match Previous Match

---

Alignment statistics for match #1

| Score | Expect | Identities | Positives | Gaps |
|---|---|---|---|---|
| 24.0 bits(49) | 13 | 7/10(70%) | 7/10(70%) | 0/10(0%) |

```
Query  7   LATTDTQYFG  16
           L   DTQYFG
Sbjct  8   LGKADTQYFG  17
```

CSARWAGGPTDTQYFG (one 7/10)

myelin basic protein specific T-cell receptor V beta-D beta-J beta, MBP TCR reactive VDJ beta {CDR3 region, muscle infiltrating lymphocytes(5)} [human, muscle infiltrating lymphocytes, HLA phenotype 1, Peptide Partial, 28 aa] [Homo sapiens]

Sequence ID: AAB25653.1Length: 28Number of Matches: 1

Range 1: 14 to 20GenPeptGraphics Next Match Previous Match

Alignment statistics for match #1

| Score | Expect | Identities | Positives | Gaps |
|---|---|---|---|---|
| 26.5 bits(55) | 1.6 | 7/7(100%) | 7/7(100%) | 0/7(0%) |

```
Query  10  TDTQYFG  16
           TDTQYFG
Sbjct  14  TDTQYFG  20
```

CSATRGRETQYFG (two 7/10)

myelin basic protein specific T-cell receptor V beta-D beta-J beta, MBP reactive TCR VDJ beta {CDR3 region, clone JO(5)} [human, normal peripheral blood lymphocytes, Peptide Partial, 25 aa] [Homo sapiens]

Sequence ID: AAB25660.1Length: 25Number of Matches: 1

Range 1: 2 to 17GenPeptGraphics Next Match Previous Match

Alignment statistics for match #1

| Score | Expect | Identities | Positives | Gaps |
|---|---|---|---|---|
| 28.2 bits(59) | 0.22 | 10/16(63%) | 10/16(62%) | 3/16(18%) |

```
Query  1   CSATR-G--RETQYFG  13
           C   R G  RETQYFG
Sbjct  2   CASSRPGNIRETQYFG  17
```

myelin basic protein specific T-cell receptor V beta-D beta-J beta, MBP reactive TCR VDJ beta {CDR3 region, clone JO(4)} [human, normal peripheral blood lymphocytes, Peptide Partial, 25 aa] [Homo sapiens]

Sequence ID: AAB25659.1Length: 25Number of Matches: 1

Range 1: 10 to 17GenPeptGraphics Next Match Previous Match

Alignment statistics for match #1

| Score | Expect | Identities | Positives | Gaps |
|---|---|---|---|---|
| 25.2 bits(52) | 2.8 | 7/8(88%) | 7/8(87%) | 0/8(0%) |

```
Query  6   GRETQYFG  13
           G ETQYFG
Sbjct  10  GQETQYFG  17
```

CSASGGDTSYEQYFG (two 7/10)

myelin basic protein specific T-cell receptor V beta-D beta-J beta, MBP reactive TCR VDJ beta {CDR3 region, clone BM(5)} [human, peripheral blood lymphocytes, HLA phenotype 1, Peptide Partial, 23 aa] [Homo sapiens]

Sequence ID: AAB25666.1Length: 23Number of Matches: 1

Range 1: 3 to 15GenPeptGraphics Next Match Previous Match

Alignment statistics for match #1

| Score | Expect | Identities | Positives | Gaps |
|---|---|---|---|---|
| 29.1 bits(61) | 0.13 | 9/13(69%) | 10/13(76%) | 0/13(0%) |

```
Query  3    ASGGDTSYEQYFG  15
            AS   + SYEQYFG
Sbjct  3    ASSQEASYEQYFG  15
```

T-cell receptor beta chain variable region {junctional region} [human, monozygous twin, myelin basic protein-specific T cell line, Peptide Partial, 22 aa] [Homo sapiens]

Sequence ID: AAB37680.1Length: 22Number of Matches: 1

Range 1: 7 to 13GenPeptGraphics Next Match Previous Match

Alignment statistics for match #1

| Score | Expect | Identities | Positives | Gaps |
|---|---|---|---|---|
| 27.4 bits(57) | 0.54 | 7/7(100%) | 7/7(100%) | 0/7(0%) |

```
Query  9    SYEQYFG  15
            SYEQYFG
Sbjct  7    SYEQYFG  13
```

CSARDQGLADSYEQYFG (two 7/10)

myelin basic protein specific T-cell receptor V beta-D beta-J beta, MBP reactive TCR VDJ beta {CDR3 region, clone BM(5)} [human, peripheral blood lymphocytes, HLA phenotype 1, Peptide Partial, 23 aa] [Homo sapiens]

Sequence ID: AAB25666.1Length: 23Number of Matches: 1

Range 1: 9 to 15GenPeptGraphics Next Match Previous Match

Alignment statistics for match #1

| Score | Expect | Identities | Positives | Gaps |
|---|---|---|---|---|
| 27.4 bits(57) | 0.74 | 7/7(100%) | 7/7(100%) | 0/7(0%) |

```
Query  11   SYEQYFG  17
            SYEQYFG
Sbjct  9    SYEQYFG  15
```

myelin basic protein specific T-cell receptor V beta-D beta-J beta, MBP reactive TCR VDJ beta {clone SE(14), rearranged CDR3 region} [human, inflammatory brain lesions, HLA phenotype 1, Peptide Partial, 23 aa] [Homo sapiens]

Sequence ID: AAB25648.1Length: 23Number of Matches: 1

Range 1: 7 to 15GenPeptGraphics Next Match Previous Match

Alignment statistics for match #1

| Score | Expect | Identities | Positives | Gaps |
|---|---|---|---|---|
| 26.1 bits(54) | 2.2 | 7/9(78%) | 7/9(77%) | 0/9(0%) |

```
Query  9   ADSYEQYFG  17
           A  YEQYFG
Sbjct  7   AGTYEQYFG  15
```

CSADPSRLGGVSYEQYFG (one 8/10 and one 7/10)

myelin basic protein-specific T-cell receptor beta chain VDJ region {clone HOO24} [human, peripheral blood mononuclear cells, Peptide Partial, 25 aa] [Homo sapiens]

Sequence ID: AAB33029.1Length: 25Number of Matches: 1

Range 1: 9 to 17GenPeptGraphics Next Match Previous Match

Alignment statistics for match #1

| Score | Expect | Identities | Positives | Gaps |
|---|---|---|---|---|
| 29.1 bits(61) | 0.21 | 8/9(89%) | 8/9(88%) | 0/9(0%) |

```
Query  10  GVSYEQYFG  18
           G SYEQYFG
Sbjct  9   GASYEQYFG  17
```

myelin basic protein specific T-cell receptor V beta-D beta-J beta, MBP reactive TCR VDJ beta {CDR3 region, clone BM(5)} [human, peripheral blood lymphocytes, HLA phenotype 1, Peptide Partial, 23 aa] [Homo sapiens]

Sequence ID: AAB25666.1Length: 23Number of Matches: 1

Range 1: 9 to 15GenPeptGraphics Next Match Previous Match

Alignment statistics for match #1

| Score | Expect | Identities | Positives | Gaps |
|---|---|---|---|---|
| 27.4 bits(57) | 0.84 | 7/7(100%) | 7/7(100%) | 0/7(0%) |

```
Query  12  SYEQYFG  18
           SYEQYFG
Sbjct  9   SYEQYFG  15
```

CSARRTETRPWEDSYEQYFG (one 8/10)

myelin basic protein specific T-cell receptor V beta-D beta-J beta, MBP reactive TCR VDJ beta {CDR3 region, clone BM(5)} [human, peripheral blood lymphocytes, HLA phenotype 1, Peptide Partial, 23 aa] [Homo sapiens]

Sequence ID: AAB25666.1Length: 23Number of Matches: 1

Range 1: 7 to 15GenPeptGraphics Next Match Previous Match

Alignment statistics for match #1

| Score | Expect | Identities | Positives | Gaps |
|---|---|---|---|---|
| 29.5 bits(62) | 0.17 | 8/9(89%) | 8/9(88%) | 0/9(0%) |

```
Query  12  EDSYEQYFG  20
           E SYEQYFG
Sbjct  7   EASYEQYFG  15
```

**Zeng W, Maciejewski JP, Chen G, Young NS. Limited heterogeneity of T cell receptor BV usage in aplastic anemia. J Clin Invest. 2001 Sep;108(5):765-73.   (29)**

Normal 2

CASSRTGKDYGYTFG (one 7/10)

myelin basic protein specific T-cell receptor V beta-D beta-J beta, MBP reactive TCR VDJ beta {clone KL-3(6), rearranged CDR3 region} [human, brain plaques, HLA phenotype 1, Peptide Partial, 24 aa] [Homo sapiens]

Sequence ID: AAB25609.1Length: 24Number of Matches: 1

Range 1: 2 to 16GenPeptGraphics Next Match Previous Match

Alignment statistics for match #1

| Score | Expect | Identities | Positives | Gaps |
|---|---|---|---|---|
| 28.2 bits(59) | 0.28 | 12/16(75%) | 12/16(75%) | 2/16(12%) |

```
Query  1   CASS-RTGKDYGYTFG  15
           CASS R G  YGYTFG
Sbjct  2   CASSLR-GTPYGYTFG  16
```

CASSGGTRDNTEAFFG (one 7/10)

TCR V beta {V-J junction, clone MS9-2C5} [human, multiple sclerosis patient, myelin basic protein-reactive T cells after vaccination, Peptide Partial, 30 aa] [Homo sapiens]

Sequence ID: AAB35724.1Length: 30Number of Matches: 1

Range 1: 11 to 17GenPeptGraphics Next Match Previous Match

Alignment statistics for match #1

| Score | Expect | Identities | Positives | Gaps |
|---|---|---|---|---|
| 25.7 bits(53) | 3.4 | 7/7(100%) | 7/7(100%) | 0/7(0%) |

```
Query  10  NTEAFFG  16
           NTEAFFG
Sbjct  11  NTEAFFG  17
```

CASSCSHPRQKNIQYFG (two 8/10)

myelin basic protein specific T-cell receptor V beta-D beta-J beta, MBP reactive TCR VDJ beta {CDR3 region, clone JO(3)} [human, normal peripheral blood lymphocytes, Peptide Partial, 23 aa] [Homo sapiens]

Sequence ID: AAB25658.1Length: 23Number of Matches: 1

Range 1: 2 to 15GenPeptGraphics Next Match Previous Match

Alignment statistics for match #1

| Score | Expect | Identities | Positives | Gaps |
|---|---|---|---|---|

31.6 bits(67) 0.019 12/17(71%) 12/17(70%) 3/17(17%)

```
Query  1    CASSCSHPRQKNIQYFG  17
            CASS    P  KNIQYFG
Sbjct  2    CASS---PGMKNIQYFG  15
```

myelin basic protein specific T-cell receptor V beta-D beta-J beta, MBP reactive TCR VDJ beta {CDR3 region, clone BM(3)} [human, peripheral blood lymphocytes, HLA phenotype 1, Peptide Partial, 27 aa] [Homo sapiens]

Sequence ID: AAB25664.1Length: 27Number of Matches: 1

Range 1: 2 to 19GenPeptGraphics Next Match Previous Match

Alignment statistics for match #1

| Score | Expect | Identities | Positives | Gaps |
|---|---|---|---|---|
| 29.5 bits(62) | 0.14 | 13/18(72%) | 13/18(72%) | 1/18(5%) |

```
Query  1    CASSCSH-PRQKNIQYFG  17
            CASS  H P  KNIQYFG
Sbjct  2    CASSLEHRPTAKNIQYFG  19
```

CASSLSRGPGTDTQYFG (two 7/10)

myelin basic protein (143-168)-specific TCR beta-chain {clone BM3.3} [human, multiple sclerosis patient, T cells, Peptide Partial, 26 aa] [Homo sapiens]

Sequence ID: AAB31427.1Length: 26Number of Matches: 1

Range 1: 1 to 14GenPeptGraphics Next Match Previous Match

Alignment statistics for match #1

| Score | Expect | Identities | Positives | Gaps |
|---|---|---|---|---|
| 31.2 bits(66) | 0.032 | 12/17(71%) | 12/17(70%) | 3/17(17%) |

```
Query  1    CASSLSRGPGTDTQYFG  17
            CASSL RG    TQYFG
Sbjct  1    CASSLTRG---YTQYFG  14
```

myelin basic protein specific T-cell receptor V beta-D beta-J beta, MBP reactive TCR VDJ beta {clone KL-3(9), rearranged CDR3 region} [human, brain plaques, HLA phenotype 1, Peptide Partial, 25 aa] [Homo sapiens]

Sequence ID: AAB25612.1Length: 25Number of Matches: 1

Range 1: 2 to 17GenPeptGraphics Next Match Previous Match

Alignment statistics for match #1

| Score | Expect | Identities | Positives | Gaps |
|---|---|---|---|---|
| 30.8 bits(65) | 0.043 | 12/17(71%) | 12/17(70%) | 1/17(5%) |

```
Query  1    CASSLSRGPGTDTQYFG  17
            CASSL  G   DTQYFG
Sbjct  2    CASSLG-GSEEDTQYFG  17
```

CASSIRDTGEQFFG (0)

None

CASSPGEWGEQFFG (0)

None

CASSPGDTGETQYFG (three 7/10)

myelin basic protein specific T-cell receptor V beta-D beta-J beta, MBP reactive TCR VDJ beta {clone LJ 1(2), rearranged CDR3 region} [human, brain plaques, HLA phenotype 1, Peptide Partial, 22 aa] [Homo sapiens]

Sequence ID: AAB25628.1Length: 22Number of Matches: 1

Range 1: 2 to 14GenPeptGraphics Next Match Previous Match

Alignment statistics for match #1

| Score | Expect | Identities | Positives | Gaps |
|---|---|---|---|---|
| 33.3 bits(71) | 0.003 | 11/15(73%) | 11/15(73%) | 2/15(13%) |

```
Query  1   CASSPGDTGETQYFG  15
           CASS  D  ETQYFG
Sbjct  2   CASS--DSSETQYFG  14
```

myelin basic protein specific T-cell receptor V beta-D beta-J beta, MBP reactive TCR VDJ beta {CDR3 region, clone JO(5)} [human, normal peripheral blood lymphocytes, Peptide Partial, 25 aa] [Homo sapiens]

Sequence ID: AAB25660.1Length: 25Number of Matches: 1

Range 1: 2 to 17GenPeptGraphics Next Match Previous Match

Alignment statistics for match #1

| Score | Expect | Identities | Positives | Gaps |
|---|---|---|---|---|
| 33.3 bits(71) | 0.004 | 12/16(75%) | 13/16(81%) | 1/16(6%) |

```
Query  1   CASS-PGDTGETQYFG  15
           CASS PG+   ETQYFG
Sbjct  2   CASSRPGNIRETQYFG  17
```

myelin basic protein specific T-cell receptor V beta-D beta-J beta, MBP reactive TCR VDJ beta {clone KL-3(10), rearranged CDR3 region} [human, brain plaques, HLA phenotype 1, Peptide Partial, 24 aa] [Homo sapiens]

Sequence ID: AAB25613.1Length: 24Number of Matches: 1

Range 1: 2 to 16GenPeptGraphics Next Match Previous Match

Alignment statistics for match #1

| Score | Expect | Identities | Positives | Gaps |
|---|---|---|---|---|
| 32.0 bits(68) | 0.010 | 11/15(73%) | 11/15(73%) | 0/15(0%) |

```
Query  1   CASSPGDTGETQYFG  15
           CASS G    ETQYFG
Sbjct  2   CASSLGGSEETQYFG  16
```

CASSTRDSGGYTFG (0)

None

CASSRVPQHFG (0)

None

CASSPGEKDTQYFG (two 7/10)

myelin basic protein specific T-cell receptor V beta-D beta-J beta, MBP reactive TCR VDJ beta {CDR3 region, clone JO(3)} [human, normal peripheral blood lymphocytes, Peptide Partial, 23 aa] [Homo sapiens]

Sequence ID: AAB25658.1Length: 23Number of Matches: 1

Range 1: 2 to 15GenPeptGraphics Next Match Previous Match

Alignment statistics for match #1

| Score | Expect | Identities | Positives | Gaps |
|---|---|---|---|---|
| 35.0 bits(75) | 6e-04 | 11/14(79%) | 12/14(85%) | 0/14(0%) |

```
Query  1    CASSPGEKDTQYFG  14
            CASSPG K+ QYFG
Sbjct  2    CASSPGMKNIQYFG  15
```

myelin basic protein specific T-cell receptor V beta-D beta-J beta, MBP reactive TCR VDJ beta {clone KL-3(9), rearranged CDR3 region} [human, brain plaques, HLA phenotype 1, Peptide Partial, 25 aa] [Homo sapiens]

Sequence ID: AAB25612.1Length: 25Number of Matches: 1

Range 1: 2 to 17GenPeptGraphics Next Match Previous Match

Alignment statistics for match #1

| Score | Expect | Identities | Positives | Gaps |
|---|---|---|---|---|
| 32.0 bits(68) | 0.009 | 12/16(75%) | 12/16(75%) | 2/16(12%) |

```
Query  1    CASSPG--EKDTQYFG  14
            CASS G  E DTQYFG
Sbjct  2    CASSLGGSEEDTQYFG  17
```

CASSSQGYTGELFFG (one 8/10 and one 7/10)

myelin basic protein-specific T-cell receptor beta chain VDJ region {clone NN2.23} [human, peripheral blood mononuclear cells, Peptide Partial, 25 aa] [Homo sapiens]

Sequence ID: AAB33028.1Length: 25Number of Matches: 1

Range 1: 1 to 17GenPeptGraphics Next Match Previous Match

Alignment statistics for match #1

| Score | Expect | Identities | Positives | Gaps |
|---|---|---|---|---|
| 29.9 bits(63) | 0.068 | 12/17(71%) | 12/17(70%) | 2/17(11%) |

```
Query  1    CASSSQ-GY-TGELFFG  15
            CASS     Y TGELFFG
Sbjct  1    CASSRRDSYKTGELFFG  17
```

myelin basic protein specific T-cell receptor V beta-D beta-J beta, MBP reactive TCR VDJ beta {clone SE(8), rearranged CDR3 region} [human, inflammatory brain lesions, HLA phenotype 1, Peptide Partial, 24 aa] [Homo sapiens]

Sequence ID: AAB25642.1Length: 24Number of Matches: 1

Range 1: 2 to 16GenPeptGraphics Next Match Previous Match

---

Alignment statistics for match #1

| Score | Expect | Identities | Positives | Gaps |
|---|---|---|---|---|
| 27.8 bits(58) | 0.41 | 11/15(73%) | 11/15(73%) | 0/15(0%) |

```
Query  1    CASSSQGYTGELFFG  15
            CASS  G  GELFFG
Sbjct  2    CASSTSGQPGELFFG  16
```

CASSRDGYEQFFG (two 7/10)

myelin basic protein-specific T-cell receptor beta chain VDJ region {clone NN2.23} [human, peripheral blood mononuclear cells, Peptide Partial, 25 aa] [Homo sapiens]

Sequence ID: AAB33028.1Length: 25Number of Matches: 1

Range 1: 1 to 17GenPeptGraphics Next Match Previous Match

Alignment statistics for match #1

| Score | Expect | Identities | Positives | Gaps |
|---|---|---|---|---|
| 27.4 bits(57) | 0.45 | 11/17(65%) | 11/17(64%) | 4/17(23%) |

```
Query  1    CASS-RDGY---EQFFG  13
            CASS RD Y   E FFG
Sbjct  1    CASSRRDSYKTGELFFG  17
```

Download GenPeptGraphics Next Previous Descriptions

myelin basic protein specific T-cell receptor V beta-D beta-J beta, MBP reactive TCR VDJ beta {clone KL-3(8), rearranged CDR3 region} [human, brain plaques, HLA phenotype 1, Peptide Partial, 25 aa] [Homo sapiens]

Sequence ID: AAB25611.1Length: 25Number of Matches: 1

Range 1: 2 to 17GenPeptGraphics Next Match Previous Match

Alignment statistics for match #1

| Score | Expect | Identities | Positives | Gaps |
|---|---|---|---|---|
| 27.4 bits(57) | 0.45 | 11/16(69%) | 12/16(75%) | 3/16(18%) |

```
Query  1    CASSRD--GY-EQFFG  13
            CASS +  GY EQFFG
Sbjct  2    CASSLELAGYNEQFFG  17
```

CASSRFGDETQYFG (one 7/10)

myelin basic protein (143-168)-specific TCR beta-chain {clone JA.1.7} [human, multiple sclerosis patient, T cells, Peptide Partial, 27 aa] [Homo sapiens]

Sequence ID: AAB31429.1Length: 27Number of Matches: 1

Range 1: 1 to 15GenPeptGraphics Next Match Previous Match

Alignment statistics for match #1

| Score | Expect | Identities | Positives | Gaps |
|---|---|---|---|---|
| 29.1 bits(61) | 0.13 | 11/17(65%) | 12/17(70%) | 5/17(29%) |

```
Query  1    CASSRF---GDETQYFG  14
```

```
            CASS     G+ETQYFG
Sbjct  1    CASS--LDGGEETQYFG  15
```

**Mamadov, Britanova, Bolotin, et al., EMBO Mol Med 2011; 3: 201-207 (1 patient)**

CASSVALGLNYEQYF (one 7/10)

myelin basic protein specific T-cell receptor V beta-D beta-J beta, MBP reactive TCR VDJ beta {clone LJ 1(6), rearranged CDR3 region} [human, brain plaques, HLA phenotype 1, Peptide Partial, 24 aa] [Homo sapiens]

Sequence ID: AAB25632.1Length: 24Number of Matches: 1

Range 1: 2 to 15GenPeptGraphics Next Match Previous Match

Alignment statistics for match #1

| Score | Expect | Identities | Positives | Gaps |
|---|---|---|---|---|
| 29.1 bits(61) | 0.14 | 12/16(75%) | 12/16(75%) | 3/16(18%) |

```
Query  1    CASS-VALGLNYEQYF  15
            CASS VA G  YEQYF
Sbjct  2    CASSLVA-GI-YEQYF  15
```

CASSLSGGAGELFF (one 7/10)

myelin basic protein specific T-cell receptor V beta-D beta-J beta, MBP reactive TCR VDJ beta {clone SE(8), rearranged CDR3 region} [human, inflammatory brain lesions, HLA phenotype 1, Peptide Partial, 24 aa] [Homo sapiens]

Sequence ID: AAB25642.1Length: 24Number of Matches: 1

Range 1: 2 to 15GenPeptGraphics Next Match Previous Match

Alignment statistics for match #1

| Score | Expect | Identities | Positives | Gaps |
|---|---|---|---|---|
| 29.9 bits(63) | 0.056 | 11/14(79%) | 11/14(78%) | 0/14(0%) |

```
Query  1    CASSLSGGAGELFF  14
            CASS SG  GELFF
Sbjct  2    CASSTSGQPGELFF  15
```

CAIKTTSGIVDEQFF (one 7/10)

myelin basic protein (84-102)-specific TCR beta-chain {clone Ob.2F3,1C3,1E10,1H8,2G9} [human, multiple sclerosis patient, T cells, Peptide Partial, 29 aa] [Homo sapiens]

Sequence ID: AAB31426.1Length: 29Number of Matches: 1

Range 1: 7 to 16GenPeptGraphics Next Match Previous Match

Alignment statistics for match #1

| Score | Expect | Identities | Positives | Gaps |
|---|---|---|---|---|
| 22.7 bits(46) | 34 | 7/10(70%) | 8/10(80%) | 0/10(0%) |

```
Query  6    TSGIVDEQFF  15
```

```
            TSG   +EQFF
Sbjct  7    TSGSLNEQFF  16
```

CASSLWEGREQFF (one 7/10)

myelin basic protein specific T-cell receptor V beta-D beta-J beta, MBP reactive TCR VDJ beta {clone KL-1(11), rearranged CDR3 region} [human, brain plaques, HLA phenotype 1, Peptide Partial, 24 aa] [Homo sapiens]

Sequence ID: AAB25602.1Length: 24Number of Matches: 1

Range 1: 2 to 15GenPeptGraphics Next Match Previous Match

Alignment statistics for match #1

| Score | Expect | Identities | Positives | Gaps |
|---|---|---|---|---|
| 23.1 bits(47) | 17 | 9/14(64%) | 10/14(71%) | 1/14(7%) |

```
Query  1    CASSL-WEGREQFF  13
            CASS   EGR Q+F
Sbjct  2    CASSSEREGRAQYF  15
```

CSVGTSEAYEQYF (two 7/10)

myelin basic protein specific T-cell receptor V beta-D beta-J beta, MBP reactive TCR VDJ beta {CDR3 region, clone BM(5)} [human, peripheral blood lymphocytes, HLA phenotype 1, Peptide Partial, 23 aa] [Homo sapiens]

Sequence ID: AAB25666.1Length: 23Number of Matches: 1

Range 1: 7 to 14GenPeptGraphics Next Match Previous Match

Alignment statistics for match #1

| Score | Expect | Identities | Positives | Gaps |
|---|---|---|---|---|
| 24.0 bits(49) | 7.8 | 7/8(88%) | 7/8(87%) | 1/8(12%) |

```
Query  7    EA-YEQYF  13
            EA YEQYF
Sbjct  7    EASYEQYF  14
```

myelin basic protein specific T-cell receptor V beta-D beta-J beta, MBP reactive TCR VDJ beta {clone SE(12), rearranged CDR3 region} [human, inflammatory brain lesions, HLA phenotype 1, Peptide Partial, 25 aa] [Homo sapiens]

Sequence ID: AAB25646.1Length: 25Number of Matches: 1

Range 1: 7 to 15GenPeptGraphics Next Match Previous Match

Alignment statistics for match #1

| Score | Expect | Identities | Positives | Gaps |
|---|---|---|---|---|
| 22.7 bits(46) | 24 | 7/9(78%) | 7/9(77%) | 0/9(0%) |

```
Query  4    GTSEAYEQY  12
            GTS  YEQY
Sbjct  7    GTSGIYEQY  15
```

CSARTTYGTDIISQHF (0)

None

CSVEIWDSSYNEQFF (one 7/10)

myelin basic protein (84-102)-specific TCR beta-chain {clone NSJL5.7} [human, multiple sclerosis patient, T cells, Peptide Partial, 28 aa] [Homo sapiens]

Sequence ID: AAB31431.1Length: 28Number of Matches: 1

Range 1: 9 to 15GenPeptGraphics Next Match Previous Match

Alignment statistics for match #1

| Score | Expect | Identities | Positives | Gaps |
|---|---|---|---|---|
| 27.8 bits(58) | 0.47 | 7/7(100%) | 7/7(100%) | 0/7(0%) |

```
Query  9    SYNEQFF  15
            SYNEQFF
Sbjct  9    SYNEQFF  15
```

CASSLAPGATNEKLFF (one 8/10 and one 7/10)

myelin basic protein specific T-cell receptor V beta-D beta-J beta, MBP TCR reactive VDJ beta {CDR3 region, muscle infiltrating lymphocytes(1)} [human, muscle infiltrating lymphocytes, HLA phenotype 1, Peptide Partial, 28 aa] [Homo sapiens]

Sequence ID: AAB25649.1Length: 28Number of Matches: 1

Range 1: 2 to 19GenPeptGraphics Next Match Previous Match

Alignment statistics for match #1

| Score | Expect | Identities | Positives | Gaps |
|---|---|---|---|---|
| 35.8 bits(77) | 5e-04 | 14/18(78%) | 14/18(77%) | 2/18(11%) |

```
Query  1    CASSL-APG-ATNEKLFF  16
            CASSL  PG  TNEKLFF
Sbjct  2    CASSLGSPGYRTNEKLFF  19
```

myelin basic protein specific T-cell receptor V beta-D beta-J beta, MBP reactive TCR VDJ beta {clone KL-3(13), rearranged CDR3 region} [human, brain plaques, HLA phenotype 1, Peptide Partial, 26 aa] [Homo sapiens]

Sequence ID: AAB25616.1Length: 26Number of Matches: 1

Range 1: 2 to 9GenPeptGraphics Next Match Previous Match

Alignment statistics for match #1

| Score | Expect | Identities | Positives | Gaps |
|---|---|---|---|---|
| 22.3 bits(45) | 53 | 7/8(88%) | 7/8(87%) | 0/8(0%) |

```
Query  1   CASSLAPG  8
           CASSLA G
Sbjct  2   CASSLASG  9
```

CASSLGENIQYF (one 7/10)

myelin basic protein specific T-cell receptor V beta-D beta-J beta, MBP reactive TCR VDJ beta {CDR3 region, clone JO(3)} [human, normal peripheral blood lymphocytes, Peptide Partial, 23 aa] [Homo sapiens]

Sequence ID: AAB25658.1Length: 23Number of Matches: 1

Range 1: 2 to 14GenPeptGraphics Next Match Previous Match

Alignment statistics for match #1

| Score | Expect | Identities | Positives | Gaps |
|---|---|---|---|---|
| 26.9 bits(56) | 0.52 | 10/13(77%) | 10/13(76%) | 1/13(7%) |

```
Query  1   CASSLG-ENIQYF  12
           CASS G  NIQYF
Sbjct  2   CASSPGMKNIQYF  14
```

CASTVDSLDTEAFF (0)

None

CASSLSGGAGELFF (one 7/10)

myelin basic protein specific T-cell receptor V beta-D beta-J beta, MBP reactive TCR VDJ beta {clone SE(8), rearranged CDR3 region} [human, inflammatory brain lesions, HLA phenotype 1, Peptide Partial, 24 aa] [Homo sapiens]

Sequence ID: AAB25642.1Length: 24Number of Matches: 1

Range 1: 2 to 15GenPeptGraphics Next Match Previous Match

Alignment statistics for match #1

| Score | Expect | Identities | Positives | Gaps |
|---|---|---|---|---|
| 29.9 bits(63) | 0.056 | 11/14(79%) | 11/14(78%) | 0/14(0%) |

```
Query  1   CASSLSGGAGELFF  14
           CASS SG  GELFF
Sbjct  2   CASSTSGQPGELFF  15
```

CASSPGLAASYNEQFF (one 8/10 and one 7/10)

myelin basic protein specific T-cell receptor V beta-D beta-J beta, MBP reactive TCR VDJ beta {clone KL-3(8), rearranged CDR3 region} [human, brain plaques, HLA phenotype 1, Peptide Partial, 25 aa] [Homo sapiens]

Sequence ID: AAB25611.1Length: 25Number of Matches: 1

Range 1: 2 to 16GenPeptGraphics Next Match Previous Match

Alignment statistics for match #1

| Score | Expect | Identities | Positives | Gaps |
|---|---|---|---|---|
| 32.9 bits(70) | 0.006 | 12/16(75%) | 12/16(75%) | 1/16(6%) |

```
Query  1   CASSPGLAASYNEQFF  16
           CASS  LA  YNEQFF
Sbjct  2   CASSLELAG-YNEQFF  16
```

myelin basic protein specific T-cell receptor V beta-D beta-J beta, MBP reactive TCR VDJ beta {clone KL-3(7), rearranged CDR3 region} [human, brain plaques, HLA phenotype 1, Peptide Partial, 24 aa] [Homo sapiens]

Sequence ID: AAB25610.1Length: 24Number of Matches: 1

Range 1: 2 to 15GenPeptGraphics Next Match Previous Match

Alignment statistics for match #1

| Score | Expect | Identities | Positives | Gaps |
|---|---|---|---|---|
| 31.2 bits(66) | 0.025 | 12/17(71%) | 12/17(70%) | 4/17(23%) |

```
Query  1    CASS-PGLAASYNEQFF  16
            CASS P    A YNEQFF
Sbjct  2    CASSQP---AVYNEQFF  15
```

CASSFKGADTQYF (one 7/10)

myelin basic protein specific T-cell receptor V beta-D beta-J beta, MBP reactive TCR VDJ beta {clone SE(9), rearranged CDR3 region} [human, inflammatory brain lesions, HLA phenotype 1, Peptide Partial, 25 aa] [Homo sapiens]

Sequence ID: AAB25643.1Length: 25Number of Matches: 1

Range 1: 2 to 16GenPeptGraphics Next Match Previous Match

Alignment statistics for match #1

| Score | Expect | Identities | Positives | Gaps |
|---|---|---|---|---|
| 27.8 bits(58) | 0.31 | 11/16(69%) | 11/16(68%) | 4/16(25%) |

```
Query  1    CASSF---KGADTQYF  13
            CASS     K ADTQYF
Sbjct  2    CASSLDLGK-ADTQYF  16
```

CASTVDSLDTEAFF (0)

None

CASSTAGVHYNEQFF (two 7/10)

myelin basic protein specific T-cell receptor V beta-D beta-J beta, MBP TCR reactive VDJ beta {CDR3 region, muscle infiltrating lymphocytes(2)} [human, muscle infiltrating lymphocytes, HLA phenotype 1, Peptide Partial, 25 aa] [Homo sapiens]

Sequence ID: AAB25650.1Length: 25Number of Matches: 1

Range 1: 2 to 16GenPeptGraphics Next Match Previous Match

Alignment statistics for match #1

| Score | Expect | Identities | Positives | Gaps |
|---|---|---|---|---|
| 33.3 bits(71) | 0.004 | 11/15(73%) | 11/15(73%) | 0/15(0%) |

```
Query  1    CASSTAGVHYNEQFF  15
            CASS  G   YNEQFF
Sbjct  2    CASSFTGAYYNEQFF  16
```

myelin basic protein specific T-cell receptor V beta-D beta-J beta, MBP reactive TCR VDJ beta {clone KL-3(7), rearranged CDR3 region} [human, brain plaques, HLA phenotype 1, Peptide Partial, 24 aa] [Homo sapiens]

Sequence ID: AAB25610.1Length: 24Number of Matches: 1

Range 1: 2 to 15GenPeptGraphics Next Match Previous Match

Alignment statistics for match #1

| Score | Expect | Identities | Positives | Gaps |
|---|---|---|---|---|
| 32.0 bits(68) | 0.010 | 11/15(73%) | 11/15(73%) | 1/15(6%) |

```
Query  1   CASSTAGVHYNEQFF  15
           CASS    V YNEQFF
Sbjct  2   CASSQPAV-YNEQFF  15
```

CASTSWAQASTDTQYF (0)

None

CASPHTGQSLPYGYTF (0)

None

CASSFYKSSEQYF (0)

None

CASSLDGYSPLHF (one 7/10)

myelin basic protein specific T-cell receptor V beta-D beta-J beta, MBP reactive TCR VDJ beta {clone KL-3(1), rearranged CDR3 region} [human, brain plaques, HLA phenotype 1, Peptide Partial, 24 aa] [Homo sapiens]

Sequence ID: AAB25604.1Length: 24Number of Matches: 1

Range 1: 2 to 8GenPeptGraphics Next Match Previous Match

Alignment statistics for match #1

| Score | Expect | Identities | Positives | Gaps |
|---|---|---|---|---|
| 24.0 bits(49) | 8.0 | 7/7(100%) | 7/7(100%) | 0/7(0%) |

```
Query  1   CASSLDG  7
           CASSLDG
Sbjct  2   CASSLDG  8
```

CASSLHGELFF (0)

None

CASGSGLAGGELFF (one 7/10)

myelin basic protein specific T-cell receptor V beta-D beta-J beta, MBP reactive TCR VDJ beta {clone SE(7), rearranged CDR3 region} [human, inflammatory brain lesions, HLA phenotype 1, Peptide Partial, 26 aa] [Homo sapiens]

Sequence ID: AAB25641.1Length: 26Number of Matches: 1

Range 1: 2 to 17GenPeptGraphics Next Match Previous Match

Alignment statistics for match #1

| Score | Expect | Identities | Positives | Gaps |
|---|---|---|---|---|
| 23.5 bits(48) | 14 | 10/16(63%) | 10/16(62%) | 2/16(12%) |

```
Query  1   CASGSGLAGG--ELFF  14
           CAS  GLAG    E FF
Sbjct  2   CASSWGLAGARDEQFF  17
```

CASSLVEAGDPYEQYF (one 8/10 and one 7/10)

myelin basic protein specific T-cell receptor V beta-D beta-J beta, MBP reactive TCR VDJ beta {clone LJ 1(6), rearranged CDR3 region} [human, brain plaques, HLA phenotype 1, Peptide Partial, 24 aa] [Homo sapiens]
Sequence ID: AAB25632.1Length: 24Number of Matches: 1
Range 1: 2 to 15GenPeptGraphics Next Match Previous Match

Alignment statistics for match #1

| Score | Expect | Identities | Positives | Gaps |
|---|---|---|---|---|
| 33.7 bits(72) | 0.003 | 13/16(81%) | 13/16(81%) | 2/16(12%) |

```
Query  1    CASSLVEAGDPYEQYF  16
            CASSLV AG  YEQYF
Sbjct  2    CASSLV-AGI-YEQYF  15
```

myelin basic protein-specific T-cell receptor beta chain VDJ region {clone HOO24} [human, peripheral blood mononuclear cells, Peptide Partial, 25 aa] [Homo sapiens]
Sequence ID: AAB33029.1Length: 25Number of Matches: 1
Range 1: 1 to 16GenPeptGraphics Next Match Previous Match

Alignment statistics for match #1

| Score | Expect | Identities | Positives | Gaps |
|---|---|---|---|---|
| 26.9 bits(56) | 1.0 | 12/18(67%) | 12/18(66%) | 4/18(22%) |

```
Query  1    CASS-LVEA-GDPYEQYF  16
            CASS L   A G  YEQYF
Sbjct  1    CASSSL--ARGASYEQYF  16
```

CASSLAPGATNEKLFF (one 8/10)
myelin basic protein specific T-cell receptor V beta-D beta-J beta, MBP TCR reactive VDJ beta {CDR3 region, muscle infiltrating lymphocytes(1)} [human, muscle infiltrating lymphocytes, HLA phenotype 1, Peptide Partial, 28 aa] [Homo sapiens]
Sequence ID: AAB25649.1Length: 28Number of Matches: 1
Range 1: 2 to 19GenPeptGraphics Next Match Previous Match

Alignment statistics for match #1

| Score | Expect | Identities | Positives | Gaps |
|---|---|---|---|---|
| 35.8 bits(77) | 5e-04 | 14/18(78%) | 14/18(77%) | 2/18(11%) |

```
Query  1    CASSL-APG-ATNEKLFF  16
            CASSL  PG  TNEKLFF
Sbjct  2    CASSLGSPGYRTNEKLFF  19
```

CASSLAGGTAHTEAFF (one 8/10)
myelin basic protein specific T-cell receptor V beta-D beta-J beta, MBP reactive TCR VDJ beta {clone KL-3(13), rearranged CDR3 region} [human, brain plaques, HLA phenotype 1, Peptide Partial, 26 aa] [Homo sapiens]
Sequence ID: AAB25616.1Length: 26Number of Matches: 1
Range 1: 2 to 10GenPeptGraphics Next Match Previous Match

Alignment statistics for match #1

| Score | Expect | Identities | Positives | Gaps |
|---|---|---|---|---|
| 25.2 bits(52) | 4.4 | 8/9(89%) | 8/9(88%) | 0/9(0%) |

```
Query  1  CASSLAGGT  9
          CASSLA GT
Sbjct  2  CASSLASGT  10
```

CASSLEASPTSDTQYF (two 8/10)

myelin basic protein specific T-cell receptor V beta-D beta-J beta, MBP reactive TCR VDJ beta {CDR3 region, clone BM(3)} [human, peripheral blood lymphocytes, HLA phenotype 1, Peptide Partial, 27 aa] [Homo sapiens]

Sequence ID: AAB25664.1Length: 27Number of Matches: 1

Range 1: 2 to 18GenPeptGraphics Next Match Previous Match

Alignment statistics for match #1

| Score | Expect | Identities | Positives | Gaps |
|---|---|---|---|---|
| 30.8 bits(65) | 0.041 | 11/17(65%) | 12/17(70%) | 1/17(5%) |

```
Query  1   CASSLEASPTS-DTQYF  16
           CASSLE  PT   + QYF
Sbjct  2   CASSLEHRPTAKNIQYF  18
```

myelin basic protein specific T-cell receptor V beta-D beta-J beta, MBP reactive TCR VDJ beta {clone KL-3(13), rearranged CDR3 region} [human, brain plaques, HLA phenotype 1, Peptide Partial, 26 aa] [Homo sapiens]

Sequence ID: AAB25616.1Length: 26Number of Matches: 1

Range 1: 2 to 17GenPeptGraphics Next Match Previous Match

Alignment statistics for match #1

| Score | Expect | Identities | Positives | Gaps |
|---|---|---|---|---|
| 28.2 bits(59) | 0.35 | 12/17(71%) | 13/17(76%) | 2/17(11%) |

```
Query  1   CASSLEASPT-SDTQYF  16
           CASSL AS T  +TQYF
Sbjct  2   CASSL-ASGTLQETQYF  17
```

CASSSTGDFSYEQYF (one 7/10)

myelin basic protein-specific T-cell receptor beta chain VDJ region {clone HOO24} [human, peripheral blood mononuclear cells, Peptide Partial, 25 aa] [Homo sapiens]

Sequence ID: AAB33029.1Length: 25Number of Matches: 1

Range 1: 1 to 16GenPeptGraphics Next Match Previous Match

Alignment statistics for match #1

| Score | Expect | Identities | Positives | Gaps |
|---|---|---|---|---|
| 29.1 bits(61) | 0.14 | 12/17(71%) | 12/17(70%) | 3/17(17%) |

```
Query  1    CASSS--TGDFSYEQYF  15
            CASSS   G  SYEQYF
Sbjct  1    CASSSLARGA-SYEQYF  16
```

CASSPHYTDTQYF (0)

None

CASSVALGLNYEQYF (0)

None

CASSVETGDLAFETQYF (one 7/10)

myelin basic protein specific T-cell receptor V beta-D beta-J beta, MBP reactive TCR VDJ beta {clone KL-3(13), rearranged CDR3 region} [human, brain plaques, HLA phenotype 1, Peptide Partial, 26 aa] [Homo sapiens]

Sequence ID: AAB25616.1Length: 26Number of Matches: 1

Range 1: 2 to 17GenPeptGraphics Next Match Previous Match

Alignment statistics for match #1

| Score | Expect | Identities | Positives | Gaps |
|---|---|---|---|---|
| 28.6 bits(60) | 0.28 | 11/17(65%) | 11/17(64%) | 1/17(5%) |

```
Query  1    CASSVETGDLAFETQYF  17
            CASS    G L  ETQYF
Sbjct  2    CASSLASGTLQ-ETQYF  17
```

CASSVVGAADTQYF (one 7/10)

myelin basic protein specific T-cell receptor V beta-D beta-J beta, MBP reactive TCR VDJ beta {clone SE(9), rearranged CDR3 region} [human, inflammatory brain lesions, HLA phenotype 1, Peptide Partial, 25 aa] [Homo sapiens]

Sequence ID: AAB25643.1Length: 25Number of Matches: 1

Range 1: 2 to 16GenPeptGraphics Next Match Previous Match

Alignment statistics for match #1

| Score | Expect | Identities | Positives | Gaps |
|---|---|---|---|---|
| 29.1 bits(61) | 0.12 | 11/15(73%) | 11/15(73%) | 1/15(6%) |

```
Query  1    CASSV-VGAADTQYF  14
            CASS    G ADTQYF
Sbjct  2    CASSLDLGKADTQYF  16
```

CASSSGQLSNTGELFF (one 7/10)

myelin basic protein specific T-cell receptor V beta-D beta-J beta, MBP reactive TCR VDJ beta {clone SE(8), rearranged CDR3 region} [human, inflammatory brain lesions, HLA phenotype 1, Peptide Partial, 24 aa] [Homo sapiens]

Sequence ID: AAB25642.1Length: 24Number of Matches: 1

Range 1: 2 to 15GenPeptGraphics Next Match Previous Match

Alignment statistics for match #1

| Score | Expect | Identities | Positives | Gaps |
|---|---|---|---|---|

29.1 bits(61) 0.16 12/17(71%) 12/17(70%) 4/17(23%)

```
Query  1    CASS-SGQLSNTGELFF  16
            CASS SGQ    GELFF
Sbjct  2    CASSTSGQ---PGELFF  15
```

**Forman, Klein, Sliver, at al., J Clin Invest 1994; 94: 1533-1542. (1 patient)**

CASKLNYNIHEQFFG (one 7/10)

myelin basic protein specific T-cell receptor V beta-D beta-J beta, MBP reactive TCR VDJ beta {clone KL-1(8), rearranged CDR3 region} [human, brain plaques, HLA phenotype 1, Peptide Partial, 25 aa] [Homo sapiens]

Sequence ID: AAB25599.1Length: 25Number of Matches: 1

Range 1: 10 to 17GenPeptGraphics Next Match Previous Match

Alignment statistics for match #1

| Score | Expect | Identities | Positives | Gaps |
|---|---|---|---|---|
| 23.5 bits(48) | 16 | 7/9(78%) | 7/9(77%) | 1/9(11%) |

```
Query  7    YNIHEQFFG  15
            YN  EQFFG
Sbjct  10   YN-NEQFFG  17
```

CASTVPGQGETEAFFG (one 7/10)

TCR V beta {V-J junction, clone MS9-2C5} [human, multiple sclerosis patient, myelin basic protein-reactive T cells after vaccination, Peptide Partial, 30 aa] [Homo sapiens]

Sequence ID: AAB35724.1Length: 30Number of Matches: 1

Range 1: 3 to 17GenPeptGraphics Next Match Previous Match

Alignment statistics for match #1

| Score | Expect | Identities | Positives | Gaps |
|---|---|---|---|---|
| 27.4 bits(57) | 0.81 | 12/17(71%) | 12/17(70%) | 3/17(17%) |

```
Query  1    CASTVPGQG-ETEAFFG  16
            CAS   GQG  TEAFFG
Sbjct  3    CASL--GQGWNTEAFFG  17
```

# S4: Full results of BLAST Search Comparing Human Myelin Basic Protein with Clostridium perfringens

P02686:RecName: Full=Myelin basic protein;...

RID
PYGZSRXB013 Search expires on 12-06 22:38 pm

- Download All

Program
BLASTP Help

- Citation

Database
nr

- See details

Query ID
P02686.3
Description
RecName: Full=Myelin basic protein; Short=MBP; AltName: Full=Myelin A1 protein; AltName: Full=Myelin membrane encephalitogenic protein [Homo sapiens] ...
Molecule type
amino acid
Query Length
304
Other reports
Distance tree of results Multiple alignment MSA viewer Help

## Filter Results

Organism only top 20 will appear
exclude
Add organism

---

Percent Identity from Percent Identity to
E value from E value to
Query Coverage from Query Coverage to

Compare these results against the new Clustered nr database Help

-
- - - - - Alignment view
- - Help
- - 
-

49 sequences selected

- Help

Download GenPeptGraphics Next Previous Descriptions

polyphosphate:AMP phosphotransferase [uncultured Clostridium sp.]
Sequence ID: SCJ29218.1Length: 491Number of Matches: 1
Related Information
Gene-associated gene details
AlphaFold Structure-3D structure displays
Identical Proteins-Identical proteins to WP_075703452.1
Range 1: 68 to 138GenPeptGraphics Next Match Previous Match

Alignment statistics for match #1

| Score | Expect | Method | Identities | Positives | Gaps |
|---|---|---|---|---|---|
| 33.6 bits(69) | 18 | Compositional matrix adjust. | 25/71(35%) | 32/71(45%) | 5/71(7%) |

```
Query  72   DPK--NAWQDAHPADPGSRPHLIRLFSRDAPGREDNTFKDRPSESDELQTIQEDSAATS-  128
            DP+    W  A P D   R  L+  F RD P R +     DR    S  L   ++ED AA S
Sbjct  68   DPRGYQVWSYADPTDEEGRRPLLWSFWRDLPPRGEMAVLDRSWYSRALTRLEEDRAAASG  127

Query  129  --ESLDVMASQ  137
              ES++    Q
Sbjct  128  LVESINTFERQ  138
```

Download GenPeptGraphics Next Previous Descriptions

cell wall-binding repeat-containing protein [Clostridium sp.]
Sequence ID: WP_291665806.1Length: 997Number of Matches: 1
Range 1: 49 to 117GenPeptGraphics Next Match Previous Match

Alignment statistics for match #1

| Score | Expect | Method | Identities | Positives | Gaps |
|---|---|---|---|---|---|
| 34.0 bits(70) | 19 | Composition-based stats. | 19/69(28%) | 38/69(55%) | 8/69(11%) |

```
Query  8    RELNAEKASTNSETNRGESEKKRNLG--ELSRTTSEDNEVFGEA------DANQNNGTSS  59
```

```
               + +NAE+ + +S T   E+ ++   G  ++ + T E NE+ GE       + ++NN  +S
Sbjct  49    QSINAEENADDSVTKGNEAAEESTTGKNDINKPTEEQNEITGEVKNEDKENVDENNVNTS  108

Query  60    QDTAVTDSK  68
              +T + +SK
Sbjct  109   LETTLEESK  117
```

Download GenPeptGraphics Next Previous Descriptions

cell wall-binding repeat-containing protein [Clostridium sp.]

Sequence ID: MBS5937893.1Length: 996Number of Matches: 1

Range 1: 48 to 116GenPeptGraphics Next Match Previous Match

Alignment statistics for match #1

| Score | Expect | Method | Identities | Positives | Gaps |
|---|---|---|---|---|---|
| 33.6 bits(69) | 19 | Composition-based stats. | 19/69(28%) | 38/69(55%) | 8/69(11%) |

```
Query  8     RELNAEKASTNSETNRGESEKKRNLG--ELSRTTSEDNEVFGEA------DANQNNGTSS  59
             + +NAE+ + +S T   E+ ++   G  ++ + T E NE+ GE       + ++NN  +S
Sbjct  48    QSINAEENADDSVTKGNEAAEESTTGKNDINKPTEEQNEITGEVKNEDKENVDENNVNTS  107

Query  60    QDTAVTDSK  68
              +T + +SK
Sbjct  108   LETTLEESK  116
```

Download GenPeptGraphics Next Previous Descriptions

hypothetical protein [Clostridium perfringens]

Sequence ID: MDJ8959718.1Length: 1172Number of Matches: 1

Range 1: 383 to 508GenPeptGraphics Next Match Previous Match

Alignment statistics for match #1

| Score | Expect | Method | Identities | Positives | Gaps |
|---|---|---|---|---|---|
| 33.1 bits(68) | 31 | Composition-based stats. | 32/139(23%) | 59/139(42%) | 24/139(17%) |

```
Query  12    AEKASTNSETNRGESEKKRNLGELSRTTSEDNEVFGEADAN--------QNNGTSSQDTA  63
             +EKA  ++E  RG +E +R   E  R  SE   V  E + N        + N T +Q+
Sbjct  383   SEKARNDAEELRGTAESERVKSEKLRVDSEKARVLAEEERNTTFTQKEEERNNTFTQNET  442

Query  64    ---VTDSKRTADPKNAWQDAHPADPGSRPHLIRLFSRDAPGREDNTFKDRPSESDELQTI  120
                 T ++   + K A ++A  A   +    +++ +                 E+++L+ +
Sbjct  443   NRNATFTQSEEERKTAFEEAENARKVAESKRVKVETSRV-------------EAEKLRVV  489

Query  121   QEDSAATSESLDVMASQKR  139
              E     +ESL V A ++R
Sbjct  490   AETGRVEAESLRVTAEEER  508
```

Download GenPeptGraphics
Sort by:

Next Previous Descriptions

hypothetical protein [Clostridium perfringens]

Sequence ID: MDU7726947.1Length: 1097Number of Matches: 3

Range 1: 263 to 301GenPeptGraphics Next Match Previous Match

Alignment statistics for match #1

| Score | Expect | Method | Identities | Positives | Gaps |
|---|---|---|---|---|---|
| 32.7 bits(67) | 38 | Composition-based stats. | 20/41(49%) | 25/41(60%) | 2/41(4%) |

```
Query  9    ELNAEKASTNSETNRGESEKKRNLGELSRTTSEDNEVFGEA  49
            + N+E A T+ E NR ESEK R L E  R +SE+  V  EA
Sbjct  263  KFNSE-AKTD-ELNRKESEKNRVLAEEERKSSEEARVSAEA  301
```

Range 2: 389 to 451GenPeptGraphics Next Match Previous Match First Match

Alignment statistics for match #2

| Score | Expect | Method | Identities | Positives | Gaps |
|---|---|---|---|---|---|
| 31.4 bits(64) | 106 | Composition-based stats. | 20/63(32%) | 28/63(44%) | 4/63(6%) |

```
Query  12   AEKASTNSETNRGESEKKRNLGELSRTTSEDNEVFGEADAN----QNNGTSSQDTAVTDS  67
            +EKA  ++E  R  SE +R   E  R  SE   V  E + N    QN      +   V +
Sbjct  389  SEKARNDAEELRVTSENERTTAERLRVDSEKARVLAEEERNNTFTQNETNRNETFEVAEE  448

Query  68   KRT  70
            +RT
Sbjct  449  ERT  451
```

Range 3: 297 to 421GenPeptGraphics Next Match Previous Match First Match

Alignment statistics for match #3

| Score | Expect | Method | Identities | Positives | Gaps |
|---|---|---|---|---|---|
| 28.8 bits(58) | 690 | Composition-based stats. | 33/143(23%) | 61/143(42%) | 31/143(21%) |

```
Query  10   LNAEKASTNSETNRGESEKKRNLGELSRTTSEDNEVFGEADANQNNGTSSQD----TAVT  65
            + AE     N+ T+  ESE++R   E+ R  +E   V  EA+  ++N  S ++     T+
Sbjct  297  VSAEAIRQNTYTDFNESEEERKSNEIKRQEAEALRV--EAETRRDNLFSEKEEERNTSFI  354

Query  66   DSKRTADPK-NAWQDAHPADPGSRPHLIRLFSRDAPGREDNTFKDRP--------SESDE  116
            +S++   +   N  QD                SR+     E  T ++             +++E
Sbjct  355  ESEKVRNAAFNKAQD----------------SRNTTFEESETIREEAFKISEKARNDAEE  398

Query  117  LQTIQEDSAATSESLDVMASQKR  139
```

```
             L+   E+   T+E L V + + R
Sbjct  399   LRVTSENERTTAERLRVDSEKAR  421
```

---

Download GenPeptGraphics Next Previous Descriptions

hypothetical protein [Clostridium perfringens]

Sequence ID: MDM0998687.1Length: 1164Number of Matches: 1

Range 1: 388 to 446GenPeptGraphics Next Match Previous Match

Alignment statistics for match #1

| Score | Expect | Method | Identities | Positives | Gaps |
|---|---|---|---|---|---|
| 32.3 bits(66) | 48 | Composition-based stats. | 20/59(34%) | 29/59(49%) | 6/59(10%) |

```
Query  3     NHAGKRELNAEKASTNSETNRGESEKKRNLGELSRTTS------EDNEVFGEADANQNN  55
             N A +   + AE+    SE  R +SEK R L E  R T+      E N  F + + N+N+
Sbjct  388   NDAEELRVTAERERVKSEKLRVDSEKARVLAEEERNTTFTQKEEERNNTFTQNETNRND  446
```

Download GenPeptGraphics Next Previous Descriptions

hypothetical protein [Clostridium perfringens]

Sequence ID: MDK0563172.1Length: 1194Number of Matches: 1

Range 1: 388 to 446GenPeptGraphics Next Match Previous Match

Alignment statistics for match #1

| Score | Expect | Method | Identities | Positives | Gaps |
|---|---|---|---|---|---|
| 31.9 bits(65) | 73 | Composition-based stats. | 20/59(34%) | 28/59(47%) | 6/59(10%) |

```
Query  3     NHAGKRELNAEKASTNSETNRGESEKKRNLGELSRTTS------EDNEVFGEADANQNN  55
             N A +   + AE     SE  R +SEK R L E  R T+      E N  F + + N+N+
Sbjct  388   NDAEELRVTAESERVKSEKLRVDSEKARVLAEEERNTTFTQKEEERNNTFTQNETNRND  446
```

Download GenPeptGraphics Next Previous Descriptions

hypothetical protein [Clostridium perfringens]

Sequence ID: WP_221346387.1Length: 1129Number of Matches: 1

Range 1: 279 to 438GenPeptGraphics Next Match Previous Match

Alignment statistics for match #1

| Score | Expect | Method | Identities | Positives | Gaps |
|---|---|---|---|---|---|
| 31.9 bits(65) | 78 | Composition-based stats. | 42/162(26%) | 69/162(42%) | 23/162(14%) |

```
Query  5     AGKRELNAEKASTNSETNRGESEKKR-------NLGELSRTTSEDNEVFGEADANQNNGT  57
             A  + + AEKA   +E  R ++E +R       N  EL R   E N V   A+A + N
Sbjct  279   AEDKRVTAEKARAKAEEERVKAETRRENTYTDFNDSELERKNNEANRV--RAEAERVNAE  336

Query  58    SSQDTAVTDSKRTADPKNAWQDA--HPADPGS-RPHLIRLFSRD--APGRE--DNTF---  107
               ++ A   +S+R   +       +A     A+ G      ++R+ +     A G E   +NTF
Sbjct  337   NGRNEAYEESERVREEAFKVSEAARNEAEEGRVAAEVLRVEAEKLRAAGEENRNNTFTQS  396
```

```
Query  108   ----KDRPSESDELQTIQEDSAATSESLDVMASQKRPSQRHG  145
                 KD   E++  + + E      ES  V A + R ++  G
Sbjct  397   EEERKDIFEEAENTRKVAESKRDENESSRVEAEKLRVTEETG  438
```

Download GenPeptGraphics Next Previous Descriptions

hypothetical protein [Clostridium perfringens]

Sequence ID: WP_197550111.1Length: 1155Number of Matches: 1

Range 1: 388 to 446GenPeptGraphics Next Match Previous Match

Alignment statistics for match #1

| Score | Expect | Method | Identities | Positives | Gaps |
|---|---|---|---|---|---|
| 31.4 bits(64) | 109 | Composition-based stats. | 20/59(34%) | 28/59(47%) | 6/59(10%) |

```
Query  3     NHAGKRELNAEKASTNSETNRGESEKKRNLGELSRTTS------EDNEVFGEADANQNN  55
             N A +   + AE     SE  R +SEK R L E  R T+      E N  F + + N+N+
Sbjct  388   NDAEELRVTAESERVKSEKLRVDSEKARVLAEEERNTTFTQKEEERNNTFTQNETNRND  446
```

Download GenPeptGraphics Next Previous Descriptions

hypothetical protein [Clostridium perfringens]

Sequence ID: WP_198616560.1Length: 1207Number of Matches: 1

Range 1: 388 to 446GenPeptGraphics Next Match Previous Match

Alignment statistics for match #1

| Score | Expect | Method | Identities | Positives | Gaps |
|---|---|---|---|---|---|
| 31.4 bits(64) | 110 | Composition-based stats. | 20/59(34%) | 28/59(47%) | 6/59(10%) |

```
Query  3     NHAGKRELNAEKASTNSETNRGESEKKRNLGELSRTTS------EDNEVFGEADANQNN  55
             N A +   + AE     SE  R +SEK R L E  R T+      E N  F + + N+N+
Sbjct  388   NDAEELRVTAESERVKSEKLRVDSEKARVLAEEERNTTFTQKEEERNNTFTQNETNRND  446
```

---

Download GenPeptGraphics Next Previous Descriptions

hypothetical protein [Clostridium perfringens]

Sequence ID: MDU7110306.1Length: 1008Number of Matches: 1

Range 1: 346 to 404GenPeptGraphics Next Match Previous Match

Alignment statistics for match #1

| Score | Expect | Method | Identities | Positives | Gaps |
|---|---|---|---|---|---|
| 31.4 bits(64) | 114 | Composition-based stats. | 20/59(34%) | 28/59(47%) | 6/59(10%) |

```
Query  3     NHAGKRELNAEKASTNSETNRGESEKKRNLGELSRTTS------EDNEVFGEADANQNN  55
             N A +   + AE     SE  R +SEK R L E  R T+      E N  F + + N+N+
Sbjct  346   NDAEELRVTAESERVKSEKLRVDSEKARVLAEEERNTTFTQKEEERNNTFTQNETNRND  404
```

Download GenPeptGraphics
Sort by:

Next Previous Descriptions

hypothetical protein [Clostridium perfringens]
Sequence ID: WP_224404492.1Length: 1151Number of Matches: 2

- See 1 more title(s) See all Identical Proteins(IPG)

Related Information
Identical Proteins-Identical proteins to WP_224404492.1
Range 1: 384 to 453GenPeptGraphics Next Match Previous Match

Alignment statistics for match #1

| Score | Expect | Method | Identities | Positives | Gaps |
|---|---|---|---|---|---|
| 31.0 bits(63) | 122 | Composition-based stats. | 25/71(35%) | 32/71(45%) | 13/71(18%) |

```
Query  13   EKASTNSETNRGESEKKRNLGELSRTTSEDNEVFGEADANQNNGTSSQ------------  60
            E+A    SE   R  E+E+  R    EL  R   +E    V  GE  + N  N    T  S+
Sbjct  384  EEAFKVSEVARNEAEEGRVAAELLRVEAEKLRVAGEENRN-NTFTQSEEERKTTFEEAEN  442

Query  61   DTAVTDSKRTA  71
            D  V +SKR A
Sbjct  443  DRKVAESKRVA  453
```

Range 2: 167 to 221GenPeptGraphics Next Match Previous Match First Match

Alignment statistics for match #2

| Score | Expect | Method | Identities | Positives | Gaps |
|---|---|---|---|---|---|
| 30.6 bits(62) | 215 | Composition-based stats. | 22/61(36%) | 31/61(50%) | 6/61(9%) |

```
Query  8    RELNAEKASTNSETNRGESEKKRNLGELSRTTSEDNEVFGEADANQNNGTSSQDTAVTDS  67
            RE  N  EKA    SE   R    E++R    EL  R   +E+N     E+       N    S++T  VT+
Sbjct  167  REKN-EKARLQSEDIRITEEEERVANELERIKAENNRKVSES-----NRKVSENTRVTNE  220

Query  68   K  68
            +
Sbjct  221  R  221
```

Download GenPeptGraphics Next Previous Descriptions

hypothetical protein [Clostridium perfringens]
Sequence ID: WP_243448075.1Length: 939Number of Matches: 1
Range 1: 388 to 446GenPeptGraphics Next Match Previous Match

Alignment statistics for match #1

| Score | Expect | Method | Identities | Positives | Gaps |
|---|---|---|---|---|---|

31.0 bits(63) 140 Composition-based stats. 20/59(34%) 28/59(47%) 6/59(10%)

```
Query  3    NHAGKRELNAEKASTNSETNRGESEKKRNLGELSRTTS------EDNEVFGEADANQNN  55
            N A +   + AE     SE  R +SEK R L E  R T+      E N  F + + N+N+
Sbjct  388  NDAEELRVTAESERVKSEKLRVDSEKARVLAEEERNTTFTQKEEERNNTFTQNETNRND  446
```

Download GenPeptGraphics Next Previous Descriptions
hypothetical protein [Clostridium perfringens]
Sequence ID: EHK2400155.1Length: 1178Number of Matches: 1
Range 1: 389 to 447GenPeptGraphics Next Match Previous Match

Alignment statistics for match #1

| Score | Expect | Method | Identities | Positives | Gaps |
|---|---|---|---|---|---|
| 31.0 bits(63) | 141 | Composition-based stats. | 20/59(34%) | 28/59(47%) | 6/59(10%) |

```
Query  3    NHAGKRELNAEKASTNSETNRGESEKKRNLGELSRTTS------EDNEVFGEADANQNN  55
            N A +   + AE     SE  R +SEK R L E  R T+      E N  F + + N+N+
Sbjct  389  NDAEELRVTAESERVKSEKLRVDSEKARVLAEEERNTTFTQKEEERNNTFTQNETNRND  447
```

Download GenPeptGraphics
Sort by:

Next Previous Descriptions

hypothetical protein [Clostridium perfringens]
Sequence ID: HBI6980750.1Length: 1194Number of Matches: 2

- See 2 more title(s) See all Identical Proteins(IPG)

Related Information
Identical Proteins-Identical proteins to HBI6980750.1
Range 1: 192 to 235GenPeptGraphics Next Match Previous Match

Alignment statistics for match #1

| Score | Expect | Method | Identities | Positives | Gaps |
|---|---|---|---|---|---|
| 31.0 bits(63) | 144 | Composition-based stats. | 17/46(37%) | 23/46(50%) | 2/46(4%) |

```
Query  10   LNAEKASTNSETNRGESEKKRNLGELSRTTSEDNEVFGEADANQNN  55
            + AE A   +E NR  +E  R L E +R T E  V  EA+ N+
Sbjct  192  IKAEAARVEAENNRKVAESNRKLAENTRKTNEAARV--EAELNRQS  235
```

Range 2: 391 to 448GenPeptGraphics Next Match Previous Match First Match

Alignment statistics for match #2

| Score | Expect | Method | Identities | Positives | Gaps |
|---|---|---|---|---|---|
| 29.3 bits(59) | 521 | Composition-based stats. | 19/58(33%) | 27/58(46%) | 6/58(10%) |

```
Query  3    NHAGKRELNAEKASTNSETNRGESEKKRNLGELSRTTS------EDNEVFGEADANQN  54
```

```
            N A +  + AE     SE  R +SEK R L +  R T+      E N  F + + N+N
Sbjct  391  NDAEELRVTAESERVKSEKLRVDSEKARVLADEERNTTFTQKEEERNNTFTQNETNRN  448
```

---

Download GenPeptGraphics Next Previous Descriptions

hypothetical protein [Clostridium perfringens]

Sequence ID: MDK0576497.1Length: 1194Number of Matches: 1

- See 1 more title(s) See all Identical Proteins(IPG)

Related Information

Identical Proteins-Identical proteins to MDK0576497.1

Range 1: 384 to 497GenPeptGraphics Next Match Previous Match

Alignment statistics for match #1

| Score | Expect | Method | Identities | Positives | Gaps |
|---|---|---|---|---|---|
| 31.0 bits(63) | 151 | Composition-based stats. | 34/129(26%) | 55/129(42%) | 17/129(13%) |

```
Query  13   EKASTNSETNRGESEKKRNLGELSRTTSEDNEVFGEADANQNNGTSSQDT--AVTDSKRT  70
            E+A   SE  R E+E+ R   E  R  SE   V   AD  +NN  +  +T    T ++
Sbjct  384  EEAFKVSEATRNEAEEGRVEAESERVKSEKARVL--ADEERNNTFTQNETNRNTTFTQNE  441

Query  71   ADPKNAWQDAHPADPGSRPHLIRLFSRDAPGREDNTFKDRPSESDELQTIQEDSAATSES  130
             + K   ++A  A             + A  +     K R  E+++L+ + E     +ES
Sbjct  442  EERKGIFEEAENA------------RKVAETKRVEAEKTR-VEAEKLRVVAETGRVEAES  488

Query  131  LDVMASQKR  139
            L V A ++R
Sbjct  489  LRVTAEEER  497
```

Download GenPeptGraphics Next Previous Descriptions

BppU family phage baseplate upper protein [Clostridium botulinum]

Sequence ID: WP_222657265.1Length: 454Number of Matches: 1

- See 1 more title(s) See all Identical Proteins(IPG)

Related Information

Identical Proteins-Identical proteins to WP_222657265.1

Range 1: 157 to 217GenPeptGraphics Next Match Previous Match

Alignment statistics for match #1

| Score | Expect | Method | Identities | Positives | Gaps |
|---|---|---|---|---|---|
| 30.6 bits(62) | 154 | Compositional matrix adjust. | 23/61(38%) | 33/61(54%) | 3/61(4%) |

```
Query  18   NSETNRGESEKKRNLGELSRTTSEDNEVFGEA--DANQNNGTSSQDTAV-TDSKRTADPK  74
            N E  R ESE+ R L E+ R  SE+  V  EA  +AN+N     + T +  +SKR  + +
```

```
Sbjct  157   NVENARVESEENRKLEEIKRVDSENIRVENEAAREANENQRVENEKTRLENESKRAENEE  216

Query  75    N  75
             N
Sbjct  217   N  217
```

Download GenPeptGraphics Next Previous Descriptions
hypothetical protein [Clostridium perfringens]
Sequence ID: WP_207658360.1Length: 1066Number of Matches: 1

- See 2 more title(s) See all Identical Proteins(IPG)

Related Information
Identical Proteins-Identical proteins to WP_207658360.1
Range 1: 388 to 446GenPeptGraphics Next Match Previous Match

Alignment statistics for match #1

| Score | Expect | Method | Identities | Positives | Gaps |
|---|---|---|---|---|---|
| 30.6 bits(62) | 161 | Composition-based stats. | 20/59(34%) | 28/59(47%) | 6/59(10%) |

```
Query  3     NHAGKRELNAEKASTNSETNRGESEKKRNLGELSRTTS------EDNEVFGEADANQNN  55
             N A +   + AE      SE  R +SEK R L E  R T+       E N  F + + N+N+
Sbjct  388   NDAEELRVTAESERVKSEKLRVDSEKARVLAEEERNTTFTQKEEERNNTFTQNETNRND  446
```

Download GenPeptGraphics Next Previous Descriptions
hypothetical protein [Clostridium perfringens]
Sequence ID: WP_224380875.1Length: 1019Number of Matches: 1

- See 1 more title(s) See all Identical Proteins(IPG)

Related Information
Identical Proteins-Identical proteins to WP_224380875.1
Range 1: 391 to 449GenPeptGraphics Next Match Previous Match

Alignment statistics for match #1

| Score | Expect | Method | Identities | Positives | Gaps |
|---|---|---|---|---|---|
| 30.6 bits(62) | 162 | Composition-based stats. | 20/59(34%) | 28/59(47%) | 6/59(10%) |

```
Query  3     NHAGKRELNAEKASTNSETNRGESEKKRNLGELSRTTS------EDNEVFGEADANQNN  55
             N A +   + AE      SE  R +SEK R L E  R T+       E N  F + + N+N+
Sbjct  391   NDAEELRVTAESERVKSEKLRVDSEKARVLAEEERNTTFTQKEEERNNTFTQNETNRND  449
```

Download GenPeptGraphics Next Previous Descriptions
BppU family phage baseplate upper protein [Clostridium novyi]

Sequence ID: WP_011722235.1Length: 614Number of Matches: 1

- See 1 more title(s) See all Identical Proteins(IPG)

Related Information

AlphaFold Structure-3D structure displays

Identical Proteins-Identical proteins to WP_011722235.1

Range 1: 275 to 351GenPeptGraphics Next Match Previous Match

Alignment statistics for match #1

| Score | Expect | Method | Identities | Positives | Gaps |
|---|---|---|---|---|---|
| 30.6 bits(62) | 198 | Compositional matrix adjust. | 24/80(30%) | 36/80(45%) | 5/80(6%) |

```
Query  13   EKASTNSETNRGESEKKRNLGELSRTTSEDNEVFGEA--DANQNNGTSSQDTAVTDSKRT  70
            EK  T +E  R E EK R   E+ R  SE+  V  E   + N+ N   ++ T V +  +
Sbjct  275  EKLRTEAEEKRVEDEKLRVEKEIERANSENTRVTAEQKRETNETNRVEAEKTRVEEWDKI  334

Query  71   ADPKNAWQDAHPADPGSRPH  90
               KN  +D  P D  +  +
Sbjct  335  ---KNTFKDNAPGDMKAVVY  351
```

Download GenPeptGraphics Next Previous Descriptions

BppU family phage baseplate upper protein [Clostridium haemolyticum]

Sequence ID: WP_053071462.1Length: 614Number of Matches: 1

Range 1: 275 to 351GenPeptGraphics Next Match Previous Match

Alignment statistics for match #1

| Score | Expect | Method | Identities | Positives | Gaps |
|---|---|---|---|---|---|
| 30.1 bits(61) | 214 | Compositional matrix adjust. | 24/80(30%) | 36/80(45%) | 5/80(6%) |

```
Query  13   EKASTNSETNRGESEKKRNLGELSRTTSEDNEVFGEA--DANQNNGTSSQDTAVTDSKRT  70
            EK  T +E  R E EK R   E+ R  SE+  V  E   + N+ N   ++ T V +  +
Sbjct  275  EKLRTEAEEKRVEDEKLRVEKEIERANSENTRVTAEQKRETNETNRVEAEKTRVEEWDKI  334

Query  71   ADPKNAWQDAHPADPGSRPH  90
               KN  +D  P D  +  +
Sbjct  335  ---KNTFKDNAPGDMKAVVY  351
```

Download GenPeptGraphics Next Previous Descriptions

BppU family phage baseplate upper protein [Clostridium botulinum]

Sequence ID: WP_003395953.1Length: 454Number of Matches: 1

- See 5 more title(s) See all Identical Proteins(IPG)

Related Information

AlphaFold Structure-3D structure displays
Identical Proteins-Identical proteins to WP_003395953.1
Range 1: 157 to 217GenPeptGraphics Next Match Previous Match

Alignment statistics for match #1

| Score | Expect | Method | Identities | Positives | Gaps |
|---|---|---|---|---|---|
| 30.1 bits(61) | 217 | Compositional matrix adjust. | 23/61(38%) | 33/61(54%) | 3/61(4%) |

```
Query  18   NSETNRGESEKKRNLGELSRTTSEDNEVFGEA--DANQNNGTSSQDTAV-TDSKRTADPK  74
            N E  R ESE+ R L E+ R  SE+  V  EA  +AN+N      + T +  +SKR  + +
Sbjct  157  NVENARVESEENRKLEEIKRVDSENIRVENEAGREANENKRVENEKTRLENESKRAENEE  216

Query  75   N  75
            N
Sbjct  217  N  217
```

Download GenPeptGraphics Next Previous Descriptions
hypothetical protein [Clostridium perfringens]
Sequence ID: MDK0720529.1Length: 1176Number of Matches: 1
Range 1: 384 to 497GenPeptGraphics Next Match Previous Match

Alignment statistics for match #1

| Score | Expect | Method | Identities | Positives | Gaps |
|---|---|---|---|---|---|
| 30.1 bits(61) | 217 | Composition-based stats. | 34/129(26%) | 55/129(42%) | 17/129(13%) |

```
Query  13   EKASTNSETNRGESEKKRNLGELSRTTSEDNEVFGEADANQNNGTSSQDT--AVTDSKRT  70
            E+A   SE  R E+E+ R   E  R  SE   V   AD  +NN  +  +T     T ++
Sbjct  384  EEAFKVSEAARNEAEEGRVEAESERVKSEKARVL--ADEERNNTFTQNETNRNTTFTQNE  441

Query  71   ADPKNAWQDAHPADPGSRPHLIRLFSRDAPGREDNTFKDRPSESDELQTIQEDSAATSES  130
             + K    ++A  A              + A  +      K R  E+++L+ + E      +ES
Sbjct  442  EERKGIFEEAENA------------RKVAETKRVEAEKTR-VEAEKLRVVAETGRVETES  488

Query  131  LDVMASQKR  139
             L V A ++R
Sbjct  489  LRVTAEEER  497
```

Download GenPeptGraphics
Sort by:

Next Previous Descriptions

hypothetical protein [Clostridium sp.]
Sequence ID: WP_316326076.1Length: 947Number of Matches: 2

- See 2 more title(s) See all Identical Proteins(IPG)

Related Information

Identical Proteins-Identical proteins to WP_316326076.1

Range 1: 388 to 445GenPeptGraphics Next Match Previous Match

Alignment statistics for match #1

| Score | Expect | Method | Identities | Positives | Gaps |
|---|---|---|---|---|---|
| 30.1 bits(61) | 252 | Composition-based stats. | 20/58(34%) | 27/58(46%) | 6/58(10%) |

```
Query  3    NHAGKRELNAEKASTNSETNRGESEKKRNLGELSRTTS------EDNEVFGEADANQN  54
            N A +  + AE     SE  R +SEK R L E  R T+      E N  F + + N+N
Sbjct  388  NDAEELRVTAESERVKSEKLRVDSEKARVLAEEERNTTFTQKEEERNNTFTQNEINRN  445
```

Range 2: 189 to 232GenPeptGraphics Next Match Previous Match First Match

Alignment statistics for match #2

| Score | Expect | Method | Identities | Positives | Gaps |
|---|---|---|---|---|---|
| 28.4 bits(57) | 821 | Composition-based stats. | 16/46(35%) | 22/46(47%) | 2/46(4%) |

```
Query  10   LNAEKASTNSETNRGESEKKRNLGELSRTTSEDNEVFGEADANQNN  55
            + AE     +E NR  +E  R L E +R T E    V  EA+ N+
Sbjct  189  IKAEAERKEAENNRKVAESNRKLAENTRKTNEAARV--EAELNRQS  232
```

Download GenPeptGraphics Next Previous Descriptions

hypothetical protein [Clostridium perfringens]

Sequence ID: MDK0630032.1Length: 1184Number of Matches: 1

Range 1: 388 to 446GenPeptGraphics Next Match Previous Match

Alignment statistics for match #1

| Score | Expect | Method | Identities | Positives | Gaps |
|---|---|---|---|---|---|
| 29.7 bits(60) | 324 | Composition-based stats. | 19/59(32%) | 28/59(47%) | 6/59(10%) |

```
Query  3    NHAGKRELNAEKASTNSETNRGESEKKRNLGELSRTTS------EDNEVFGEADANQNN  55
            N A +  + AE     +E  R +SEK R L E  R T+      E N  F + + N+N+
Sbjct  388  NDAEELRVTAESERVKAEKLRVDSEKARVLAEEERNTTFTQKEEERNNTFTQNETNRND  446
```

Download GenPeptGraphics Next Previous Descriptions

DNA replication protein dnaD [uncultured Clostridium sp.]

Sequence ID: SCI51868.1Length: 413Number of Matches: 1

Related Information

AlphaFold Structure-3D structure displays

Identical Proteins-Identical proteins to WP_262585152.1

Range 1: 100 to 197GenPeptGraphics Next Match Previous Match

Alignment statistics for match #1

| Score | Expect | Method | Identities | Positives | Gaps |
|---|---|---|---|---|---|

| Score | Expect | Method | Identities | Positives | Gaps |
|---|---|---|---|---|---|
| 29.3 bits(59) | 426 | Compositional matrix adjust. | 33/101(33%) | 47/101(46%) | 20/101(19%) |

```
Query  6      GKRELNAEKASTNSETNRGESEKKRNLGELSRTT-SEDNEVFGEADANQNNGT-------  57
              GKR+ +A +A   +E   G     R++G L+ TT S D E FG+A  N   G
Sbjct  100    GKRQ-DAGQADFSTEQTTGHI--SRDVGRLADTTRSSDAERFGDAARNSEQGADATLPGA  156

Query  58     ---SSQDTAVTDSKRTADPKN------AWQDAHPADPGSRP  89
                  + +TA   S RTA P+       + Q   PADP ++P
Sbjct  157    AARDTSNTAAAPSGRTAAPQIRSVTMLSHQSLEPADPYAKP  197
```

Download GenPeptGraphics Next Previous Descriptions

hypothetical protein [Clostridium perfringens]

Sequence ID: MDM0678296.1Length: 1156Number of Matches: 1

Range 1: 388 to 464GenPeptGraphics Next Match Previous Match

Alignment statistics for match #1

| Score | Expect | Method | Identities | Positives | Gaps |
|---|---|---|---|---|---|
| 29.3 bits(59) | 442 | Composition-based stats. | 24/77(31%) | 33/77(42%) | 8/77(10%) |

```
Query  3      NHAGKRELNAEKASTNSETNRGESEKKRNLGELSRTTS------EDNEVFGEADANQNNG  56
              N A +  + AE     SE  R +SEK R L E  R T+      E N  F + +  +
Sbjct  388    NDAEELRVTAESERVKSEKLRVDSEKARVLAEEERNTTFTQKEEERNNTFTQNEEERQGV  447

Query  57     TSSQDTA--VTDSKRTA  71
                  + A  V +SKR A
Sbjct  448    FEEAENARKVAESKRVA  464
```

Download GenPeptGraphics Next Previous Descriptions

hypothetical protein [Clostridium perfringens]

Sequence ID: WP_252796140.1Length: 864Number of Matches: 1

- See 4 more title(s) See all Identical Proteins(IPG)

Related Information

Identical Proteins-Identical proteins to WP_252796140.1

Range 1: 266 to 295GenPeptGraphics Next Match Previous Match

Alignment statistics for match #1

| Score | Expect | Method | Identities | Positives | Gaps |
|---|---|---|---|---|---|
| 29.3 bits(59) | 452 | Compositional matrix adjust. | 15/30(50%) | 19/30(63%) | 0/30(0%) |

```
Query  20     ETNRGESEKKRNLGELSRTTSEDNEVFGEA  49
              E NR E+EK R L E+ R +SE+  V  EA
Sbjct  266    ELNRKEAEKIRVLAEVERKSSEESRVAAEA  295
```

Download GenPeptGraphics Next Previous Descriptions
hypothetical protein [Clostridium perfringens]
Sequence ID: WP_228581235.1Length: 864Number of Matches: 1

- See 4 more title(s) See all Identical Proteins(IPG)

Related Information
Identical Proteins-Identical proteins to WP_228581235.1
Range 1: 266 to 295GenPeptGraphics Next Match Previous Match

Alignment statistics for match #1

| Score | Expect | Method | Identities | Positives | Gaps |
|---|---|---|---|---|---|
| 29.3 bits(59) | 466 | Compositional matrix adjust. | 15/30(50%) | 19/30(63%) | 0/30(0%) |

```
Query  20   ETNRGESEKKRNLGELSRTTSEDNEVFGEA  49
            E NR E+EK R L E+ R +SE+  V  EA
Sbjct  266  ELNRKEAEKIRVLAEVERKSSEESRVAAEA  295
```

Download GenPeptGraphics Next Previous Descriptions
hypothetical protein [Clostridium perfringens]
Sequence ID: HBI6966192.1Length: 1048Number of Matches: 1

- See 4 more title(s) See all Identical Proteins(IPG)

Related Information
Identical Proteins-Identical proteins to HBI6966192.1
Range 1: 384 to 450GenPeptGraphics Next Match Previous Match

Alignment statistics for match #1

| Score | Expect | Method | Identities | Positives | Gaps |
|---|---|---|---|---|---|
| 29.3 bits(59) | 480 | Composition-based stats. | 20/67(30%) | 32/67(47%) | 5/67(7%) |

```
Query  13   EKASTNSETNRGESEKKRNLGELSRTTSEDNEVFGEADANQNNGTSSQDTAVT-----DS  67
            E+A   SE  R E+E+ R   EL R  +E   V GE + N      S ++   T      ++
Sbjct  384  EEAFKVSEAARNEAEEGRVAAELLRAEAEKLRVTGEENRNATFTQSEEERKATFEEAENA  443

Query  68   KRTADPK  74
            ++ A+ K
Sbjct  444  RKVAESK  450
```

Download GenPeptGraphics Next Previous Descriptions
hypothetical protein [Clostridium perfringens]
Sequence ID: HBJ6023808.1Length: 979Number of Matches: 1

- See 1 more title(s) See all Identical Proteins(IPG)

Related Information

Identical Proteins-Identical proteins to HBJ6023808.1

Range 1: 277 to 346GenPeptGraphics Next Match Previous Match

Alignment statistics for match #1

| Score | Expect | Method | Identities | Positives | Gaps |
|---|---|---|---|---|---|
| 29.3 bits(59) | 501 | Composition-based stats. | 24/71(34%) | 32/71(45%) | 13/71(18%) |

```
Query  13   EKASTNSETNRGESEKKRNLGELSRTTSEDNEVFGEADANQNNGTSSQDT----------  62
            E+A   SE  R E+E+ R   EL R  +E   V GE + N N  T S++
Sbjct  277  EEAFKVSEAARNEAEEGRVAAELLRAEAEKLRVAGEENRN-NTFTQSEEERKCVFEEAEN  335

Query  63   --AVTDSKRTA  71
               V +SKR A
Sbjct  336  ARKVAESKRVA  346
```

Download GenPeptGraphics

Sort by:

Next Previous Descriptions

hypothetical protein [Clostridium perfringens]

Sequence ID: MDM0462880.1Length: 1053Number of Matches: 2

Range 1: 384 to 453GenPeptGraphics Next Match Previous Match

Alignment statistics for match #1

| Score | Expect | Method | Identities | Positives | Gaps |
|---|---|---|---|---|---|
| 28.8 bits(58) | 548 | Composition-based stats. | 22/70(31%) | 30/70(42%) | 11/70(15%) |

```
Query  13   EKASTNSETNRGESEKKRNLGELSRTTSEDNEVFGEADANQNNGTSSQD-----------  61
            E+A   SE  R E+E+ R   EL R  +E   V GE + N       S ++
Sbjct  384  EEAFKVSEASRNEAEEGRVAAELLRVEAEKLRVAGEENRNTTFTQSEEERQGVFEEAENA  443

Query  62   TAVTDSKRTA  71
              V +SKR A
Sbjct  444  RKVAESKRVA  453
```

Range 2: 447 to 477GenPeptGraphics Next Match Previous Match First Match

Alignment statistics for match #2

| Score | Expect | Method | Identities | Positives | Gaps |
|---|---|---|---|---|---|
| 28.4 bits(57) | 951 | Composition-based stats. | 13/31(42%) | 17/31(54%) | 0/31(0%) |

```
Query  12   AEKASTNSETNRGESEKKRNLGELSRTTSED  42
            AE     +ET RGE+EK R   E  R  +E+
Sbjct  447  AESKRVAAETGRGEAEKLRVTAETERVEAEN  477
```

Download GenPeptGraphics
Sort by:
Next Previous Descriptions

hypothetical protein [Clostridium perfringens]
Sequence ID: MDM0722965.1Length: 1179Number of Matches: 2

- See 1 more title(s) See all Identical Proteins(IPG)

Related Information
Identical Proteins-Identical proteins to MDM0722965.1
Range 1: 388 to 464GenPeptGraphics Next Match Previous Match

Alignment statistics for match #1

| Score | Expect | Method | Identities | Positives | Gaps |
|---|---|---|---|---|---|
| 28.8 bits(58) | 551 | Composition-based stats. | 24/77(31%) | 33/77(42%) | 8/77(10%) |

```
Query  3    NHAGKRELNAEKASTNSETNRGESEKKRNLGELSRTTS------EDNEVFGEADANQNNG  56
            N A +   + AE     SE  R +SEK R L E  R T+       E N  F + +  +
Sbjct  388  NDAEELRVTAESERVKSEKLRVDSEKARVLAEEERNTTFTQKEEERNNTFTQNEEERQGI  447

Query  57   TSSQDTA--VTDSKRTA  71
                + A  V +SKR A
Sbjct  448  FEEAENARKVAESKRVA  464
```

Range 2: 189 to 232GenPeptGraphics Next Match Previous Match First Match

Alignment statistics for match #2

| Score | Expect | Method | Identities | Positives | Gaps |
|---|---|---|---|---|---|
| 28.8 bits(58) | 648 | Composition-based stats. | 16/46(35%) | 22/46(47%) | 2/46(4%) |

```
Query  10   LNAEKASTNSETNRGESEKKRNLGELSRTTSEDNEVFGEADANQNN  55
            + AE     +E NR  +E  R L E +R T E   V  EA+ N+
Sbjct  189  IKAEAERKEAENNRKVAESNRKLAENTRKTNEAARV--EAELNRQS  232
```

Download GenPeptGraphics Next Previous Descriptions
PTS transporter subunit EIIC [Clostridium sp. Marseille-Q2269]
Sequence ID: WP_251861130.1Length: 527Number of Matches: 1
Range 1: 404 to 446GenPeptGraphics Next Match Previous Match

Alignment statistics for match #1

| Score | Expect | Method | Identities | Positives | Gaps |
|---|---|---|---|---|---|
| 28.8 bits(58) | 590 | Compositional matrix adjust. | 17/43(40%) | 24/43(55%) | 2/43(4%) |

```
Query  90   HLIRLFSRDAPGREDNTFK--DRPSESDELQTIQEDSAATSES  130
             LI+ FS   PGREDN  K  ++     + L    E++AA+ ES
Sbjct  404  FLIKKFSLKTPGREDNEVKLVNKKEFKESLDKSSEEAAASKES  446
```

Download GenPeptGraphics Next Previous Descriptions
DnaD domain protein [uncultured Clostridium sp.]
Sequence ID: SCH24497.1Length: 413Number of Matches: 1
Related Information
AlphaFold Structure-3D structure displays
Range 1: 100 to 197GenPeptGraphics Next Match Previous Match

Alignment statistics for match #1

| Score | Expect | Method | Identities | Positives | Gaps |
| --- | --- | --- | --- | --- | --- |
| 28.8 bits(58) | 607 | Compositional matrix adjust. | 33/101(33%) | 46/101(45%) | 20/101(19%) |

```
Query  6    GKRELNAEKASTNSETNRGESEKKRNLGELSRTT-SEDNEVFGEADANQNNGT-------  57
            GKR+ +A +A   +E   G     R++G L+ TT S D E FG+A  N   G
Sbjct  100  GKRQ-DAGQADFSTEQTTGHI--SRDVGRLADTTRSSDAERFGDAARNSEQGADATLPGA  156

Query  58   ---SSQDTAVTDSKRTADPKN------AWQDAHPADPGSRP  89
               + +TA   S RTA P+       + Q   PADP  +P
Sbjct  157  AAYDTSNTAAAPSGRTAAPQIRSITMLSHQSLEPADPYVKP  197
```

---

Download GenPeptGraphics Next Previous Descriptions
hypothetical protein [Clostridium perfringens]
Sequence ID: EHK2362478.1Length: 1017Number of Matches: 1
Range 1: 388 to 445GenPeptGraphics Next Match Previous Match

Alignment statistics for match #1

| Score | Expect | Method | Identities | Positives | Gaps |
| --- | --- | --- | --- | --- | --- |
| 28.8 bits(58) | 613 | Composition-based stats. | 19/58(33%) | 27/58(46%) | 6/58(10%) |

```
Query  3    NHAGKRELNAEKASTNSETNRGESEKKRNLGELSRTTS------EDNEVFGEADANQN  54
            N A +  + AE     +E  R +SEK R L E  R T+       E N  F + + N+N
Sbjct  388  NDAEELRVTAESERVKAEKLRVDSEKARVLAEEERNTTFTQKEEERNNTFTQNETNRN  445
```

Download GenPeptGraphics Next Previous Descriptions
phage baseplate upper protein [Clostridium botulinum]
Sequence ID: WP_061323077.1Length: 454Number of Matches: 1

- See 1 more title(s) See all Identical Proteins(IPG)

Related Information
Identical Proteins-Identical proteins to WP_061323077.1
Range 1: 157 to 203GenPeptGraphics Next Match Previous Match

Alignment statistics for match #1

---

| Score | Expect | Method | Identities | Positives | Gaps |
|---|---|---|---|---|---|
| 28.8 bits(58) | 663 | Compositional matrix adjust. | 19/47(40%) | 25/47(53%) | 2/47(4%) |

```
Query  18   NSETNRGESEKKRNLGELSRTTSEDNEVFGEA--DANQNNGTSSQDT  62
            N E  R ESE+ R L E+ R  SE+  V  EA  +AN+N     + T
Sbjct  157  NVENARVESEENRKLEEIKRVDSENIRVENEAGREANENQRVENEKT  203
```

Download GenPeptGraphics Next Previous Descriptions
hypothetical protein [Clostridium sp. AF34-10BH]
Sequence ID: WP_207664171.1Length: 190Number of Matches: 1
Range 1: 32 to 87GenPeptGraphics Next Match Previous Match

Alignment statistics for match #1

| Score | Expect | Method | Identities | Positives | Gaps |
|---|---|---|---|---|---|
| 28.4 bits(57) | 672 | Compositional matrix adjust. | 20/59(34%) | 31/59(52%) | 3/59(5%) |

```
Query  12   AEKASTNSETNRGESEKKRNLGELSRTTSEDNEVFGEADANQNNGTSSQDTAVTDSKRT  70
            AEK     +E+NR    EK  +  + E ++ T   + E+  E       NGT S+   AV   S++T
Sbjct  32   AEKRDAHAESNRNIEEKAKTV-EGAKVT--ETEIPNEVQITNPNGTFSHKLAVVHSQKT  87
```

Download GenPeptGraphics Next Previous Descriptions
phage baseplate upper protein [Clostridium botulinum]
Sequence ID: WP_075143212.1Length: 454Number of Matches: 1

- See 1 more title(s) See all Identical Proteins(IPG)

Related Information
Identical Proteins-Identical proteins to WP_075143212.1
Range 1: 157 to 203GenPeptGraphics Next Match Previous Match

Alignment statistics for match #1

| Score | Expect | Method | Identities | Positives | Gaps |
|---|---|---|---|---|---|
| 28.4 bits(57) | 709 | Compositional matrix adjust. | 19/47(40%) | 25/47(53%) | 2/47(4%) |

```
Query  18   NSETNRGESEKKRNLGELSRTTSEDNEVFGEA--DANQNNGTSSQDT  62
            N E  R ESE+ R L E+ R  SE+  V  EA  +AN+N     + T
Sbjct  157  NVENARVESEENRKLEEIKRVDSENIRVENEAGREANENQRVENEKT  203
```

Download GenPeptGraphics Next Previous Descriptions
BppU family phage baseplate upper protein [Clostridium botulinum]
Sequence ID: WP_021106728.1Length: 454Number of Matches: 1

- See 5 more title(s) See all Identical Proteins(IPG)

Related Information

---

[Identical Proteins]-Identical proteins to WP_021106728.1

Range 1: 157 to 203[GenPeptGraphics] Next Match Previous Match

Alignment statistics for match #1

| Score | Expect | Method | Identities | Positives | Gaps |
|---|---|---|---|---|---|
| 28.4 bits(57) | 709 | Compositional matrix adjust. | 19/47(40%) | 25/47(53%) | 2/47(4%) |

```
Query  18   NSETNRGESEKKRNLGELSRTTSEDNEVFGEA--DANQNNGTSSQDT  62
            N E  R ESE+ R L E+ R  SE+  V  EA  +AN+N     + T
Sbjct  157  NVENARVESEENRKLEEIKRVDSENIRVENEAGREANENQRVENEKT  203
```

---

[Download] [GenPeptGraphics] Next Previous [Descriptions]

BppU family phage baseplate upper protein [Clostridium botulinum]

Sequence ID: [EJE7236807.1]Length: 621Number of Matches: 1

Range 1: 194 to 228[GenPeptGraphics] Next Match Previous Match

Alignment statistics for match #1

| Score | Expect | Method | Identities | Positives | Gaps |
|---|---|---|---|---|---|
| 28.4 bits(57) | 753 | Compositional matrix adjust. | 15/35(43%) | 20/35(57%) | 0/35(0%) |

```
Query  19   SETNRGESEKKRNLGELSRTTSEDNEVFGEADANQ  53
             ETNR E EK R + E+ R    E+N V  E++  Q
Sbjct  194  NETNRIENEKNRAVDEVIRGKNEENRVTKESERTQ  228
```

[Download] [GenPeptGraphics] Next Previous [Descriptions]

hypothetical protein [Clostridium perfringens]

Sequence ID: [EJT5930513.1]Length: 1077Number of Matches: 1

- [See 2 more title(s)] [See all Identical Proteins(IPG)]

Related Information

[Identical Proteins]-Identical proteins to EJT5930513.1

Range 1: 384 to 451[GenPeptGraphics] Next Match Previous Match

Alignment statistics for match #1

| Score | Expect | Method | Identities | Positives | Gaps |
|---|---|---|---|---|---|
| 28.4 bits(57) | 773 | Composition-based stats. | 22/68(32%) | 30/68(44%) | 11/68(16%) |

```
Query  13   EKASTNSETNRGESEKKRNLGELSRTTSEDNEVFGEADANQNNGTSSQD-----------  61
            E+A   SE  R E+E+ R   EL R  +E   V GE + N      S ++
Sbjct  384  EEAFKVSEAARNEAEEGRVAAELLRVEAEKLRVAGEENRNTTFTQSEEERQGVFEEAENA  443

Query  62   TAVTDSKR  69
              VT+SKR
Sbjct  444  RKVTESKR  451
```

Download GenPeptGraphics Next Previous Descriptions

hypothetical protein [Clostridium perfringens]

Sequence ID: MDM0738572.1Length: 961Number of Matches: 1

Range 1: 384 to 451GenPeptGraphics Next Match Previous Match

Alignment statistics for match #1

| Score | Expect | Method | Identities | Positives | Gaps |
|---|---|---|---|---|---|
| 28.4 bits(57) | 845 | Composition-based stats. | 22/68(32%) | 30/68(44%) | 11/68(16%) |

```
Query  13   EKASTNSETNRGESEKKRNLGELSRTTSEDNEVFGEADANQNNGTSSQD-----------  61
            E+A   SE  R E+E+ R   EL R  +E   V GE + N     S ++
Sbjct  384  EEAFKVSEAARNEAEEGRVAAELLRVEAEKLRVAGEENRNTTFTQSEEERQGVFEEAENA  443

Query  62   TAVTDSKR  69
              VT+SKR
Sbjct  444  RKVTESKR  451
```

Download GenPeptGraphics Next Previous Descriptions

BppU family phage baseplate upper protein, partial [Clostridium botulinum]

Sequence ID: WP_154698359.1Length: 280Number of Matches: 1

Range 1: 89 to 135GenPeptGraphics Next Match Previous Match

Alignment statistics for match #1

| Score | Expect | Method | Identities | Positives | Gaps |
|---|---|---|---|---|---|
| 28.0 bits(56) | 877 | Compositional matrix adjust. | 19/47(40%) | 25/47(53%) | 2/47(4%) |

```
Query  18   NSETNRGESEKKRNLGELSRTTSEDNEVFGEA--DANQNNGTSSQDT  62
            N E  R ESE+ R L E+ R  SE+  V  EA  +AN+N     + T
Sbjct  89   NVENARVESEENRKLEEIKRVDSENIRVENEAGREANENQRVENEKT  135
```

Download GenPeptGraphics Next Previous Descriptions

BppU family phage baseplate upper protein [Clostridium botulinum]

Sequence ID: WP_003362081.1Length: 454Number of Matches: 1

- See 5 more title(s) See all Identical Proteins(IPG)

Related Information

AlphaFold Structure-3D structure displays

Identical Proteins-Identical proteins to WP_003362081.1

Range 1: 157 to 203GenPeptGraphics Next Match Previous Match

Alignment statistics for match #1

| Score | Expect | Method | Identities | Positives | Gaps |
|---|---|---|---|---|---|
| 28.4 bits(57) | 884 | Compositional matrix adjust. | 19/47(40%) | 25/47(53%) | 2/47(4%) |

```
Query  18    NSETNRGESEKKRNLGELSRTTSEDNEVFGEA--DANQNNGTSSQDT  62
             N E  R ESE+ R L E+ R  SE+  V  EA  +AN+N      + T
Sbjct  157   NVENARVESEENRKLEEIKRVDSENIRVENEAGREANENQRVENEKT  203
```

---

Download GenPeptGraphics Next Previous Descriptions

BppU family phage baseplate upper protein [Clostridium botulinum]

Sequence ID: WP_024933168.1Length: 454Number of Matches: 1

- See 1 more title(s) See all Identical Proteins(IPG)

Related Information

Identical Proteins-Identical proteins to WP_024933168.1

Range 1: 157 to 203GenPeptGraphics Next Match Previous Match

Alignment statistics for match #1

| Score | Expect | Method | Identities | Positives | Gaps |
|---|---|---|---|---|---|
| 28.4 bits(57) | 892 | Compositional matrix adjust. | 19/47(40%) | 25/47(53%) | 2/47(4%) |

```
Query  18    NSETNRGESEKKRNLGELSRTTSEDNEVFGEA--DANQNNGTSSQDT  62
             N E  R ESE+ R L E+ R  SE+  V  EA  +AN+N      + T
Sbjct  157   NVENARVESEENRKLEEIKRVDSENIRVENEAGREANENQRVENEKT  203
```

Download GenPeptGraphics Next Previous Descriptions

DUF2479 domain-containing protein [Clostridium botulinum]

Sequence ID: NFE32077.1Length: 454Number of Matches: 1

Range 1: 157 to 203GenPeptGraphics Next Match Previous Match

Alignment statistics for match #1

| Score | Expect | Method | Identities | Positives | Gaps |
|---|---|---|---|---|---|
| 28.4 bits(57) | 909 | Compositional matrix adjust. | 19/47(40%) | 25/47(53%) | 2/47(4%) |

```
Query  18    NSETNRGESEKKRNLGELSRTTSEDNEVFGEA--DANQNNGTSSQDT  62
             N E  R ESE+ R L E+ R  SE+  V  EA  +AN+N      + T
Sbjct  157   NVENARVESEENRKLEEIKRVDSENIRVENEAGREANENQRVENEKT  203
```

Download GenPeptGraphics

Sort by:

---

Next Previous Descriptions

hypothetical protein [Clostridium perfringens]

Sequence ID: WP_221373623.1Length: 1167Number of Matches: 2

Range 1: 447 to 477GenPeptGraphics Next Match Previous Match

Alignment statistics for match #1

| Score | Expect | Method | Identities | Positives | Gaps |
|---|---|---|---|---|---|

| Score | Expect | Method | Identities | Positives | Gaps |
|---|---|---|---|---|---|
| 28.4 bits(57) | 920 | Composition-based stats. | 13/31(42%) | 17/31(54%) | 0/31(0%) |

```
Query  12   AEKASTNSETNRGESEKKRNLGELSRTTSED  42
            AE      +ET RGE+EK R    E   R   +E+
Sbjct  447  AESKRVAAETGRGEAEKLRVTAETERVEAEN  477
```

Range 2: 384 to 453GenPeptGraphics Next Match Previous Match First Match

Alignment statistics for match #2

| Score | Expect | Method | Identities | Positives | Gaps |
|---|---|---|---|---|---|
| 28.0 bits(56) | 993 | Composition-based stats. | 22/70(31%) | 30/70(42%) | 11/70(15%) |

```
Query  13   EKASTNSETNRGESEKKRNLGELSRTTSEDNEVFGEADANQNNGTSSQD-----------  61
            E+A    SE   R E+E+ R    EL R   +E    V GE + N       S ++
Sbjct  384  EEAFKVSEAARNEAEEGRVAAELLRVEAEKLRVAGEENRNTTFTQSEEERQGVFEEAENA  443

Query  62   TAVTDSKRTA  71
              V +SKR A
Sbjct  444  RKVAESKRVA  453
```

Download GenPeptGraphics Next Previous Descriptions

hypothetical protein [Clostridium perfringens]

Sequence ID: WP_314162297.1Length: 1198Number of Matches: 1

- See 2 more title(s) See all Identical Proteins(IPG)

Related Information

Identical Proteins-Identical proteins to WP_314162297.1

Range 1: 447 to 477GenPeptGraphics Next Match Previous Match

Alignment statistics for match #1

| Score | Expect | Method | Identities | Positives | Gaps |
|---|---|---|---|---|---|
| 28.4 bits(57) | 957 | Composition-based stats. | 13/31(42%) | 17/31(54%) | 0/31(0%) |

```
Query  12   AEKASTNSETNRGESEKKRNLGELSRTTSED  42
            AE      +ET RGE+EK R    E   R   +E+
Sbjct  447  AESKRVAAETGRGEAEKLRVTAETERVEAEN  477
```

---



# results for RID-PYJKZEF7016

- Recent Results
- Saved Strategies
- Help

- 

- Save Search
- Search Summary

- How to read this report?
- BLAST Help Videos
- Back to Traditional Results Page

Your search is limited to records that include: Clostridium difficle (taxid:1496)

- 

Job Title

P02686:RecName: Full=Myelin basic protein;...

RID

PYJKZEF7016 Search expires on 12-06 23:06 pm

- Download All

Program

BLASTP Help

- Citation

Database

nr

- See details

Query ID

P02686.3

Description

RecName: Full=Myelin basic protein; Short=MBP; AltName: Full=Myelin A1 protein; AltName: Full=Myelin membrane encephalitogenic protein [Homo sapiens] ...

Molecule type

amino acid

Query Length

304

Other reports

Distance tree of results Multiple alignment MSA viewer Help

# Filter Results

Organism only top 20 will appear
exclude
Add organism

Percent Identity from Percent Identity to
E value from E value to
Query Coverage from Query Coverage to
Compare these results against the new Clustered nr database Help

- 
- 
- 
- 
- 
- Alignment view
- 
- Help
- 
- 
- 

80 sequences selected

- Help

Download GenPeptGraphics Next Previous Descriptions

hypothetical protein [Clostridioides difficile]
Sequence ID: MDU1095993.1Length: 106Number of Matches: 1
Related Information
Identical Proteins-Identical proteins to WP_111918167.1
Range 1: 29 to 87GenPeptGraphics Next Match Previous Match

Alignment statistics for match #1

| Score | Expect | Method | Identities | Positives | Gaps |
|---|---|---|---|---|---|
| 29.3 bits(91) | 8.1 | Compositional matrix adjust. | 10/59(17%) | 34/59(57%) | 3/59(5%) |

```
Query  46   FGEADANQNNGTSSQDTAVTDSKRTADPKNAWQDAHPADP---GSRPHLIRLFSRDAPG  101
            F + D +Q++ T++++ A  + ++    ++ W++ +  D    G + +L R++ +++ +
Sbjct  29   FIDNDVEQKELTAARQKAYEELRKIDTENKHWEELDNIDCAYVGEWINLCRFYFKQGVA  87
```

Download GenPeptGraphics Next Previous Descriptions

molybdopterin-dependent oxidoreductase [Clostridioides difficile]
Sequence ID: MCI9976216.1Length: 746Number of Matches: 1
Range 1: 195 to 235GenPeptGraphics Next Match Previous Match

Alignment statistics for match #1

| Score | Expect | Method | Identities | Positives | Gaps |
|---|---|---|---|---|---|
| 30.3 bits(95) | 21 | Compositional matrix adjust. | 13/44(30%) | 25/44(56%) | 3/44(6%) |

```
Query  257  PGFGYGGRASDYKSAHKGFKGVDAQGTLSKIFKLGGRDSRSGSP  300
            ++++Y+G+ + Y + ++ F      +  LSKIF L+    R  SP
Sbjct  195  ADYDYSGKLTVYSPNQNTF---AFRVILSKIFELSMNKVRLVSP  235
```

Download GenPeptGraphics Next Previous Descriptions

hypothetical protein [Clostridioides difficile]

Sequence ID: HBG3822393.1Length: 403Number of Matches: 1

Range 1: 322 to 382GenPeptGraphics Next Match Previous Match

Alignment statistics for match #1

| Score | Expect | Method | Identities | Positives | Gaps |
|---|---|---|---|---|---|
| 29.3 bits(91) | 37 | Compositional matrix adjust. | 10/61(16%) | 31/61(50%) | 0/61(0%) |

```
Query  196  HPARTAHYGSLPQKSHGRTQDENPVVHFFKNIVTPRTPPPSQGKGRGLSLSRFSWGAEGQ  255
            +P +  HY+ +P++     ++ ++   ++ +IVT +     +  K +   + +F W  + +
Sbjct  322  NPYKKLHYSIIPHRVCKNIENSKSKNYYYWEIVTKHIRRKGFFKMKKRIFFKFKWNRDTN  381

Query  256  R  256
            +
Sbjct  382  K  382
```

Download GenPeptGraphics Next Previous Descriptions

DEAD/DEAH box helicase family protein [Clostridioides difficile]

Sequence ID: MCD8746199.1Length: 684Number of Matches: 1

Range 1: 598 to 658GenPeptGraphics Next Match Previous Match

Alignment statistics for match #1

| Score | Expect | Method | Identities | Positives | Gaps |
|---|---|---|---|---|---|
| 29.0 bits(90) | 49 | Compositional matrix adjust. | 10/61(16%) | 31/61(50%) | 0/61(0%) |

```
Query  196  HPARTAHYGSLPQKSHGRTQDENPVVHFFKNIVTPRTPPPSQGKGRGLSLSRFSWGAEGQ  255
            +P +  HY+ +P++     ++ ++   ++ +IVT +     +  K +   + +F W  + +
Sbjct  598  NPYKKLHYSIIPHRVCKNIENSKSKNYYYWEIVTKHIRRKGFFKMKKRIFFKFKWNRDTN  657

Query  256  R  256
            +
Sbjct  658  K  658
```

Download GenPeptGraphics Next Previous Descriptions

DEAD/DEAH box helicase family protein [Clostridioides difficile]

Sequence ID: MCA0551516.1Length: 689Number of Matches: 1

Range 1: 598 to 658GenPeptGraphics Next Match Previous Match

Alignment statistics for match #1

| Score | Expect | Method | Identities | Positives | Gaps |
|---|---|---|---|---|---|
| 29.0 bits(90) | 51 | Compositional matrix adjust. | 10/61(16%) | 31/61(50%) | 0/61(0%) |

```
Query  196   HPARTAHYGSLPQKSHGRTQDENPVVHFFKNIVTPRTPPPSQGKGRGLSLSRFSWGAEGQ  255
             +P +   HY+ +P++      ++ ++    ++ +IVT +     +   K +    + +F W   + +
Sbjct  598   NPYKKLHYSIIPHRVCKNIENSKSKNYYYWEIVTKHIRRKGFFKMKKRIFFKFKWNRDTN  657

Query  256   R  256
             +
Sbjct  658   K  658
```

Download GenPeptGraphics Next Previous Descriptions

Flp pilus assembly protein, ATPase CpaF [Clostridioides difficile]

Sequence ID: VTR09234.1Length: 402Number of Matches: 1

Related Information

AlphaFold Structure-3D structure displays

Identical Proteins-Identical proteins to WP_004022101.1

Range 1: 117 to 206GenPeptGraphics Next Match Previous Match

Alignment statistics for match #1

| Score | Expect | Method | Identities | Positives | Gaps |
|---|---|---|---|---|---|
| 28.5 bits(88) | 61 | Compositional matrix adjust. | 23/95(24%) | 50/95(52%) | 8/95(8%) |

```
Query  63    AVTDSKRTADPKNAWQDAHPADPGSRPHLIRLFSRDAPGREDNT---FKDRPSESDELQT  119
             A+   ++R   D++ +W  D++ ++ G  R     +RL +   +P    D T    ++     S  S +L +
Sbjct  117   ALGAGRRLDDAQ-PWVDGQLSNVGRRGYTVRLHAIIPPLAADGTCISLRVLRSASKDLPS  175

Query  120   IQEDSAATSESLDVMASQKRPSQRHGSKYLATAST  154
             + +++A   SE + V++          RH    +L   ++T
Sbjct  176   LIDNGAIPSEVVPVITD----ILRHRLAFLVIGGT  206
```

Download GenPeptGraphics Next Previous Descriptions

DEAD/DEAH box helicase family protein [Clostridioides difficile]

Sequence ID: MBH7142746.1Length: 658Number of Matches: 1

Range 1: 567 to 627GenPeptGraphics Next Match Previous Match

Alignment statistics for match #1

| Score | Expect | Method | Identities | Positives | Gaps |
|---|---|---|---|---|---|
| 28.7 bits(89) | 68 | Compositional matrix adjust. | 10/61(16%) | 31/61(50%) | 0/61(0%) |

```
Query  196   HPARTAHYGSLPQKSHGRTQDENPVVHFFKNIVTPRTPPPSQGKGRGLSLSRFSWGAEGQ  255
             +P +   HY+ +P++      ++ ++    ++ +IVT +     +   K +    + +F W   + +
Sbjct  567   NPYKKLHYSIIPHRVCKNIENSKSKNYYYWEIVTKHIRRKGFFKMKKRIFFKFKWNRDTN  626
```

```
Query  256  R  256
            +
Sbjct  627  K  627
```

Download GenPeptGraphics Next Previous Descriptions
zinc-binding dehydrogenase [Clostridioides difficile]
Sequence ID: MBS5788714.1Length: 355Number of Matches: 1
Related Information
Identical Proteins-Identical proteins to WP_312214935.1
Range 1: 4 to 44GenPeptGraphics Next Match Previous Match

Alignment statistics for match #1

| Score | Expect | Method | Identities | Positives | Gaps |
|---|---|---|---|---|---|
| 27.4 bits(84) | 133 | Compositional matrix adjust. | 11/41(27%) | 23/41(56%) | 0/41(0%) |

```
Query  203   YGSLPQKSHGRTQDENPVVHFFKNIVTPRTPPPSQGKGRGL  243
             YG ++   + G+ + E+PV    +  I+ PR  +P  +  ++L
Sbjct  4     YGMISVGNAGWLEKEKPVAGPYDAIIRPRVVAPCTSDVHAL  44
```

Download GenPeptGraphics Next Previous Descriptions
helix-turn-helix family protein [Clostridioides difficile P28]
Sequence ID: EQJ64203.1Length: 194Number of Matches: 1

- See 1 more title(s) See all Identical Proteins(IPG)

Related Information
Gene-associated gene details
AlphaFold Structure-3D structure displays
Identical Proteins-Identical proteins to WP_002607172.1
Range 1: 11 to 44GenPeptGraphics Next Match Previous Match

Alignment statistics for match #1

| Score | Expect | Method | Identities | Positives | Gaps |
|---|---|---|---|---|---|
| 26.6 bits(81) | 168 | Compositional matrix adjust. | 15/34(44%) | 22/34(64%) | 1/34(2%) |

```
Query  165   RHRDTGILDSIGRFFGGDRGAPKR-GSGKDSHHP  197
             RHRD+   L+S+G  F  G   +++ KR   SG   S+ P
Sbjct  11    RHRDSLSLESVGDFVGVSKSTVKRWESGESSNVP  44
```

fficial website of the United States government

Skip to main page content

# results for RID-PYJV4TY6013

- Home
- Recent Results
- Saved Strategies
- Help

- 

- Save Search
- Search Summary

- How to read this report?
- BLAST Help Videos
- Back to Traditional Results Page

Your search is limited to records that include: Clostridium perfringens (taxid:1502)

- 

Job Title

P02686:RecName: Full=Myelin basic protein;...

RID

PYJV4TY6013 Search expires on 12-06 23:10 pm

- Download All

Program

BLASTP Help

- Citation

Database

nr

- See details

Query ID

P02686.3

Description

RecName: Full=Myelin basic protein; Short=MBP; AltName: Full=Myelin A1 protein; AltName: Full=Myelin membrane encephalitogenic protein [Homo sapiens] ...

Molecule type

amino acid

Query Length

304

Other reports

Distance tree of results Multiple alignment MSA viewer Help

# Filter Results

Organism only top 20 will appear
exclude
Add organism

Percent Identity from Percent Identity to
E value from E value to
Query Coverage from Query Coverage to
Compare these results against the new Clustered nr database Help

- 
- Alignment view
- Help

262 sequences selected

- Help

Download GenPeptGraphics

Sort by:

Next Previous Descriptions

hypothetical protein [Clostridium perfringens]
Sequence ID: MDJ8959718.1Length: 1172Number of Matches: 2
Range 1: 383 to 508GenPeptGraphics Next Match Previous Match

Alignment statistics for match #1

| Score | Expect | Method | Identities | Positives | Gaps |
|---|---|---|---|---|---|
| 33.1 bits(68) | 4.8 | Composition-based stats. | 32/139(23%) | 59/139(42%) | 24/139(17%) |

```
Query  12    AEKASTNSETNRGESEKKRNLGELSRTTSEDNEVFGEADAN--------QNNGTSSQDTA  63
             +EKA  ++E  RG +E +R   E  R  SE   V  E + N        + N T +Q+
Sbjct  383  SEKARNDAEELRGTAESERVKSEKLRVDSEKARVLAEEERNTTFTQKEEERNNTFTQNET  442

Query  64    ---VTDSKRTADPKNAWQDAHPADPGSRPHLIRLFSRDAPGREDNTFKDRPSESDELQTI  120
                T ++   + K A ++A  A    +    +++ +                 E+++L+ +
Sbjct  443  NRNATFTQSEEERKTAFEEAENARKVAESKRVKVETSRV-------------EAEKLRVV  489

Query  121  QEDSAATSESLDVMASQKR  139
              E     +ESL V A ++R
Sbjct  490  AETGRVEAESLRVTAEEER  508
```

Range 2: 167 to 208GenPeptGraphics Next Match Previous Match First Match

Alignment statistics for match #2

| Score | Expect | Method | Identities | Positives | Gaps |
|---|---|---|---|---|---|
| 26.7 bits(53) | 403 | Composition-based stats. | 16/43(37%) | 23/43(53%) | 1/43(2%) |

```
Query  8     RELNAEKASTNSETNRGESEKKRNLGELSRTTSEDNEVFGEAD  50
             RE N EKA   SE  R   E++R    EL R  +E++    E++
Sbjct  167  REKN-EKARLQSEDIRITGEEERKTNELERIQAENDRKVSESN  208
```

Download GenPeptGraphics Next Previous Descriptions

cell wall-binding protein [Clostridium perfringens]

Sequence ID: WP_265860424.1Length: 1623Number of Matches: 1

- See 1 more title(s) See all Identical Proteins(IPG)

Related Information

Identical Proteins-Identical proteins to WP_265860424.1

Range 1: 172 to 253GenPeptGraphics Next Match Previous Match

Alignment statistics for match #1

| Score | Expect | Method | Identities | Positives | Gaps |
|---|---|---|---|---|---|
| 33.1 bits(68) | 5.4 | Composition-based stats. | 27/93(29%) | 39/93(41%) | 28/93(30%) |

```
Query  3     NHAGKRELNAEK-ASTNSETNRGESEKKRNLGELSRTTSEDNEVFGEADANQ--------  53
             N    K E+ +E+   S + ET + + E K+  GE         + EV  + D NQ
Sbjct  172  NRDKKVEVKSEEIKSKDNETSKRDEESKKIKGE-------NKEVESKLDKNQALKSDYIN  224

Query  54    --------NNGTSSQDTAVTDSKRTADPKNAWQ  78
                      GT SQ+ +VTD   +      KN WQ
Sbjct  225  INESGQALTVGTESQNNSVTDENK----KNGWQ  253
```

Download GenPeptGraphics Next Previous Descriptions

cell wall-binding protein [Clostridium perfringens]

Sequence ID: WP_164786888.1Length: 1701Number of Matches: 1

- See 2 more title(s) See all Identical Proteins(IPG)

Related Information

Identical Proteins-Identical proteins to WP_164786888.1

Range 1: 172 to 253GenPeptGraphics Next Match Previous Match

Alignment statistics for match #1

| Score | Expect | Method | Identities | Positives | Gaps |
|---|---|---|---|---|---|
| 32.7 bits(67) | 5.6 | Composition-based stats. | 27/93(29%) | 39/93(41%) | 28/93(30%) |

```
Query  3    NHAGKRELNAEK-ASTNSETNRGESEKKRNLGELSRTTSEDNEVFGEADANQ--------  53
            N   K E+ +E+   S + ET + + E K+  GE         + EV  + D NQ
Sbjct  172  NRDKKVEVKSEEIKSKDNETSKRDEESKKIKGE-------NKEVESKLDKNQALKSDYIN  224

Query  54   --------NNGTSSQDTAVTDSKRTADPKNAWQ  78
                    GT SQ+ +VTD  +     KN WQ
Sbjct  225  INESGQALTVGTESQNNSVTDENK----KNGWQ  253
```

Download GenPeptGraphics

Sort by:

Next Previous Descriptions

hypothetical protein [Clostridium perfringens]

Sequence ID: MDU7726947.1Length: 1097Number of Matches: 3

Range 1: 263 to 301GenPeptGraphics Next Match Previous Match

Alignment statistics for match #1

| Score | Expect | Method | Identities | Positives | Gaps |
|---|---|---|---|---|---|
| 32.7 bits(67) | 5.9 | Composition-based stats. | 20/41(49%) | 25/41(60%) | 2/41(4%) |

```
Query  9    ELNAEKASTNSETNRGESEKKRNLGELSRTTSEDNEVFGEA  49
            + N+E A T+ E NR ESEK R L E  R +SE+  V  EA
Sbjct  263  KFNSE-AKTD-ELNRKESEKNRVLAEEERKSSEEARVSAEA  301
```

Range 2: 389 to 451GenPeptGraphics Next Match Previous Match First Match

Alignment statistics for match #2

| Score | Expect | Method | Identities | Positives | Gaps |
|---|---|---|---|---|---|
| 31.4 bits(64) | 17 | Composition-based stats. | 20/63(32%) | 28/63(44%) | 4/63(6%) |

```
Query  12   AEKASTNSETNRGESEKKRNLGELSRTTSEDNEVFGEADAN----QNNGTSSQDTAVTDS  67
            +EKA  ++E  R  SE +R   E  R  SE   V  E + N    QN     +   V +
Sbjct  389  SEKARNDAEELRVTSENERTTAERLRVDSEKARVLAEEERNNTFTQNETNRNETFEVAEE  448
```

```
Query  68   KRT  70
            +RT
Sbjct  449  ERT  451
```

Range 3: 297 to 421GenPeptGraphics Next Match Previous Match First Match

Alignment statistics for match #3

| Score | Expect | Method | Identities | Positives | Gaps |
|---|---|---|---|---|---|
| 28.8 bits(58) | 108 | Composition-based stats. | 33/143(23%) | 61/143(42%) | 31/143(21%) |

```
Query  10   LNAEKASTNSETNRGESEKKRNLGELSRTTSEDNEVFGEADANQNNGTSSQD----TAVT  65
            + AE     N+ T+   ESE++R   E+ R  +E    V   EA+  ++N  S ++     T+
Sbjct  297  VSAEAIRQNTYTDFNESEEERKSNEIKRQEAEALRV--EAETRRDNLFSEKEEERNTSFI  354

Query  66   DSKRTADPK-NAWQDAHPADPGSRPHLIRLFSRDAPGREDNTFKDRP--------SESDE  116
            +S++   +    N  QD                SR+     E  T ++             +++E
Sbjct  355  ESEKVRNAAFNKAQD----------------SRNTTFEESETIREEAFKISEKARNDAEE  398

Query  117  LQTIQEDSAATSESLDVMASQKR  139
             L+    E+    T+E L V + + R
Sbjct  399  LRVTSENERTTAERLRVDSEKAR  421
```

Download GenPeptGraphics Next Previous Descriptions

cell wall-binding protein [Clostridium perfringens]

Sequence ID: MDK0651334.1Length: 1558Number of Matches: 1

Range 1: 172 to 253GenPeptGraphics Next Match Previous Match

Alignment statistics for match #1

| Score | Expect | Method | Identities | Positives | Gaps |
|---|---|---|---|---|---|
| 32.7 bits(67) | 6.7 | Composition-based stats. | 26/86(30%) | 39/86(45%) | 14/86(16%) |

```
Query  3    NHAGKRELNAEK-ASTNSETNRGESEKKRNLGELSRTTS--EDNEVFGEADANQNN----  55
            N    K E+ +E+   S  + ET  +  + E  K+   GE    + S   + N+         N N
Sbjct  172  NRDNKVEVKSEEIKSKDNETSKRDEESKKIKGENKESESKLDKNKALKSDYININESVQA  231

Query  56   ---GTSSQDTAVTDSKRTADPKNAWQ  78
               GT SQ+ +VTD      + KN WQ
Sbjct  232  LTVGTESQNNSVTDE----NEKNGWQ  253
```

Download GenPeptGraphics Next Previous Descriptions

cell wall-binding protein [Clostridium perfringens]

Sequence ID: WP_049040193.1Length: 1558Number of Matches: 1

---

- See 3 more title(s) See all Identical Proteins(IPG)

Related Information

Identical Proteins-Identical proteins to WP_049040193.1

Range 1: 172 to 253GenPeptGraphics Next Match Previous Match

Alignment statistics for match #1

| Score | Expect | Method | Identities | Positives | Gaps |
|---|---|---|---|---|---|
| 32.7 bits(67) | 6.7 | Composition-based stats. | 26/86(30%) | 39/86(45%) | 14/86(16%) |

```
Query  3    NHAGKRELNAEK-ASTNSETNRGESEKKRNLGELSRTTS--EDNEVFGEADANQNN----  55
            N   K E+ +E+   S + ET + + E K+  GE    + S  + N+        N N
Sbjct  172  NRDNKVEVKSEEIKSKDNETSKRDEESKKIKGENKESESKLDKNKALKSDYININESVQA  231

Query  56   ---GTSSQDTAVTDSKRTADPKNAWQ  78
               GT SQ+ +VTD      + KN WQ
Sbjct  232  LTVGTESQNNSVTDE----NEKNGWQ  253
```

Download GenPeptGraphics Next Previous Descriptions

cell wall-binding protein [Clostridium perfringens]

Sequence ID: MDK0910287.1Length: 1282Number of Matches: 1

Range 1: 172 to 253GenPeptGraphics Next Match Previous Match

Alignment statistics for match #1

| Score | Expect | Method | Identities | Positives | Gaps |
|---|---|---|---|---|---|
| 32.7 bits(67) | 7.4 | Composition-based stats. | 26/86(30%) | 38/86(44%) | 14/86(16%) |

```
Query  3    NHAGKRELNAEK-ASTNSETNRGESEKKRNLGELSRTTS--EDNEVFGEADANQNN----  55
            N   K E+ +E+   S + ET + + E K+  GE      S  + N+        N N
Sbjct  172  NRDNKVEVKSEEIKSKDNETSKRDEESKKIKGENKEVESKLDKNQALKSDYININESGQA  231

Query  56   ---GTSSQDTAVTDSKRTADPKNAWQ  78
               GT SQ+ +VTD      + KN WQ
Sbjct  232  VIAGTESQNNSVTDE----NEKNGWQ  253
```

Download GenPeptGraphics

Sort by:

---

Next Previous Descriptions

hypothetical protein [Clostridium perfringens]

Sequence ID: MDM0998687.1Length: 1164Number of Matches: 3

Range 1: 388 to 446GenPeptGraphics Next Match Previous Match

Alignment statistics for match #1

| Score | Expect | Method | Identities | Positives | Gaps |
|---|---|---|---|---|---|
| 32.3 bits(66) | 7.6 | Composition-based stats. | 20/59(34%) | 29/59(49%) | 6/59(10%) |

```
Query  3    NHAGKRELNAEKASTNSETNRGESEKKRNLGELSRTTS------EDNEVFGEADANQNN  55
            N A +  + AE+    SE  R +SEK R L E  R T+      E N  F + + N+N+
Sbjct  388  NDAEELRVTAERERVKSEKLRVDSEKARVLAEEERNTTFTQKEEERNNTFTQNETNRND  446
```

Range 2: 469 to 507GenPeptGraphics Next Match Previous Match First Match

Alignment statistics for match #2

| Score | Expect | Method | Identities | Positives | Gaps |
|---|---|---|---|---|---|
| 27.5 bits(55) | 222 | Composition-based stats. | 14/39(36%) | 19/39(48%) | 0/39(0%) |

```
Query  12   AEKASTNSETNRGESEKKRNLGELSRTTSEDNEVFGEAD  50
            AE     SE+ R E+EK R   E  R  +E+  V  E +
Sbjct  469  AESKRVKSESSRAEAEKVRVSAETRRVEAENLRVTAEEE  507
```

Range 3: 257 to 309GenPeptGraphics Next Match Previous Match First Match

Alignment statistics for match #3

| Score | Expect | Method | Identities | Positives | Gaps |
|---|---|---|---|---|---|
| 25.8 bits(51) | 713 | Composition-based stats. | 25/56(45%) | 31/56(55%) | 3/56(5%) |

```
Query  9    ELNAEKASTNSETNRGESEKKRNLGELSRTTSEDNEVFGEADANQNNGTSSQDTAV  64
            E N+E A T+ E NR E+EK R L E  R +SE+  V  EA   QN  T   D+ V
Sbjct  257  EFNSE-AKTD-ELNRKEAEKNRVLAEEERKSSEESRVAAEA-IRQNTYTDFNDSEV  309
```

Download GenPeptGraphics Next Previous Descriptions

cell wall-binding protein [Clostridium perfringens]

Sequence ID: MDM1009651.1Length: 1557Number of Matches: 1

Range 1: 173 to 248GenPeptGraphics Next Match Previous Match

Alignment statistics for match #1

| Score | Expect | Method | Identities | Positives | Gaps |
|---|---|---|---|---|---|
| 32.3 bits(66) | 7.8 | Composition-based stats. | 24/80(30%) | 35/80(43%) | 13/80(16%) |

```
Query  8    RELNAEKASTNSETNRGESEKKRNLGELSRTTS--EDNEVFGEADANQNN-------GTS  58
            R+   E  S + ET + + E K+  GE   + S  + N+       N N        GT
Sbjct  173  RDKKVEVKSKDNETSKRDEESKKIKGENKESESKLDKNKALKSDYININESGQALTVGTE  232

Query  59   SQDTAVTDSKRTADPKNAWQ  78
            SQ+ +VTD     + KN WQ
Sbjct  233  SQNNSVTDE----NEKNGWQ  248
```

Download GenPeptGraphics Next Previous Descriptions

hypothetical protein [Clostridium perfringens]

Sequence ID: WP_164808655.1Length: 1210Number of Matches: 1

- [See 1 more title(s)]() [See all Identical Proteins(IPG)]()

Related Information

Identical Proteins-Identical proteins to WP_164808655.1

Range 1: 173 to 248GenPeptGraphics Next Match Previous Match

Alignment statistics for match #1

| Score | Expect | Method | Identities | Positives | Gaps |
| --- | --- | --- | --- | --- | --- |
| 32.3 bits(66) | 8.1 | Composition-based stats. | 24/80(30%) | 35/80(43%) | 13/80(16%) |

```
Query  8    RELNAEKASTNSETNRGESEKKRNLGELSRTTS--EDNEVFGEADANQNN-------GTS  58
            R+   E  S + ET + + E K+  GE   + S  + N+       N N        GT
Sbjct  173  RDKKVEVKSKDNETSKRDEESKKIKGENKESESKLDKNKALKSDYININESGQALTVGTE  232

Query  59   SQDTAVTDSKRTADPKNAWQ  78
            SQ+ +VTD     + KN WQ
Sbjct  233  SQNNSVTDE----NEKNGWQ  248
```

Download GenPeptGraphics Next Previous Descriptions

cell wall-binding protein [Clostridium perfringens]

Sequence ID: EGT3613252.1Length: 1557Number of Matches: 1

Range 1: 173 to 248GenPeptGraphics Next Match Previous Match

Alignment statistics for match #1

| Score | Expect | Method | Identities | Positives | Gaps |
| --- | --- | --- | --- | --- | --- |
| 32.3 bits(66) | 8.1 | Composition-based stats. | 24/80(30%) | 35/80(43%) | 13/80(16%) |

```
Query  8    RELNAEKASTNSETNRGESEKKRNLGELSRTTS--EDNEVFGEADANQNN-------GTS  58
            R+   E  S + ET + + E K+  GE   + S  + N+       N N        GT
Sbjct  173  RDKKVEVKSKDNETSKRDEESKKIKGENKESESKLDKNKALKSDYININESGQALTVGTE  232

Query  59   SQDTAVTDSKRTADPKNAWQ  78
            SQ+ +VTD     + KN WQ
Sbjct  233  SQNNSVTDE----NEKNGWQ  248
```

Download GenPeptGraphics Next Previous Descriptions

cell wall-binding protein [Clostridium perfringens]

Sequence ID: WP_208335829.1Length: 1559Number of Matches: 1

- See 1 more title(s) See all Identical Proteins(IPG)

Related Information

Identical Proteins-Identical proteins to WP_208335829.1

Range 1: 173 to 254GenPeptGraphics Next Match Previous Match

Alignment statistics for match #1

| Score | Expect | Method | Identities | Positives | Gaps |
|---|---|---|---|---|---|
| 32.3 bits(66) | 9.5 | Composition-based stats. | 26/86(30%) | 38/86(44%) | 14/86(16%) |

```
Query  3    NHAGKRELNAEK-ASTNSETNRGESEKKRNLGELSRTTSE--DNEVFGEADANQNN----  55
            N    K E+ +E+   S + ET + + E K+   GE      S+    N+         N N
Sbjct  173  NRDNKVEVKSEEIKSKDNETSKRDEESKKIKGENKEVESKLGKNQALKSDYININESGQA  232

Query  56   ---GTSSQDTAVTDSKRTADPKNAWQ  78
               GT SQ+ +VTD      + KN WQ
Sbjct  233  LIVGTESQNNSVTDE----NEKNGWQ  254
```

Download GenPeptGraphics Next Previous Descriptions

hypothetical protein [Clostridium perfringens]

Sequence ID: EGT0000563.1Length: 1216Number of Matches: 1

Range 1: 173 to 254GenPeptGraphics Next Match Previous Match

Alignment statistics for match #1

| Score | Expect | Method | Identities | Positives | Gaps |
|---|---|---|---|---|---|
| 32.3 bits(66) | 9.7 | Composition-based stats. | 26/86(30%) | 38/86(44%) | 14/86(16%) |

```
Query  3    NHAGKRELNAEK-ASTNSETNRGESEKKRNLGELSRTTS--EDNEVFGEADANQNN----  55
            N    K E+ +E+   S + ET + + E K+   GE      S   + N+         N N
Sbjct  173  NRDKKLEVKSEEIKSKDNETSKRDEESKKIKGENKEVESKLDKNQALKSDYININESGQA  232

Query  56   ---GTSSQDTAVTDSKRTADPKNAWQ  78
               GT SQ+ +VTD      + KN WQ
Sbjct  233  LIVGTESQNNSVTDE----NEKNGWQ  254
```

Download GenPeptGraphics Next Previous Descriptions

cell wall-binding protein [Clostridium perfringens]

Sequence ID: MDU4074061.1Length: 1624Number of Matches: 1

Range 1: 173 to 254GenPeptGraphics Next Match Previous Match

Alignment statistics for match #1

| Score | Expect | Method | Identities | Positives | Gaps |
|---|---|---|---|---|---|
| 32.3 bits(66) | 9.7 | Composition-based stats. | 27/93(29%) | 39/93(41%) | 28/93(30%) |

```
Query  3    NHAGKRELNAEK-ASTNSETNRGESEKKRNLGELSRTTSEDNEVFGEADANQ--------  53
            N    K E+ +E+   S + ET + + E K+   GE          + EV   + D NQ
Sbjct  173  NRDKKVEVKSEEIKSKDNETSKIDEESKKIKGE-------NKEVESKLDKNQALKSDYIN  225

Query  54   --------NNGTSSQDTAVTDSKRTADPKNAWQ  78
                    GT SQ+ +VTD  +      KN WQ
Sbjct  226  INESGQALTVGTESQNNSVTDENK----KNGWQ  254
```

[Download](#) [GenPeptGraphics](#)
Sort by:

---

Next Previous [Descriptions](#)

hypothetical protein [Clostridium perfringens]
Sequence ID: [WP_197736970.1](#)Length: 1225Number of Matches: 3

- [See 2 more title(s)](#) [See all Identical Proteins(IPG)](#)

Related Information
[Identical Proteins](#)-Identical proteins to WP_197736970.1
Range 1: 395 to 452[GenPeptGraphics](#) Next Match Previous Match

Alignment statistics for match #1

| Score | Expect | Method | Identities | Positives | Gaps |
|---|---|---|---|---|---|
| 31.9 bits(65) | 10 | Composition-based stats. | 20/58(34%) | 28/58(48%) | 6/58(10%) |

```
Query  3    NHAGKRELNAEKASTNSETNRGESEKKRNLGELSRTTS------EDNEVFGEADANQN  54
            N A +  + AE+    SE  R +SEK R L E  R T+      E N  F + + N+N
Sbjct  395  NDAEELRVTAERERVKSEKLRVDSEKARVLAEEERNTTFTQKEEERNNTFTQNETNRN  452
```

Range 2: 490 to 546[GenPeptGraphics](#) Next Match Previous Match [First Match](#)

Alignment statistics for match #2

| Score | Expect | Method | Identities | Positives | Gaps |
|---|---|---|---|---|---|
| 27.1 bits(54) | 334 | Composition-based stats. | 20/60(33%) | 25/60(41%) | 3/60(5%) |

```
Query  12   AEKASTNSETNRGESEKKRNLGELSRTTSEDNEVFGEADANQNNGTSSQDTAVTDSKRTA  71
            AEK    +ET R E+E  R + E  R  +E      E   N   G  S +T      RTA
Sbjct  490  AEKLRVTAETERVEAENLRVISEEERVAAETKR---EEGFNAFEGKISANTKELKGARTA  546
```

Range 3: 476 to 514[GenPeptGraphics](#) Next Match Previous Match [First Match](#)

Alignment statistics for match #3

| Score | Expect | Method | Identities | Positives | Gaps |
|---|---|---|---|---|---|
| 26.2 bits(52) | 597 | Composition-based stats. | 14/39(36%) | 19/39(48%) | 0/39(0%) |

```
Query  12   AEKASTNSETNRGESEKKRNLGELSRTTSEDNEVFGEAD  50
            AE     +ET R E+EK R   E  R  +E+  V   E +
Sbjct  476  AESKRVAAETGRVEAEKLRVTAETERVEAENLRVISEEE  514
```

---

[Download](#) [GenPeptGraphics](#) Next Previous [Descriptions](#)

hypothetical protein [Clostridium perfringens]
Sequence ID: [MDK0668143.1](#)Length: 938Number of Matches: 1

- See 1 more title(s) See all Identical Proteins(IPG)

Related Information

Identical Proteins-Identical proteins to MDK0668143.1

Range 1: 172 to 253GenPeptGraphics Next Match Previous Match

Alignment statistics for match #1

| Score | Expect | Method | Identities | Positives | Gaps |
|---|---|---|---|---|---|
| 31.9 bits(65) | 11 | Composition-based stats. | 26/86(30%) | 39/86(45%) | 14/86(16%) |

```
Query  3    NHAGKRELNAEK-ASTNSETNRGESEKKRNLGELSRTTS--EDNEVFGEADANQNN----  55
            N   K E+ +E+  S + ET + + E K+  GE   + S  + N+       N N
Sbjct  172  NRDNKVEVKSEEIKSKDNETSKRDEESKKIKGENKESESKLDKNKALKSDYININESVQA  231

Query  56   ---GTSSQDTAVTDSKRTADPKNAWQ  78
               GT SQ+ +VTD     + KN WQ
Sbjct  232  LTVGTESQNNSVTDE----NEKNGWQ  253
```

Download GenPeptGraphics

Sort by:

Next Previous Descriptions

hypothetical protein [Clostridium perfringens]

Sequence ID: MDK0563172.1Length: 1194Number of Matches: 2

Range 1: 388 to 446GenPeptGraphics Next Match Previous Match

Alignment statistics for match #1

| Score | Expect | Method | Identities | Positives | Gaps |
|---|---|---|---|---|---|
| 31.9 bits(65) | 12 | Composition-based stats. | 20/59(34%) | 28/59(47%) | 6/59(10%) |

```
Query  3    NHAGKRELNAEKASTNSETNRGESEKKRNLGELSRTTS------EDNEVFGEADANQNN  55
            N A +  + AE     SE  R +SEK R L E  R T+      E N  F + + N+N+
Sbjct  388  NDAEELRVTAESERVKSEKLRVDSEKARVLAEEERNTTFTQKEEERNNTFTQNETNRND  446
```

Range 2: 469 to 505GenPeptGraphics Next Match Previous Match First Match

Alignment statistics for match #2

| Score | Expect | Method | Identities | Positives | Gaps |
|---|---|---|---|---|---|
| 26.2 bits(52) | 537 | Composition-based stats. | 14/37(38%) | 18/37(48%) | 0/37(0%) |

```
Query  12   AEKASTNSETNRGESEKKRNLGELSRTTSEDNEVFGE  48
            AE     +ET R E+EK R   E  R  +E+  V  E
Sbjct  469  AESKRVAAETGRVEAEKLRVTAETERVEAENLRVISE  505
```

Download GenPeptGraphics Next Previous Descriptions

cell wall-binding protein [Clostridium perfringens]

Sequence ID: MDK0615158.1Length: 1215Number of Matches: 1

Range 1: 172 to 253GenPeptGraphics Next Match Previous Match

Alignment statistics for match #1

| Score | Expect | Method | Identities | Positives | Gaps |
|---|---|---|---|---|---|
| 31.9 bits(65) | 12 | Composition-based stats. | 26/86(30%) | 38/86(44%) | 14/86(16%) |

```
Query  3    NHAGKRELNAEK-ASTNSETNRGESEKKRNLGELSRTTS--EDNEVFGEADANQNN----  55
            N   K E+ +E+  S + ET + + E K+  GE     S  + N+       N N
Sbjct  172  NRDKKVEVKSEEIKSKDNETSKRDEESKKIKGENKEVESKLDKNQALKSDYININESGQA  231

Query  56   ---GTSSQDTAVTDSKRTADPKNAWQ  78
               GT SQ+ +VTD     + KN WQ
Sbjct  232  LTVGTESQNNSVTDE----NEKNGWQ  253
```

Download GenPeptGraphics

Sort by:

Next Previous Descriptions

hypothetical protein [Clostridium perfringens]

Sequence ID: WP_221346387.1Length: 1129Number of Matches: 3

Range 1: 279 to 438GenPeptGraphics Next Match Previous Match

Alignment statistics for match #1

| Score | Expect | Method | Identities | Positives | Gaps |
|---|---|---|---|---|---|
| 31.9 bits(65) | 12 | Composition-based stats. | 42/162(26%) | 69/162(42%) | 23/162(14%) |

```
Query  5    AGKRELNAEKASTNSETNRGESEKKR-------NLGELSRTTSEDNEVFGEADANQNNGT  57
            A  + + AEKA   +E  R ++E +R       N  EL R   E N V   A+A + N
Sbjct  279  AEDKRVTAEKARAKAEEERVKAETRRENTYTDFNDSELERKNNEANRV--RAEAERVNAE  336

Query  58   SSQDTAVTDSKRTADPKNAWQDA--HPADPGS-RPHLIRLFSRD--APGRE--DNTF---  107
              ++ A  +S+R  +      +A    A+ G     ++R+ +    A G E  +NTF
Sbjct  337  NGRNEAYEESERVREEAFKVSEAARNEAEEGRVAAEVLRVEAEKLRAAGEENRNNTFTQS  396

Query  108  ----KDRPSESDELQTIQEDSAATSESLDVMASQKRPSQRHG  145
                KD   E++  + + E      ES  V A + R ++  G
Sbjct  397  EEERKDIFEEAENTRKVAESKRDENESSRVEAEKLRVTEETG  438
```

Range 2: 189 to 232GenPeptGraphics Next Match Previous Match First Match

Alignment statistics for match #2

| Score | Expect | Method | Identities | Positives | Gaps |
|---|---|---|---|---|---|
| 27.5 bits(55) | 226 | Composition-based stats. | 16/46(35%) | 22/46(47%) | 2/46(4%) |

```
Query  10   LNAEKASTNSETNRGESEKKRNLGELSRTTSEDNEVFGEADANQNN  55
            + AE     +E NR  +E  R L E +R T E   V  EA+ N+
```

```
Sbjct  189   IKAEAERKAAENNRKVAETNRKLAENTRKTNEAARV--EAELNRQS  232
```

Range 3: 156 to 297GenPeptGraphics Next Match Previous Match First Match

Alignment statistics for match #3

| Score | Expect | Method | Identities | Positives | Gaps |
|---|---|---|---|---|---|
| 27.1 bits(54) | 377 | Composition-based stats. | 37/146(25%) | 59/146(40%) | 22/146(15%) |

```
Query  12    AEKASTNSETNRGESEKKRNLGELSRTTSEDNEVFGEAD--ANQNNGTSSQDTAVTDSKR  69
             AE      E  R  SEK+R   E  RTT E + +   EA+  A +NN    +  A T+ K
Sbjct  156   AEIKRKANELLRERSEKERVRLESIRTTEEADRIKAEAERKAAENN----RKVAETNRKL  211

Query  70    TADPKNAWQDAHPADPGSRPHL-------------IRLFS---RDAPGREDNTFKDRPSE  113
               + +   + A        R  L             +R+ +   R       +N F+    E
Sbjct  212   AENTRKTNEAARVEAELNRQSLFEENEEIRNENENLRIEAEKERKLTFSNNNKFESERVE  271

Query  114   SDELQTIQEDSAATSESLDVMASQKR  139
             +++L+ I ED   T+E     A ++R
Sbjct  272   AEKLRAIAEDKRVTAEKARAKAEEER  297
```

Download GenPeptGraphics Next Previous Descriptions

cell wall-binding protein [Clostridium perfringens]

Sequence ID: MDM0926206.1Length: 1557Number of Matches: 1

Range 1: 173 to 248GenPeptGraphics Next Match Previous Match

Alignment statistics for match #1

| Score | Expect | Method | Identities | Positives | Gaps |
|---|---|---|---|---|---|
| 31.4 bits(64) | 14 | Composition-based stats. | 25/80(31%) | 35/80(43%) | 13/80(16%) |

```
Query  8     RELNAEKASTNSETNR--GESEKKRNLGELSRTTSEDNEVFGEADANQNN-------GTS  58
             R+ N E  S + ET +   ESEK     + S +  + N+       N N         GT
Sbjct  173   RDKNVEVKSKDNETSKRYEESEKIEVENKESESKLDKNQALKSDYININESGQALTVGTE  232

Query  59    SQDTAVTDSKRTADPKNAWQ  78
             SQ+ +VTD      + KN WQ
Sbjct  233   SQNNSVTDE----NEKNGWQ  248
```

Download GenPeptGraphics Next Previous Descriptions

cell wall-binding protein [Clostridium perfringens]

Sequence ID: MDK0939109.1Length: 1557Number of Matches: 1

Range 1: 173 to 248GenPeptGraphics Next Match Previous Match

Alignment statistics for match #1

| Score | Expect | Method | Identities | Positives | Gaps |
|---|---|---|---|---|---|

| Score | Expect | Method | Identities | Positives | Gaps |
|---|---|---|---|---|---|
| 31.4 bits(64) | 14 | Composition-based stats. | 25/80(31%) | 35/80(43%) | 13/80(16%) |

```
Query  8    RELNAEKASTNSETNR--GESEKKRNLGELSRTTSEDNEVFGEADANQNN-------GTS  58
            R+ N E  S + ET +   ESEK      + S +  + N+        N N         GT
Sbjct  173  RDKNVEVKSKDNETSKRYEESEKIEVENKESESKLDKNQALKSDYININESGQALTVGTE  232

Query  59   SQDTAVTDSKRTADPKNAWQ  78
            SQ+ +VTD      + KN WQ
Sbjct  233  SQNNSVTDE----NEKNGWQ  248
```

Download GenPeptGraphics Next Previous Descriptions

cell wall-binding protein [Clostridium perfringens]

Sequence ID: EHK2346405.1Length: 1557Number of Matches: 1

Range 1: 173 to 248GenPeptGraphics Next Match Previous Match

Alignment statistics for match #1

| Score | Expect | Method | Identities | Positives | Gaps |
|---|---|---|---|---|---|
| 31.4 bits(64) | 15 | Composition-based stats. | 25/80(31%) | 35/80(43%) | 13/80(16%) |

```
Query  8    RELNAEKASTNSETNR--GESEKKRNLGELSRTTSEDNEVFGEADANQNN-------GTS  58
            R+ N E  S + ET +   ESEK      + S +  + N+        N N         GT
Sbjct  173  RDKNVEVKSKDNETSKRYEESEKIEVENKESESKLDKNQALKSDYININESGQALTVGTE  232

Query  59   SQDTAVTDSKRTADPKNAWQ  78
            SQ+ +VTD      + KN WQ
Sbjct  233  SQNNSVTDE----NEKNGWQ  248
```

Download GenPeptGraphics Next Previous Descriptions

cell wall-binding protein [Clostridium perfringens]

Sequence ID: MDK0841025.1Length: 1559Number of Matches: 1

Range 1: 173 to 254GenPeptGraphics Next Match Previous Match

Alignment statistics for match #1

| Score | Expect | Method | Identities | Positives | Gaps |
|---|---|---|---|---|---|
| 31.4 bits(64) | 15 | Composition-based stats. | 26/86(30%) | 38/86(44%) | 14/86(16%) |

```
Query  3    NHAGKRELNAEK-ASTNSETNRGESEKKRNLGELSRTTS--EDNEVFGEADANQNN----  55
            N    K E+ +E+   S + ET + + E K+  GE      S   + N+        N N
Sbjct  173  NRDKKVEVRSEEIKSKDNETSKRDEESKKIEGENKEAESKLDKNQALKSDYININESGQA  232

Query  56   ---GTSSQDTAVTDSKRTADPKNAWQ  78
               GT SQ+ +VTD      + KN WQ
Sbjct  233  LTVGTESQNNSVTDE----NEKNGWQ  254
```

Download GenPeptGraphics Next Previous Descriptions
cell wall-binding protein [Clostridium perfringens]
Sequence ID: WP_283676285.1Length: 1189Number of Matches: 1
Range 1: 61 to 142GenPeptGraphics Next Match Previous Match

Alignment statistics for match #1

| Score | Expect | Method | Identities | Positives | Gaps |
| --- | --- | --- | --- | --- | --- |
| 31.4 bits(64) | 15 | Composition-based stats. | 26/86(30%) | 38/86(44%) | 14/86(16%) |

```
Query  3    NHAGKRELNAEK-ASTNSETNRGESEKKRNLGELSRTTS--EDNEVFGEADANQNN----  55
            N   K E+ +E+   S + ET + + E K+   GE      S   + N+        N N
Sbjct  61   NRDKKVEVRSEEIKSKDNETSKRDEESKKIEGENKEAESKLDKNQALKSDYININESGQA  120

Query  56   ---GTSSQDTAVTDSKRTADPKNAWQ  78
               GT SQ+ +VTD      + KN WQ
Sbjct  121  LTVGTESQNNSVTDE----NEKNGWQ  142
```

Download GenPeptGraphics Next Previous Descriptions
cell wall-binding protein [Clostridium perfringens]
Sequence ID: WP_131439925.1Length: 1563Number of Matches: 1

- See 2 more title(s) See all Identical Proteins(IPG)

Related Information
Identical Proteins-Identical proteins to WP_131439925.1
Range 1: 173 to 254GenPeptGraphics Next Match Previous Match

Alignment statistics for match #1

| Score | Expect | Method | Identities | Positives | Gaps |
| --- | --- | --- | --- | --- | --- |
| 31.4 bits(64) | 15 | Composition-based stats. | 26/86(30%) | 38/86(44%) | 14/86(16%) |

```
Query  3    NHAGKRELNAEK-ASTNSETNRGESEKKRNLGELSRTTS--EDNEVFGEADANQNN----  55
            N   K E+ +E+   S + ET + + E K+   GE      S   + N+        N N
Sbjct  173  NRDKKVEVRSEEIKSKDNETSKRDEESKKIEGENKEAESKLDKNQALKSDYININESGQA  232

Query  56   ---GTSSQDTAVTDSKRTADPKNAWQ  78
               GT SQ+ +VTD      + KN WQ
Sbjct  233  LTVGTESQNNSVTDE----NEKNGWQ  254
```

Download GenPeptGraphics

Sort by:

Next Previous Descriptions

hypothetical protein [Clostridium perfringens]
Sequence ID: HAT4146051.1Length: 1124Number of Matches: 4

Range 1: 400 to 449GenPeptGraphics Next Match Previous Match

Alignment statistics for match #1

| Score | Expect | Method | Identities | Positives | Gaps |
|---|---|---|---|---|---|
| 31.4 bits(64) | 16 | Composition-based stats. | 17/50(34%) | 26/50(52%) | 6/50(12%) |

```
Query  12   AEKASTNSETNRGESEKKRNLGELSRTTS------EDNEVFGEADANQNN  55
            AE+     +E+ R +SEK R L +  R T+       E N  F + + N+NN
Sbjct  400  AEEGRVEAESERVKSEKARALADEERNTTFTQKEEERNNTFTQNETNRNN  449
```

Range 2: 295 to 363GenPeptGraphics Next Match Previous Match First Match

Alignment statistics for match #2

| Score | Expect | Method | Identities | Positives | Gaps |
|---|---|---|---|---|---|
| 28.0 bits(56) | 176 | Composition-based stats. | 18/69(26%) | 32/69(46%) | 2/69(2%) |

```
Query  4    HAGKRELNAEKASTNSETNRGESEKKRNLGELSRTTSEDNEVFGEADANQ--NNGTSSQD  61
             A +  + AE     N+ T+  +SE +R    E  R  +E   V  E+  N+  N     ++
Sbjct  295  NAEEERIKAETRRENTYTDFNDSEVERKNNEANRVRAETERVKAESGRNELFNTNEEKRN  354

Query  62   TAVTDSKRT  70
            +   +S+RT
Sbjct  355  STFIESERT  363
```

Range 3: 192 to 235GenPeptGraphics Next Match Previous Match First Match

Alignment statistics for match #3

| Score | Expect | Method | Identities | Positives | Gaps |
|---|---|---|---|---|---|
| 28.0 bits(56) | 178 | Composition-based stats. | 16/46(35%) | 22/46(47%) | 2/46(4%) |

```
Query  10   LNAEKASTNSETNRGESEKKRNLGELSRTTSEDNEVFGEADANQNN  55
            L +E      +E NR  +E  R L E +R T E   V  EA+ N+
Sbjct  192  LKSEAERKEAENNRKVAESNRKLAENTRKTNEAARV--EAELNRQS  235
```

Range 4: 282 to 334GenPeptGraphics Next Match Previous Match First Match

Alignment statistics for match #4

| Score | Expect | Method | Identities | Positives | Gaps |
|---|---|---|---|---|---|
| 27.5 bits(55) | 217 | Composition-based stats. | 17/53(32%) | 25/53(47%) | 7/53(13%) |

```
Query  5    AGKRELNAEKASTNSETNRGESEKKR-------NLGELSRTTSEDNEVFGEAD  50
            A   + + AEKA  N+E  R ++E +R       N  E+ R   E N V  E +
Sbjct  282  AEDKRVTAEKARANAEEERIKAETRRENTYTDFNDSEVERKNNEANRVRAETE  334
```

Download GenPeptGraphics Next Previous Descriptions

cell wall-binding protein [Clostridium perfringens]
Sequence ID: WP_265869768.1Length: 1627Number of Matches: 1

- See 2 more title(s) See all Identical Proteins(IPG)

Related Information
Identical Proteins-Identical proteins to WP_265869768.1
Range 1: 172 to 253GenPeptGraphics Next Match Previous Match

Alignment statistics for match #1

| Score | Expect | Method | Identities | Positives | Gaps |
|---|---|---|---|---|---|
| 31.4 bits(64) | 16 | Composition-based stats. | 26/93(28%) | 39/93(41%) | 28/93(30%) |

```
Query  3    NHAGKRELNAEK-ASTNSETNRGESEKKRNLGELSRTTSEDNEVFGEADANQ--------  53
            N   K E+ +E+   S + ET + + E K+   GE         + EV   + D NQ
Sbjct  172  NRDKKVEVKSEEIKSKDNETSKRDEESKKIKGE-------NKEVESKLDKNQALKSDYIN  224

Query  54   --------NNGTSSQDTAVTDSKRTADPKNAWQ  78
                      GT SQ+ +VTD  +     K+ WQ
Sbjct  225  INESGQALTAGTESQNNSVTDENK----KDGWQ  253
```

Download GenPeptGraphics Next Previous Descriptions
cell wall-binding protein [Clostridium perfringens]
Sequence ID: MDK0928209.1Length: 1558Number of Matches: 1
Range 1: 172 to 253GenPeptGraphics Next Match Previous Match

Alignment statistics for match #1

| Score | Expect | Method | Identities | Positives | Gaps |
|---|---|---|---|---|---|
| 31.4 bits(64) | 16 | Composition-based stats. | 26/86(30%) | 38/86(44%) | 14/86(16%) |

```
Query  3    NHAGKRELNAEK-ASTNSETNRGESEKKRNLGELSRTTS--EDNEVFGEADANQNN----  55
            N   K E+ +E+   S + ET + + E K+   GE      S   + N+         N N
Sbjct  172  NRDKKVEVKSEEIKSKDNETSKRDEESKKIKGENKEVESKLDKNKALKSDYININEYGQA  231

Query  56   ---GTSSQDTAVTDSKRTADPKNAWQ  78
               GT SQ+ +VTD      + KN WQ
Sbjct  232  LTAGTESQNNSVTDE----NEKNGWQ  253
```

Download GenPeptGraphics Next Previous Descriptions
cell wall-binding protein [Clostridium perfringens]
Sequence ID: EIF6296494.1Length: 1558Number of Matches: 1
Range 1: 172 to 253GenPeptGraphics Next Match Previous Match

Alignment statistics for match #1

| Score | Expect | Method | Identities | Positives | Gaps |
|---|---|---|---|---|---|

| | | | | | |
|---|---|---|---|---|---|
| 31.4 bits(64) | 17 | Composition-based stats. | 26/93(28%) | 39/93(41%) | 28/93(30%) |

```
Query  3    NHAGKRELNAEK-ASTNSETNRGESEKKRNLGELSRTTSEDNEVFGEADANQ--------  53
            N   K E+ +E+  S + ET + + E K+  GE       + EV  + D NQ
Sbjct  172  NRDNKVEVKSEEIKSKDNETSKRDEESKKIKGE-------NKEVESKLDKNQALKSDYIN  224

Query  54   --------NNGTSSQDTAVTDSKRTADPKNAWQ  78
                      GT SQ+ +VTD  +    K+ WQ
Sbjct  225  INESGQALTVGTESQNNSVTDENK----KDGWQ  253
```

Download GenPeptGraphics Next Previous Descriptions

hypothetical protein [Clostridium perfringens]

Sequence ID: ELC8413661.1Length: 1215Number of Matches: 1

Range 1: 172 to 253GenPeptGraphics Next Match Previous Match

Alignment statistics for match #1

| Score | Expect | Method | Identities | Positives | Gaps |
|---|---|---|---|---|---|
| 31.4 bits(64) | 17 | Composition-based stats. | 26/86(30%) | 38/86(44%) | 14/86(16%) |

```
Query  3    NHAGKRELNAEK-ASTNSETNRGESEKKRNLGELSRTTS--EDNEVFGEADANQNN----  55
            N   K E+ +E+  S + ET + + E K+  GE     S  + N+       N N
Sbjct  172  NRDKKVEVKSEEIKSKDNETSKRDEESKKIKGENKEVESKLDKNKALKSDYININESGQA  231

Query  56   ---GTSSQDTAVTDSKRTADPKNAWQ  78
               GT SQ+ +VTD     + KN WQ
Sbjct  232  LTVGTESQNNSVTDE----NEKNGWQ  253
```

---

Download GenPeptGraphics Next Previous Descriptions

cell wall-binding protein [Clostridium perfringens]

Sequence ID: WP_003471161.1Length: 1559Number of Matches: 1

- See 3 more title(s) See all Identical Proteins(IPG)

Related Information

Identical Proteins-Identical proteins to WP_003471161.1

Range 1: 173 to 254GenPeptGraphics Next Match Previous Match

Alignment statistics for match #1

| Score | Expect | Method | Identities | Positives | Gaps |
|---|---|---|---|---|---|
| 31.4 bits(64) | 17 | Composition-based stats. | 26/86(30%) | 38/86(44%) | 14/86(16%) |

```
Query  3    NHAGKRELNAEK-ASTNSETNRGESEKKRNLGELSRTTS--EDNEVFGEADANQNN----  55
            N   K E+ +E+  S + ET + + E K+  GE     S  + N+       N N
Sbjct  173  NRDKKVEVRSEEIKSKDNETSKRDEESKKIEGENKEAESKLDKNQALKSDYININESGQA  232
```

```
Query  56    ---GTSSQDTAVTDSKRTADPKNAWQ  78
                GT SQ+ +VTD      + KN WQ
Sbjct  233   LTVGTESQNNSVTDE----NEKNGWQ  254
```

Download GenPeptGraphics Next Previous Descriptions

hypothetical protein [Clostridium perfringens]

Sequence ID: WP_197550111.1Length: 1155Number of Matches: 1

Range 1: 388 to 446GenPeptGraphics Next Match Previous Match

Alignment statistics for match #1

| Score | Expect | Method | Identities | Positives | Gaps |
| --- | --- | --- | --- | --- | --- |
| 31.4 bits(64) | 17 | Composition-based stats. | 20/59(34%) | 28/59(47%) | 6/59(10%) |

```
Query  3     NHAGKRELNAEKASTNSETNRGESEKKRNLGELSRTTS------EDNEVFGEADANQNN  55
             N A +  + AE     SE  R +SEK R L E  R T+       E N  F + + N+N+
Sbjct  388   NDAEELRVTAESERVKSEKLRVDSEKARVLAEEERNTTFTQKEEERNNTFTQNETNRND  446
```

Download GenPeptGraphics

Sort by:

Next Previous Descriptions

hypothetical protein [Clostridium perfringens]

Sequence ID: WP_198616560.1Length: 1207Number of Matches: 2

Range 1: 388 to 446GenPeptGraphics Next Match Previous Match

Alignment statistics for match #1

| Score | Expect | Method | Identities | Positives | Gaps |
| --- | --- | --- | --- | --- | --- |
| 31.4 bits(64) | 17 | Composition-based stats. | 20/59(34%) | 28/59(47%) | 6/59(10%) |

```
Query  3     NHAGKRELNAEKASTNSETNRGESEKKRNLGELSRTTS------EDNEVFGEADANQNN  55
             N A +  + AE     SE  R +SEK R L E  R T+       E N  F + + N+N+
Sbjct  388   NDAEELRVTAESERVKSEKLRVDSEKARVLAEEERNTTFTQKEEERNNTFTQNETNRND  446
```

Range 2: 198 to 232GenPeptGraphics Next Match Previous Match First Match

Alignment statistics for match #2

| Score | Expect | Method | Identities | Positives | Gaps |
| --- | --- | --- | --- | --- | --- |
| 25.8 bits(51) | 763 | Composition-based stats. | 14/37(38%) | 20/37(54%) | 2/37(5%) |

```
Query  19    SETNRGESEKKRNLGELSRTTSEDNEVFGEADANQNN  55
             +E +R  SE+ R L E +R T E   V  EA+ N+
Sbjct  198   AENDRKVSERNRKLSENTRKTNEAARV--EAELNRQS  232
```

Download GenPeptGraphics Next Previous Descriptions

cell wall-binding protein [Clostridium perfringens]

Sequence ID: MDM0911824.1Length: 1281Number of Matches: 1

- See 1 more title(s) See all Identical Proteins(IPG)

Related Information

Identical Proteins-Identical proteins to MDM0911824.1

Range 1: 172 to 253GenPeptGraphics Next Match Previous Match

Alignment statistics for match #1

| Score | Expect | Method | Identities | Positives | Gaps |
|---|---|---|---|---|---|
| 31.4 bits(64) | 17 | Composition-based stats. | 26/86(30%) | 38/86(44%) | 14/86(16%) |

```
Query  3    NHAGKRELNAEK-ASTNSETNRGESEKKRNLGELSRTTS--EDNEVFGEADANQNN----  55
            N   K E+ +E+  S + ET + + E K+  GE     S  + N+        N N
Sbjct  172  NTDKKVEVKSEEIKSKDNETSKRDEESKKIKGENKEVESKLDKNQALKSDYININESGQA  231

Query  56   ---GTSSQDTAVTDSKRTADPKNAWQ  78
               GT SQ+ +VTD      + KN WQ
Sbjct  232  VIAGTESQNNSVTDE----NEKNGWQ  253
```

Download GenPeptGraphics

Sort by:

Next Previous Descriptions

---

hypothetical protein [Clostridium perfringens]

Sequence ID: MDU7110306.1Length: 1008Number of Matches: 4

Range 1: 346 to 404GenPeptGraphics Next Match Previous Match

Alignment statistics for match #1

| Score | Expect | Method | Identities | Positives | Gaps |
|---|---|---|---|---|---|
| 31.4 bits(64) | 18 | Composition-based stats. | 20/59(34%) | 28/59(47%) | 6/59(10%) |

```
Query  3    NHAGKRELNAEKASTNSETNRGESEKKRNLGELSRTTS------EDNEVFGEADANQNN  55
            N A +   + AE      SE   R +SEK R L E  R T+       E N  F + + N+N+
Sbjct  346  NDAEELRVTAESERVKSEKLRVDSEKARVLAEEERNTTFTQKEEERNNTFTQNETNRND  404
```

Range 2: 147 to 190GenPeptGraphics Next Match Previous Match First Match

Alignment statistics for match #2

| Score | Expect | Method | Identities | Positives | Gaps |
|---|---|---|---|---|---|
| 26.7 bits(53) | 397 | Composition-based stats. | 15/46(33%) | 22/46(47%) | 2/46(4%) |

```
Query  10   LNAEKASTNSETNRGESEKKRNLGELSRTTSEDNEVFGEADANQNN  55
            + AE +   +E  R  +E  R L E +R T E   V  EA+ N+
Sbjct  147  IKAETSRVEAENKRKVAESNRKLAENTRKTNEAARV--EAELNRQS  190
```

Range 3: 143 to 179GenPeptGraphics Next Match Previous Match First Match

Alignment statistics for match #3

| Score | Expect | Method | Identities | Positives | Gaps |
|---|---|---|---|---|---|
| 26.7 bits(53) | 416 | Composition-based stats. | 13/37(35%) | 18/37(48%) | 0/37(0%) |

```
Query  13   EKASTNSETNRGESEKKRNLGELSRTTSEDNEVFGEA  49
            E     +ET R E+E KR + E  R  +E+     EA
Sbjct  143  EADRIKAETSRVEAENKRKVAESNRKLAENTRKTNEA  179
```

Range 4: 441 to 496GenPeptGraphics Next Match Previous Match First Match

Alignment statistics for match #4

| Score | Expect | Method | Identities | Positives | Gaps |
|---|---|---|---|---|---|
| 26.7 bits(53) | 485 | Composition-based stats. | 19/56(34%) | 24/56(42%) | 2/56(3%) |

```
Query  12   AEKASTNSETNRGESEKKRNLGELSRTTSEDN--EVFGEADANQNNGTSSQDTAVT  65
            AEK    +ET R E+E  R   E  R  +E    E F + D+  N  T     A T
Sbjct  441  AEKLRVTAETGRVEAENLRATAEEERVAAETKREEGFNKFDSKINANTKELKAART  496
```

Download GenPeptGraphics Next Previous Descriptions

cell wall-binding protein [Clostridium perfringens]

Sequence ID: EGT0691972.1Length: 1559Number of Matches: 1

Range 1: 173 to 254GenPeptGraphics Next Match Previous Match

Alignment statistics for match #1

| Score | Expect | Method | Identities | Positives | Gaps |
|---|---|---|---|---|---|
| 31.0 bits(63) | 19 | Composition-based stats. | 26/86(30%) | 38/86(44%) | 14/86(16%) |

```
Query  3    NHAGKRELNAEK-ASTNSETNRGESEKKRNLGELSRTTS--EDNEVFGEADANQNN----  55
            N   K E+ +E+  S + ET + + E K+  GE     S  + N+       N N
Sbjct  173  NRDKKVEVRSEEIKSKDNETSKRDEESKKIEGENKEVESKLDKNQALKSDYININESGQA  232

Query  56   ---GTSSQDTAVTDSKRTADPKNAWQ  78
               GT SQ+ +VTD     + KN WQ
Sbjct  233  LIVGTESQNNSVTDE----NEKNGWQ  254
```

Download GenPeptGraphics

Sort by:

Next Previous Descriptions

hypothetical protein [Clostridium perfringens]

Sequence ID: WP_224404492.1Length: 1151Number of Matches: 4

- See 1 more title(s) See all Identical Proteins(IPG)

Related Information

Identical Proteins-Identical proteins to WP_224404492.1

Range 1: 384 to 453GenPeptGraphics Next Match Previous Match

Alignment statistics for match #1

| Score | Expect | Method | Identities | Positives | Gaps |
|---|---|---|---|---|---|
| 31.0 bits(63) | 19 | Composition-based stats. | 25/71(35%) | 32/71(45%) | 13/71(18%) |

```
Query  13   EKASTNSETNRGESEKKRNLGELSRTTSEDNEVFGEADANQNNGTSSQ------------  60
            E+A   SE  R E+E+ R   EL R  +E   V GE + N N  T S+
Sbjct  384  EEAFKVSEVARNEAEEGRVAAELLRVEAEKLRVAGEENRN-NTFTQSEEERKTTFEEAEN  442

Query  61   DTAVTDSKRTA  71
            D  V +SKR A
Sbjct  443  DRKVAESKRVA  453
```

Range 2: 167 to 221GenPeptGraphics Next Match Previous Match First Match

Alignment statistics for match #2

| Score | Expect | Method | Identities | Positives | Gaps |
|---|---|---|---|---|---|
| 30.6 bits(62) | 34 | Composition-based stats. | 22/61(36%) | 31/61(50%) | 6/61(9%) |

```
Query  8    RELNAEKASTNSETNRGESEKKRNLGELSRTTSEDNEVFGEADANQNNGTSSQDTAVTDS  67
            RE N EKA    SE  R    E++R    EL R  +E+N     E+      N    S++T VT+
Sbjct  167  REKN-EKARLQSEDIRITEEEERVANELERIKAENNRKVSES-----NRKVSENTRVTNE  220

Query  68   K  68
            +
Sbjct  221  R  221
```

Range 3: 261 to 288GenPeptGraphics Next Match Previous Match First Match

Alignment statistics for match #3

| Score | Expect | Method | Identities | Positives | Gaps |
|---|---|---|---|---|---|
| 27.1 bits(54) | 337 | Composition-based stats. | 14/29(48%) | 18/29(62%) | 1/29(3%) |

```
Query  14   KASTNSETNRGESEKKRNLGELSRTTSED  42
            KA T+ E NR E+E KR   E +R  +ED
Sbjct  261  KAKTD-EVNRKEAETKRVEAEKTRVIAED  288
```

Range 4: 260 to 310GenPeptGraphics Next Match Previous Match First Match

Alignment statistics for match #4

| Score | Expect | Method | Identities | Positives | Gaps |
|---|---|---|---|---|---|
| 25.8 bits(51) | 769 | Composition-based stats. | 20/56(36%) | 26/56(46%) | 5/56(8%) |

```
Query  3    NHAGKRELNAEKASTNSETNRGESEKKRNLGELSRTTSEDNEVFGEADANQNNGTS  58
            N A   E+N ++A    ET R E+EK R + E  R  +E      E D  QN  T
Sbjct  260  NKAKTDEVNRKEA----ETKRVEAEKTRVIAEDERKKAETSRQEAE-DIRQNTYTN  310
```

Download GenPeptGraphics Next Previous Descriptions
cell wall-binding protein [Clostridium perfringens]
Sequence ID: WP_143830898.1Length: 1562Number of Matches: 1

- See 2 more title(s) See all Identical Proteins(IPG)

Related Information
Identical Proteins-Identical proteins to WP_143830898.1
Range 1: 172 to 253GenPeptGraphics Next Match Previous Match

Alignment statistics for match #1

| Score | Expect | Method | Identities | Positives | Gaps |
|---|---|---|---|---|---|
| 31.0 bits(63) | 19 | Composition-based stats. | 26/86(30%) | 38/86(44%) | 14/86(16%) |

```
Query  3    NHAGKRELNAEK-ASTNSETNRGESEKKRNLGELSRTTS--EDNEVFGEADANQNN----  55
            N   K E+ +E+  S + ET + + E K+  GE     S  + N+        N N
Sbjct  172  NRDKKVEVKSEEIKSKDNETSKRDEESKKIKGENKEVESKLDKNKALKSDYININESGQA  231


Query  56   ---GTSSQDTAVTDSKRTADPKNAWQ  78
               GT SQ+ +VTD      + KN WQ
Sbjct  232  LTVGTESQNNSVTDE----NEKNGWQ  253
```

Download GenPeptGraphics Next Previous Descriptions
cell wall-binding protein [Clostridium perfringens]
Sequence ID: WP_221389100.1Length: 1558Number of Matches: 1

- See 1 more title(s) See all Identical Proteins(IPG)

Related Information
Identical Proteins-Identical proteins to WP_221389100.1
Range 1: 174 to 249GenPeptGraphics Next Match Previous Match

Alignment statistics for match #1

| Score | Expect | Method | Identities | Positives | Gaps |
|---|---|---|---|---|---|
| 31.0 bits(63) | 20 | Composition-based stats. | 24/87(28%) | 35/87(40%) | 27/87(31%) |

```
Query  8    RELNAEKASTNSETNRGESEKKRNLGELSRTTSEDNEVFGEADANQ--------------  53
            R+    E  S + ET + + E K+  GE        + EV   + D NQ
Sbjct  174  RDKKVEVKSKDNETSKRDEESKKIKGE-------NKEVESKLDKNQALKSDYININESGQ  226


Query  54   --NNGTSSQDTAVTDSKRTADPKNAWQ  78
```

```
                GT SQ+ +VTD  +    K+ WQ
Sbjct  227  ALTVGTESQNNSVTDENK----KDGWQ  249
```

Download GenPeptGraphics Next Previous Descriptions

cell wall-binding protein [Clostridium perfringens]

Sequence ID: MBO3379752.1Length: 1558Number of Matches: 1

Range 1: 174 to 249GenPeptGraphics Next Match Previous Match

Alignment statistics for match #1

| Score | Expect | Method | Identities | Positives | Gaps |
|---|---|---|---|---|---|
| 31.0 bits(63) | 20 | Composition-based stats. | 23/80(29%) | 34/80(42%) | 13/80(16%) |

```
Query  8    RELNAEKASTNSETNRGESEKKRNLGELSRTTS--EDNEVFGEADANQNN-------GTS  58
            R+   E  S + ET + + E K+  GE     S  + N+       N N        GT
Sbjct  174  RDKKVEVKSKDNETSKRDEESKKIKGENKEVESKLDKNQALKSDYININESGQALTVGTE  233

Query  59   SQDTAVTDSKRTADPKNAWQ  78
            SQ+ +VTD  +    K+ WQ
Sbjct  234  SQNNSVTDENK----KDGWQ  249
```

Download GenPeptGraphics Next Previous Descriptions

cell wall-binding protein [Clostridium perfringens]

Sequence ID: WP_131336839.1Length: 1558Number of Matches: 1

- See 1 more title(s) See all Identical Proteins(IPG)

Related Information

Identical Proteins-Identical proteins to WP_131336839.1

Range 1: 174 to 249GenPeptGraphics Next Match Previous Match

Alignment statistics for match #1

| Score | Expect | Method | Identities | Positives | Gaps |
|---|---|---|---|---|---|
| 31.0 bits(63) | 21 | Composition-based stats. | 24/87(28%) | 35/87(40%) | 27/87(31%) |

```
Query  8    RELNAEKASTNSETNRGESEKKRNLGELSRTTSEDNEVFGEADANQ--------------  53
            R+   E  S + ET + + E K+  GE       + EV  + D NQ
Sbjct  174  RDKKVEVKSKDNETSKRDEESKKIKGE-------NKEVESKLDKNQALKSDYININESGQ  226

Query  54   --NNGTSSQDTAVTDSKRTADPKNAWQ  78
                GT SQ+ +VTD  +    K+ WQ
Sbjct  227  ALTVGTESQNNSVTDENK----KDGWQ  249
```

Download GenPeptGraphics Next Previous Descriptions

PTS transporter subunit IIBC [Clostridium perfringens]
Sequence ID: WP_096515033.1Length: 551Number of Matches: 1

- See 4 more title(s) See all Identical Proteins(IPG)

Related Information
Identical Proteins-Identical proteins to WP_096515033.1
Range 1: 450 to 517GenPeptGraphics Next Match Previous Match

Alignment statistics for match #1

| Score | Expect | Method | Identities | Positives | Gaps |
| --- | --- | --- | --- | --- | --- |
| 27.1 bits(54) | 334 | Compositional matrix adjust. | 23/79(29%) | 34/79(43%) | 11/79(13%) |

```
Query  1    MGNHAGKRELNAEKASTNSETNRGESEKKRNLGELSRTTSEDNEVFGEADANQNNGTSSQ  60
            MGN+    + NA KAS+NS     E   K     +     ++N V  + DA      +
Sbjct  450  MGNYIDNEDENANKASSNSNASMDELAVK----AIELLGGKENIV--DVDACM----TRL  499

Query  61   DTAVTDSKRTADPKNAWQD  79
               V D ++  D K AW+D
Sbjct  500  RVTVKDIEKVGDEK-AWKD  517
```

---

Download GenPeptGraphics Next Previous Descriptions
PTS transporter subunit IIBC [Clostridium perfringens]
Sequence ID: WP_003480115.1Length: 551Number of Matches: 1

- See 5 more title(s) See all Identical Proteins(IPG)

Related Information
Identical Proteins-Identical proteins to WP_003480115.1
Range 1: 450 to 517GenPeptGraphics Next Match Previous Match

Alignment statistics for match #1

| Score | Expect | Method | Identities | Positives | Gaps |
| --- | --- | --- | --- | --- | --- |
| 27.1 bits(54) | 334 | Compositional matrix adjust. | 23/79(29%) | 34/79(43%) | 11/79(13%) |

```
Query  1    MGNHAGKRELNAEKASTNSETNRGESEKKRNLGELSRTTSEDNEVFGEADANQNNGTSSQ  60
            MGN+    + NA KAS+NS     E   K     +     ++N V  + DA      +
Sbjct  450  MGNYIDNEDENANKASSNSNASMDELAVK----AIELLGGKENIV--DVDACM----TRL  499

Query  61   DTAVTDSKRTADPKNAWQD  79
               V D ++  D K AW+D
Sbjct  500  RVTVKDIEKVGDEK-AWKD  517
```

Download GenPeptGraphics Next Previous Descriptions
hypothetical protein [Clostridium perfringens]

Sequence ID: MDM0469376.1Length: 1292Number of Matches: 1

Range 1: 232 to 258GenPeptGraphics Next Match Previous Match

Alignment statistics for match #1

| Score | Expect | Method | Identities | Positives | Gaps |
|---|---|---|---|---|---|
| 27.1 bits(54) | 335 | Composition-based stats. | 12/31(39%) | 17/31(54%) | 4/31(12%) |

```
Query  48   EADANQNNGTSSQDTAVTDSKRTADPKNAWQ  78
            EA+      GT SQ+ +VTD   +     K+ WQ
Sbjct  232  EAEQAVTTGTESQNNSVTDENK----KDGWQ  258
```

Download GenPeptGraphics Next Previous Descriptions

ABC transporter ATP-binding protein/permease [Clostridium perfringens]

Sequence ID: MDM1012966.1Length: 575Number of Matches: 1

- See 2 more title(s) See all Identical Proteins(IPG)

Related Information

Identical Proteins-Identical proteins to MDM1012966.1

Range 1: 438 to 545GenPeptGraphics Next Match Previous Match

Alignment statistics for match #1

| Score | Expect | Method | Identities | Positives | Gaps |
|---|---|---|---|---|---|
| 27.1 bits(54) | 335 | Compositional matrix adjust. | 31/110(28%) | 49/110(44%) | 24/110(21%) |

```
Query  3    NHAGKRE-----LNAEKASTNSETNRGESE----KKRNLGELSRTTSEDNEV--FGEADA  51
            N+A K+       +N++K    + E    G S+     +K+ L   L+RT    + EV   F EA +
Sbjct  438  NYALKKANLLDFVNSKKEKLDLEVGEGGSKLSGGQKQRLA-LARTILANREVIIFDEATS  496

Query  52   NQN-----------NGTSSQDTAVTDSKRTADPKNAWQDAHPADPGSRPH  90
            N +              N   S + TA+   S R A+ KNA     +   D G+
Sbjct  497  NIDVESENMIWKSINEISKEKTAIVISHRLANVKNA-DRIYMLDKGALVE  545
```

Download GenPeptGraphics

Sort by:

Next Previous Descriptions

hypothetical protein [Clostridium perfringens]

Sequence ID: WP_224391364.1Length: 1148Number of Matches: 2

- See 1 more title(s) See all Identical Proteins(IPG)

Related Information

Identical Proteins-Identical proteins to WP_224391364.1

Range 1: 261 to 288GenPeptGraphics Next Match Previous Match

Alignment statistics for match #1

| Score | Expect | Method | Identities | Positives | Gaps |
|---|---|---|---|---|---|
| 27.1 bits(54) | 336 | Composition-based stats. | 14/29(48%) | 18/29(62%) | 1/29(3%) |

```
Query  14    KASTNSETNRGESEKKRNLGELSRTTSED  42
             KA T+ E NR E+E KR   E +R  +ED
Sbjct  261   KAKTD-EVNRKEAETKRVEAEKTRVIAED  288
```

Range 2: 260 to 310GenPeptGraphics Next Match Previous Match First Match

Alignment statistics for match #2

| Score | Expect | Method | Identities | Positives | Gaps |
|---|---|---|---|---|---|
| 25.8 bits(51) | 762 | Composition-based stats. | 20/56(36%) | 26/56(46%) | 5/56(8%) |

```
Query  3     NHAGKRELNAEKASTNSETNRGESEKKRNLGELSRTTSEDNEVFGEADANQNNGTS  58
             N A   E+N ++A    ET R E+EK R + E  R  +E       E D  QN  T
Sbjct  260   NKAKTDEVNRKEA----ETKRVEAEKTRVIAEDERKKAETSRQEAE-DIRQNTYTN  310
```

Download GenPeptGraphics Next Previous Descriptions

ABC transporter ATP-binding protein/permease [Clostridium perfringens]

Sequence ID: MDM1004110.1Length: 575Number of Matches: 1

Range 1: 438 to 545GenPeptGraphics Next Match Previous Match

Alignment statistics for match #1

| Score | Expect | Method | Identities | Positives | Gaps |
|---|---|---|---|---|---|
| 27.1 bits(54) | 342 | Compositional matrix adjust. | 31/110(28%) | 49/110(44%) | 24/110(21%) |

```
Query  3     NHAGKRE-----LNAEKASTNSETNRGESE----KKRNLGELSRTTSEDNEV--FGEADA  51
             N+A K+       +N++K   + E    G S+     +K+ L   L+RT   + EV   F EA +
Sbjct  438   NYALKKANLLDFVNSKKEKLDLEVGEGGSKLSGGQKQRLA-LARTILANREVIIFDEATS  496

Query  52    NQN----------NGTSSQDTAVTDSKRTADPKNAWQDAHPADPGSRPH  90
             N +             N  S + TA+  S R A+ KNA    +  D G+
Sbjct  497   NIDVESENMIWKSINEISKEKTAIVISHRLANVKNA-DRIYMLDKGALVE  545
```

Download GenPeptGraphics Next Previous Descriptions

ABC transporter ATP-binding protein/permease [Clostridium perfringens]

Sequence ID: MDM0981024.1Length: 575Number of Matches: 1

- See 1 more title(s) See all Identical Proteins(IPG)

Related Information

Identical Proteins-Identical proteins to MDM0981024.1

Range 1: 438 to 545GenPeptGraphics Next Match Previous Match

Alignment statistics for match #1

| Score | Expect | Method | Identities | Positives | Gaps |
| --- | --- | --- | --- | --- | --- |
| 27.1 bits(54) | 342 | Compositional matrix adjust. | 31/110(28%) | 49/110(44%) | 24/110(21%) |

```
Query  3    NHAGKRE-----LNAEKASTNSETNRGESE----KKRNLGELSRTTSEDNEV--FGEADA  51
            N+A K+       +N++K    + E    G S+     +K+ L   L+RT    + EV   F EA +
Sbjct  438  NYALKKANLLDFVNSKKEKLDLEVGEGGSKLSGGQKQRLA-LARTILANREVIIFDEATS  496

Query  52   NQN-----------NGTSSQDTAVTDSKRTADPKNAWQDAHPADPGSRPH  90
            N +              N   S + TA+   S R A+ KNA     +   D G+
Sbjct  497  NIDVESENMIWKSINEISKEKTAIVISHRLANVKNA-DRIYMLDKGALVE  545
```

[Download](#) [GenPeptGraphics](#) Next Previous [Descriptions](#)

PTS transporter subunit IIBC [Clostridium perfringens]

Sequence ID: [WP_197926616.1](#)Length: 551Number of Matches: 1

- [See 2 more title(s)](#) [See all Identical Proteins(IPG)](#)

Related Information

[Identical Proteins](#)-Identical proteins to WP_197926616.1

Range 1: 450 to 517[GenPeptGraphics](#) Next Match Previous Match

Alignment statistics for match #1

| Score | Expect | Method | Identities | Positives | Gaps |
| --- | --- | --- | --- | --- | --- |
| 27.1 bits(54) | 344 | Compositional matrix adjust. | 23/79(29%) | 34/79(43%) | 11/79(13%) |

```
Query  1    MGNHAGKRELNAEKASTNSETNRGESEKKRNLGELSRTTSEDNEVFGEADANQNNGTSSQ  60
            MGN+      + NA  KAS+NS       E    K        +        ++N  V   + DA         +
Sbjct  450  MGNYIDNEDENANKASSNSNASMDELAVK----AIELLGGKENIV--DVDACM----TRL  499

Query  61   DTAVTDSKRTADPKNAWQD  79
               V  D ++   D K AW+D
Sbjct  500  RVTVKDIEKVGDEK-AWKD  517
```

[Download](#) [GenPeptGraphics](#) Next Previous [Descriptions](#)

PTS transporter subunit IIBC [Clostridium perfringens]

Sequence ID: [WP_283696422.1](#)Length: 551Number of Matches: 1

Range 1: 450 to 517[GenPeptGraphics](#) Next Match Previous Match

Alignment statistics for match #1

| Score | Expect | Method | Identities | Positives | Gaps |
| --- | --- | --- | --- | --- | --- |
| 27.1 bits(54) | 347 | Compositional matrix adjust. | 23/79(29%) | 34/79(43%) | 11/79(13%) |

```
Query  1    MGNHAGKRELNAEKASTNSETNRGESEKKRNLGELSRTTSEDNEVFGEADANQNNGTSSQ  60
            MGN+      + NA  KAS+NS       E    K        +        ++N  V   + DA         +
Sbjct  450  MGNYIDNEDENANKASSNSNASMDELAVK----AIELLGGKENIV--DVDACM----TRL  499
```

```
Query  61   DTAVTDSKRTADPKNAWQD  79
               V D ++  D K AW+D
Sbjct  500  RVTVKDIEKVGDEK-AWKD  517
```

[Download](#) [GenPept](#)[Graphics](#) Next Previous [Descriptions](#)

PTS transporter subunit IIBC [Clostridium perfringens]

Sequence ID: [WP_283694064.1](#)Length: 551Number of Matches: 1

Range 1: 450 to 517[GenPept](#)[Graphics](#) Next Match Previous Match

Alignment statistics for match #1

| Score | Expect | Method | Identities | Positives | Gaps |
|---|---|---|---|---|---|
| 27.1 bits(54) | 347 | Compositional matrix adjust. | 23/79(29%) | 34/79(43%) | 11/79(13%) |

```
Query  1    MGNHAGKRELNAEKASTNSETNRGESEKKRNLGELSRTTSEDNEVFGEADANQNNGTSSQ  60
            MGN+     + NA  KAS+NS      E    K      +      ++N  V   + DA        +
Sbjct  450  MGNYIDNEDENANKASSNSNASMDELAVK----AIELLGGKENIV--DVDACM----TRL  499

Query  61   DTAVTDSKRTADPKNAWQD  79
               V D ++  D K AW+D
Sbjct  500  RVTVKDIEKVGDEK-AWKD  517
```

[Download](#) [GenPept](#)[Graphics](#) Next Previous [Descriptions](#)

ABC transporter ATP-binding protein/permease [Clostridium perfringens]

Sequence ID: [MDM0970125.1](#)Length: 575Number of Matches: 1

Range 1: 438 to 545[GenPept](#)[Graphics](#) Next Match Previous Match

Alignment statistics for match #1

| Score | Expect | Method | Identities | Positives | Gaps |
|---|---|---|---|---|---|
| 27.1 bits(54) | 349 | Compositional matrix adjust. | 31/110(28%) | 49/110(44%) | 24/110(21%) |

```
Query  3    NHAGKRE-----LNAEKASTNSETNRGESE----KKRNLGELSRTTSEDNEV--FGEADA  51
            N+A K+      +N++K   + E   G S+     +K+ L   L+RT   + EV   F EA +
Sbjct  438  NYALKKANLLDFVNSKKEKLDLEVGEGGSKLSGGQKQRLA-LARTILANREVIIFDEATS  496

Query  52   NQN-----------NGTSSQDTAVTDSKRTADPKNAWQDAHPADPGSRPH  90
            N +              N  S + TA+  S R A+ KNA     +   D G+
Sbjct  497  NIDVESENMIWKSINEISKEKTAIVISHRLANVKNA-DRIYMLDKGALVE  545
```

[Download](#) [GenPept](#)[Graphics](#) Next Previous [Descriptions](#)

ABC transporter ATP-binding protein/permease [Clostridium perfringens]

Sequence ID: [MDK0766872.1](#)Length: 575Number of Matches: 1

Range 1: 438 to 545[GenPept](#)[Graphics](#) Next Match Previous Match

Alignment statistics for match #1

| Score | Expect | Method | Identities | Positives | Gaps |
| --- | --- | --- | --- | --- | --- |
| 27.1 bits(54) | 349 | Compositional matrix adjust. | 31/110(28%) | 49/110(44%) | 24/110(21%) |

```
Query  3    NHAGKRE-----LNAEKASTNSETNRGESE----KKRNLGELSRTTSEDNEV--FGEADA  51
            N+A K+       +N++K    + E    G S+     +K+ L    L+RT    + EV   F EA +
Sbjct  438  NYALKKANLLDFVNSKKEKLDLEVGEGGSKLSGGQKQRLA-LARTILANREVIIFDEATS  496

Query  52   NQN-----------NGTSSQDTAVTDSKRTADPKNAWQDAHPADPGSRPH  90
            N +              N  S + TA+  S R A+ KNA     +  D G+
Sbjct  497  NIDVESENMIWKSINEISKEKTAIVISHRLANVKNA-DRIYMLDKGALVE  545
```

Download GenPeptGraphics Next Previous Descriptions

PTS transporter subunit IIBC [Clostridium perfringens]

Sequence ID: WP_271803808.1Length: 551Number of Matches: 1

- See 3 more title(s) See all Identical Proteins(IPG)

Related Information

Identical Proteins-Identical proteins to WP_271803808.1

Range 1: 450 to 517GenPeptGraphics Next Match Previous Match

Alignment statistics for match #1

| Score | Expect | Method | Identities | Positives | Gaps |
| --- | --- | --- | --- | --- | --- |
| 27.1 bits(54) | 351 | Compositional matrix adjust. | 23/79(29%) | 34/79(43%) | 11/79(13%) |

```
Query  1    MGNHAGKRELNAEKASTNSETNRGESEKKRNLGELSRTTSEDNEVFGEADANQNNGTSSQ  60
            MGN+      + NA KAS+NS       E    K       +       ++N V    + DA        +
Sbjct  450  MGNYIDNEDENANKASSNSNASMDELAVK----AIELLGGKENIV--DVDACM----TRL  499

Query  61   DTAVTDSKRTADPKNAWQD  79
               V D ++   D K AW+D
Sbjct  500  RVTVKDIEKVGDEK-AWKD  517
```

Download GenPeptGraphics

Sort by:

Next Previous Descriptions

hypothetical protein [Clostridium perfringens]

Sequence ID: MDM0789937.1Length: 1161Number of Matches: 2

Range 1: 261 to 288GenPeptGraphics Next Match Previous Match

Alignment statistics for match #1

| Score | Expect | Method | Identities | Positives | Gaps |
| --- | --- | --- | --- | --- | --- |
| 27.1 bits(54) | 363 | Composition-based stats. | 14/29(48%) | 18/29(62%) | 1/29(3%) |

```
Query  14    KASTNSETNRGESEKKRNLGELSRTTSED  42
             KA T+ E NR E+E KR   E +R  +ED
Sbjct  261   KAKTD-EVNRKEAETKRVEAEKTRVIAED  288
```

Range 2: 260 to 310GenPeptGraphics Next Match Previous Match First Match

Alignment statistics for match #2

| Score | Expect | Method | Identities | Positives | Gaps |
|---|---|---|---|---|---|
| 25.8 bits(51) | 823 | Composition-based stats. | 20/56(36%) | 26/56(46%) | 5/56(8%) |

```
Query  3     NHAGKRELNAEKASTNSETNRGESEKKRNLGELSRTTSEDNEVFGEADANQNNGTS  58
             N A   E+N ++A    ET R E+EK R + E  R  +E      E D  QN  T
Sbjct  260   NKAKTDEVNRKEA----ETKRVEAEKTRVIAEDERKKAETSRQEAE-DIRQNTYTN  310
```

Download GenPeptGraphics Next Previous Descriptions

PTS transporter subunit IIBC [Clostridium perfringens]

Sequence ID: WP_115613013.1Length: 551Number of Matches: 1

- See 4 more title(s) See all Identical Proteins(IPG)

Related Information

AlphaFold Structure-3D structure displays

Identical Proteins-Identical proteins to WP_115613013.1

Range 1: 450 to 518GenPeptGraphics Next Match Previous Match

Alignment statistics for match #1

| Score | Expect | Method | Identities | Positives | Gaps |
|---|---|---|---|---|---|
| 27.1 bits(54) | 368 | Compositional matrix adjust. | 20/69(29%) | 31/69(44%) | 14/69(20%) |

```
Query  1     MGNHAGKRELNAEKASTNSETNRGESEK--------KRNLGEL------SRTTSEDNEVF  46
             MGN+     + NA  KAS+NS      E              K N+ ++        R T +D E+
Sbjct  450   MGNYIDNEDENANKASSNSNASMDELAVKAIELLGGKENIVDVDACMTRLRVTVKDIEMV  509

Query  47    GEADANQNN  55
             G+   A ++N
Sbjct  510   GDEKAWKDN  518
```

Download GenPeptGraphics Next Previous Descriptions

phenylalanine--tRNA ligase subunit beta [Clostridium perfringens]

Sequence ID: ELC8441778.1Length: 793Number of Matches: 1

Range 1: 614 to 647GenPeptGraphics Next Match Previous Match

Alignment statistics for match #1

| Score | Expect | Method | Identities | Positives | Gaps |
|---|---|---|---|---|---|

26.7 bits(53) 381 Compositional matrix adjust. 12/34(35%) 18/34(52%) 0/34(0%)

```
Query  170  GILDSIGRFFGGDRGAPKRGSGKDSHHPARTAHY  203
            GI++ +     G     + KR SG  S+HP +TA
Sbjct  614  GIVENLLDTLGLKSASFKRMSGNPSYHPGKTAEL  647
```

Download GenPeptGraphics Next Previous Descriptions

PTS transporter subunit EIIC [Clostridium perfringens]

Sequence ID: HAT4113474.1Length: 551Number of Matches: 1

Range 1: 450 to 518GenPeptGraphics Next Match Previous Match

Alignment statistics for match #1

| Score | Expect | Method | Identities | Positives | Gaps |
| --- | --- | --- | --- | --- | --- |
| 26.7 bits(53) | 389 | Compositional matrix adjust. | 20/69(29%) | 31/69(44%) | 14/69(20%) |

```
Query  1    MGNHAGKRELNAEKASTNSETNRGESEK--------KRNLGEL------SRTTSEDNEVF  46
            MGN+     + NA KAS+NS      E            K N+ ++         R T +D E+
Sbjct  450  MGNYIDNEDENANKASSNSNASMDELAVKAIELLGGKENIVDVDACMTRLRVTVKDIEMV  509

Query  47   GEADANQNN  55
            G+  A ++N
Sbjct  510  GDEKAWKDN  518
```

Download GenPeptGraphics Next Previous Descriptions

PTS transporter subunit IIBC [Clostridium perfringens]

Sequence ID: WP_003461077.1Length: 551Number of Matches: 1

- See 3 more title(s) See all Identical Proteins(IPG)

Related Information

AlphaFold Structure-3D structure displays

Identical Proteins-Identical proteins to WP_003461077.1

Range 1: 450 to 517GenPeptGraphics Next Match Previous Match

Alignment statistics for match #1

| Score | Expect | Method | Identities | Positives | Gaps |
| --- | --- | --- | --- | --- | --- |
| 26.7 bits(53) | 404 | Compositional matrix adjust. | 23/79(29%) | 34/79(43%) | 11/79(13%) |

```
Query  1    MGNHAGKRELNAEKASTNSETNRGESEKKRNLGELSRTTSEDNEVFGEADANQNNGTSSQ  60
            MGN+     + NA KAS+NS+     E    K      +       +N  V   + DA        +
Sbjct  450  MGNYIDNEDENANKASSNSKASMDELAVK----AIELLGGRENIV--DVDACM----TRL  499

Query  61   DTAVTDSKRTADPKNAWQD  79
               V D ++   D K AW+D
Sbjct  500  RVTVKDIEKVGDEK-AWKD  517
```

[Download](#) [GenPeptGraphics](#)

Sort by:

---

Next Previous [Descriptions](#)

hypothetical protein [Clostridium perfringens]

Sequence ID: [MDK0590748.1](#)Length: 1147Number of Matches: 2

Range 1: 261 to 288[GenPeptGraphics](#) Next Match Previous Match

Alignment statistics for match #1

| Score | Expect | Method | Identities | Positives | Gaps |
|---|---|---|---|---|---|
| 26.7 bits(53) | 411 | Composition-based stats. | 14/29(48%) | 18/29(62%) | 1/29(3%) |

```
Query  14   KASTNSETNRGESEKKRNLGELSRTTSED  42
            KA T+ E NR E+E KR   E +R  +ED
Sbjct  261  KAKTD-EVNRKEAETKRVEAEKTRVIAED  288
```

Range 2: 260 to 310[GenPeptGraphics](#) Next Match Previous Match [First Match](#)

Alignment statistics for match #2

| Score | Expect | Method | Identities | Positives | Gaps |
|---|---|---|---|---|---|
| 25.8 bits(51) | 904 | Composition-based stats. | 20/56(36%) | 26/56(46%) | 5/56(8%) |

```
Query  3    NHAGKRELNAEKASTNSETNRGESEKKRNLGELSRTTSEDNEVFGEADANQNNGTS  58
            N A   E+N ++A    ET R E+EK R + E  R  +E      E D  QN  T
Sbjct  260  NKAKTDEVNRKEA----ETKRVEAEKTRVIAEDERKKAETSRQEAE-DIRQNTYTN  310
```

[Download](#) [GenPeptGraphics](#) Next Previous [Descriptions](#)

PTS transporter subunit EIIC [Clostridium perfringens]

Sequence ID: [EGS9999252.1](#)Length: 551Number of Matches: 1

Range 1: 450 to 517[GenPeptGraphics](#) Next Match Previous Match

Alignment statistics for match #1

| Score | Expect | Method | Identities | Positives | Gaps |
|---|---|---|---|---|---|
| 26.7 bits(53) | 428 | Compositional matrix adjust. | 23/79(29%) | 34/79(43%) | 11/79(13%) |

```
Query  1    MGNHAGKRELNAEKASTNSETNRGESEKKRNLGELSRTTSEDNEVFGEADANQNNGTSSQ  60
            MGN+    + NA KAS+NS+    E   K     +      +N V  + DA      +
Sbjct  450  MGNYIDNEDENANKASSNSKASMDELAVK----AIELLGGRENIV--DVDACM----TRL  499

Query  61   DTAVTDSKRTADPKNAWQD  79
               V D ++  D K AW+D
Sbjct  500  RVTVKDIEKVGDEK-AWKD  517
```

[Download](#) [GenPeptGraphics](#) Next Previous [Descriptions](#)

FtsX-like permease family protein [Clostridium perfringens]
Sequence ID: WP_283691678.1Length: 1132Number of Matches: 1
Range 1: 363 to 392GenPeptGraphics Next Match Previous Match

Alignment statistics for match #1

| Score | Expect | Method | Identities | Positives | Gaps |
|---|---|---|---|---|---|
| 26.7 bits(53) | 439 | Composition-based stats. | 15/30(50%) | 18/30(60%) | 2/30(6%) |

```
Query  18   NSETNRGE-SEKKRNLGELSRT-TSEDNEV  45
            N E NR E  EK  NL  LS+T TS  N++
Sbjct  363  NPEENRNELNEKIENLKSLSKTITSLSNDI  392
```

Download GenPeptGraphics Next Previous Descriptions
hypothetical protein [Clostridium perfringens]
Sequence ID: WP_198640759.1Length: 1084Number of Matches: 1

- See 2 more title(s) See all Identical Proteins(IPG)

Related Information
Identical Proteins-Identical proteins to WP_198640759.1
Range 1: 261 to 288GenPeptGraphics Next Match Previous Match

Alignment statistics for match #1

| Score | Expect | Method | Identities | Positives | Gaps |
|---|---|---|---|---|---|
| 26.7 bits(53) | 446 | Composition-based stats. | 14/29(48%) | 18/29(62%) | 1/29(3%) |

```
Query  14   KASTNSETNRGESEKKRNLGELSRTTSED  42
            KA T+ E NR E+E KR   E +R  +ED
Sbjct  261  KAKTD-EVNRKEAETKRVEAEKTRVIAED  288
```

Download GenPeptGraphics Next Previous Descriptions
hypothetical protein [Clostridium perfringens]
Sequence ID: MDK0566401.1Length: 69Number of Matches: 1
Range 1: 48 to 57GenPeptGraphics Next Match Previous Match

Alignment statistics for match #1

| Score | Expect | Method | Identities | Positives | Gaps |
|---|---|---|---|---|---|
| 25.4 bits(50) | 474 | Composition-based stats. | 7/10(70%) | 9/10(90%) | 0/10(0%) |

```
Query  230  PRTPPPSQGK  239
            P TPPP++GK
Sbjct  48   PTTPPPAEGK  57
```

Download GenPeptGraphics Next Previous Descriptions
BppU family phage baseplate upper protein [Clostridium perfringens]

---

Sequence ID: MDU3375584.1Length: 967Number of Matches: 1

- See 2 more title(s) See all Identical Proteins(IPG)

Related Information
Identical Proteins-Identical proteins to MDU3375584.1
Range 1: 166 to 201GenPeptGraphics Next Match Previous Match

Alignment statistics for match #1

| Score | Expect | Method | Identities | Positives | Gaps |
|---|---|---|---|---|---|
| 26.7 bits(53) | 484 | Composition-based stats. | 15/38(39%) | 19/38(50%) | 2/38(5%) |

```
Query  18   NSETNRGESEKKRNLGELSRTTSEDNEVFGEADANQNN  55
            N E  R ++E+KRN  E+ R   E  E FG    N  N
Sbjct  166  NQEALREDAERKRNEAEIIR--EEKIEQFGSQVTNITN  201
```

Download GenPeptGraphics Next Previous Descriptions
PTS transporter subunit IIBC [Clostridium perfringens]
Sequence ID: WP_265918137.1Length: 551Number of Matches: 1

- See 1 more title(s) See all Identical Proteins(IPG)

Related Information
Identical Proteins-Identical proteins to WP_265918137.1
Range 1: 450 to 517GenPeptGraphics Next Match Previous Match

Alignment statistics for match #1

| Score | Expect | Method | Identities | Positives | Gaps |
|---|---|---|---|---|---|
| 26.7 bits(53) | 485 | Compositional matrix adjust. | 22/79(28%) | 34/79(43%) | 11/79(13%) |

```
Query  1    MGNHAGKRELNAEKASTNSETNRGESEKKRNLGELSRTTSEDNEVFGEADANQNNGTSSQ  60
            MGN+     + NA KAS+NS      E    K      +      ++N V   + DA       +
Sbjct  450  MGNYIDNEDENANKASSNSNASMDELAVK----AIELLGGKENIV--DVDACM----TRL  499

Query  61   DTAVTDSKRTADPKNAWQD  79
               + D ++   D K AW+D
Sbjct  500  RVTIKDIEKVGDEK-AWKD  517
```

Download GenPeptGraphics Next Previous Descriptions
ATP-binding protein [Clostridium perfringens]
Sequence ID: MDU8978372.1Length: 78Number of Matches: 1
Range 1: 8 to 71GenPeptGraphics Next Match Previous Match

Alignment statistics for match #1

| Score | Expect | Method | Identities | Positives | Gaps |
|---|---|---|---|---|---|

| Score | Expect | Method | Identities | Positives | Gaps |
|---|---|---|---|---|---|
| 25.4 bits(50) | 494 | Compositional matrix adjust. | 19/64(30%) | 25/64(39%) | 6/64(9%) |

```
Query  211   HGRTQDENPVVHFFKNIVTPRTPPPSQGKGRGLSLSRFSWGAEGQRPGF------GYGGR  264
              G   DEN V H F+       T     +  G GL LS    W  + ++  F       G G R
Sbjct  8     NGIGMDENDVPHIFERFFRADTSRVRKNGGTGLGLSIAKWIIDNHKGYFSVLSRKGIGTR  67

Query  265   ASDY  268
              + Y
Sbjct  68    ITVY  71
```

---

Download GenPeptGraphics Next Previous Descriptions

hypothetical protein [Clostridium perfringens]

Sequence ID: EGT0000319.1Length: 488Number of Matches: 1

Range 1: 382 to 424GenPeptGraphics Next Match Previous Match

Alignment statistics for match #1

| Score | Expect | Method | Identities | Positives | Gaps |
|---|---|---|---|---|---|
| 26.2 bits(52) | 503 | Compositional matrix adjust. | 15/44(34%) | 22/44(50%) | 1/44(2%) |

```
Query  41    EDNEVFGEADANQNNGTSSQDTAVTDSKRTADPKNAWQDAHPAD  84
             E N+ F E +AN NNG   +D  + + KR  +  N   D   +D
Sbjct  382   EINKPFEEKEAN-NNGVKKEDNNLKNEKRDENKNNILNDKKISD  424
```

Download GenPeptGraphics Next Previous Descriptions

PTS transporter subunit IIBC [Clostridium perfringens]

Sequence ID: WP_164809571.1Length: 551Number of Matches: 1

- See 5 more title(s) See all Identical Proteins(IPG)

Related Information

Identical Proteins-Identical proteins to WP_164809571.1

Range 1: 450 to 517GenPeptGraphics Next Match Previous Match

Alignment statistics for match #1

| Score | Expect | Method | Identities | Positives | Gaps |
|---|---|---|---|---|---|
| 26.2 bits(52) | 523 | Compositional matrix adjust. | 23/79(29%) | 33/79(41%) | 11/79(13%) |

```
Query  1     MGNHAGKRELNAEKASTNSETNRGESEKKRNLGELSRTTSEDNEVFGEADANQNNGTSSQ  60
             MGN+     + NA KAS+NS      E   K      +        +N V  + DA        +
Sbjct  450   MGNYIDNEDENANKASSNSNASMDELAVK----AIELLGGRENIV--DVDACM----TRL  499

Query  61    DTAVTDSKRTADPKNAWQD  79
                V D ++  D K AW+D
Sbjct  500   RVTVKDIEKVGDEK-AWKD  517
```

Download GenPeptGraphics Next Previous Descriptions
hypothetical protein [Clostridium perfringens]
Sequence ID: EJT6340407.1Length: 967Number of Matches: 1
Range 1: 166 to 201GenPeptGraphics Next Match Previous Match

Alignment statistics for match #1

| Score | Expect | Method | Identities | Positives | Gaps |
|---|---|---|---|---|---|
| 26.2 bits(52) | 532 | Composition-based stats. | 15/38(39%) | 19/38(50%) | 2/38(5%) |

```
Query  18   NSETNRGESEKKRNLGELSRTTSEDNEVFGEADANQNN  55
            N E  R ++E+KRN  E+ R   E  E FG    N  N
Sbjct  166  NQEALREDAERKRNEAEIIR--EEKIEQFGSQVTNITN  201
```

Download GenPeptGraphics Next Previous Descriptions
PTS transporter subunit EIIC [Clostridium perfringens]
Sequence ID: ELC8361772.1Length: 551Number of Matches: 1
Range 1: 450 to 517GenPeptGraphics Next Match Previous Match

Alignment statistics for match #1

| Score | Expect | Method | Identities | Positives | Gaps |
|---|---|---|---|---|---|
| 26.2 bits(52) | 539 | Compositional matrix adjust. | 23/79(29%) | 33/79(41%) | 11/79(13%) |

```
Query  1    MGNHAGKRELNAEKASTNSETNRGESEKKRNLGELSRTTSEDNEVFGEADANQNNGTSSQ  60
            MGN+     + NA KAS+NS      E    K      +      +N V  + DA       +
Sbjct  450  MGNYIDNEDENANKASSNSNASMDELAVK----AIELLGGRENIV--DVDACM----TRL  499

Query  61   DTAVTDSKRTADPKNAWQD  79
               V D ++  D K AW+D
Sbjct  500  RVTVKDIEKVGDEK-AWKD  517
```

Download GenPeptGraphics Next Previous Descriptions
PTS transporter subunit EIIC [Clostridium perfringens]
Sequence ID: HAT4318601.1Length: 551Number of Matches: 1

- See 3 more title(s) See all Identical Proteins(IPG)

Related Information
Identical Proteins-Identical proteins to HAT4318601.1
Range 1: 450 to 517GenPeptGraphics Next Match Previous Match

Alignment statistics for match #1

| Score | Expect | Method | Identities | Positives | Gaps |
|---|---|---|---|---|---|
| 26.2 bits(52) | 539 | Compositional matrix adjust. | 23/79(29%) | 33/79(41%) | 11/79(13%) |

```
Query  1    MGNHAGKRELNAEKASTNSETNRGESEKKRNLGELSRTTSEDNEVFGEADANQNNGTSSQ  60
```

```
             MGN+     + NA  KAS+NS      E    K      +       +N  V   + DA       +
Sbjct  450   MGNYIDNEDENANKASSNSNASMDELAVK----AIELLGGRENIV--DVDACM----TRL  499

Query  61    DTAVTDSKRTADPKNAWQD  79
                V  D ++   D  K AW+D
Sbjct  500   RVTVKDIEKVGDEK-AWKD  517
```

---

Download GenPeptGraphics Next Previous Descriptions

PTS transporter subunit EIIC [Clostridium perfringens]

Sequence ID: HAT4243701.1Length: 551Number of Matches: 1

- See 1 more title(s) See all Identical Proteins(IPG)

Related Information

Identical Proteins-Identical proteins to HAT4243701.1

Range 1: 450 to 517GenPeptGraphics Next Match Previous Match

Alignment statistics for match #1

| Score | Expect | Method | Identities | Positives | Gaps |
|---|---|---|---|---|---|
| 26.2 bits(52) | 554 | Compositional matrix adjust. | 23/79(29%) | 33/79(41%) | 11/79(13%) |

```
Query  1     MGNHAGKRELNAEKASTNSETNRGESEKKRNLGELSRTTSEDNEVFGEADANQNNGTSSQ  60
             MGN+     + NA  KAS+NS      E    K      +       +N  V   + DA       +
Sbjct  450   MGNYIDNEDENANKASSNSNASMDELAVK----AIELLGGRENIV--DVDACM----TRL  499

Query  61    DTAVTDSKRTADPKNAWQD  79
                V  D ++   D  K AW+D
Sbjct  500   RVTVKDIEKVGDEK-AWKD  517
```

Download GenPeptGraphics Next Previous Descriptions

PTS transporter subunit IIBC [Clostridium perfringens]

Sequence ID: WP_003455570.1Length: 551Number of Matches: 1

- See 5 more title(s) See all Identical Proteins(IPG)

Related Information

AlphaFold Structure-3D structure displays

Identical Proteins-Identical proteins to WP_003455570.1

Range 1: 450 to 517GenPeptGraphics Next Match Previous Match

Alignment statistics for match #1

| Score | Expect | Method | Identities | Positives | Gaps |
|---|---|---|---|---|---|
| 26.2 bits(52) | 554 | Compositional matrix adjust. | 23/79(29%) | 33/79(41%) | 11/79(13%) |

```
Query  1     MGNHAGKRELNAEKASTNSETNRGESEKKRNLGELSRTTSEDNEVFGEADANQNNGTSSQ  60
```

```
              MGN+    + NA KAS+NS     E   K      +      +N V  + DA      +
Sbjct  450   MGNYIDNEDENANKASSNSNASMDELAVK----AIELLGGRENIV--DVDACM----TRL  499

Query  61    DTAVTDSKRTADPKNAWQD  79
                V D ++  D K AW+D
Sbjct  500   RVTVKDIEKVGDEK-AWKD  517
```

Download GenPeptGraphics Next Previous Descriptions
hypothetical protein [Clostridium perfringens]
Sequence ID: WP_265881617.1Length: 901Number of Matches: 1

- See 2 more title(s) See all Identical Proteins(IPG)

Related Information
Identical Proteins-Identical proteins to WP_265881617.1
Range 1: 711 to 765GenPeptGraphics Next Match Previous Match

Alignment statistics for match #1

| Score | Expect | Method | Identities | Positives | Gaps |
|---|---|---|---|---|---|
| 26.2 bits(52) | 567 | Compositional matrix adjust. | 19/57(33%) | 28/57(49%) | 2/57(3%) |

```
Query  24    GESEKKRNLGELSRTTSEDNEVFGEADANQNNGTSSQDTAVTDSKRTADPKNAWQDA  80
             GE EK   +  ++R  S  N +   +D NQNN   S+  A  D K      +NA++ A
Sbjct  711   GEIEKYNPIDYITRNQSVFNNLI--SDYNQNNSDISKKIAEQDQKNIKFTENAYEKA  765
```

Download GenPeptGraphics Next Previous Descriptions
phenylalanine--tRNA ligase subunit beta [Clostridium perfringens]
Sequence ID: HAT4189856.1Length: 793Number of Matches: 1
Range 1: 614 to 647GenPeptGraphics Next Match Previous Match

Alignment statistics for match #1

| Score | Expect | Method | Identities | Positives | Gaps |
|---|---|---|---|---|---|
| 26.2 bits(52) | 579 | Compositional matrix adjust. | 12/34(35%) | 18/34(52%) | 0/34(0%) |

```
Query  170   GILDSIGRFFGGDRGAPKRGSGKDSHHPARTAHY  203
             GI++ +    G     + KR SG  S+HP +TA
Sbjct  614   GIVENLLDSLGLKSASFKRMSGNPSYHPGKTAEL  647
```

Download GenPeptGraphics Next Previous Descriptions
PTS transporter subunit IIBC [Clostridium perfringens]
Sequence ID: WP_283717902.1Length: 551Number of Matches: 1
Range 1: 450 to 517GenPeptGraphics Next Match Previous Match

Alignment statistics for match #1

| Score | Expect | Method | Identities | Positives | Gaps |
|---|---|---|---|---|---|
| 26.2 bits(52) | 581 | Compositional matrix adjust. | 23/79(29%) | 33/79(41%) | 11/79(13%) |

```
Query  1    MGNHAGKRELNAEKASTNSETNRGESEKKRNLGELSRTTSEDNEVFGEADANQNNGTSSQ  60
            MGN+    + NA KAS+NS     E   K     +      +N V  + DA      +
Sbjct  450  MGNYIDNEDENANKASSNSNASMDELAVK----AIELLGGRENIV--DVDACM----TRL  499

Query  61   DTAVTDSKRTADPKNAWQD  79
               V D ++  D K AW+D
Sbjct  500  RVTVKDIEKVGDEK-AWKD  517
```

---

Download GenPeptGraphics Next Previous Descriptions

hypothetical protein [Clostridium perfringens]

Sequence ID: ELU5588313.1Length: 1912Number of Matches: 1

Range 1: 373 to 438GenPeptGraphics Next Match Previous Match

Alignment statistics for match #1

| Score | Expect | Method | Identities | Positives | Gaps |
|---|---|---|---|---|---|
| 26.2 bits(52) | 599 | Composition-based stats. | 17/67(25%) | 31/67(46%) | 4/67(5%) |

```
Query  88   RPHLIRLFSRDAPG--REDNTFK-DRPSESDELQTIQEDSAATSESLDVMASQKRPSQRH  144
            RPH++R  + D     + D  F+   P+ ++E   I   S   SE+ D    + R  +
Sbjct  373  RPHIVREWTADVTDDKKTDSKFRISTPTRANETSEIMI-SKTQSEAYDTRTVRDRFEEET  431

Query  145  GSKYLAT  151
            G+ Y+ +
Sbjct  432  GTNYIPS  438
```

Download GenPeptGraphics Next Previous Descriptions

peptidoglycan DD-metalloendopeptidase family protein [Clostridium perfringens]

Sequence ID: MDK0631309.1Length: 399Number of Matches: 1

Range 1: 82 to 125GenPeptGraphics Next Match Previous Match

Alignment statistics for match #1

| Score | Expect | Method | Identities | Positives | Gaps |
|---|---|---|---|---|---|
| 26.2 bits(52) | 607 | Compositional matrix adjust. | 15/44(34%) | 23/44(52%) | 3/44(6%) |

```
Query  8    RELNAEKASTNSETNRGESEKKRNLGELSRTTSED---NEVFGE  48
            + LN E +ST+SE N+ E   + N  E+ +   E     E+ GE
Sbjct  82   KTLNNEISSTDSEINKVEKSIEENTTEIEKAKKEQELKKEILGE  125
```

Download GenPeptGraphics Next Previous Descriptions

peptidoglycan DD-metalloendopeptidase family protein [Clostridium perfringens]

Sequence ID: MDU2827017.1Length: 399Number of Matches: 1

Range 1: 82 to 125GenPeptGraphics Next Match Previous Match

Alignment statistics for match #1

| Score | Expect | Method | Identities | Positives | Gaps |
|---|---|---|---|---|---|
| 26.2 bits(52) | 637 | Compositional matrix adjust. | 15/44(34%) | 23/44(52%) | 3/44(6%) |

```
Query  8     RELNAEKASTNSETNRGESEKKRNLGELSRTTSED---NEVFGE  48
             + LN E +ST+SE N+ E   + N  E+ +   E     E+ GE
Sbjct  82    KTLNNEISSTDSEINKVEKSIEENTTEIEKAKKEQELKQEILGE  125
```

Download GenPeptGraphics Next Previous Descriptions

peptidoglycan DD-metalloendopeptidase family protein [Clostridium perfringens]

Sequence ID: MDM0927148.1Length: 399Number of Matches: 1

Range 1: 82 to 125GenPeptGraphics Next Match Previous Match

Alignment statistics for match #1

| Score | Expect | Method | Identities | Positives | Gaps |
|---|---|---|---|---|---|
| 26.2 bits(52) | 650 | Compositional matrix adjust. | 15/44(34%) | 23/44(52%) | 3/44(6%) |

```
Query  8     RELNAEKASTNSETNRGESEKKRNLGELSRTTSED---NEVFGE  48
             + LN E +ST+SE N+ E   + N  E+ +   E     E+ GE
Sbjct  82    KTLNNEISSTDSEINKVEKSIEENTTEIEKAKKEQELKKEILGE  125
```

Download GenPeptGraphics Next Previous Descriptions

M23 family metallopeptidase [Clostridium perfringens]

Sequence ID: WP_078233194.1Length: 399Number of Matches: 1

- See 5 more title(s) See all Identical Proteins(IPG)

Related Information

AlphaFold Structure-3D structure displays

Identical Proteins-Identical proteins to WP_078233194.1

Range 1: 82 to 125GenPeptGraphics Next Match Previous Match

Alignment statistics for match #1

| Score | Expect | Method | Identities | Positives | Gaps |
|---|---|---|---|---|---|
| 25.8 bits(51) | 681 | Compositional matrix adjust. | 15/44(34%) | 23/44(52%) | 3/44(6%) |

```
Query  8     RELNAEKASTNSETNRGESEKKRNLGELSRTTSED---NEVFGE  48
             + LN E +ST+SE N+ E   + N  E+ +   E     E+ GE
Sbjct  82    KTLNNEISSTDSEINKVEKSIEENTTEIEKAKKEQELKKEILGE  125
```

Download GenPeptGraphics Next Previous Descriptions

FIVAR domain-containing protein [Clostridium perfringens]

Sequence ID: HBC2031549.1Length: 1187Number of Matches: 1

- See 3 more title(s) See all Identical Proteins(IPG)

Related Information
Identical Proteins-Identical proteins to HBC2031549.1
Range 1: 142 to 187GenPeptGraphics Next Match Previous Match

Alignment statistics for match #1

| Score | Expect | Method | Identities | Positives | Gaps |
|---|---|---|---|---|---|
| 26.2 bits(52) | 687 | Composition-based stats. | 14/46(30%) | 23/46(50%) | 3/46(6%) |

```
Query  168  DTGILDSIGRFF---GGDRGAPKRGSGKDSHHPARTAHYGSLPQKS  210
            +TGI+D  G  +     D+G    GS  DS+ P +    G++P  +
Sbjct  142  NTGIVDNEGNLYIWGMNDQGQLGTGSTTDSYRPVKVNGKGAIPTNA  187
```

Download GenPeptGraphics Next Previous Descriptions
SNF2-related protein [Clostridium perfringens]
Sequence ID: WP_265887693.1Length: 1068Number of Matches: 1

- See 1 more title(s) See all Identical Proteins(IPG)

Related Information
Identical Proteins-Identical proteins to WP_265887693.1
Range 1: 852 to 868GenPeptGraphics Next Match Previous Match

Alignment statistics for match #1

| Score | Expect | Method | Identities | Positives | Gaps |
|---|---|---|---|---|---|
| 26.2 bits(52) | 698 | Composition-based stats. | 10/17(59%) | 11/17(64%) | 0/17(0%) |

```
Query  7    KRELNAEKASTNSETNR  23
            K ELNA K S   SE N+
Sbjct  852  KNELNANKVSEKSEKNK  868
```

Download GenPeptGraphics Next Previous Descriptions
SNF2-related protein [Clostridium perfringens]
Sequence ID: WP_240260323.1Length: 1068Number of Matches: 1
Range 1: 852 to 868GenPeptGraphics Next Match Previous Match

Alignment statistics for match #1

| Score | Expect | Method | Identities | Positives | Gaps |
|---|---|---|---|---|---|
| 26.2 bits(52) | 698 | Composition-based stats. | 10/17(59%) | 11/17(64%) | 0/17(0%) |

```
Query  7    KRELNAEKASTNSETNR  23
            K ELNA K S   SE N+
Sbjct  852  KNELNANKVSEKSEKNK  868
```

---

Download GenPeptGraphics Next Previous Descriptions
SNF2-related protein [Clostridium perfringens]
Sequence ID: WP_242976672.1Length: 1068Number of Matches: 1
Range 1: 852 to 868GenPeptGraphics Next Match Previous Match

Alignment statistics for match #1

| Score | Expect | Method | Identities | Positives | Gaps |
|---|---|---|---|---|---|
| 26.2 bits(52) | 698 | Composition-based stats. | 10/17(59%) | 11/17(64%) | 0/17(0%) |

```
Query  7     KRELNAEKASTNSETNR   23
             K ELNA K S  SE N+
Sbjct  852   KNELNANKVSEKSEKNK   868
```

Download GenPeptGraphics Next Previous Descriptions
peptidoglycan DD-metalloendopeptidase family protein [Clostridium perfringens]
Sequence ID: MDM0964256.1Length: 389Number of Matches: 1
Range 1: 72 to 115GenPeptGraphics Next Match Previous Match

Alignment statistics for match #1

| Score | Expect | Method | Identities | Positives | Gaps |
|---|---|---|---|---|---|
| 25.8 bits(51) | 706 | Compositional matrix adjust. | 15/44(34%) | 23/44(52%) | 3/44(6%) |

```
Query  8     RELNAEKASTNSETNRGESEKKRNLGELSRTTSED---NEVFGE  48
             + LN E +ST+SE N+ E    + N  E+ +    E      E+ GE
Sbjct  72    KTLNNEISSTDSEINKVEKSIEENTTEIEKAKKEQELKKEILGE  115
```

---

Download GenPeptGraphics Next Previous Descriptions
DEAD/DEAH box helicase [Clostridium perfringens]
Sequence ID: MDM0473488.1Length: 1069Number of Matches: 1
Range 1: 853 to 869GenPeptGraphics Next Match Previous Match

Alignment statistics for match #1

| Score | Expect | Method | Identities | Positives | Gaps |
|---|---|---|---|---|---|
| 25.8 bits(51) | 718 | Composition-based stats. | 10/17(59%) | 11/17(64%) | 0/17(0%) |

```
Query  7     KRELNAEKASTNSETNR   23
             K ELNA K S  SE N+
Sbjct  853   KNELNANKVSEKSEKNK   869
```

Download GenPeptGraphics Next Previous Descriptions
BglII/BstYI family type II restriction endonuclease [Clostridium perfringens]
Sequence ID: WP_057258391.1Length: 194Number of Matches: 1

- See 2 more title(s) See all Identical Proteins(IPG)

Related Information
AlphaFold Structure-3D structure displays
Identical Proteins-Identical proteins to WP_057258391.1
Range 1: 153 to 188GenPeptGraphics Next Match Previous Match

Alignment statistics for match #1

| Score | Expect | Method | Identities | Positives | Gaps |
|---|---|---|---|---|---|
| 25.8 bits(51) | 724 | Compositional matrix adjust. | 13/36(36%) | 18/36(50%) | 0/36(0%) |

```
Query  191  GKDSHHPARTAHYGSLPQKSHGRTQDENPVVHFFKN  226
            GK S + A T H+G L  +  GR      P++ F  N
Sbjct  153  GKGSSYGASTTHFGKLQPRIDGRCAGGCPILVFAIN  188
```

Download GenPeptGraphics Next Previous Descriptions
DEAD/DEAH box helicase [Clostridium perfringens]
Sequence ID: MDM0493197.1Length: 1069Number of Matches: 1
Range 1: 853 to 869GenPeptGraphics Next Match Previous Match

Alignment statistics for match #1

| Score | Expect | Method | Identities | Positives | Gaps |
|---|---|---|---|---|---|
| 25.8 bits(51) | 725 | Composition-based stats. | 10/17(59%) | 11/17(64%) | 0/17(0%) |

```
Query  7    KRELNAEKASTNSETNR  23
            K ELNA K S  SE N+
Sbjct  853  KNELNANKVSEKSEKNK  869
```

Download GenPeptGraphics Next Previous Descriptions
FtsX-like permease family protein [Clostridium perfringens]
Sequence ID: EGT3616019.1Length: 1132Number of Matches: 1
Range 1: 518 to 576GenPeptGraphics Next Match Previous Match

Alignment statistics for match #1

| Score | Expect | Method | Identities | Positives | Gaps |
|---|---|---|---|---|---|
| 25.8 bits(51) | 769 | Composition-based stats. | 21/60(35%) | 27/60(45%) | 7/60(11%) |

```
Query  2    GNHAGKRELNAEKASTN-SETNRGESEK-----KRNLGELSRTTSEDNEVFGEADANQNN  55
            G   G+RELN  KA   + SE     E EK      K  L E  R   E+ E    +D ++ N
Sbjct  518  GKVTGERELNKAKAKLDESEAQIAEGEKVIAENKEKLAE-GRKEIEEKEKSTLSDLDECN  576
```

Download GenPeptGraphics Next Previous Descriptions
extracellular solute-binding protein [Clostridium perfringens]
Sequence ID: ELC8444128.1Length: 349Number of Matches: 1
Range 1: 301 to 347GenPeptGraphics Next Match Previous Match

Alignment statistics for match #1

| Score | Expect | Method | Identities | Positives | Gaps |
|---|---|---|---|---|---|
| 25.8 bits(51) | 781 | Compositional matrix adjust. | 13/47(28%) | 20/47(42%) | 2/47(4%) |

```
Query  195  HHPARTAHYGSLPQKSHGRTQDENPVVHFFKNIVTPRTP--PPSQGK  239
            + P     + +L         +EN  V++F  IV P  P  P + GK
Sbjct  301  YSPDWIKDFKTLDIDNNVIFDNENKWVQYFNSIVKPEAPTKPVANGK  347
```

---

Download GenPeptGraphics Next Previous Descriptions

ATP-binding cassette domain-containing protein [Clostridium perfringens]

Sequence ID: MDU7110228.1Length: 420Number of Matches: 1

Range 1: 323 to 390GenPeptGraphics Next Match Previous Match

Alignment statistics for match #1

| Score | Expect | Method | Identities | Positives | Gaps |
|---|---|---|---|---|---|
| 25.8 bits(51) | 806 | Compositional matrix adjust. | 21/69(30%) | 30/69(43%) | 14/69(20%) |

```
Query  35   LSRTTSEDNEV--FGEADANQN-----------NGTSSQDTAVTDSKRTADPKNAWQDAH  81
            L+RT   + EV  F EA +N +           N  S + TA+  S R A+ KNA    +
Sbjct  323  LARTILANREVIIFDEATSNIDVESENMIWKSINEISKEKTAIVISHRLANVKNA-DRIY  381

Query  82   PADPGSRPH  90
              D G+
Sbjct  382  MLDKGALVE  390
```

Download GenPeptGraphics Next Previous Descriptions

M23 family metallopeptidase [Clostridium perfringens]

Sequence ID: WP_208376368.1Length: 399Number of Matches: 1

- See 1 more title(s) See all Identical Proteins(IPG)

Related Information

AlphaFold Structure-3D structure displays

Identical Proteins-Identical proteins to WP_208376368.1

Range 1: 82 to 125GenPeptGraphics Next Match Previous Match

Alignment statistics for match #1

| Score | Expect | Method | Identities | Positives | Gaps |
|---|---|---|---|---|---|
| 25.8 bits(51) | 817 | Compositional matrix adjust. | 15/44(34%) | 23/44(52%) | 3/44(6%) |

```
Query  8    RELNAEKASTNSETNRGESEKKRNLGELSRTTSED---NEVFGE  48
            + LN E +ST+SE  + E   + N  E+ +T  E      E+ GE
Sbjct  82   KTLNNEISSTDSEISKVEKSIEENTTEIEKTKKEQELKKEILGE  125
```

Download GenPeptGraphics Next Previous Descriptions

hypothetical protein [Clostridium perfringens]

Sequence ID: EGT3606917.1Length: 349Number of Matches: 1

Range 1: 102 to 175GenPeptGraphics Next Match Previous Match

Alignment statistics for match #1

| Score | Expect | Method | Identities | Positives | Gaps |
|---|---|---|---|---|---|
| 25.8 bits(51) | 835 | Compositional matrix adjust. | 25/75(33%) | 30/75(40%) | 14/75(18%) |

```
Query  229  TPRTPPPSQ---GKGRGLSLSRFSWGAE----GQRPGFGYGG--RASDY----KSAHKGF  275
             P+T  P       G G+ L R S G E     QR  FGYGG    +D+    K A K
Sbjct  102  IPKTTKPKLLYAANGSGVDLVRLS-GNETKKLAQRKTFGYGGYYEITDFLGNPKQAFKDA  160

Query  276  KGVDAQGTLSKIFKL  290
                A   + K F L
Sbjct  161  VSQLANAKMEKFFDL  175
```

Download GenPeptGraphics Next Previous Descriptions

hypothetical protein [Clostridium perfringens]

Sequence ID: EGT3601845.1Length: 233Number of Matches: 1

Range 1: 37 to 77GenPeptGraphics Next Match Previous Match

Alignment statistics for match #1

| Score | Expect | Method | Identities | Positives | Gaps |
|---|---|---|---|---|---|
| 25.4 bits(50) | 945 | Compositional matrix adjust. | 14/41(34%) | 21/41(51%) | 1/41(2%) |

```
Query  10  LNAEKASTNS-ETNRGESEKKRNLGELSRTTSEDNEVFGEA  49
           L+++K   N  + N+G  E   NL E+     E  E+FGE
Sbjct  37  LDSQKVEINNNDINKGIKEMLTNLFEIGDAKIEKQELFGEC  77
```

Download GenPeptGraphics Next Previous Descriptions

LPXTG cell wall anchor domain-containing protein [Clostridium perfringens]

Sequence ID: HBI6996330.1Length: 243Number of Matches: 1

- See 3 more title(s) See all Identical Proteins(IPG)

Related Information

Identical Proteins-Identical proteins to HBI6996330.1

Range 1: 141 to 175GenPeptGraphics Next Match Previous Match

Alignment statistics for match #1

| Score | Expect | Method | Identities | Positives | Gaps |
|---|---|---|---|---|---|
| 25.4 bits(50) | 972 | Compositional matrix adjust. | 12/35(34%) | 22/35(62%) | 1/35(2%) |

```
Query  98   DAPGRED-NTFKDRPSESDELQTIQEDSAATSESL  131
            + PG+E  N +K++ ++ D + T+ +DS    ESL
Sbjct  141  EFPGKEAGNEYKNKKAQVDWIFTVVKDSYGEEESL  175
```

Download GenPeptGraphics Next Previous Descriptions

hypothetical protein [Clostridium perfringens]

Sequence ID: WP_283685184.1Length: 721Number of Matches: 1

Range 1: 429 to 473GenPeptGraphics Next Match Previous Match

Alignment statistics for match #1

| Score | Expect | Method | Identities | Positives | Gaps |
|---|---|---|---|---|---|
| 25.4 bits(50) | 982 | Compositional matrix adjust. | 19/56(34%) | 27/56(48%) | 11/56(19%) |

```
Query  7    KRELNAEKASTNSETNRGESEKKRNLGELSRTTSEDNEVFGEADANQNNGTSSQDT  62
            K  LN+EKAS N + N  ++++           SEDN    E D N  N  S+ +T
Sbjct  429  KEGLNSEKASENGDINNSDTQE-----------SEDNNSTLEQDVNSENSESANET  473
```
